# Supplementary material for: Multiarm multistage randomised controlled trial of inflammatory signal inhibitors (MATIS) for patients hospitalised with COVID-19 pneumonia during the UK pandemic
Source: BMJ Open. 2026 Feb 5;16(2):e100583. doi: 10.1136/bmjopen-2025-100583 (PMC12887464; doi:10.1136/bmjopen-2025-100583)
Supplement: Supplementary data [file bmjopen-16-2-s005.pdf]

Supplementary Appendix 5

Missing data methods for the primary model and results using alternative assumptions

In the primary analysis model, we imputed missing WHO COVID-19 status using multiple imputation under the assumption of missing at random (MAR). That is the probability of being missing is not dependent on the values of unobserved data, conditional on the observed values of the variables included in the analysis model. We imputed *m* datasets using chained equations based on the percentage of participants with incomplete outcomes, where *m* was rounded to the nearest 10.[11] As outcome data were classified as missing in 6% of patients, an m=10 was used.

Variables in the imputation model included baseline WHO COVID-19 grade (3 or 4), age (<65 or ≥65), receipt of systemic corticosteroid and/or receipt of an IL6 inhibitor (at the time of or prior to randomisation into MATIS) and prior COVID vaccination. Auxiliary variables suspected to be associated with the outcome and to be predictors of missingness included receipt of any effective COVID-19 treatments (steroids or IL6 inhibitors) post-randomisation (>1 day post-randomisation) and WHO COVID-19 severity grades at day 1 and day 7.

Convergence issues were encountered when running the imputation model separately by treatment arm (the preferred approach).[11] Missing primary outcomes were therefore imputed using the full dataset with treatment arm included as an additional variable. The final analysis mode; was run omitting prior use of steroids as a covariate due to instability results from zero event counts within strata.

Alternative missing data assumptions were also considered: 1) all participants with missing outcome had poor outcome e.g. all missing >=5, and 2): that all participants with missing outcome had good outcome e.g. all missing <5 were also conducted (Table S5).

Table S6: Primary Outcome Statistical Model Results with Alternative Missingness Assumptions

Grade >=5 by Day 14	Adjusted odds ratio (95% CI; p-value[a])	
	Fostamatinib vs SOC	Ruxolitinib vs SOC
Sensitivity analyses		
Assume all missing have poor outcome	1.08 (0.49 to 2.40); 0.847	0.73 (0.32 to 1.67); 0.453
Assume all missing have good outcome	1.17 (0.51 to 2.69); 0.710	0.63 (0.26 to 1.57); 0.323

[a] p-values are two-sided

[b] adjusted for baseline COVID severity, age category, use of steroid (where model allows), use of IL6 inhibitor and prior COVID vaccination
