# Supplementary material for: Multiarm multistage randomised controlled trial of inflammatory signal inhibitors (MATIS) for patients hospitalised with COVID-19 pneumonia during the UK pandemic
Source: BMJ Open. 2026 Feb 5;16(2):e100583. doi: 10.1136/bmjopen-2025-100583 (PMC12887464; doi:10.1136/bmjopen-2025-100583)
Supplement: Supplementary data [file bmjopen-16-2-s001.pdf]

MATIS TRIAL

Multi-arm trial of Inflammatory Signal Inhibitors for COVID-19

Statistical Analysis Plan II – Formal Interim Analysis and Final Analysis

Version 1.0

Date: 29/11/2021

EudraCT Number: 2020-001750-22

IRAS ID: 282552

[redacted]

[redacted]

[redacted]

[redacted]

[redacted]

[redacted]

[redacted]

Hazell L, *et al. BMJ Open* 2026; 16:e100583. doi: 10.1136/bmjopen-2025-100583

[redacted]

2

Document version history log

Date	Version	Changes made
29/11/2021	1.0	

Table of contents

1. Study summary 7
1.1 Principal research objectives 7
1.1.1 Primary objective 7
1.1.2 Primary outcome of the main study 7
1.1.3 Secondary outcomes of the main study 7
1.2 Trial design 8
1.3 Study population 10
1.3.1 Eligibility criteria 10
1.4 Treatment groups 11
1.4.1 Standard of care 11
1.4.2 Future changes to Standard of Care 11
1.4.3 Ruxolitinib 12
1.4.4 Fostamatinib 12
1.4.5 Treatment duration 12
1.5 Sample size estimation 12
1.5.1 Sample size for formal interim analysis (Stage 1) 13
1.5.2 Sample size for full trial (Stage 2) 13
1.5.3 Informal interim analysis 13
1.6 Randomisation 14
1.7 Blinding 14
1.8 Brief description of proposed analysis 14
1.9 Informal interim analysis outcomes - Completed 29th Jan 2021 15
2. Data analysis plan – data description 17
2.1 Recruitment and participant flow 17
2.2 Baseline characteristics 17
2.3 Co-enrolment 17
2.4 Standard of Care 17
2.5 Withdrawals, loss to follow-up and other missing data 17
2.6 Adherence to allocated treatment and dose summaries 17
2.7 Descriptive statistics for outcome measures 17
2.8 Serious adverse events 18
3. Data analysis plan - Inferential analysis 20
3.1 Primary outcome of the main study - Modified WHO grading 20
3.1.1 Definition of the primary outcome 20
3.1.2 Statistical model at stage 1 20
3.1.3 Statistical model at stage 2 23

3.1.4 Model assessment of stage 2 analysis 25
3.1.5 Stage 2 exploratory analysis for primary outcome 26
3.1.6 Stage 2 subgroup analysis for primary outcome 28
3.2 Secondary outcomes of the main study 28
3.2.1 Severe COVID-19 within 28 days 29
3.2.2 Modified WHO COVID-19 score 29
3.2.3 Components of the modified WHO COVID-19 score 30
3.2.4 Length of stay 31
3.2.5 Continuous laboratory values 31
References 34

STUDY MANAGEMENT

**CHIEF INVESTIGATOR**	Dr Nichola Cooper
	Imperial College Healthcare NHS Trust, Hammersmith Hospital, Du Cane Road, W12 0HS, London
	[redacted]
**SPONSOR**	Imperial College London
	JRCO, Imperial College London and Imperial College Healthcare NHS Trust; Room 215, Level 2, Medical School Building, St Marys Campus
	[redacted]
	[redacted]
**FUNDER**	Imperial Biomedical Research Centre
**CO-INVESTIGATORS**	[redacted]
	[redacted]
	[redacted]
	[redacted]
	[redacted]
	[redacted]
	[redacted]
	[redacted]
	[redacted]
	[redacted]
**STATISTICIANS**	[redacted]
	[redacted]
	[redacted]
	[redacted]

1. Study summary

The MATIS trial is a multi-arm, multi-stage randomised controlled trial (RCT) of ruxolitinib (RUX) and fostamatinib (FOS) for COVID-19 pneumonia. The statistical analysis plan (SAP) is based on protocol version 2.2 which can be seen for full design details.

1.1 Principal research objectives

1.1.1 Primary objective

To evaluate the efficacy of ruxolitinib (RUX) and fostamatinib (FOS) compared to standard of care (SOC) in the treatment of COVID-19 pneumonia in participants with mild disease.

1.1.2 Primary outcome of the main study

The proportion with severe COVID-19 pneumonia within 14 days of randomization. Where severe COVID-19 pneumonia is defined by a modified WHO COVID-19 Ordinal Score ≥ 5 (on the 9 point scale), comprising the following indicators of disease severity:

- Death *OR*
- Requirement for invasive ventilation plus organ support OR
- Requirement for invasive ventilation *OR*
- Requirement for non-invasive ventilation including CPAP or high flow oxygen *OR*
- O_2 saturation < 90% on ≥60% inspired oxygen

1.1.3 Secondary outcomes of the main study

Each of the outcomes below at 14 and 28 days between RUX or FOS versus SOC:

- Change in severity on the modified WHO COVID-19 Ordinal Scale (3 or 4 to 5,6,7,8,9)
- Mortality (Y/N)
- Invasive ventilation (Y/N)
- ECMO (Y/N)
- Non-invasive ventilation including CPAP and high flow nasal oxygen (Y/N)
- Renal replacement therapy (Y/N)
- Venous thromboembolism (Y/N)
- Inflammatory markers CRP, LDH, ferritin, D-dimer (units)
- Length of stay (days)
- Serum creatinine (units).

- Re-admission data – were they readmitted within 28 days? (from free text in SAEs)

1.2 Trial design

A two stage, open label, randomized (1:1:1) controlled trial with formal interim analysis after 171 participants (57 per arm) which also includes an informal interim analysis after 30 participants (10 per arm) complete follow-up. Due to the three different stages in this trial, the MATIS SAP is separated into two sections. The aim of this statistical analysis plan (SAP) is to describe in detail the analysis that will be undertaken for the formal interim analysis and final analysis. The informal interim analysis was to provide an early opportunity to check the dosing regimen, see MATIS SAP I – Dose Verification for a detailed description of the statistical analysis conducted at this stage.

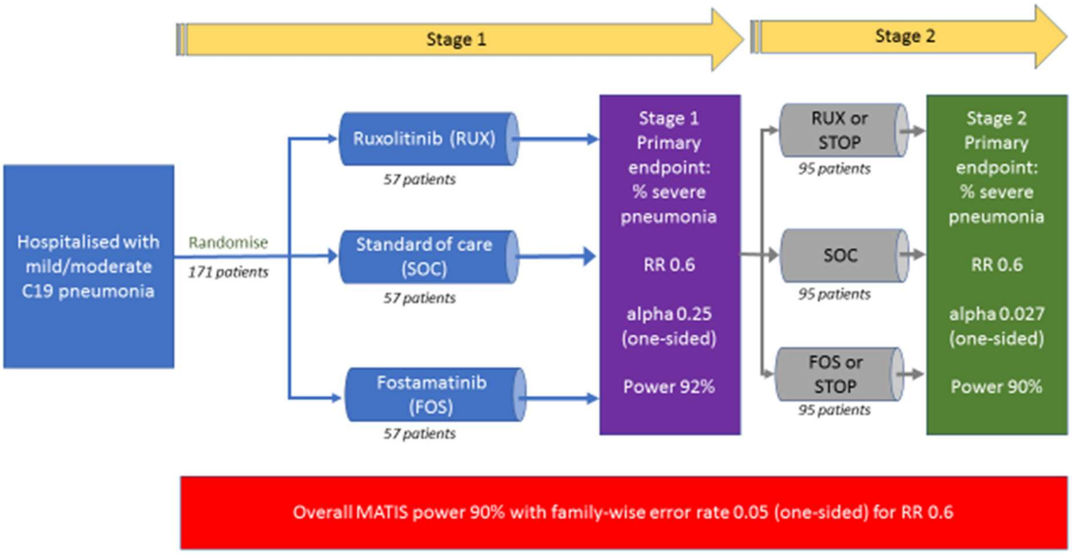

Figure 1: Schematic of study Design

Treatment is for 14 days from baseline. Patients will receive follow-up assessments at 1, 7, 14 and 28 days after the first dose. The time allowed for screening assessments allows time for eligibility assessments to be included.

- Screening Period: up to 7 days to the day of randomisation
- Treatment Period: 14 days
- Follow-up period: Day 14 to 28

Patients who have recovered and are fit for discharge from hospital during the treatment period, will be discharged home with trial medication to complete a fixed 14 day course. Such patients will be followed up weekly with telephone monitoring until day 28 and additional blood test monitoring where practically possible.

Table 1. Study visits

	Screening	Baseline				Last visit
Study Day	***-6 to 0***	***0***	***1[+]***	***7[#]***	***14[#]***	***28[#]***
Time window		-2 days	*+1 day*	*± 3 day*	*± 3 day*	*± 3 day*
Informed consent	x					
Inclusion/Exclusion criteria	x	x				
Demographic data	x					
Medical history including major comorbidities	x					
Physical exam	x					
COVID-19 diagnosis, duration and severity	x					
Pregnancy test	x					
Vital signs	x	x	x	x	x	x
NEWS score	x	x	x	x	x	x
Inflammatory markers (CRP, D-dimer, LDH, ferritin, full blood count)		x	x	x	x	x
PK levels*					x	x
Full blood count[¶]		*Daily while on CYP3A4 inhibitor*				
FiO_2	x	x	x	x	x	x
SpO_2 and/or PaO_2	x	x	x	x	x	x
Randomisation		x				
Weight and Estimated height		x				
Safety monitoring of liver and renal function	x	x	x	x	x	x
Length of stay						
Serious Adverse Events	x	x	x	x	x	x
Concomitant medications	x	x	x	x	x	x
Serum		x		x		x
Plasma		x		x		x
RNA in PAX gene tube		x		x		x
PBMC		x		x		x
Nasosorption biomarkers		x		x		x

[+] data that is routinely collected for clinical purposes will be recorded and a visit is not required
[#] in the event that the patient recovers from COVID19 and is discharged, these visits will not be mandated. Instead a visit should occur on the date of discharge. Where possible, patients will be contacted by telephone at day 7, 14 and 28 and invited to attend for blood tests to monitor resolution of illness.
*Ruxolitinib dose for these patients will be assessed after 10 dialysis patients complete Day 14 through pharmacokinetics (PK) levels which will be assessed.
¶For patients randomized to ruxolitinib and prescribed a strong CYP3A4 inhibitor or dual CYP3A4/CYP2C9 inhibitor. Please refer to Section 7.8 of study protocol v1.9 for details.

9

1.3 Study population

1.3.1 Eligibility criteria

Inclusion criteria

- Patients age ≥ 18 years at screening.
- Patients meeting criteria of:
    - Hospitalization *AND*
    - SARS-CoV2 infection (clinically suspected[1] or laboratory confirmed) *AND*
    - Radiological change consistent with COVID-19 disease
- Mild or moderate (Grade 3 or 4 severity by modified WHO COVID-19 Ordinal Scale) C19 pneumonia.
- CRP ≥ 30mg/L at any time point.
- Informed consent from patient or personal or professional representative.
- No medical history that might, in the opinion of the responsible clinician, put the patient at significant risk if he/she were to participate in the trial.
- Agreement to abstain from sexual intercourse or use contraception that is >99% effective for all participants of childbearing potential for 42 days.[2]
- For male participants, agreement to abstain from sperm donation for 42 days after the last dose of study drug.[3]
- Non-English speakers will be able to join the study. If patients are unable to understand verbal or written information in English - hospital translation services will be requested at the participating site for the participant where possible.
- Patients normally on non-invasive ventilation such as continuous positive airway pressure (CPAP) at home are eligible.

[1] Clinical suspicion of COVID-19 pneumonia will be confirmed by two independent clinicians.
[2] Females must be either post-menopausal for at least 1 year or surgically sterile; or if female of child-bearing potential, must not be pregnant or lactating and must agree to use an acceptable method of birth control throughout the duration of the trial and for 30 days following the last dose. Acceptable methods of birth control are defined as: hormonal contraception (pill, injection or implant) used consistently for at least 30 days prior to enrollment, intrauterine device (IUD), double-barrier (ie, condom and spermicide, or condom and diaphragm), or true abstinence (when this is in line with the preferred and usual lifestyle of the subject).
[3] Male subject with a partner of childbearing potential must agree to use an appropriate double-barrier method of contraception during the time interval between administration of the first dose of study drug and 30 days following the last dose, or must agree to true abstinence (when this is in line with the preferred and usual lifestyle of the subject).

Exclusion criteria

- Requiring either invasive or non-invasive ventilation including CPAP or high flow nasal oxygen at any point after hospital admission and before baseline not related to a pre-existing condition (e.g. obstructive sleep apnoea)
- Grade ≥ 5 severity on the WHO COVID-19 Ordinal Scale.
- O_2 saturation < 90% on ≥ 60% inspired oxygen at baseline.
- In the opinion of the investigator, progression to death is inevitable within the next 24 hours, irrespective of the provision of therapy.
- Any medical condition or concomitant medication that in the opinion of the investigator would compromise subjects' safety or compliance with study procedures.
- Any medical condition which in the opinion of the principal investigator would compromise the scientific integrity of the study.
- Known severe allergic reactions to the investigational agents.
- Use of drugs within the preceding 14 days that are known to interact with any study treatment (FOS or RUX), as listed in the Summary of Product Characteristics.
- Child Pugh B or C grade hepatic dysfunction.
- Pregnant or breast feeding.

1.4 Treatment groups

1.4.1 Standard of care

It is accepted that SOC may change during a rapidly evolving pandemic. Co-enrolment to other trials and rescue therapy, either pre- or post-randomisation, is permitted.

1.4.2 Future changes to Standard of Care

The study team will remain cognisant of the prevailing global literature during the current pandemic on effective therapies for COVID-19 pneumonia. If a treatment emerges with efficacy and safety profile that is superior to SOC then options for adoption of the new therapy as SOC will be discussed with the DMC. Specifically, the impact that the new SOC might have on original trial statistical parameters and outcomes measures will be considered noting that SOC is received by participants in all three arms.

1.4.3 Ruxolitinib

Table 2. Ruxolitinib (RUX)

Period	*Dose*	*Frequency*
Day 1-7	10mg	Twice daily
Day 8-14	5mg	Twice daily

1.4.4 Fostamatinib

Table 3. Fostamatinib (FOS)

Period	*Dose*	*Frequency*
Day 1-7	150mg	Twice daily
Day 8-14	100mg	Twice daily

1.4.5 Treatment duration

The planned duration of treatment is 14 days. Participants may be discontinued from treatment earlier due to unacceptable toxicity or disease progression, which will be recorded as serious adverse events (SAEs). If a participant is discharged prior to day 14 they will go home and continue with the treatment plan.

1.5 Sample size estimation

In the following Stage 1 refers to the formal interim analysis after 171 participants have been recruited and completed 14 days of follow-up. Stage 2 refers to the final analysis when recruitment and follow-up are complete, and it includes all the participants.

171 (57 per arm) patients with COVID-19 pneumonia will be recruited to Stage 1, if the trial progresses to Stage 2 an additional maximum of 285 (95 per arm) will be recruited, resulting in a potential sample size of 456 if three trial arms continue for the whole trial (152 per arm). These numbers have been chosen to provide power of 90% (minimum marginal power) with a maximum 5% chance of an intervention arm being recommended when it provides no improvement over control (5% one-sided family-wise error rate) and has been inflated by 5% for missing outcome data. Patients will be enrolled into RUX, FOS or SOC groups, as shown in figure 1.

The trial is designed to compare two experimental treatment arms to a shared control arm, with equal treatment allocation over two unequally spaced stages. Stage one will be undertaken after 37.5% of maximum sample size have been recruited and completed follow-up. A separate stopping rule will be

used which means the trial will continue until a decision is made for all experimental treatment arms. The sample size in each stage is allowed to vary according to the number of treatments present. The sample size calculation is based on the calculation in the R Shiny app for multi-stage group-sequential multi-arm clinical trials for a Bernoulli distributed primary outcome found here https://mjgrayling.shinyapps.io/multiarm/ but with an inflation to allow for unequal allocation across stages (0.375 of maximum possible sample in stage 1 and 0.625 of sample in stage 2).

1.5.1 Sample size for formal interim analysis (Stage 1)

Current rate of severe pneumonia in the standard of care is 50%. In the case that an experimental arm reduces this to 30% (relative risk, RR, 0.6), then the design provides a 92% chance (one-sided alpha of 0.25, equivalent to stopping for futility if the one-sided p-value is >0.25) of it being recommended to continue to stage 2.

The design recommends stopping an intervention arm at stage 1 if the one-sided p-value is >0.25 but this is a non-binding rule (i.e. the overall power is calculated assuming this rule is always followed and the family-wise error rate (FWER) is calculated assuming it is not followed). The actual FWER will be lower if the rule is always followed).

Recruitment to the trial will continue during this interim analysis stage.

1.5.2 Sample size for full trial (Stage 2)

If the trial is recommended to continue at Stage 2 based on DMC interim review, the full trial will need to recruit an additional 95 for each arm that continues (152 per arm in total; 456 to the complete trial). This will provide 90% power to detect a change of RR of 0.6 (50% to 30%), with one-sided alpha of 0.027 (chosen to give a 5% FWER) and has been inflated by 5% for missing outcome data. Here the power is the chance of an effective treatment passing stage 1 and being recommended after stage 2.

1.5.3 Informal interim analysis

An informal analysis will take place after 10 participants have been recruited to each arm and followed up for 14 days. No hypothesis testing will be performed, the analysis has been set out in “MATIS SAP I Dose Verification Informal Analysis ver1.0”. The DMC will review the data with guidance contained in the SAP I.

Hazell L, *et al. BMJ Open* 2026; 16:e100583. doi: 10.1136/bmjopen-2025-100583

1.6 Randomisation

Patients will be randomized to one of three actively recruiting trial arms, RUX, FOS or SOC. Eligible patients will be allocated using a central web-based randomisation service that uses randomisation sequences with random block sizes that are stratified by age (<65 vs ≥65) and site. If stage 2 occurs with one intervention arm stopped then the randomisation ratio will be 1:1.

1.7 Blinding

The analysis described in this SAP will be undertaken primarily by the trial statistician who will be blind to treatment assignment. The trial statistician will only see sub-group bind data throughout the trial, which means treatment assignment will be coded as group A versus group B versus group C, without knowing which treatments A, B and C refer to. Any analysis of primary efficacy outcomes at the interim analysis stage or data with the potential to unblind the trial statistician (e.g. adherence to allocated intervention) at the interim analysis will be undertaken by an independent statistician from Newcastle University. All final analysis will be undertaken by the blinded statistician who will perform the blinded analysis prior to analysis of any data with the potential to unblind, for example analysis of the adherence data. The senior statistician based in Imperial Clinical Trials Unit will hold the information to unblind the data for closed DMC discussions.

1.8 Brief description of proposed analysis

Efficacy analysis population: We will use the intention-to-treat (ITT) population which includes all randomised participants analysed in the treatment arm to which they were allocated to regardless of treatment subsequently received, multiple imputation will be used to account for any missing data such that all randomised participants are included in the final analysis. At the formal interim analysis multiple imputation will be undertaken if greater than 5% of outcome data are missing, if ≤5% missing a complete case analysis will be undertaken.

Safety analysis population: We will include participants who received at least one dose of study medication and analysis will be according to treatment received, regardless of randomised treatment assignment.

The formal interim analysis will be conducted after 171 participants have been recruited and have completed 14 days of follow-up after randomisation. If an experimental treatment is recommended to continue an additional 95 participants will be recruited for each arm that continues and final analysis will be completed after all participants have completed 28 days of follow-up after randomisation.

Hazell L, *et al. BMJ Open* 2026; 16:e100583. doi: 10.1136/bmjopen-2025-100583

Formal interim and final analyses will be carried out sub-group blind and follow the ITT principle. That is, all randomised patients will be analysed in the treatment arm to which they were allocated to regardless of treatment subsequently received, multiple imputation will be used to include all randomise participants for the final analysis, at the formal interim analysis multiple imputation will be undertaken if greater than 5% of outcome data are missing. The analysis of the serious adverse events will be undertaken on the safety population.

All regression analysis will include adjustment for age group (<65 years or ≥65 years) and site where appropriate, since these were stratification factors in the randomisation.

All confidence intervals will be presented at the 95% level.

For the formal interim analysis (stage 1) one-sided p-values ≤ 0.25 will indicate that an experimental treatment should be recommended to continue to stage 2 and for the final (stage 2) analysis one-sided p-value ≤ 0.027 will be interpreted as statistically significant for the primary outcome.

1.9 Informal interim analysis outcomes - Completed 29th Jan 2021

At the end of the initial stage, the mean CRP values over days 1, 7, 14 and 28 (adjusted for baseline) will be calculated by treatment arm. The mean CRP will also be calculated by treatment arm and visit (adjusted for baseline). These results will form part of the evidence to verify the dose and duration of treatment for the next stage. The pre-specified criteria guiding this inference are listed in full below.

- Dose continues unchanged: For each treatment arm (RUX or FOX) the dose and duration will remain unchanged if there is an absolute mean CRP decrease of approximately 25% or more compared to SOC by day 14 and judged to be maintained by day 28.

- Dose escalation or duration increase: For each treatment arm (RUX or FOX) an adjustment to the dose and duration will be discussed if the mean CRP decrease is less than approximately 25% compared to SOC by day 14.

The results were taken into consideration along with the number of SAEs and SARs and consistency of treatment effect across subgroups including renal failure, use of immunomodulators and age group. Platelet counts and neutrophil counts were also examined over time to monitor the treatment effect in renal failure patients with a dose reduction.

Hazell L, *et al. BMJ Open* 2026; 16:e100583. doi: 10.1136/bmjopen-2025-100583

The Data Monitoring Committee and chief investigator, with contribution from the unblinded senior statistician and independent statistician reviewed the unblinded efficacy data and made a recommendation on the 29th January 2021 to the Trial Steering Committee to continue to the next stage maintaining existing dosing schedule.

Hazell L, *et al. BMJ Open* 2026; 16:e100583. doi: 10.1136/bmjopen-2025-100583

2. Data analysis plan – data description

2.1 Recruitment and participant flow

Please see section 2.1 in SAP I. This will be undertaken for stage I and stage II

2.2 Baseline characteristics

Please see section 2.2 in SAP I. For ease of presentation median and IQRs will be removed from this analysis. This will be undertaken for stage I and stage II

2.3 Co-enrolment

Please see section 2.3 in SAP I. This will be undertaken for stage I and stage II

2.4 Standard of Care

Please see section 2.4 in SAP I. This will be undertaken for stage I and stage II

2.5 Withdrawals, loss to follow-up and other missing data

Please see section 2.5 in SAP I. This will all be undertaken for stage II, only withdrawal summaries will be prepared for Stage I.

2.6 Adherence to allocated treatment and dose summaries

Please see section 2.6 in SAP I. This will be undertaken for stage I and stage II

All analysis of adherence data at the formal interim analysis will be completed by the independent statistician in Newcastle University to ensure the trial statistician remains blind and only DMC members will see these results.

2.7 Descriptive statistics for outcome measures

The proportion of participants with the primary outcome of WHO COVID-19 ≥ 5 by day 14 will be summarised by treatment arm and repeated for day 28. This will be undertaken for stage I and stage II.

In addition, the proportion of participants experiencing each of the components of the WHO COVID-19 severity scale score (mortality, invasive ventilation plus organ support (renal replacement therapy, ECMO or inotropic support), invasive ventilation, non-invasive ventilation including CPAP and high

flow nasal oxygen) at any point up to day 14 (repeated up to day 28) will be summarised by treatment arm. This will be undertaken for stage I and stage II.

Trajectories of mean (SD) grades over time by treatment arm will also be plotted. This will be undertaken for stage II only.

Time spent at each of the components will be summarised as medians and IQRs by treatment arm. This will be undertaken for stage II only.

All stage 1 analysis of the WHO COVID-19 grades will be completed by the independent statistician in Newcastle University to ensure the trial statistician remains blind and only DMC members will see these results.

Inflammatory markers CRP, LDH, ferritin, D-dimer, and serum creatinine values will be summarised by treatment arm over time and presented as means (SD) and median (IQR). This will be undertaken for stage II

Summaries (means (SD) and median (IQR)) of length of stay will be tabulated amongst those that do not withdraw or die. This will be undertaken for stage II

2.8 Serious adverse events

Incidence of venous thromboembolism and serious adverse events will be collected in this study to assess safety.

The proportion of participants experiencing a venous thromboembolism will be summarised by treatment arm and summaries of the time since randomisation will be presented. The number of readmissions will also be extracted from free text in SAE reports and will be summarised by treatment arm.

Serious adverse events coded as per the CTCAE will be summarised at the preferred term level and body system class. Events will be tabulated by treatment arm including information on the number with at least one event and the number of events to account for recurrent events.

Raw data for each SAE will be listed with information on event type, timing duration and outcome.

All above analysis will be undertaken for stage I and stage II

For stage II analysis we will also undertake the following analysis:

In addition, incident rate ratios for each event between treatment arms (RUX vs SOC or FOS vs SOC) with corresponding 95% CI will be calculated to account for differential follow-up and recurrent events with 95% CIs using a suitable model e.g. a negative binomial model or ZIP as appropriate. These results will be presented in both a table and graphically using a dot plot.

For events where time-to-event analysis is deemed appropriate we will present Kaplan-Meier plots with extended at risk tables for events that occur once within participants and mean cumulative function plots where events can recur within participants.[1, 2]

Blood measurements will be analysed as continuous outcomes calculating a mean difference and 95% CIs over the study follow-up period using a linear mixed effect model and the between arm effect will be plotted over time. Participants will be included as a random intercept with fixed effects for treatment arm, time and baseline outcome values. Stratification variables age and site, plus receipt of known effective treatments dexamethasone and/or tociluzimab, prior vaccination against COVID-19 and receipt of paxlovid and/or molnupiravir at the time of or prior to randomisation into MATIS will also be included as fixed effects. These outcomes will also be categorised as abnormal/normal and analysed using the distributional approach to retain power at day 14 and 28.[3]

Hazell L, et al. BMJ Open 2026; 16:e100583. doi: 10.1136/bmjopen-2025-100583

3. Data analysis plan - Inferential analysis

3.1 Primary outcome of the main study - Modified WHO grading

3.1.1 Definition of the primary outcome

The proportion of participants progressing from mild to severe COVID-19 pneumonia within 14 days. Where severe COVID-19 pneumonia is defined by a modified WHO COVID-19 Ordinal Score ≥ 5 (on the 9 point scale), comprising the following indicators of disease severity: Death (9) *OR* Requirement for invasive ventilation plus organ support (8) Requirement for invasive ventilation (7) *OR* Requirement for non-invasive ventilation including CPAP or high flow oxygen (6) *OR* O_2 saturation < 90% on ≥60% inspired oxygen (5). These are captured in the database as follows:

Modified WHO COVID-19 Ordinal Score	Database
(9) Death	
(8) Requirement for invasive ventilation plus organ support	Has the patient required renal replacement therapy? OR Has the patient required ECMO? OR Has the patient required inotropic support?<br>AND<br>Has the patient required intubation and mechanical ventilation?
(7) Requirement for invasive ventilation	Has the patient required intubation and mechanical ventilation?
(6) Requirement for non-invasive ventilation including CPAP or high flow oxygen	Has the patient required NIV, CPAP or high flow nasal oxygen?
(5) O_2 saturation < 90% on ≥60% inspired oxygen	Has the patient's oxygen saturation been < 90% on 60% inspired oxygen?

3.1.2 Statistical model at stage 1

The stage one primary analysis will consist of fitting two separate binomial regression models for the RUX vs SOC and FOS vs SOC comparisons in order that we can undertake an adjusted analysis using a propensity score approach. Specifically a generalized linear model with WHO COVID-19 status (<5 or ≥5) as the outcome and treatment group as a covariate. We will adjust the treatment effect for the

following prespecified covariates using a propensity score approach. The propensity score model will include:

- baseline WHO COVID-19 score (3 or 4)
- age (<65 or ≥65)
- receipt of dexamethasone (prior randomisation or up to 1 day post randomisation)
- receipt of tociluzimab (prior randomisation or up to 1 day post randomisation)

In the event that all participants are on one of the effective treatments that covariate will be removed from the propensity score. We will adjust using the propensity score from a logistic regression model including the above covariates. We will also fit an unadjusted generalized linear model with WHO COVID-19 status (<5 or ≥5) as the outcome and treatment group as a covariate.

The models will be fitted with a binomial distribution and logit link function and the treatment effect will be reported as an odds ratio with corresponding 95% confidence interval and p-values. P-values will be one-sided and judged at the 0.25 level. Given that the number of sites is likely to be small we will not include site as a covariate in this stage 1 analysis.

Every effort will be made to obtain follow-up data for all participants including those that stop treatment. The number and proportion of participants missing WHO COVID-19 status by treatment group and visit will be tabulated (see section 2.5 above). For participants who are discharged early the assumption will be made that they did not progress to grade 5 WHO scale. If there is a >5% rate of missing outcomes after this, we will impute missing WHO COVID-19 data in the primary analysis model using multiple imputation under the assumption of missing at random (MAR). That is the probability of being missing is not dependent on the values of unobserved data, conditional on the observed values of the variables included in the analysis model. Imputation under MAR using chained equations following the guidance of White et al. will be undertaken.[4] Variables in the imputation model will include the variables in the primary analysis model described above plus baseline WHO COVID-19 score (3 or 4), age (<65 or ≥65), receipt of dexamethasone and/or tociluzimab at the time of or prior to randomisation into MATIS. Additional auxiliary variables that are suspected to be associated with the outcome and are predictors of missingness will also be included. These are receipt of any effective treatments post randomisation as a result of co-enrolment when occurring post-randomisation, and receipt of known effective treatments dexamethasone and tociluzimab post-randomisation (>1 day post randomisation) and WHO COVID-19 scores at day 1 and day 7. Using White et al.'s rule of thumb we will impute m datasets based on the percentage of participants with incomplete outcomes, where m will be rounded to the nearest 10, for example if 8% of participants have incomplete primary outcome then m=10 will be used.

Before undertaking the multiple imputation as described above we will impute any missing baseline data deterministically only using other baseline data, this includes the baseline WHO COVID-19 status, age, receipt of dexamethasone and/or tociluzimab at the time of or prior to randomisation into MATIS. To do this we will impute missing values with the mean values of observed data. In line with White et al., outcomes will be imputed separately for each treatment group.

Imputed datasets will then be analysed using the primary analysis model described above.

A sensitivity analysis on the primary analysis will be undertaken if greater than 5% of outcome data are missing to examine the missing at random assumption. We will examine extreme examples assuming 1) that all participants with missing outcome had poor outcome e.g. all missing >=5, and 2) that all participants with missing outcome had good outcome e.g. all missing <5.

i. Compliance

Adherence to assigned treatment (RUX and FOS) will be calculated for each participant as the proportion of dose received per day of the prescribed dose per day and presented as the percentage received. This will be summarised by treatment group as described in section 2.6 above.

To explore the impact of compliance with the planned treatment protocol, we will undertake a per-protocol analysis to examine the treatment effect in those that take most of the treatment. We will define 'per-protocol' population as individuals who receives ≥ 90% of prescribed dose and refit the primary analysis model on this population.

ii. Time of enrolment

An exploratory analysis of the primary outcome will be undertaken in order to assess the impact of time of enrolment into the MATIS study. A categorical variable will be created by splitting the recruitment period into quartiles such that participants are grouped into approximately four equal sized groups based on time of enrolment into the MATIS study. A crude odds ratio will be calculated for each quartile.

All stage 1 analysis of the WHO COVID-19 grades will be completed by the independent statistician in Newcastle University to ensure the trial statistician remains blind and only DMC members will see these results.

3.1.3 Statistical model at stage 2

The stage two primary analysis will consist of fitting one binomial regression model regardless of whether one or two experimental treatments progress from stage one. Specifically a generalized linear model with WHO COVID-19 status (<5 or ≥5) as the outcome and treatment group, baseline WHO COVID-19 score (3 or 4), age (<65 or ≥65), receipt of known effective treatments dexamethasone and/or tociluzimab, prior vaccination against COVID-19 (y/n) and receipt of paxlovid and/or molnupiravir at the time of or prior to randomisation into MATIS as covariates will be fitted. Effective treatments will be included as separate covariates in the primary analysis model. Given the anticipated small number of sites in this study, site will also be included in the model as a fixed effect.[5] In the event that all participants are on one of the effective treatments that covariate will be removed from the model.

If including site as a fixed effect results in unstable model estimates e.g. if there are a number of sites with very few randomisations we will exclude site from the model and we will only adjust for baseline WHO COVID score, age group, receipt of known effective treatments dexamethasone and/or tociluzimab, prior vaccination against COVID-19, and receipt of paxlovid and/or molnupiravir.[6] We will also consider not adjusting for paxlovid and/or molnupiravir if the number of participants receiving these are low. If after removing sites and treatments the model is not stable due to event rate:covariate ratio we will adjust for all covariates using a propensity score approach.

The model will be fitted with a binomial distribution and logit link function and the treatment effect will be reported as an odds ratio with corresponding 95% confidence interval and p-values. P-values will be one-sided and judged at the 0.027 level. Therefore, a two-sided p-value < 0.054 will be used to indicate a significant treatment effect.

The stage two model to be fitted to estimate the treatment effects will be:

$$logit(\pi_{ij}) = \beta_0 + \beta_1 RUX_{ij} + \beta_2 FOS_{ij} + \beta_3 WHO_{ij}^0 + \beta_4 AGE_{ij}^0 + \beta_5 DEX_{ij}^0 + \beta_6 TOC_{ij}^0 + \beta_7 VAC_{ij}^0 + \beta_8 PAX_{ij}^0 + \beta_9 MOL_{ij}^0 + \beta_{10}\, SITE_1 + \beta_{11}\, SITE_2 + \cdots + \beta_{12}\, SITE_{j-1}$$

RUX_{ij}: dummy variable for treatment ($RUX_{ij} = 0\ or\ 1$) for participant i in j[th] site

FOS_{ij}: dummy variable for treatment ($FOS_{ij} = 0\ or\ 1$) for participant i in j[th] site

WHO_{ij}^0: dummy variable for baseline WHO COVID-19 value for participant i in j[th] site ($WHO_{ij} = 0\ or\ 1\ where\ 0\ indicates\ score\ of\ 3\ and\ 1\ indicates\ score\ of\ 4$)

AGE_{ij}^0: dummy variable for age-group ($AGE_{ij} = 0\ or\ 1\ where\ 0\ indicates < 65\ years\ and\ 1\ indicates\ \geq 65$) for participant i

DEX_{ij}^0: dummy variable for receipt of dexamethasone at the time of or prior to randomisation (includes day of randomisation or one day post randomisation) ($DEX_{ij} = 0\ or\ 1$) for participant i

TOC_{ij}^0: dummy variable for receipt of tociluzimab at the time of or prior to randomisation (includes day of randomisation or one day post randomisation) ($TOC_{ij} = 0\ or\ 1$) for participant i

VAC_{ij}^0: dummy variable for prior vaccination against COVID-19 at the time of or prior to randomisation (includes day of randomisation or one day post randomisation) ($VAC_{ij} = 0\ or\ 1$) for participant i

PAX_{ij}^0: dummy variable for receipt of paxlovid at the time of or prior to randomisation (includes day of randomisation or one day post randomisation) ($PAX_{ij} = 0\ or\ 1$) for participant i

MOL_{ij}^0: dummy variable for receipt of molnupiravir at the time of or prior to randomisation (includes day of randomisation or one day post randomisation) ($MOL_{ij} = 0\ or\ 1$) for participant i$Site_j$: dummy variable for each of the jth sites

π_{ij}: probability of WHO COVID-19 ≥5 for the ith participant in j[th] site

To account for missing outcomes in the primary analysis model we will impute missing WHO COVID-19 status using multiple imputation under the assumption of missing at random (MAR) as described in the primary statistical model at stage 1 section 3.1.2 plus the imputation model will contain the following additional variables: prior vaccination against COVID-19, and receipt of paxlovid and/or molnupiravir at the time of or prior to randomisation into MATIS, plus post-randomisation receipt of paxlovid and/or molnupiravir. Multiple imputation will be used for the final analysis regardless of levels of missingness at this stage.

As well as reporting odds ratios and 95% CIs, the model will be used to estimate the difference in proportion of participants with modified WHO COVID-19 Ordinal Score ≥ 5.

3.1.4 Model assessment of stage 2 analysis

The primary analysis model will be assessed for model fit and outlying or influential observations as per methods described below.

To determine the fit of the model by using summary measures of goodness of fit and by assessing the estimation ability of the model:

i. Perform the goodness-of-fit test using the Hosmer-Lemeshow test to look at the discrepancy between the observed and estimated frequency of outcomes (Stata command *estat gof*).
ii. Examination of the ROC curve (Stata command *lroc*) to assess the correct identification of outcome based on varying probability thresholds.
iii. Plot of the Pearson's standardised residuals (*predict.., rstand*) against predicted values (*predict..*). Plots showing trends suggest poor model fit or misspecification of model.

If model assumptions are deemed to be invalid or the model appears to be miss-specified, we will undertake a sensitivity analysis with an alternative model.

To determine if there are any observations that do not fit the model or have a large influence on the model:

i. Check for outliers/predicted values that have poor fit by plotting deviance residuals (*predict .., deviance*) against predicted values (*predict ..*); standardised Pearson's residuals (*predict…, rstand*) against predicted; and leverage (predict .., hat) against predicted.
ii. Investigate whether the extreme outlying observations have been recorded correctly and if so, compare model coefficient estimates with and without the identified influential observations
iii. Use Cook's distance (stata command *predict.., cook*) to identify influential observations

Hazell L, *et al. BMJ Open* 2026; 16:e100583. doi: 10.1136/bmjopen-2025-100583

3.1.5 Stage 2 exploratory analysis for primary outcome

i. Co-enrolment at time of randomisation

An exploratory analysis of the primary outcome will be undertaken in order to assess the impact of co-enrolment to an 'active'* arm of another study at the time of or prior to randomisation into MATIS (includes day of randomisation or one day post randomisation). Individual treatments will be included as separate covariates in the primary analysis model described above. In the event of low event rates the sensitivity analysis will be repeated removing one covariate at a time.

*We will only consider those co-enrolled to an 'active' arm of another study. Co-enrolment to the standard of care arm in other studies will not be considered an active treatment.

ii. Co-enrolment post randomisation and receipt of rescue medication

We will explore what the direct effect of the treatment is on the outcome, and whether there is an indirect effect of receipt of active treatment as a result of co-enrolment when occurring post-randomisation, and receipt of known effective treatments dexamethasone, tociluzimab, paxlovid and molnupiravir post-randomisation (>1 day post randomisation). Where post-randomisation co-enrolment or receipt of known effective treatments must occur prior to the occurrence of the outcome. We will do this via a mediation analysis using a structural equation modelling approach using the Stata *paramed* command.[7, 8]

The analysis will allow us to decompose the total observed treatment effect into mediated (indirect, described by the natural indirect effect in the Stata output) and non-mediated (direct, described by the natural direct (and controlled direct effect) effect estimate in Stata output) components. Each post-randomisation active intervention and known effective treatments will be treated as an individual mediator, thus we will estimate the mediated effect separately for each. Analysis models will adjust for baseline WHO COVID-19 score (3 or 4), age (<65 or ≥65), receipt of known effective treatments dexamethasone and/or tociluzimab, prior vaccination against COVID-19, and receipt of paxlovid and/or molnupiravir at the time of or prior to randomisation into MATIS and site as fixed as per the primary analysis model described in section 3.1.3. The model will be modified in a similar manner as the primary outcome modelling in the event of low event rates.

iii. Compliance

We will undertake an exploratory analysis to examine the treatment effect (reported as a risk ratio (RR)) adjusted for compliance. This will be achieved through a complier adjusted case (CACE) analysis using a two-stage residual inclusion estimator (2SRI) approach with randomisation group as the instrumental variable. The primary analysis model described in 3.1.3 will be refitted with the log link function (instead of logit function) to give the treatment effect as a RR with corresponding 95% confidence interval and p-values. The CACE will then be estimated for this model and we will compare the CACE estimates of the RR to the RR obtained from the refitted primary analysis model. Randomisation meets the criteria for an adequate instrument variable since (i) randomisation predicts the treatment receipt, (ii) randomisation is unconfounded with the outcome and we assume (iii) no direct effect of randomisation on the outcome (other than via treatment receipt) – *the exclusion restriction.* After the second stage of estimation, the standard errors of the estimates will be corrected using the method of Terza.[9, 10]

This approach can only include adherence information in the intervention groups as the equivalent usage in the control participants is not recorded (as the control intervention is SOC and can differ between participants and is likely to evolve with time). SOC will be assumed to be 100% compliant with prescribed treatment.

We will define a complier as an individual who receives ≥ 90% of prescribed dose. In addition to the analysis using a 90% threshold to define compliance we will undertake alternative analysis defining a ‘complier’ as an individual who receives ≥ 60%, ≥ 70% and ≥ 80% of prescribed dose to explore the impact of alternative definitions of compliance.

Analysis will adjust for baseline WHO COVID-19 score (3 or 4), age (<65 or ≥65)), receipt of known effective treatments dexamethasone and/or tociluzimab, prior vaccination against COVID-19, and receipt of paxlovid and/or molnupiravir at the time of or prior to randomisation into MATIS and site as fixed as per the primary analysis model described in section 3.1.3. The model will be modified in a similar manner as the primary outcome modelling in the event of low event rates.

iv. Time of enrolment

An exploratory analysis of the primary outcome will be undertaken in order to assess the impact of time of enrolment. A categorical variable will be created by splitting the recruitment period into quartiles such that participants are grouped into approximately four equal sized groups based on time of enrolment into the MATIS study. This variable will then be included as a covariate in the primary analysis model described above as a treatment group-by-time interaction term to assess the impact of time of recruitment on the treatment effect estimate.

3.1.6 Stage 2 subgroup analysis for primary outcome

Subgroup analysis will be performed for the primary outcome for the final analysis only to explore the uniformity of the treatment effect. The following subgroups will be examined by adding a treatment-by-subgroup interaction term to the same analysis model as for the primary outcome:

- Sex: male vs female;
- Age: <65 years vs ≥65 years
- Obesity: <25, 25-29.9, ≥30
- Chronic heart disease: yes vs no;
- Lung disease: yes vs no;
- Kidney disease: yes vs no;
- Liver disease: yes vs no;
- Diabetes: yes vs no;
- Immunocompromised: yes vs no;
- Smoking status: current vs non-smoker;
- CRP > 200mg/L: yes vs no and/or D-dimer >2500mg/mL: yes vs no;
- Time since onset of symptoms:0-5, 6-10, >10 days prior to randomisation

P-values for each interaction term will be presented. No adjustment for multiple tests will be made and the results will be viewed as hypothesis generating only. The consistency of estimates will be depicted visually by means of a forest plot.

3.2 Secondary outcomes of the main study

Analysis of the secondary outcomes listed below will be undertaken for the final analysis only. Each of the outcomes will be examined at 14 and 28 days and comparisons will be between RUX or FOS versus SOC:

- Change in severity on the modified WHO COVID-19 Ordinal Scale (3 or 4 to 5,6,7,8,9)
- Death (yes/no)
- Invasive ventilation (yes/no)
- ECMO (yes/no)
- Non-invasive ventilation including CPAP and high flow nasal oxygen (yes/no)
- Renal replacement therapy (yes/no)
- Venous thromboembolism (yes/no)
- Inflammatory markers CRP, LDH, ferritin, D-dimer (units)

Hazell L, *et al. BMJ Open* 2026; 16:e100583. doi: 10.1136/bmjopen-2025-100583

- Length of stay (days)
- Serum creatinine

3.2.1 Severe COVID-19 within 28 days

The proportion of participants progressing from mild to severe COVID-19 pneumonia within 28 days will be analysed using the main analysis model as described in section 3.1.3, replacing the 14 day outcome with the 28 day outcome.

3.2.2 Modified WHO COVID-19 score

A mixed effects ordered logistic regression will be used to estimate the odds ratio of disease severity as measured by the modified WHO COVID-19 (ordinal scale) over time by treatment arm. Specifically a generalized linear model with modified WHO COVID-19 status (scores 3/4 through to 9) as the outcome (ordered categorical variable) with S ordinal categories s (s=3/4, 5, 6, 7, 8, 9) and treatment group, time, time-by-treatment arm interaction, baseline WHO COVID-19 score (3 or 4), age (<65 or ≥65), receipt of known effective treatments dexamethasone and/or tociluzimab, prior vaccination against COVID-19, and receipt of paxlovid and/or molnupiravir at the time of or prior to randomisation into MATIS as covariates will be fitted. Participants will be included as a random intercept (ζ_{ij}). Given the anticipated small number of sites in this study, site will be included in the model as a fixed effect.[5] The model will be fitted using the *ologit* link function (or the *meologit* command) in Stata and the treatment effect will be reported as an odds ratio with corresponding 95% confidence intervals.

The model to be fitted will be:

$$
\begin{aligned}
logit\big(pr(\pi_{ij} > s)\big) \\
&= \beta_1 RUX_{ij} + \beta_2 FOS_{ij} + \beta_3 WHO^0_{ij} + \beta_4 AGE^0_{ij} + \beta_5 DEX^0_{ij} + \beta_6 TOC^0_{ij} \\
&+ \beta_7 VAC^0_{ij} + \beta_8 PAX^0_{ij} + \beta_9 MOL^0_{ij} + \beta_{10}\, SITE_1 + \beta_{11}\, SITE_2 + \cdots \\
&+ \beta_{12}\, SITE_{j-1} + \beta_{13} time_7 + \beta_{14} time_{14} + \beta_{15} RUX_{ij} * time_7 + \beta_{16} RUX_{ij} \\
&* time_{14} + \beta_{17} FOS_{ij} * time_7 + \beta_{18} FOS_{ij} * time_{14} + \zeta_{ij} - \kappa_s
\end{aligned}
$$

RUX_{ij}: dummy variable for treatment ($RUX_{ij} = 0\ or\ 1$) for participant i in j[th] site

FOS_{ij}: dummy variable for treatment ($FOS_{ij} = 0\ or\ 1$) for participant i in j[th] site

WHO^0_{ij}: dummy variable for baseline WHO COVID-19 value for participant i in j[th] site ($WHO_{ij} = 0\ or\ 1\ where\ 0\ indicates\ score\ of\ 3\ and\ 1\ indicates\ score\ of\ 4$)

AGE_{ij}^{0}: dummy variable for age-group ($AGE_{ij} = 0\ or\ 1\ where\ 0\ indicates <$
$65\ years\ and\ 1\ indicates\ \geq 65$) for participant i

DEX_{ij}^{0}: dummy variable for receipt of dexamethasone at the time of or prior to randomisation (includes day of randomisation or one day post randomisation) ($DEX_{ij} = 0\ or\ 1$) for participant i

TOC_{ij}^{0}: dummy variable for receipt of toczulimab at the time of or prior to randomisation (includes day of randomisation or one day post randomisation) ($TOC_{ij} = 0\ or\ 1$) for participant i

VAC_{ij}^{0}: dummy variable for prior vaccination against COVID-19 at the time of or prior to randomisation (includes day of randomisation or one day post randomisation) ($VAC_{ij} = 0\ or\ 1$) for participant i

PAX_{ij}^{0}: dummy variable for receipt of paxlovid at the time of or prior to randomisation (includes day of randomisation or one day post randomisation) ($PAX_{ij} = 0\ or\ 1$) for participant i

MOL_{ij}^{0}: dummy variable for receipt of molnupiravir at the time of or prior to randomisation (includes day of randomisation or one day post randomisation) ($MOL_{ij} = 0\ or\ 1$) for participant i

$Site_j$: dummy variable for each of the jth sites

$time_x$: dummy variable for time (= 0 or 1) at time point x days. Day 1 is represented by $time_7 = 0$ and $time_{14} = 0$

$\zeta_{ij} \sim N(0, \sigma^2)$: participant specific random intercept

κ_s: indicates the $\kappa_1, \kappa_{2,\ldots.}\kappa_{S-1}$ cut-points for each of the possible S outcomes of the WHO COVID-19 ordinal scale

$\left(pr(\pi_{ij} > s)\right)$: cumulative probability of the response being in a category higher than s

π_{ij}: is the odds ratio of more severe versus less severe illness (regardless of where the ordinal scale is cut)

The model will be modified in a similar manner as the primary outcome modelling in the event of low event rates.

This analysis will be repeated on the original WHO COVID-19 (ordinal scale).

3.2.3 Components of the modified WHO COVID-19 score

The following outcomes will be examined at day 14 and day 28 by replacing the primary outcome in the main analysis model as described in section 3.1.3:

- Death (yes/no)
- Requirement for invasive ventilation plus organ support (yes/no)
- Invasive ventilation (yes/no)

Hazell L, et al. BMJ Open 2026; 16:e100583. doi: 10.1136/bmjopen-2025-100583

- Non-invasive ventilation including CPAP and high flow nasal oxygen (yes/no)
- Renal replacement therapy (yes/no)
- Venous thromboembolism (yes/no)

Each model will be fitted on complete cases and no imputation of missing values or sensitivity analysis will be undertaken.

3.2.4 Length of stay

The time to discharge will be summarised by treatment arm using a Kaplan-Meier plot with extended at risk tables, detailing the numbers at risk, censored and discharged at each discrete time point, censoring at the day 28 visit. Withdrawals and deaths will be censored at the date of occurrence. In addition, summaries (means (SD) and median (IQR)) of length of stay will be tabulated amongst those that do not withdraw or die.

In addition, a Cox proportional hazards model will be used to estimate the hazard ratio of time to discharge between intervention arms with age (<65 or ≥65), site and receipt of known effective treatments dexamethasone and/or tociluzimab and vaccination status at the time of or prior to randomisation into MATIS included as covariates.

3.2.5 Continuous laboratory values

For inflammatory markers of CRP, LDH, ferritin, D-dimer and serum creatinine levels a linear mixed effect model will be used to estimate the mean difference in values between intervention arms (RUX and FOS) and SOC at each follow-up visit (day 1, day 7 and day 14).[11] Participants will be included as a random intercept with fixed effects for time, time-by-treatment arm interaction and baseline values. Stratification variables age and site, plus receipt of known effective treatments dexamethasone and/or tociluzimab, prior vaccination against COVID-19, and receipt of paxlovid and/or molnupiravir at the time of or prior to randomisation into MATIS will also be included as fixed effects. Baseline adjusted treatment group differences with 95% confidence intervals will be presented for each time-point.

For example for CRP the model to be fitted to estimate the treatment effect at each visit up to day 14 will be:

$$\begin{aligned} Y_{ijt} = \ & \beta_0 + \beta_1 RUX_{ij} + \beta_2 FOS_{ij} + \beta_3 CRP_{ij}^0 + \beta_4 AGE_{ij}^0 + \beta_5 DEX_{ij}^0 \\ & + \beta_6 TOC_{ij}^0 + \beta_7 VAC_{ij}^0 + \beta_8 PAX_{ij}^0 + \beta_9 MOL_{ij}^0 \ \beta_{10} time_7 \\ & + \beta_{11} time_{14} + \beta_{12} RUX_{ij} * time_7 + \beta_{13} RUX_{ij} * time_{14} \\ & + \beta_{14} FOS_{ij} * time_7 + \beta_{15} FOS_{ij} * time_{14} + \beta_{16} SITE_1 \\ & + \beta_{17} SITE_2 + \cdots + \beta_{18} SITE_{j-1} + b_{1,i} + e_{it} \end{aligned}$$

for

t = 1 to 3 time points (day 1, 7 and 14), i = 1 to 30 participants

RUX_{ij}: dummy variable for treatment ($RUX_{ij} = 0 \ or \ 1$) for participant i

FOX_{ij}: dummy variable for treatment ($FOS_{ij} = 0 \ or \ 1$) for participant i

CRP_{ij}^0: baseline CRP value for participant i

DEX_{ij}^0: dummy variable for receipt of dexamethasone at the time of or prior to randomisation (includes day of randomisation or one day post randomisation) ($DEX_{ij} = 0 \ or \ 1$) for participant i

TOC_{ij}^0: dummy variable for receipt of tociluzimab at the time of or prior to randomisation (includes day of randomisation or one day post randomisation) ($TOC_{ij} = 0 \ or \ 1$) for participant i

VAC_{ij}^0: dummy variable for prior vaccination against COVID-19 at the time of or prior to randomisation (includes day of randomisation or one day post randomisation) ($VAC_{ij} = 0 \ or \ 1$) for participant i

PAX_{ij}^0: dummy variable for receipt of paxlovid at the time of or prior to randomisation (includes day of randomisation or one day post randomisation) ($PAX_{ij} = 0 \ or \ 1$) for participant i

MOL_{ij}^0: dummy variable for receipt of molnupiravir at the time of or prior to randomisation (includes day of randomisation or one day post randomisation) ($MOL_{ij} = 0 \ or \ 1$) for participant i

$time_x$: dummy variable for time (= 0 or 1) at time point x days. Day 1 is represented by $time_7 = 0$ and $time_{14} = 0$

$Site_j$: dummy variable for each of the jth sites

$b_{1,i} \sim N(0, \sigma_{b1}^2)$, $e_{it} \sim N(0, \sigma_e^2)$

Where $b_{1,i}$ is the random intercept at the participant level. Each of $b_{1,i}$ and e_{it} are assumed to follow normal distributions. An unstructured covariance matrix will be used and the model will be fitted using restricted maximum likelihood (REML). This model makes assumptions about random effects distributions, correlation structures and residuals, which will all be investigated. If the model fails to converge model estimates will not be presented.

The above will be repeated for each inflammatory marker up to day 14 and additionally up to day 28.

References

1. Morris TP, Jarvis CI, Cragg W, et al. Proposals on Kaplan–Meier plots in medical research and a survey of stakeholder views: KMunicate. *BMJ Open* 2019; 9: e030215. DOI: 10.1136/bmjopen-2019-030215.
2. Siddiqui O. Statistical methods to analyze adverse events data of randomized clinical trials. *Journal of Biopharmaceutical Statistics* 2009; 19: 889-899. DOI: http://dx.doi.org/10.1080/10543400903105463.
3. Chis Ster A, Phillips R, Sauzet O, et al. Improving analysis practice of continuous adverse event outcomes in randomised controlled trials - a distributional approach. *Trials* 2021; 22: 419. DOI: 10.1186/s13063-021-05343-0.
4. White IR, Royston P and Wood AM. Multiple imputation using chained equations: Issues and guidance for practice. *Statistics in Medicine* 2011; 30: 377-399. DOI: https://doi.org/10.1002/sim.4067.
5. Kahan BC. Accounting for centre-effects in multicentre trials with a binary outcome – when, why, and how? *BMC Medical Research Methodology* 2014; 14: 20. DOI: 10.1186/1471-2288-14-20.
6. Kahan BC and Morris TP. Improper analysis of trials randomised using stratified blocks or minimisation. *Stat Med* 2012; 31: 328-340. 2011/12/06. DOI: 10.1002/sim.4431.
7. Valeri L and Vanderweele TJ. Mediation analysis allowing for exposure-mediator interactions and causal interpretation: theoretical assumptions and implementation with SAS and SPSS macros. *Psychological methods* 2013; 18: 137-150. 2013/02/04. DOI: 10.1037/a0031034.
8. Liu H, Emsley R, Dunn G, et al. paramed: A command to perform causal mediation analysis using parametric models. 2014.
9. Cook JA, MacLennan GS, Palmer T, et al. Instrumental variable methods for a binary outcome were used to informatively address noncompliance in a randomized trial in surgery. *J Clin Epidemiol* 2018; 96: 126-132. 2017/11/22. DOI: 10.1016/j.jclinepi.2017.11.011.
10. Terza JV. Simpler Standard Errors for Two-stage Optimization Estimators. *The Stata Journal* 2016; 16: 368-385. DOI: 10.1177/1536867x1601600206.
11. Twisk J, Bosman L, Hoekstra T, et al. Different ways to estimate treatment effects in randomised controlled trials. *Contemporary Clinical Trials Communications* 2018; 10: 80-85. DOI: https://doi.org/10.1016/j.conctc.2018.03.008.
