# Supplementary material for: Multiarm multistage randomised controlled trial of inflammatory signal inhibitors (MATIS) for patients hospitalised with COVID-19 pneumonia during the UK pandemic
Source: BMJ Open. 2026 Feb 5;16(2):e100583. doi: 10.1136/bmjopen-2025-100583 (PMC12887464; doi:10.1136/bmjopen-2025-100583)
Supplement: Supplementary data [file bmjopen-16-2-s002.pdf]

Supplementary Appendix 2

Characteristics of participants in MATIS stratified by predominant COVID-19 variant at time of randomisation

Table S1: Baseline characteristics of all participants by predominant variant at time of randomisation

Patient characteristic, n(%) unless otherwise specified	Alpha (up to May 2021) (N=112)	Delta (May to Dec 2021) (N=61)	Omicron (post Dec 2021) (N=12)
Age in years			
Mean (SD)	60.8 (14.7)	55.9 (16.6)	65.8 (17.1)
Sex			
Male	79 (70.5)	43 (70.5)	6 (50.0)
Female	33 (29.5)	18 (29.5)	6 (50.0)
Ethnicity			
White	41 (36.6)	30 (49.2)	8 (66.7)
Mixed or multiple ethnic groups	1 (0.9)	1 (1.6)	0 (0.0)
Asian or Asian British	14 (12.5)	8 (13.1)	4 (33.3)
Black, Black British, Caribbean or African	12 (10.7)	7 (11.5)	0 (0.0)
Other ethnic group	44 (39.3)	15 (24.6)	0 (0.0)
BMI in kg/m2			
N (N missing)	106 (6)	55 (6)	11 (1)
Mean (SD)	30.2 (6.4)	31.0 (7.8)	26.5 (5.1)
Severity of Covid			
Grade 3	17 (15.2)	13 (21.3)	3 (25.0)
Grade 4	95 (84.8)	48 (78.7)	9 (75.0)
Chronic lung disease			
N (N missing)	111 (1)	60 (1)	12 (0)
Yes	15 (13.5)	13 (21.7)	2 (16.7)
Time from onset of symptoms			
N (N missing)	103 (9)	51 (10)	11 (1)
Mean (SD)	9 (4)	10 (4)	12 (5)
Diabetes			
N (N missing)	111 (1)	60 (1)	12 (0)
Yes	37 (33.3)	13 (21.7)	4 (33.3)
Hypertension			
N (N missing)	111 (1)	60 (1)	12 (0)
Yes	53 (47.7)	21 (35.0)	7 (58.3)
Ischaemic heart disease			
N (N missing)	111 (1)	60 (1)	12 (0)
Yes	17 (15.3)	9 (15.0)	5 (41.7)
Heart failure			
N (N missing)	111 (1)	60 (1)	12 (0)
Yes	3 (2.7)	2 (3.3)	2 (16.7)
Immunocompromised			
N (N missing)	111 (1)	60 (1)	12 (0)
Yes	4 (3.6)	2 (3.3)	2 (16.7)
End-stage renal failure			
N (N missing)	111 (1)	60 (1)	12 (0)
Yes	10 (9.0)	2 (3.3)	1 (8.3)
Liver cirrhosis			
N (N missing)	111 (1)	60 (1)	12 (0)
Yes	0 (0.0)	0 (0.0)	0 (0.0)
Current smoker			

N (N missing)	110 (2)	60 (1)	12 (0)
Yes	5 (4.5)	0 (0.0)	0 (0.0)
Prior Covid Vaccination			
N (N missing)	112 (0)	61 (0)	12 (0)
Yes	9 (8.0)	20 (32.8)	6 (50.0)
Serum creatinine (µmol/L)			
N (N missing)	111 (1)	59 (2)	12 (0)
Mean (SD)	139 (209)	95 (85)	115 (112)
C-reactive protein (mg/L)			
N (N missing)	111 (1)	59 (2)	12 (0)
Mean (SD)	115 (70)	100 (75)	76 (46)
Lactate dehydrogenase (IU/L)			
N (N missing)	73 (39)	28 (33)	8 (4)
Mean (SD)	478 (255)	388 (153)	390 (173)
Ferritin (µg/L)			
N (N missing)	98 (14)	43 (18)	10 (2)
Mean (SD)	1659 (2343)	1235 (1145)	472 (285)
D-dimer (ng/ml)			
N (N missing)	103 (9)	49 (12)	9 (3)
Mean (SD)	1851 (3302)	996 (764)	1391 (1082)
