# Supplementary material for: Multiarm multistage randomised controlled trial of inflammatory signal inhibitors (MATIS) for patients hospitalised with COVID-19 pneumonia during the UK pandemic
Source: BMJ Open. 2026 Feb 5;16(2):e100583. doi: 10.1136/bmjopen-2025-100583 (PMC12887464; doi:10.1136/bmjopen-2025-100583)
Supplement: Supplementary data [file bmjopen-16-2-s003.pdf]

Supplementary Appendix 3

Standard of care treatments received pre- and post-randomisation

Table S2: Selected immunological therapies at or prior to randomisation by treatment arm

Drug	FOS (N=58)	RUX (N=64)	SOC (N=63)
Dexamethasone			
Received ≥1 dose, n (%)	56 (96.6)	57 (89.1)	55 (87.3)
Median (IQR) time since initiation (days); n missing	1 (0, 1); 0	1 (1, 2); 0	1 (0, 1); 0
Median (IQR) duration (days), n missing	7 (5, 9); 0	9 (5, 13); 0	8 (5, 10); 1
Prednisolone			
Received ≥1 dose, n (%)	0 (0.0)	1 (1.6)	1 (1.6)
Median (IQR) time since initiation (days); n missing	N/A	1 (1, 1); 0	1 (1, 1); 0
Median (IQR) duration (days), n missing	N/A	6 (6, 6); 0	1 (1, 1); 0
Methylprednisolone			
Received ≥1 dose, n (%)	0 (0.0)	0 (0.0)	0 (0.0)
Median (IQR) time since initiation (days); n missing	N/A	N/A	N/A
Median (IQR) duration (days), n missing	N/A	N/A	N/A
Hydrocortisone			
Received ≥1 dose, n (%)	0 (0.0)	1 (1.6)	0 (0.0)
Median (IQR) time since initiation (days); n missing	N/A	1 (1, 1); 0	N/A
Median (IQR) duration (days), n missing	N/A	1 (1, 1); 0	N/A
Tocilizumab			
Received ≥1 dose, n (%)	9 (15.5)	8 (12.5)	7 (11.1)
Median (IQR) time since initiation (days); n missing	0 (0, 1); 0	0 (0, 1); 0	0 (0, 1); 0
Median (IQR) duration (days), n missing	1 (1, 1); 0	1 (1, 1); 0	1 (1, 1); 0
Sarilumab			
Received ≥1 dose, n (%)	2 (3.4)	5 (7.8)	2 (3.2)
Median (IQR) time since initiation (days); n missing	1 (0, 1); 0	0 (0, 1); 0	1 (0, 1); 0
Median (IQR) duration (days), n missing	1 (1, 1); 0	1 (1, 1); 0	1 (1, 1); 0
Any IL6 therapy			
Received ≥1 dose, n (%)	11 (19.0)	13 (20.3)	9 (14.3)
Median (IQR) time since initiation (days); n missing	0 (0, 1); 0	0 (0, 1); 0	0 (0, 1); 0
Median (IQR) duration (days), n missing	1 (1, 1); 0	1 (1, 1); 0	1 (1, 1); 0
Baricitinib			
Received ≥1 dose, n (%)	0 (0.0)	0 (0.0)	0 (0.0)
Median (IQR) time since initiation (days); n missing	N/A	N/A	N/A
Median (IQR) duration (days), n missing	N/A	N/A	N/A

N/A: not applicable

Table S3 Standard of care treatments after randomisation by treatment arm

Drug group	FOS (N=58)	RUX (N=64)	SOC (N=63)
Anti-viral agents			
Received ≥1 dose, n (%)	3 (5.2)	3 (4.7)	3 (4.8)
Median (IQR) time since initiation (days); n missing	10 (6, 11); 0	1 (1, 3); 0	1 (1, 1); 0
Median (IQR) duration (days); n missing	1 (1, 4); 0	5 (5, 5); 0	5 (5, 6); 0
Immuno-modulating agents			
Received ≥1 dose, n (%)	14 (24.1)	13 (20.3)	16 (25.4)
Median (IQR) time since initiation (days); n missing	8 (5, 10); 0	9 (2, 14); 0	3 (1, 5); 0
Median (IQR) duration (days); n missing	18 (2, 22); 2	8 (1, 11); 0	3 (1, 24); 0
Antibiotics			
Received ≥1 dose, n (%)	19 (32.8)	16 (25.0)	14 (22.2)
Median (IQR) time since initiation (days); n missing	7 (4, 13); 0	8 (4, 14); 0	5 (1, 12); 0
Median (IQR) duration (days); n missing	6 (3, 8); 1	7 (3, 13); 0	6 (3, 8); 0
Anticoagulation treatment			
Received ≥1 dose, n (%)	6 (10.3)	7 (10.9)	10 (15.9)
Median (IQR) time since initiation (days); n missing	10 (2, 19); 0	5 (3, 8); 0	8 (3, 10); 0
Median (IQR) duration (days); n missing	20 (9, 31); 1	8 (2, 22); 0	18 (5, 28); 3
Antiplatelet therapy			
Received ≥1 dose, n (%)	3 (5.2)	2 (3.1)	2 (3.2)
Median (IQR) time since initiation (days); n missing	1 (1, 2); 0	6 (1, 11); 0	2 (2, 2); 0
Median (IQR) duration (days); n missing	4 (4, 27); 0	16 (16, 16); 1	29 (29, 29); 0
Convalescent serum therapy			
Received ≥1 dose, n (%)	0 (0.0)	2 (3.1)	1 (1.6)
Median (IQR) time since initiation (days); n missing	N/A	1 (1, 2); 0	1 (1, 1); 0
Median (IQR) duration (days); n missing	N/A	2 (1, 2); 0	2 (2, 2); 0
Ronapreve®			
Received ≥1 dose, n (%)	3 (5.2)	2 (3.1)	2 (3.2)
Median (IQR) time since initiation (days); n missing	2 (1, 5); 0	2 (1, 3); 0	2 (1, 2); 0
Median (IQR) duration (days); n missing	1 (1, 1); 0	1 (1, 1); 0	1 (1, 1); 0

NA: not applicable

Table S4 Selected immunological therapies after randomisation by treatment arm

Drug	FOS (N=58)	RUX (N=64)	SOC (N=63)
Dexamethasone			
Received ≥1 dose, n (%)	0 (0.0)	2 (3.1)	4 (6.3)
Median (IQR) time since initiation (days); n missing	N/A	1 (1, 1); 0	2 (1, 3); 0
Median (IQR) duration (days), n missing	N/A	5 (4, 5); 0	17 (4, 29); 0
Prednisolone			
Received ≥1 dose, n (%)	6 (10.3)	4 (6.3)	2 (3.2)
Median (IQR) time since initiation (days); n missing	8 (8, 10); 0	16 (14, 22); 0	13 (13, 14); 0
Median (IQR) duration (days), n missing	22 (18, 25); 2	12 (8, 15); 0	24 (20, 24); 0
Methylprednisolone			
Received ≥1 dose, n (%)	3 (5.2)	2 (3.1)	0 (0.0)
Median (IQR) time since initiation (days); n missing	3 (1, 4); 0	3 (3, 3); 0	N/A
Median (IQR) duration (days), n missing	3 (1, 4); 0	3 (3, 3); 0	N/A
Hydrocortisone			
Received ≥1 dose, n (%)	3 (5.2)	1 (1.6)	1 (1.6)
Median (IQR) time since initiation (days); n missing	7 (5, 16); 0	8 (8, 8); 0	5 (5, 5); 0
Median (IQR) duration (days), n missing	2 (1, 3); 0	1 (1, 1); 0	3 (3, 3); 0
Tocilizumab			
Received ≥1 dose, n (%)	4 (6.9)	6 (9.4)	9 (14.3)
Median (IQR) time since initiation (days); n missing	2 (1, 8); 0	2 (1, 2); 0	3 (1, 4); 0
Median (IQR) duration (days), n missing	1 (1, 1); 0	1 (1, 1); 0	1 (1, 2); 0
Sarilumab			
Received ≥1 dose, n (%)	0 (0.0)	0 (0.0)	0 (0.0)
Median (IQR) time since initiation (days); n missing	N/A	N/A	N/A
Median (IQR) duration (days), n missing	N/A	N/A	N/A
Any IL6 therapy			
Received ≥1 dose, n (%)	4 (6.9)	6 (9.4)	9 (14.3)
Median (IQR) time since initiation (days); n missing	2 (1, 8); 0	2 (1, 2); 0	3 (1, 4); 0
Median (IQR) duration (days), n missing	1 (1, 1); 0	1 (1, 1); 0	1 (1, 2); 0
Baricitinib			
Received ≥1 dose, n (%)	0 (0.0)	0 (0.0)	1 (1.6)
Median (IQR) time since initiation (days); n missing	N/A	N/A	11 (11, 11); 0
Median (IQR) duration (days), n missing	N/A	N/A	11 (11, 11); 0

N/A: not applicable
