# Supplementary material for: Multiarm multistage randomised controlled trial of inflammatory signal inhibitors (MATIS) for patients hospitalised with COVID-19 pneumonia during the UK pandemic
Source: BMJ Open. 2026 Feb 5;16(2):e100583. doi: 10.1136/bmjopen-2025-100583 (PMC12887464; doi:10.1136/bmjopen-2025-100583)
Supplement: Supplementary data [file bmjopen-16-2-s004.pdf]

Supplementary Appendix 4

Co-enrolment to other randomised controlled trials

Table S5: Co-enrolment to other randomised clinical trials by treatment arm

Patient characteristic, n(%) unless otherwise specified	Fostamatinib (N=58)	Ruxolitinib (N=64)	Standard of care (N=63)
Co-enrolled in another study pre-randomisation n (%)			
Any randomised study	5 (8.6)	4 (6.3)	5 (7.9)
RECOVERY	3 (5.2)	3 (4.7)	2 (3.2)
REMAP-CAP	1 (1.7)	1 (1.6)	0 (0.0)
NOCoV2	0 (0.0)	0 (0.0)	1 (1.6)
C19-ACS	1 (1.7)	0 (0.0)	2 (3.2)
Co-enrolled in another study post-randomisation n (%)			
Any randomised study)	2 (3.4)	6 (9.4)	7 (11.1)
RECOVERY	1 (1.7)	1 (1.6)	3 (4.8)
REMAP-CAP	1 (1.7)	2 (3.1)	2 (3.2)
NOCoV2	0 (0.0)	2 (3.1)	2 (3.2)
C19-ACS	0 (0.0)	1 (1.6)	1 (1.6)
