# Supplementary material for: Multiarm multistage randomised controlled trial of inflammatory signal inhibitors (MATIS) for patients hospitalised with COVID-19 pneumonia during the UK pandemic
Source: BMJ Open. 2026 Feb 5;16(2):e100583. doi: 10.1136/bmjopen-2025-100583 (PMC12887464; doi:10.1136/bmjopen-2025-100583)
Supplement: Supplementary data [file bmjopen-16-2-s006.pdf]

Supplementary Appendix 6

Secondary Outcomes

Table S7: Secondary outcomes: Grade >=5 by Day 28

	N	n (%)	Adjusted odds ratio[a] (95% CI)
**Grade >=5 by Day 28[b]**			
Fostamatinib	58	16 (27.6)	1.17 (0.51 to 2.69)
Ruxolitinib	62	11 (17.7)	0.70 (0.29 to 1.72)
SOC	61	15 (24.6)	REF

[a] adjusted for baseline COVID severity, age category, use of IL6 inhibitor and prior COVID vaccination

[b] multiple imputation used to input missing outcome status for 4 patients in each arm

Table S8: Secondary outcomes up to Day 14

	N	n (%)	Adjusted odds ratio* (95% CI)
**Individual components of modified WHO scale**			
O2 saturation < 90% on ≥ 60% inspired oxygen			
Fostamatinib	55	16 (29.1)	1.84 (0.75 to 4.56)
Ruxolitinib	58	9 (15.5)	0.87 (0.32 to 2.37)
SOC	57	11 (19.3)	REF
Non-invasive ventilation			
Fostamatinib	55	13 (23.6)	0.95 (0.39 to 2.28)
Ruxolitinib	58	9 (15.5)	0.61 (0.24 to 1.56)
SOC	57	14 (24.6)	REF
Mechanical ventilation			
Fostamatinib	55	4 (7.3)	2.01 (0.35 to 11.66)
Ruxolitinib	58	3 (5.2)	1.58 (0.25 to 10.09)
SOC	57	2 (3.5)	REF
Mechanical ventilation with organ support			
Fostamatinib	54	3 (5.6)	3.02 (0.30 to 30.71)
Ruxolitinib	58	2 (3.4)	2.00 (0.17 to 23.44)
SOC	57	1 (1.8)	REF
Death			
Fostamatinib	55	4 (7.4)	1.60 (0.32 to 8.01)
Ruxolitinib	58	0 (0.0)	Not estimable
SOC	57	3 (5.5)	REF
**Maximum severity grade by Day 14**			
Grade 5			
Fostamatinib	55	1 (1.8)	Not estimable
Ruxolitinib	58	1 (1.7)	Not estimable
SOC	57	0 (0.0)	REF
Grade 6			
Fostamatinib	55	7 (12.7)	0.60 (0.21 to 1.69)
Ruxolitinib	58	6 (10.3)	0.52 (0.17 to 1.54)
SOC	57	11 (19.3)	REF
Grade 7			
Fostamatinib	55	1 (1.8)	0.95 (0.06 to 16.04)
Ruxolitinib	58	1 (1.7)	1.16 (0.07 to 19.73)
SOC	57	1 (1.8)	REF
Grade 8			
Fostamatinib	55	3 (5.5)	Not estimable
Ruxolitinib	58	2 (3.4)	Not estimable
SOC	57	0 (0.0)	REF
Grade 9			
Fostamatinib	55	4 (7.3)	1.60 (0.32 to 8.01)
Ruxolitinib	58	0 (0.0)	Not estimable
SOC	57	3 (5.3)	REF

*adjusted for baseline COVID severity, age category, use of IL6 inhibitor and prior COVID vaccination

Table S9: Individual components of modified WHO scale up to Day 28

Component of modified WHO scale	N	n (%)	Adjusted odds ratio[a] (95% CI)
O2 saturation < 90% on ≥ 60% inspired oxygen			
Fostamatinib	54	16 (29.6)	1.94 (0.78 to 4.82)
Ruxolitinib	58	10 (17.2)	0.97 (0.37 to 2.58)
SOC	57	11 (19.3)	REF
Non-invasive ventilation			
Fostamatinib	54	13 (24.1)	0.99 (0.41 to 2.37)
Ruxolitinib	58	10 (17.2)	0.68 (0.27 to 1.70)
SOC	57	14 (24.6)	REF
Mechanical ventilation			
Fostamatinib	54	4 (7.4)	1.95 (0.33 to 11.32)
Ruxolitinib	58	3 (5.2)	1.51 (0.24 to 9.71)
SOC	55	2 (3.6)	REF
Mechanical ventilation with organ support			
Fostamatinib	53	3 (5.7)	2.95 (0.29 to 30.05)
Ruxolitinib	57	3 (5.3)	3.14 (0.31 to 32.22)
SOC	55	1 (1.8)	REF
Death			
Fostamatinib	54	5 (9.3)	2.44 (0.51 to 11.71)
Ruxolitinib	58	2 (3.4)	0.67 (0.10 to 4.37);
SOC	55	3 (5.5)	REF

[a] adjusted for baseline COVID severity, age category, use of IL6 inhibitor and prior COVID vaccination

[b] multiple imputation used to input missing outcome status for 4 patients in each arm

Table S10: Maximum severity grade by Day 28

Maximum severity grade by Day 28	N	n (%)	Adjusted odds ratio[a] (95% CI)
Grade 5			
Fostamatinib	54	1 (1.9)	Not estimable
Ruxolitinib	58	1 (1.7)	Not estimable
SOC	55	0 (0.0)	REF
Grade 6			
Fostamatinib	54	7 (13.0)	0.76 (0.26 to 2.27)
Ruxolitinib	58	6 (10.3)	0.66 (0.21 to 2.04)
SOC	55	9 (16.4)	REF
Grade 7			
Fostamatinib	54	1 (1.9)	0.90 (0.05 to 15.48)
Ruxolitinib	58	0 (0.0)	Not estimable
SOC	55	1 (1.8)	REF
Grade 8			
Fostamatinib	54	2 (3.7)	Not estimable
Ruxolitinib	58	2 (3.4)	Not estimable
SOC	55	0 (0.0)	REF
Grade 9			
Fostamatinib	54	5 (9.3)	2.44 (0.51 to 11.71)
Ruxolitinib	58	2 (3.4)	0.67 (0.10 to 4.37)
SOC	55	3 (5.5)	REF

[a] adjusted for baseline COVID severity, age category, use of IL6 inhibitor and prior COVID vaccination

[b] multiple imputation used to input missing outcome status for 4 patients in each arm

Table S11: Change in odds of progressing to a worse severity between trial arms

Outcome definition	Adjusted[a] odds ratio (95% CI two-sided p-value)	
	Fostamatinib vs SOC	Ruxolitinib vs SOC
COVID severity at each time point (N=180[b])		
Day 1	1.00 (0.35 to 2.82); 0.994	0.68 (0.25 to 1.91); 0.469
Day 7	0.94 (0.31 to 2.89); 0.915	0.51 (0.16 to 1.57); 0.239
Day 14	2.37 (0.67 to 8.39); 0.182	0.77 (0.21 to 2.78); 0.689

[a] Adjusted for age category, baseline severity, use of steroid at baseline, use of IL6 inhibitor at baseline, received covid vaccine

[b] Includes N=180 ie all patients with at least one post-baseline outcome measurement

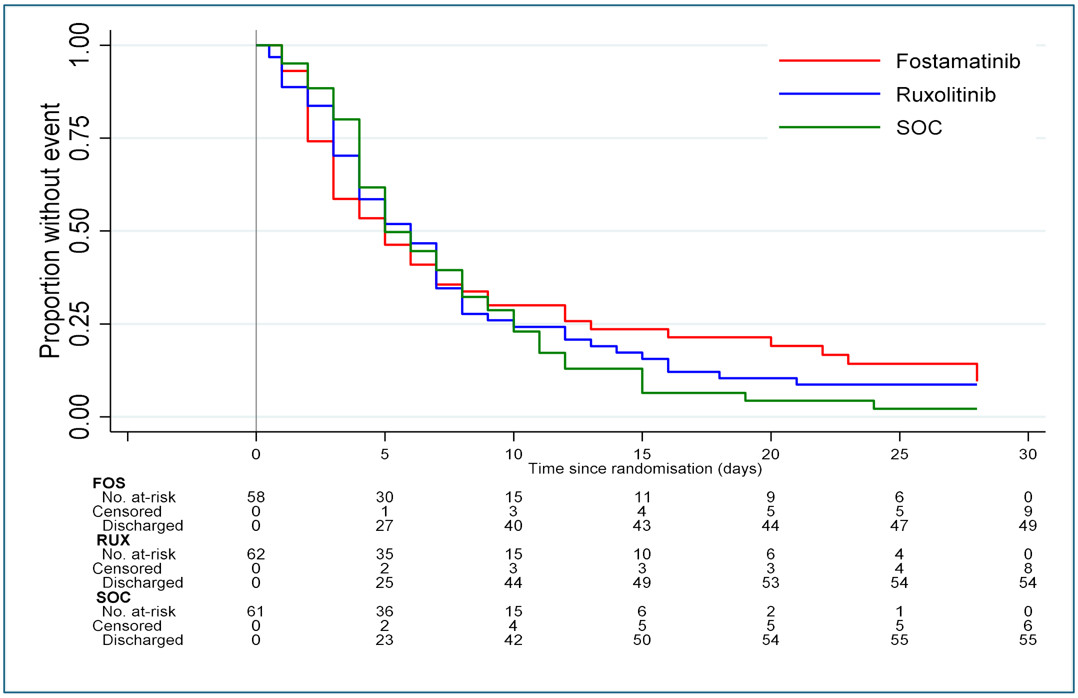

Figure S1: Kaplan Meier estimates of time to discharge (from randomisation) by treatment arm, censored at day 28[a]

[a] withdrawals and deaths are censored at the date of occurrence

Table S12: Readmissions by Day 28

Trial arm	N	n (%)	Adjusted odds ratio[a] (95% CI)
Fostamatinib	54	3 (5.6)	Not estimated
Ruxolitinib	58	8 (13.8)	Not estimated
SOC	55	8 (14.6)	Not estimated

Table S13: Results from mixed effects linear regression for inflammatory markers and serum creatinine

Marker	Fostamatinib vs SOC		Ruxolitinib vs SOC	
	Adjusted[a] mean difference	95% CI two-sided p-value	Adjusted[a] mean difference	95% CI two-sided p-value
**CRP (mg/L)**				
Day 1	-6.2	-22.4 to 10.0; 0.452	-7.7	-23.8 to 8.4; 0.350
Day 7	9.4	-8.2 to 27.0; 0.294	-11.9	-29.1 to 5.3; 0.176
Day 14	18.2	-6.1 to 42.5; 0.141	-6.8	-30.0 to16.3; 0.562
Day 28	3.7	-19.7 to 27.2; 0.755	-18.3	-40.5 to 3.8; 0.105
**LDH (IU/L)**				
Day 1	-11.8	-81.2 to 57.6; 0.739	-33.0	-106.7 to 40.7; 0.380
Day 7	39.5	-31.9 to 110.8; 0.278	56.6	-17.9 to 131.2; 0.136
Day 14	9.6	-84.5 to 103.7; 0.841	-34.7	-137.3 to 67.8; 0.507
Day 28	97.3	3.7 to 190.8; 0.042	54.3	-42.8 to 151.4; 0.273
**Ferritin (ug/L)**				
Day 1	278	-447 to 1003; 0.452	-101	-826 to 624; 0.784
Day 7	227	-487 to 941; 0.533	35	-668 to 739; 0.921
Day 14	29	-701 to 759; 0.938	-403	-1121 to 316; 0.272
Day 28	-20	-782 to 742; 0.959	-357	-1103 to 388; 0.347
**D-dimer (ng/ml)**				
Day 1	-360	-1191 to 471; 0.396	-97	-964 to 770; 0.826
Day 7	-487	-1325 to 351; 0.255	22	-808 to 853; 0.958
Day 14	85	-891 to 1062; 0.864	80	-864 to 1023; 0.869
Day 28	615	-388 to 1619; 0.229	401	-568 to 1370; 0.417
**Creatinine (μmol/L)[b]**				
Day 1	-10.1	-29.8 to 9.7; 0.317	-10.1	-29.7 to 9.5; 0.312
Day 7	12.3	-8.9 to 33.5; 0.255	16.1	-4.9 to 37.2; 0.133
Day 14	34.3	6.8 to 61.8; 0.014	21.2	-5.4 to 47.9; 0.119
Day 28	43.6	15.7 to 71.5; 0.002	27.2	0.6 to 53.7; 0.045

[a] adjusted for baseline value, site, use of steroid at baseline, use of IL6 inhibitor at baseline, age category and receipt of COVID vaccine; [b] analysis excludes three patients in RUX arm receiving dialysis
