# Supplementary figures and images for: Multiarm multistage randomised controlled trial of inflammatory signal inhibitors (MATIS) for patients hospitalised with COVID-19 pneumonia during the UK pandemic
Source: BMJ Open. 2026 Feb 5;16(2):e100583. doi: 10.1136/bmjopen-2025-100583 (PMC12887464; doi:10.1136/bmjopen-2025-100583)

## **Supplementary Appendix 8**

### **Adverse Events by System Organ Class**

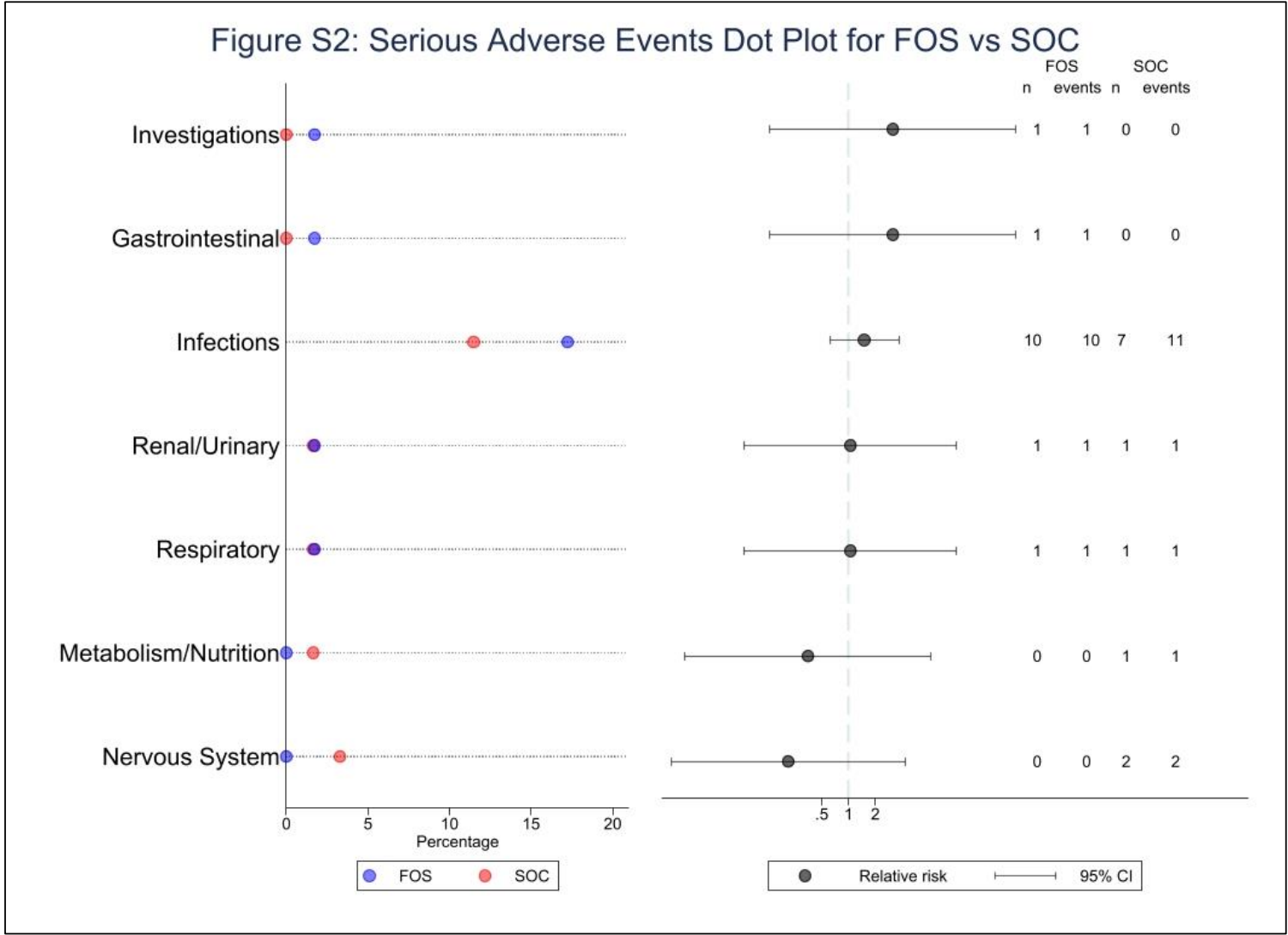

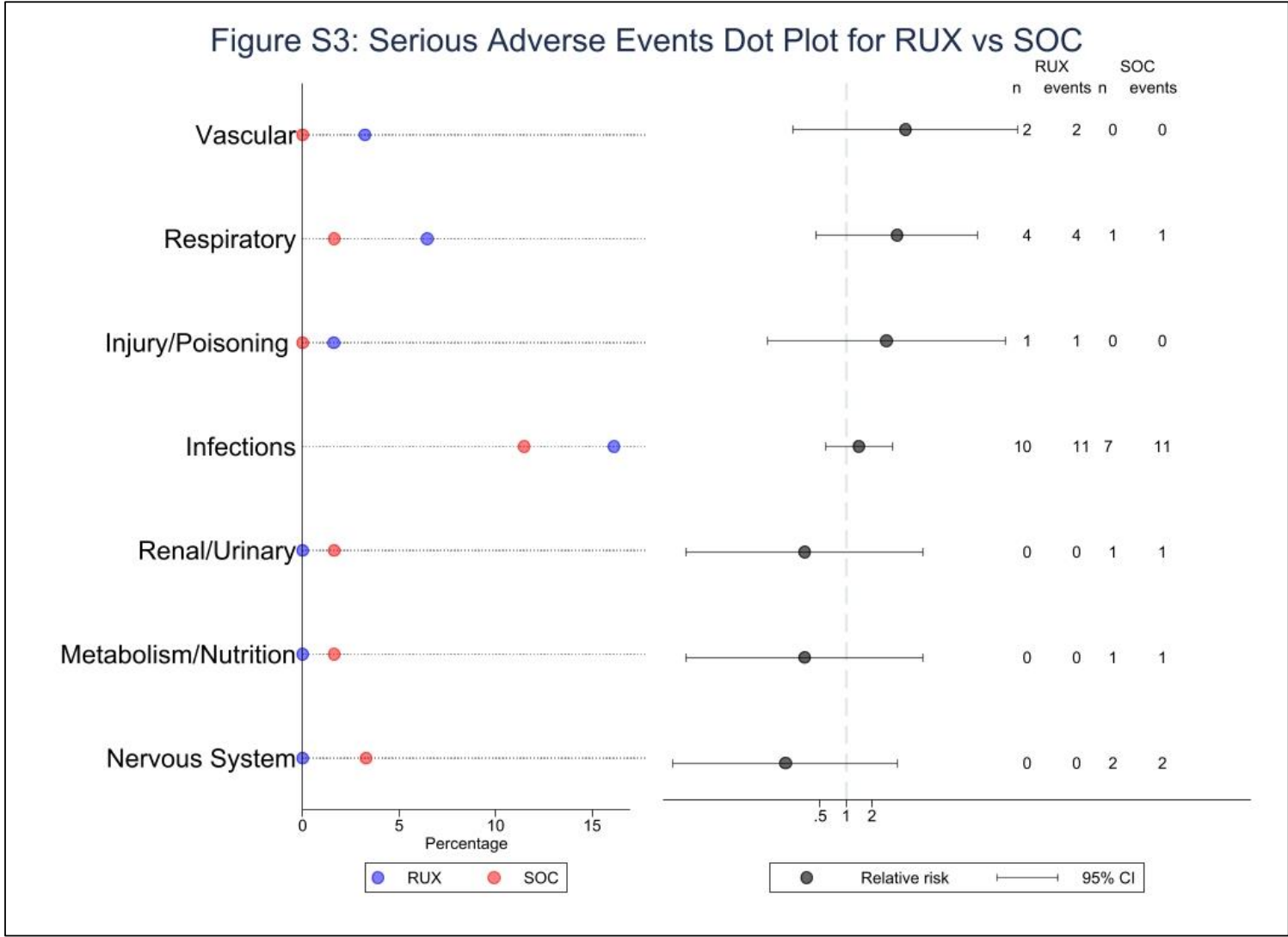

Supplement: Supplementary data [file bmjopen-16-2-s008.pdf]
