# Supplementary material for: Multiarm multistage randomised controlled trial of inflammatory signal inhibitors (MATIS) for patients hospitalised with COVID-19 pneumonia during the UK pandemic
Source: BMJ Open. 2026 Feb 5;16(2):e100583. doi: 10.1136/bmjopen-2025-100583 (PMC12887464; doi:10.1136/bmjopen-2025-100583)
Supplement: Supplementary data [file bmjopen-16-2-s007.pdf]

Supplementary Appendix 7

Exploratory analyses

Table S14: Primary outcome model results in patients who received ≥ 90% of prescribed dose

Per-protocol	No. (%) of compliers	Adjusted[a] odds ratio (95% CI; p-value[b])	
		**ITT**	**Per-protocol**
Fostamatinib	40 (69.0)	1.19 (0.51 to 2.76); 0.681	1.19 (0.47 to 2.99); 0.716
Ruxolitinib	46 (74.2)	0.63 (0.25 to 1.57); 0.323	0.80 (0.31 to 2.06); 0.641
SOC	61 (100.0)	REF	REF
**CACE**	**No. (%) of compliers**	**Adjusted[a] risk difference (95% CI; p-value[b])**	
		**ITT**	**CACE**
Fostamatinib	40 (69.0)	0.03 (-0.12 to 0.17); 0.729	0.05 (-0.18 to 0.28); 0.642
Ruxolitinib	46 (74.2)	-0.06 (-0.17 to 0.06); 0.328	-0.10 (-0.29 to 0.10); 0.328
SOC	61 (100.0)	REF	REF

[a] adjusted for baseline COVID severity, age category, use of IL6 inhibitor, prior COVID vaccination; model run on imputed dataset

[b] p-values are two-sided

Table S15: Primary outcome model results stratified by time of randomisation

Grade >=5 by Day 14	N			Odds ratio (95% CI; p-value[a])	
	**FOS**	**RUX**	**SOC**	**Fostamatinib vs SOC**	**Ruxolitinib vs SOC**
Stratified analysis by time of enrolment					
Quartile 1 (Jan 2021)	16	15	15	0.45 (0.10 to 2.01; 0.299)	0.44 (0.09 to 2.15; 0.313)
Quartile 2 (Feb 2021)	15	16	14	4.71 (0.73 to 30.28; 0.102)	1.50 (0.21 to 10.81; 0.687)
Quartile 3 (Sep 2021)	15	15	15	Not estimable	0.92 (0.15 to 5.53; 0.924)
Quartile 4 (Aug 2022)	12	16	17	3.10 (0.56 to 16.96; 0.193)	Not estimable

a p-values are two-sided
