# Supplementary material for: Multiarm multistage randomised controlled trial of inflammatory signal inhibitors (MATIS) for patients hospitalised with COVID-19 pneumonia during the UK pandemic
Source: BMJ Open. 2026 Feb 5;16(2):e100583. doi: 10.1136/bmjopen-2025-100583 (PMC12887464; doi:10.1136/bmjopen-2025-100583)
Supplement: Author's manuscript [file bmjopen-2025-100583.draft_revisions.pdf]

BMJ Open is committed to open peer review. As part of this commitment we make the peer review history of every article we publish publicly available.

When an article is published we post the peer reviewers' comments and the authors' responses online. We also post the versions of the paper that were used during peer review. These are the versions that the peer review comments apply to.

The versions of the paper that follow are the versions that were submitted during the peer review process. They are not the versions of record or the final published versions. They should not be cited or distributed as the published version of this manuscript.

BMJ Open is an open access journal and the full, final, typeset and author-corrected version of record of the manuscript is available on our site with no access controls, subscription charges or pay-per-view fees (http://bmjopen.bmj.com).

If you have any questions on BMJ Open's open peer review process please email info.bmjopen@bmj.com

BMJ Open

Multi-arm multi-stage randomised controlled trial of Inflammatory Signal Inhibitors (MATIS) for hospitalised patients with mild or moderate COVID-19 pneumonia

Journal:	*BMJ Open*
Manuscript ID	bmjopen-2025-100583
Article Type:	Original research
Date Submitted by the Author:	18-Feb-2025

Complete List of Authors:	Hazell, Lorna; Imperial College London, Imperial College Clinical Trials Unit<br>Pillay, Clio; Imperial College London, Department of Immunology and Inflammation; Imperial College Healthcare NHS Trust<br>Cornelius, Victoria; Imperial College London, Imperial College Clinical Trials Unit<br>Phillips, Rachel; Imperial College London, Imperial College Clinical Trials Unit<br>Charania, Asad; Imperial College London, Department of Immunology and Inflammation; Imperial College Healthcare NHS Trust<br>Wason, James; Newcastle University, Population Health Sciences Institute<br>Cherlin, Svetlana; Newcastle University, Population Health Sciences Institute<br>Savic, Sinisa; Leeds Teaching Hospitals NHS Trust, Immunology; University of Leeds,<br>Whittington, Ashley; Northwick Park Hospital, Infectious Diseases and Acute Medicine<br>Neelakantan, Pratap; Royal Berkshire NHS Foundation Trust, Haemato-Oncology<br>Collini, Paul; Sheffield Teaching Hospitals NHS Foundation Trust, Department of Infectious Diseases<br>Cook, Lucy; Imperial College London, Department of Immunology and Inflammation; Imperial College Healthcare NHS Trust<br>Willicome, Michelle; Imperial College London, Department of Immunology and Inflammation; Imperial College Healthcare NHS Trust<br>Milojkovic, Dragana; Imperial College London, Department of Immunology and Inflammation; Imperial College Healthcare NHS Trust<br>Kon, Onn Min; Imperial College Healthcare NHS Trust, Respiratory Medicine; Imperial College London, National Heart and Lung Institute<br>Youngstein, Taryn; Imperial College London, National Heart and Lung Institute; Imperial College Healthcare NHS Trust<br>Innes, Andrew; Imperial College London, Department of Immunology and Inflammation; Imperial College Healthcare NHS Trust<br>Thursz, Mark; Imperial College London, Department of Metabolism, Digestion and Reproduction; Imperial College Healthcare NHS Trust<br>Cooke, Graham; Imperial College London, Department of Infectious Diseases; Imperial College Healthcare NHS Trust<br>Vergis, Nikhil; Imperial College London, Department of Immunology and Inflammation; Imperial College Healthcare NHS Trust

	Cooper, Nichola; Imperial College London, Department of Immunology and Inflammation; Imperial College Healthcare NHS Trust

Keywords:	Clinical Trial, COVID-19, Drug Therapy

SCHOLARONE™ Manuscripts

I, the Submitting Author has the right to grant and does grant on behalf of all authors of the Work (as defined in the below author licence), an exclusive licence and/or a non-exclusive licence for contributions from authors who are: i) UK Crown employees; ii) where BMJ has agreed a CC-BY licence shall apply, and/or iii) in accordance with the terms applicable for US Federal Government officers or employees acting as part of their official duties; on a worldwide, perpetual, irrevocable, royalty-free basis to BMJ Publishing Group Ltd ("BMJ") its licensees and where the relevant Journal is co-owned by BMJ to the co-owners of the Journal, to publish the Work in this journal and any other BMJ products and to exploit all rights, as set out in our licence.

The Submitting Author accepts and understands that any supply made under these terms is made by BMJ to the Submitting Author unless you are acting as an employee on behalf of your employer or a postgraduate student of an affiliated institution which is paying any applicable article publishing charge ("APC") for Open Access articles. Where the Submitting Author wishes to make the Work available on an Open Access basis (and intends to pay the relevant APC), the terms of reuse of such Open Access shall be governed by a Creative Commons licence – details of these licences and which Creative Commons licence will apply to this Work are set out in our licence referred to above.

Other than as permitted in any relevant BMJ Author's Self Archiving Policies, I confirm this Work has not been accepted for publication elsewhere, is not being considered for publication elsewhere and does not duplicate material already published. I confirm all authors consent to publication of this Work and authorise the granting of this licence.

For peer review only

CONFIDENTIAL PRE-PUBLICATION MANUSCRIPT

TITLE: Multi-arm multi-stage randomised controlled trial of Inflammatory Signal Inhibitors (MATIS) for hospitalised patients with mild or moderate COVID-19 pneumonia in the UK

Authors:

Lorna Hazell, Trial Statistician (0000-0002-5962-0648) * [1]

Dr Clio Pillay, Trial Manager* [2,3]

Professor Victoria Cornelius, Professor in Medical Statistics and Trials Methodology (0000-0002-0080-1065) [1]

Dr Rachel Phillips, Senior Lecturer in Medical Statistics and Clinical Trials (0000-0002-3634-7845) [1]

Dr Asad Charania (0000-0001-8908-4535) [2,3]

Professor James M. S. Wason, Professor of Biostatistics (0000-0002-4691-126X) [4]

Dr Svetlana Cherlin, Senior Research Associate (0000-0001-9689-3699) [4]

Professor Sinisa Savic, Professor of Clinical Immunology (0000-0001-7910-0554) [5]

Dr Ashley Whittington, Consultant in Infectious Diseases and Acute Medicine [6]

Dr Pratap Neelakantan, Consultant Haematologist [7]

Dr Paul Collini, Senior Clinical Lecturer in Infectious Diseases (0000-0001-6696-6826) [8]

Dr Lucy Cook, Consultant Haematologist (0000-0002-8234-5090) [2,3]

Dr Michelle Willicome, Clinical Reader in Transplantation [2,3]

Dr Dragana Milojkovic, Consultant Haematologist [2,3]

Professor Onn Min Kon, Respiratory Consultant (0000-0003-2647-4688) [3,9]

Dr Taryn Youngstein, Consultant Rheumatologist (0000-0002-8297-556X) [3,9]

Dr Andrew J. Innes, Honorary Clinical Senior Lecturer (0000-0003-0918-8882) [2,3]

For peer review

CONFIDENTIAL PRE-PUBLICATION MANUSCRIPT

Professor Mark Thurz, Director of Academic Health Science Centre (0000-0002-8218-192X) [3,10]

Professor Graham S. Cooke, Deputy Dean of the Faculty of Medicine (0000-0001-6475-5056) [3,11]

Dr Nikhil Vergis, Honorary Clinical Senior Lecturer (0000-0002-4157-6757) [$ 2,3]

Professor Nichola Cooper, Professor of Immune Haematology (0000-0002-9845-8292) [$ 2,3]

Corresponding author:

Nichola Cooper, n.cooper@imperial.ac.uk

[*] Both authors contributed equally to this work.

[$] Both authors contributed equally to this work.

For peer review only

CONFIDENTIAL PRE-PUBLICATION MANUSCRIPT

Author Affiliations

1. Imperial Clinical Trials Unit, Imperial College London, UK
2. Department of Immunology and Inflammation, Imperial College London, UK
3. Imperial College NHS Healthcare Trust, London, UK.
4. Population Health Sciences Institute, Newcastle University, Newcastle upon Tyne, UK
5. Clinical Immunology and Allergy, Leeds Teaching Hospitals NHS Trust, Leeds, UK.
6. Infectious Diseases and Acute Medicine, Northwick Park Hospital, London, UK
7. Department of Haematology, Royal Berkshire Hospital, Reading, UK
8. Department of Infectious Diseases, Sheffield Teaching Hospitals NHS Foundation Trust, Sheffield, UK
9. National Heart and Lung Institute, Imperial College London, UK
10. Department of Metabolism, Digestion and Reproduction, Imperial College London, UK
11. Department of Infectious Diseases, Imperial College London, UK

For peer review only

Copyright/license for publication

The Corresponding Author has the right to grant on behalf of all authors and does grant on behalf of all authors, an exclusive licence (or non-exclusive for government employees) on a worldwide basis to the BMJ Publishing Group Ltd to permit this article (if accepted) to be published in BMJ editions and any other BMJPGL products and sublicences such use and exploit all subsidiary rights, as set out in our licence.

Competing interest statement

All authors have completed the Unified Competing Interest form (available on request from the corresponding author). LH, CP, RP, AW, PN, PC, LC, MT and NV declare no support from any organisation for the submitted work; no financial relationships with any organisations that might have an interest in the submitted work in the previous three years and no other relationships or activities that could appear to have influenced the submitted work. VC is a co-investigator leading statistical design, methods, and analysis for the MATIS trial, funded by Novartis and Rigel, is a committee member for the NIHR Academy Doctoral board, a DMEC member for the NIHR HTA-funded BAY Project. Behavioural Activation for Young people with depression in specialist child and adolescent mental health services and is chair of the DMC for NIHR HTA-funded RADAR: Research into Antipsychotic Discontinuation and Reductions. AC has been an employee of Roche since 2022. DM has received honorary payments from Novartis, Incyte and Ascentage Pharma, has participated on an advisory board for Ascentage Pharma and has received investigator-led institutional research funding from Incyte. MW has received honorary payments from AstraZeneca, has participated on an advisory board or data monitoring committee for

For peer review only - http://bmjopen.bmj.com/site/about/guidelines.xhtml

CONFIDENTIAL PRE-PUBLICATION MANUSCRIPT

Hansa Pharmaceuticals, received equipment, materials, drugs, medical writing, gifts or other services from Moderna and has had a leadership or fiduciary role in other board, society, committee or advocacy group, paid or unpaid for Hansa Pharmaceuticals. OMK has participated on an advisory board or data monitoring committee for the NIHR DILI study and has had team members funded by grants from Cepheid. GC has participated in an end point review committee for Sanofi and is a board member for the MHRA. SS holds the Chair of British Society for Immunology-Clinical immunology professional network. JW and SC have had team members funded by grants from Novartis and Rigel. TY is recipient of a grant from UKRI MRC BHF Cardio-IMID Partnership, has received consulting fees for the Ad Board Abbvie 2024 and is Treasurer for the UK and Ireland Vasculitis and Rare Disease Group. AJI has received consulting fees, honoraria for speakers' bureaus, international travel support and/or personal fees from Novartis and Incyte. NC has received research grants from Novartis and Rigel for conducting MATIS, and honoraria for consultancy from Novartis, Sobi, Sanofi, Amgen, Argenyx.

Contributorship and guarantor information

Contributors: NC conceived the research idea and led the trial. NC attained funding for the trial. NC, CP, SS, AC, AW, PN, PC, LC, MW, DM, OMK, TY, AJI, MT, GC and NV were principal investigators or investigators and contributed to the inception, trial management, data collection and/or completion of the trial. RP, JW, VC and NC were responsible for the study design and statistical analysis plan. LH, SC and RP

For peer review only - http://bmjopen.bmj.com/site/about/guidelines.xhtml

CONFIDENTIAL PRE-PUBLICATION MANUSCRIPT

conducted the statistical analysis. LH wrote the first draft of the manuscript with input from RP, VC, CP and NC. All authors approved of the submitted version.

The corresponding author (NC) attests that all listed authors meet authorship criteria and that no others meeting the criteria have been omitted. NC, as guarantor, accepts full responsibility for the work and/or the conduct of the study, had access to the data, and controlled the decision to publish.

Transparency declaration

NC affirms that the manuscript is an honest, accurate, and transparent account of the study being reported; that no important aspects of the study have been omitted; and that any discrepancies from the study as originally planned (and registered) have been explained.

Role of the funding source

The MATIS trial was sponsored by Imperial College London and was jointly funded by NIHR Imperial Biomedical Research Centre, Novartis, the manufacturer of ruxolitinib (Jakavi®) and Rigel, the manufacturer of fostamatinib (Tavlesse®).

The sponsor and funders had no control over the study design; data collection, analysis, or interpretation of data; in the writing of the report; or in the decision to

For peer review only - http://bmjopen.bmj.com/site/about/guidelines.xhtml

submit the article for publication. All authors confirm independence from the funders, had full access to all of the data (including statistical reports and tables) and take responsibility for the integrity of the data and the accuracy of the data analysis.

Trial registration details

The MATIS trial was registered prospectively in ClinicalTrials.gov (NCT04581954 and EUDRA-CT (2020-001750-22) before enrolling the first participant.

Summary box

What is already known on this topic

- Adaptive clinical trial designs such as the multi-arm multi-stage design can help to focus research efforts towards more promising treatments.
- During the COVID-19 pandemic, there was an urgent need to identify safe and effective treatments to reduce mortality and healthcare burden.
- The potential role of immunomodulator therapies in the treatment of COVID-19 pneumonia is uncertain.

What this study adds

- We found no evidence that fostamatinib reduced progression to severe COVID-19 pneumonia in patients with mild to moderate disease compared to routine care.
- Results for ruxolitinib were more favourable but could not be confirmed due to early termination of our trial. Further research is needed.

For peer review only - http://bmjopen.bmj.com/site/about/guidelines.xhtml

CONFIDENTIAL PRE-PUBLICATION MANUSCRIPT

Abstract

Objectives: To determine the safety and efficacy of ruxolitinib and fostamatinib compared to standard of care (SOC) for the treatment of mild to moderate COVID-19 pneumonia.

Design: Adaptive multi-arm, multi-stage, randomised, open label trial (3-arm, 2-stage)

Setting: Five hospitals in England between October 2020 and September 2022.

Participants: Hospitalised patients (≥18 years) with mild/moderate COVID-19 pneumonia defined by a modified WHO COVID severity grade <5.

Interventions: Participants were randomly assigned 1:1:1 to receive ruxolitinib (10mg bd for 7 days then 5mg bd for 7 days), fostamatinib (150mg bd for 7 days then 100mg bd for 7 days) or SOC.

Main outcome measures: Primary outcome was development of severe COVID pneumonia (modified WHO severity grade ≥5) within 14 days of randomisation. Secondary outcomes included mortality, invasive and non-invasive ventilation, venous thromboembolism, duration of hospital stay, readmissions, inflammatory markers and serious adverse events (SAE).

For peer review only - http://bmjopen.bmj.com/site/about/guidelines.xhtml

CONFIDENTIAL PRE-PUBLICATION MANUSCRIPT

Results: At Stage 1, 181 patients were randomised, with 4 assessed as ineligible post-randomisation. Fostamatinib was stopped early for futility with 16 participants (27.6%, N=58) developing severe COVID pneumonia compared to 15 (25.0%, N=60) in the SOC arm (adjusted odds ratio (aOR) compared to SOC: 1.12; 95% confidence interval (CI): 0.49 to 2.58; p value = 0.608). Ruxolitinib progressed to Stage 2 but the trial was stopped early due to slow recruitment. At the final analysis, a lower proportion of participants developed severe COVID pneumonia in the ruxolitinib arm (n=10, 16.1%, N=62) than in the SOC arm (15, 24.6%, N=61) (aOR: 0.63; 95% CI: 0.25 to 1.57; p=0.161). Four (7.4%) participants in the fostamatinib arm, none in the ruxolitinib arm and three (5.5%) in the SOC arm died within 14 days of randomisation. Infections were the most frequently reported serious adverse event and were numerically higher in the fostamatinib (10, 17.2%) and ruxolitinib (10, 16.1%) arms compared to SOC (7, 11.5%). Two unexpected serious adverse reactions occurred in the ruxolitinib arm only.

Conclusions: We found no evidence that fostamatinib was superior to SOC for the treatment of mild to moderate COVID-19 pneumonia. The ruxolitinib treatment effect estimate was in favour of benefit, but due to early stopping, the trial was underpowered to confirm ruxolitinib's benefit in this population. Further study is needed.

Trial registration: NCT04581954; EUDRA-CT: 2020-001750-22

For peer review only - http://bmjopen.bmj.com/site/about/guidelines.xhtml

Introduction

Severe COVID-19 pneumonia is characterised by respiratory and multi-organ failure in the context of marked systemic inflammation and increased thrombotic risks. Elevations in circulating inflammatory molecules are associated with poor prognosis and in a subset of patients, drive acute lung injury which, in the early stages of the 2020 pandemic, resulted in high rates of catastrophic multi-organ failure and death.[1]

Therapeutic interventions targeting inflammatory signalling might reduce the severity of the inflammatory response phase and associated lung damage thereby averting respiratory failure and the need for mechanical ventilation. Data from China and Italy during the pandemic suggested that immediate resolution of symptoms could be achieved using anti-interleukin 6 (IL6) therapy and Janus kinase (JAK) inhibitors in patients with severe disease.[2 3] The SYK pathway was also considered a potential target; it is activated by many receptors, including C-type lectin (CLEC) or C-type lectin-like receptors (CLR), and Fc receptors.[4] Inhibition of the SYK pathway has been shown to inhibit platelet aggregation in heparin-Induced thrombocytopenia models,[5] a disease with many similar features to COVID-19.

We hypothesised that there may be an early window of opportunity to treat the COVID-19 hyperinflammatory syndrome before acute lung injury leads to organ failure. We designed a randomised controlled, multi-arm multi-stage trial in patients with mild to moderate COVID pneumonia to evaluate the effect of early intervention

For peer review only - http://bmjopen.bmj.com/site/about/guidelines.xhtml

with two inflammatory signal inhibitors which are already licensed for use in other clinical indications: ruxolitinib (JAK inhibitor) [6] and fostamatinib (small molecule spleen tyrosine kinase (SYK) inhibitor).[7]

Methods

Trial design and participants

The Multi-Arm Trial of Inflammatory Signal Inhibitors for COVID-19 (MATIS) was an open-label multi-arm, multi-stage randomised (1:1:1) controlled trial (RCT) of ruxolitinib (RUX) and fostamatinib (FOS) for COVID-19 pneumonia compared to routine standard of care (SOC). Eligible participants were hospitalised patients in the UK with mild to moderate COVID pneumonia defined by a severity grade less than 5 on the modified World Health Organisation (WHO) severity scale (Table 1).[8]

Table 1. Modified WHO COVID-19 Severity Scale, from the WHO R&D Blueprint

Patient state	Descriptor	Grade
Uninfected	No clinical or virological evidence of infection	0
Ambulatory	No limitation of activities	1
	Limitation of activities	2
Hospitalised mild disease	Hospitalised, no oxygen therapy	3
	Oxygen by mask or nasal prongs	4
Hospitalised severe disease	SpO_2< 90% on FiO_2 ≥60% by face mask	5
	Non-invasive ventilation, CPAP or high-flow oxygen	6
	Intubation and mechanical ventilation	7
	Ventilation + additional organ support (vasopressors, RRT, ECMO)	8
Dead	Death	9

Published in the COVID Therapeutic Trial Synopsis, February 18th 2020[8]

For peer review only - http://bmjopen.bmj.com/site/about/guidelines.xhtml

CONFIDENTIAL PRE-PUBLICATION MANUSCRIPT

The trial design (Figure 1) is further described in the trial protocol.[9] A planned formal interim analysis (Stage 1) was conducted on 10th March 2022 after 177 eligible participants had been randomised and completed 14 days of follow-up. A pre-specified threshold of one-sided p-value ≤0.25 was required to progress treatment with RUX and/or FOS to Stage 2. The Independent Data Monitoring Committee (IDMC) recommended stopping the FOS arm for futility at Stage 1 (see Results section). Recruitment continued for the RUX and SOC arms, however, due to the slow recruitment because of the reduction in new COVID-19 cases, the trial was stopped early on 2nd September 2022 after only recruiting an additional 4 participants. Therefore, the final analysis presented in this manuscript includes all three trial arms as if they had been a conventional one-stage RCT comparing RUX and FOS with SOC.

Randomisation and blinding

Participants were randomly allocated to RUX, FOS or SOC using a central web-based randomisation service that used randomisation sequences with random block sizes stratified by age (<65 vs ≥65) and study site. Staff treating participants, participant-facing research staff and participants were unblind to study treatment. IDMC reports prior to the Stage 1 analysis were prepared by the trial statistician who was subgroup blind. Stage 1 analysis was performed by an independent, unblind statistician at Newcastle University. The final analyses were undertaken by an unblind trial statistician at Imperial College Clinical Trials Unit. All analyses were undertaken after the Statistical Analysis Plan was signed off.

For peer review only - http://bmjopen.bmj.com/site/about/guidelines.xhtml

CONFIDENTIAL PRE-PUBLICATION MANUSCRIPT

Interventions

The planned duration of treatment was 14 days. Participants in the RUX arm were prescribed 10mg twice daily for 7 days reducing to 5mg twice daily for a further 7 days. Participants in the FOS arm were prescribed 150mg twice daily for 7 days reducing to 100mg twice daily for a further 7 days. Study treatment was given in combination with SOC. Due to the evolving clinical landscape for the management of COVID-19, the SOC was not predefined and varied during the course of the trial (Tables S2 and S3). Co-enrolment to other trials, additional treatment and rescue therapy was permitted at the discretion of the treating clinicians.

Follow up

Participants were followed up at 1, 7, 14 and 28 days after randomisation. Those discharged from hospital before Day 14 completed trial medication at home and were followed up by telephone or using patient health records, where available, up to Day 28.

Outcomes

All comparisons were between RUX or FOS versus SOC. The primary outcome was defined as the proportion with severe COVID-19 pneumonia within 14 days of randomisation, where severe COVID-19 pneumonia was defined by a modified WHO COVID-19 Ordinal Grade ≥ 5 (on the 9-point scale, Table 1).[8] Secondary outcomes, examined at Day 14 and Day 28 included change in severity on the modified WHO COVID-19 Ordinal Scale (Grade 3 or 4 to 5,6,7,8,9), individual components of the primary outcome (mortality, non-invasive ventilation including continuous positive airway pressure (CPAP) and high flow nasal oxygen, invasive ventilation), organ

For peer review only

support, venous thromboembolism, length of hospital stay, readmission within 28 days, inflammatory markers (C-reactive protein (CRP), lactose dehydrogenase (LDH), ferritin, D-dimer), serum creatinine and serious adverse events (SAEs). Non-serious adverse events were not collected.

Sample size

A sample size of 171 (57 per arm) participants at Stage 1 and an additional maximum of 285 (95 per arm) was selected to provide power of 90% (minimum marginal power) with a maximum 5% chance of an intervention arm being recommended when it provides no improvement over control (5% one-sided family-wise error rate), inflated by 5% for missing outcome data (full details are described in the trial protocol).[9]

Statistical analysis

Participant flow through the trial was described using a Consolidated Standards of Reporting Trials (CONSORT) flow chart.[10] A scatter plot was used to show the trajectory of participant recruitment over time. Simple descriptive statistics were used to summarise baseline participant characteristics, co-enrolment to other trials and SOC medications received at baseline and post-randomisation.

The modified intention-to-treat (ITT) population included all eligible randomised participants analysed in the treatment arm to which they were allocated to regardless of treatment subsequently received. Participants found to have been randomised in error were excluded from the modified ITT population. Participants were assumed to have received 14 days of treatment unless there was evidence for stopping earlier.

For peer review only - http://bmjopen.bmj.com/site/about/guidelines.xhtml

CONFIDENTIAL PRE-PUBLICATION MANUSCRIPT

For the primary outcome, we used a generalized linear model with a binomial distribution and logit link function to compare the odds of developing severe COVID pneumonia between RUX or FOS and SOC. At Stage 1, two separate models were used to compare the effects of FOS vs SOC and RUX vs SOC. These models were adjusted for using a propensity score derived from a logistic regression model that included baseline WHO COVID-19 severity grade (3 or 4), age (<65 or ≥65), receipt of steroids at baseline and receipt of interleukin-6 (IL6) inhibitors at baseline.

For the final analysis, we included all three trial arms in a single model. Baseline WHO COVID-19 severity grade (3 or 4), age (<65 or ≥65), receipt of interleukin-6 (IL6) inhibitors at the time of or prior to randomisation and prior vaccination against COVID-19 were included as separate covariates in this model. As there were several sites with very few randomised participants, we excluded study site from the primary analysis models. Multiple imputation was used to account for any missing data such that all eligible randomised participants were included in both the Stage 1 and final analyses (See Supplementary Appendix 3).[11] Treatment effects were reported as odds ratios with corresponding 95% confidence intervals (CI) and one-sided p-values.

The original two-stage design specified that these would be judged at the 0.027 level (i.e. a two-sided p-value < 0.054 was to be used to indicate a significant treatment effect). However, as the planned sample size was not reached, the p-values presented should not be interpreted with reference to this threshold as the trial was significantly underpowered. Interpretation should be focused on the point estimate and 95% confidence interval as a way to assess the evidence of a treatment effect.

For peer review only - http://bmjopen.bmj.com/site/about/guidelines.xhtml

Analysis of secondary outcomes was performed for the final analysis only. Here, the primary analysis model was repeated to compare the odds of developing severe pneumonia by Day 28. This model was also run separately for each individual component of the primary outcome and the maximum COVID severity grade reached by Day 14 and Day 28. In addition, COVID severity grade was modelled as an ordinal variable using a mixed ordinal logistic regression model to compare the odds of progression to more severe disease at Day 14. Mixed linear regression models were used to compare changes in inflammatory markers and serum creatinine up to Day 28 and a Cox proportional hazards model was used to compare the rates of hospital discharge between trial arms. The frequency of venous thromboembolism, readmission within 28 days and SAE were summarised descriptively. Dot plots were used to visually display between group differences in SAE frequencies (as risk ratios) grouping events at system organ class level.

Sensitivity analyses were undertaken to examine alternative approaches to handling missing data firstly, assuming that all participants with missing outcome had poor outcome e.g., all missing assumed severity grade >=5, and secondly assuming that all participants with missing outcome had good outcome e.g., all missing assumed severity grade <5. Additional exploratory analysis assessing the impact of participant adherence[12], time of enrolment to the trial and subgroup analysis of the primary outcome by age, sex, BMI, cardiovascular disease, chronic lung disease, end-stage-renal failure, liver cirrhosis, diabetes, baseline CRP and D-dimer, immunocompromisation, smoking status and number of days since onset of COVID at randomisation are briefly described in the Supplementary Appendices 5 and 6).

For peer review only - http://bmjopen.bmj.com/site/about/guidelines.xhtml

A planned sensitivity analysis of the primary outcome adjusting for receipt of effective COVID treatments through co-enrolment to the active arm of another randomised controlled was not performed since such treatments were not found to have been prescribed to trial participants as part of co-enrolment to another trial. Another sensitivity analysis to explore both the direct effect of treatment on the outcome and any indirect effect of receiving known effective treatments as rescue medications was not conducted as all of the participants who received these medications post-randomisation to MATIS received the medication on or after the index date for the primary outcome.

Stage 1 analysis was performed in R (https://www.R-project.org/). All other analysis was undertaken using Stata/IC version 16.1, (StataCorp, College Station, TX, USA).

Patient and Public Involvement

Information on MATIS trial design was included on clinicaltrial.gov. A twitter page was set up to explain the trial to a wider audience, and the protocol was published in Trials (2021).[9]

Ethics

The Surrey Research Ethics Committee (REC) and Health Regulator Authority (HRA) reviewed and granted approval for this trial (IRAS ID 282552). The study was conducted in accordance with the recommendations for physicians involved in research on human subjects adopted by the 18th World Medical Assembly, Helsinki 1964 and later revisions.[13]

For peer review only - http://bmjopen.bmj.com/site/about/guidelines.xhtml

Results

Participant flow and baseline characteristics

Between October 2020 and September 2022,185 patients from 5 UK hospitals in England were enrolled (Figure 2). Recruitment followed the peaks and troughs of the COVID pandemic waves observed in the UK during the trial period (Figure 3).

During the 28-day follow-up, 13 participants withdrew consent and one was lost to follow-up. Four patients found to be ineligible post-randomisation were excluded from the analyses. A slightly higher proportion of participants completed follow up but discontinued study medication early in the FOS arm (13, 22.4%) than in the RUX arm (11, 17.2%). Thirty-eight (66.7%) participants in the FOS arm and 46 (71.9%) in the RUX arm were assumed to have completed the 14-day treatment course.

A higher proportion of participants in the RUX arm compared to the SOC arm were female (35.9% vs. 30.2%), were of white ethnicity (48.4% vs. 42.9%), had a COVID severity grade of 3 at baseline (21.9% vs. 14.3%) or had received a COVID vaccination (25.0% vs. 17.5%) (Table 2). In contrast, these characteristics were less prevalent in the FOS arm compared to SOC, with the exception of the baseline severity grade of 3 (17.2% in FOS vs. 13.3% in SOC).

For peer review only - http://bmjopen.bmj.com/site/about/guidelines.xhtml

CONFIDENTIAL PRE-PUBLICATION MANUSCRIPT

Table 2: Baseline characteristics by treatment arm

Patient characteristic, n (%) unless otherwise specified	Fostamatinib (N=58)	Ruxolitinib (N=64)	Standard of care (N=63)
Age in years			
Mean (SD)	58.9 (15.0)	60.5 (15.8)	59.1 (16.3)
Sex			
Male	43 (74.1)	41 (64.1)	44 (69.8)
Female	15 (25.9)	23 (35.9)	19 (30.2)
Ethnicity			
White	21 (36.2)	31 (48.4)	27 (42.9)
Mixed or multiple ethnic groups	1 (1.7)	0 (0.0)	1 (1.6)
Asian or Asian British	7 (12.1)	11 (17.2)	8 (12.7)
Black, Black British, Caribbean or African	5 (8.6)	7 (10.9)	7 (11.1)
Other ethnic group	24 (41.4)	15 (23.4)	20 (31.7)
BMI in kg/m2			
N (N missing)	55 (3)	57 (7)	60 (3)
Mean (SD)	30.1 (6.5)	30.6 (7.1)	29.9 (6.9)
Severity of Covid			
Grade 3	10 (17.2)	14 (21.9)	9 (14.3)
Grade 4	48 (82.8)	50 (78.1)	54 (85.7)
Chronic lung disease			
N (N missing)	58 (0)	62 (2)	63 (0)
Yes	7 (12.1)	12 (19.4)	11 (17.5)
Diabetes			
N (N missing)	58 (0)	62 (2)	63 (0)
Yes	15 (25.9)	19 (30.6)	20 (31.7)
Hypertension			
N (N missing)	58 (0)	62 (2)	63 (0)
Yes	25 (43.1)	28 (45.2)	28 (44.4)
Ischaemic heart disease			
N (N missing)	58 (0)	62 (2)	63 (0)
Yes	11 (19.0)	10 (16.1)	10 (15.9)
Heart failure			
N (N missing)	58 (0)	62 (2)	63 (0)
Yes	2 (3.4)	3 (4.8)	2 (3.2)
Immunocompromised			
N (N missing)	58 (0)	62 (2)	63 (0)
Yes	1 (1.7)	5 (8.1)	2 (3.2)
End-stage renal failure			
N (N missing)	58 (0)	62 (2)	63 (0)
Yes	4 (6.9)	6 (9.7)	3 (4.8)
Liver cirrhosis			

For peer review only - http://bmjopen.bmj.com/site/about/guidelines.xhtml

Patient characteristic, n (%) unless otherwise specified	Fostamatinib (N=58)	Ruxolitinib (N=64)	Standard of care (N=63)
N (N missing)	58 (0)	62 (2)	63 (0)
Yes	0 (0.0)	0 (0.0)	0 (0.0)
Current smoker			
N (N missing)	58 (0)	62 (2)	62 (1)
Yes	0 (0.0)	2 (3.2)	3 (4.8)
Prior Covid Vaccination			
N (N missing)	58 (0)	64 (0)	63 (0)
Yes	8 (13.8)	16 (25.0)	11 (17.5)
Serum creatinine (umol/L)			
N (N missing)	56 (2)	59 (0)	61 (0)
Mean (SD)	133 (202)	89 (56)	121 (174)

The majority of participants in all three trial arms were receiving anticoagulant therapy at baseline (Table 3), most were receiving an immuno-modulating agent, specifically dexamethasone (Supplementary Table S1) and just under half had received an antiviral agent (Table 3). More participants in the SOC arm had received antibiotics at baseline (74.6%) compared to the FOS (58.6%) or RUX (64.1%) arms whereas fewer participants initiated antibiotics after randomisation in the SOC arm (22.2%) compared to the FOS (32.8%) and RUX (25.0%) arms (Supplementary Table S2). While fewer participants in the SOC (14.3%) arm had received an interleukin-6 (IL6) inhibitor at baseline compared to the FOS (19.0%) and RUX (20.3%) arms (Supplementary Table S1), these were initiated more frequently after randomisation in the SOC arm (14.3%) compared to the FOS (6.9%) and RUX (9.4%) arms (Supplementary Table S3).

For peer review only - http://bmjopen.bmj.com/site/about/guidelines.xhtml

CONFIDENTIAL PRE-PUBLICATION MANUSCRIPT

Table 3: Standard of care treatments received at or prior to randomisation by treatment arm

Drug group	FOS (N=58)	RUX (N=64)	SOC (N=63)
Anti-viral agents			
Received ≥1 dose, n (%)	27 (46.6)	31 (48.4)	29 (46.0)
Median (IQR) time since initiation (days); n missing	0 (0, 1); 0	0 (0, 1); 0	0 (0, 1); 0
Median (IQR) duration (days); n missing	5 (4, 6); 0	5 (5, 6); 0	5 (4, 5); 0
Immuno-modulating agents			
Received ≥1 dose, n (%)	56 (96.6)	58 (90.6)	56 (88.9)
Median (IQR) time since initiation (days); n missing	1 (0, 1); 0	1 (0, 1); 0	1 (0, 1); 0
Median (IQR) duration (days); n missing	6 (4, 9); 0	8 (5, 11); 0	7 (4, 10); 1
Antibiotics			
Received ≥1 dose, n (%)	34 (58.6)	41 (64.1)	47 (74.6)
Median (IQR) time since initiation (days); n missing	1 (0, 2); 0	1 (0, 2); 0	1 (1, 2); 0
Median (IQR) duration (days); n missing	5 (2, 7); 0	4 (2, 7); 0	6 (2, 7); 0
Anticoagulation treatment			
Received ≥1 dose, n (%)	54 (93.1)	54 (84.4)	57 (90.5)
Median (IQR) time since initiation (days); n missing	1 (0, 1); 0	1 (0, 1); 0	1 (0, 1); 0
Median (IQR) duration (days); n missing	9 (5, 25); 1	9 (5, 13); 1	9 (6, 13); 0
Antiplatelet therapy			
Received ≥1 dose, n (%)	6 (10.3)	1 (1.6)	2 (3.2)
Median (IQR) time since initiation (days); n missing	1 (0, 1); 0	1 (1, 1); 0	2 (0, 2); 0
Median (IQR) duration (days); n missing	17 (3, 30); 4	Missing	1 (1, 1); 0
Convalescent serum therapy			
Received ≥1 dose, n (%)	0 (0.0)	1 (1.6)	1 (1.6)
Median (IQR) time since initiation (days); n missing	N/A	6 (6, 6); 0	0 (0, 0); 0
Median (IQR) duration (days); n missing	N/A	1 (1, 1); 0	2 (2, 2); 0
Ronapreve®			
Received ≥1 dose, n (%)	1 (1.7)	1 (1.6)	0 (0.0)
Median (IQR) time since initiation (days); n missing	0 (0, 0); 0	0 (0, 0); 0	N/A
Median (IQR) duration (days); n missing	1 (1, 1); 0	1 (1, 1); 0	N/A

N/A: not applicable

For peer review only

CONFIDENTIAL PRE-PUBLICATION MANUSCRIPT

Prior to randomisation, similar proportions (approximately 6-9%) of participants had been co-enrolled on another RCT across the three trial arms. Co-enrolment after randomisation to MATIS was higher in the SOC arm (11.1%) and RUX arm (9.4%) than in the FOS arm (3.4%) (Supplementary Table S4).

Primary outcome

At Stage 1 (N=177), there was no evidence of a reduction in the odds of progression to severe COVID pneumonia for FOS: adjusted odds ratio, compared to SOC: 1.12; 95% confidence interval (CI): 0.49 to 2.58; one-sided p value = 0.608 (Table 4). Futility was declared for the FOS arm on the basis of the one-sided p-value exceeding the prespecified threshold of 0.25[9] while recruitment to the RUX (and SOC) arm was recommended to continue (p=0.152, Table 4).

Table 4: Primary Outcome Statistical Model Results

Grade >=5 by Day 14	N	n[a] (%)	Unadjusted		Adjusted[b]	
			Odds ratio (95% CI)	p-value[c]	Odds ratio (95% CI)	p-value[c]
Stage 1						
Fostamatinib	58	16 (27.6)	1.13 (0.50 to 2.58)	0.766	1.12 (0.49 to 2.58)	**0.608**
Ruxolitinib	59	10 (16.9)	0.62 (0.25 to 1.53)	0.300	0.60 (0.22 to 1.60)	**0.152**
SOC	60	15 (25.0)	1.0		1.0	
Final Analysis						
Fostamatinib	58	16 (27.6)	1.19 (0.52 to 2.71)	0.684	1.19 (0.51 to 2.76)	**0.659**
Ruxolitinib	62	10 (16.1)	0.59 (0.24 to 1.44)	0.243	0.63 (0.25 to 1.57)	**0.161**
SOC	61	15 (24.6)	1.0		1.0	

[a] multiple imputation used at Stage 1 and final analysis to impute primary outcome status for 3 patients in the FOS arm, 4 in the RUX arm and 4 in the SOC arm.

[b] Stage1: propensity score adjusted; Final analysis: adjusted for baseline COVID severity, age category, use of IL6 inhibitor and prior COVID vaccination

For peer review only - http://bmjopen.bmj.com/site/about/guidelines.xhtml

[c] p-values are two-sided except for those in **bold** where one-sided p-values are presented; p-values for final analysis are nominal as planned sample size not reached

In the final analysis (N=181, Table 4), the numbers (%) of participants with severe COVID pneumonia by Day 14 were 16 (27.6%, N=58) and 10 (16.1%, N=62) in the FOS and RUX arms respectively, and 15 (24.6%, N=61) in the SOC arm.

There remained a numerically higher odds of reaching a COVID severity grade ≥5 in the FOS arm compared to SOC (OR: 1.19; 95% CI: 0.51 to 2.76; nominal one-sided p-value: 0.659).

In contrast, a numerically lower odds of severe COVID pneumonia was observed for RUX compared to SOC (OR: 0.63; 95% CI: 0.25 to 1.57; p=0.161). In both comparisons, the 95% confidence intervals were wide and included 1. Study conclusions remained unchanged when applying alternative missing data assumptions (Supplementary Table S5).

Secondary outcomes

Results from the primary analysis model when extended to include data up to Day 28 (COVID severity grade≥ 5 by Day 28) were very similar to that for Day 14 (Supplementary Table S6). The primary outcome at Day 14 and Day 28 was most commonly triggered by participants experiencing oxygen saturation levels < 90% on receipt of ≥ 60% inspired oxygen or receipt of non-invasive ventilation (Supplementary Tables S7 to S9). Seven participants died (4 in the FOS arm and 3 in the SOC arm) on or before Day 14 and a further three died (2 in the RUX arm and 1 in the SOC arm) on or before Day 28. Individual models for each of the components of the primary outcome are presented, however, event counts are small and resulting model estimates are imprecise. Results from the mixed ordinal logistic

For peer review only - http://bmjopen.bmj.com/site/about/guidelines.xhtml

regression model were broadly consistent with the primary analysis model (Supplementary Table S10).

Nine participants (4 in the FOS arm, 3 in the RUX arm and 2 in the SOC arm) received mechanical ventilation by Day 14, of whom 6 received organ support (inotropes) (Table Supplementary S7). Six participants (2 in the FOS arm, 3 in the RUX arm and 1 in the SOC arm) had a venous thromboembolism (VTE) event including one participant with a serious VTE in the SOC arm. Two participants (one in the RUX and one in the FOS arm) had the VTE event on the day of randomisation.

There was no evidence of a difference in the time to discharge from randomisation for either FOS or RUX compared to SOC (Supplementary Figure S1). Hazard ratios from the Cox model were 0.96 (95% CI 0.64 to 1.43) for FOS vs SOC and 1.05 (95% CI 0.71 to 1.55) for RUX vs SOC, after adjusting for age, site, prior COVID vaccination, receipt of steroids and receipt of IL6 inhibitors at baseline. Readmission by Day 28 was more frequent in the RUX (8 participants, 13.8%) and SOC (8, 14.6%) arms than in the FOS arm (3, 5.6%) (Supplementary Table S11). No difference in levels of inflammatory markers was observed between trial arms (Supplementary Table S12). Statistically significant differences in serum creatinine levels were noted with higher mean values in the FOS arm at Day 14 (adjusted mean difference vs. SOC: 34.3 μmol/L; 95% CI 6.8 to 61.8) and at Day 28 (43.6 μmol/L; 95% CI 15.7 to 71.5) and in the FOS arm at Day 28 (adjusted mean difference vs. SOC: 27.2 μmol/L; 95% CI 0.6 to 53.7) (Supplementary Table S12).

Safety

For peer review only - http://bmjopen.bmj.com/site/about/guidelines.xhtml

Safety was evaluated in all 181 eligible, randomised participants. Four patients were excluded from this analysis as they were randomised in error. A total of 48 SAEs were reported in 38 participants with a higher proportion experiencing at least one SAE in the RUX (14, 22.6% of participants) and FOS arms (13, 22.4%) compared to SOC (11, 17.5%) (Table 5).

Table 5: Summary of Serious Adverse Events type by treatment arm

	**Fostamatinib (N=58)**		**Ruxolitinib (N=62)**		**Standard of care (N=61)**	
Event type	Number of events	Number of participants with events (%)	Number of events	Number of participants with events (%)	Number of events	Number of participants with events (%)
SAE	14	13 (22.4)	18	14 (22.6)	16	11 (17.5)
SAR	1	1 (1.7)	2	2 (3.2)	0	0 (0.0)
USAR	0	0 (0.0)	2[a]	2 (3.2)	0	0 (0.0)

Acronyms: SAE – serious adverse event; SAR – serious adverse reaction; USAR – unexpected serious adverse reaction

[a] This included one patient with hypotension and one patient with lymphoedema

Three of the reported SAEs were considered to be possibly related to study treatment. These were classified as serious adverse reactions (SARs) and included one expected SAR in the FOS arm (lung infection) and two unexpected SARs in the RUX arm (one case each of hypotension and lymphoedema). The most frequently reported SAE in all three trial arms were infections with numerically higher frequencies in the FOS (10, 17.2%) and ruxolitinib (10, 16.1%) arms compared to SOC (7, 11.5%) (Supplementary Figures S2 and S3). These included 5 cases of lung infection in RUX arm (4 participants) and 3 cases in in the FOS arm (3 participants), both higher than the SOC arm (1 participant). One event of lung

For peer review only - http://bmjopen.bmj.com/site/about/guidelines.xhtml

infection in the RUX arm resulted in a fatal outcome. None of the 10 fatal events recorded in the trial were considered to be related to study treatment.

Exploratory analyses

Results from the per-protocol analysis in participants who received ≥ 90% of prescribed dose were consistent with the primary analysis (Supplementary Table S13). The complier-adjusted risk difference (RD) estimates were slightly further away from the null than the corresponding ITT analyses but did not reach statistical significance (complier-adjusted vs ITT: FOS vs SOC: RD 0.05 vs 0.03; RUX vs SOC: RD -0.10 vs -0.06). Stratification of the analysis by grouping participants into approximately four equal sized groups based on time of randomisation resulted in somewhat imprecise estimates and were inconclusive (Supplementary Table S14). There was no strong evidence for any subgroup effects (Supplementary Figures S4 and S5). These results should be interpreted with caution due to the small sample size within subgroups.

Discussion

Principal findings

MATIS was an open-label, multi-centre, multi-arm, and multi-stage randomised controlled trial of ruxolitinib and fostamatinib for COVID-19 pneumonia compared to routine standard of care. The study was designed between March and April 2020, with participants recruited largely from the second and third waves of the UK pandemic, with the majority of participants recruited from three hospitals in North-

For peer review only - http://bmjopen.bmj.com/site/about/guidelines.xhtml

West London. Due to the success of the COVID-19 vaccine leading to a dramatic reduction in hospitalisation and mortality, the study was closed in September 2022, before reaching the power needed to conclude its end points.

Despite not having power for a definitive outcome, there was a suggestive finding indicating lower odds (OR: 0.63) of progressing to severe COVID-19 pneumonia (grade ≥5) associated with ruxolitinib compared to SOC, even though the wide 95% confidence interval (0.25 to 1.57) and a p-value of 0.161 warrant cautious interpretation of the findings. In contrast, there was no suggestive finding indicating lower odds for fostamatinib. In fact, the odds were higher (OR 1.19) for progressing to severe COVID-19 pneumonia associated with fostamatinib compared to SOC. Although, again, there was a wide 95% confidence interval (0.51 to 2.76) and a p-value of 0.659.

Although, the study was not powered to assess the secondary outcome of death, no deaths were recorded in the ruxolitinib arm in the first 14 days while 4 participants in the fostamatinib arm and 3 in the SOC arm dies during this period. There was no difference in time to discharge between either active treatment and SOC, but there were fewer readmissions in the fostamatinib arm. In terms of safety, there were higher numbers of lung infections in the active treatment arms including one infection-related death in the ruxolitinib arm, however this event was not considered to be related to study treatment. We noted an increase in serum creatinine in both arms, however the clinical relevance of this finding is unclear.

Comparison with other studies

For peer review only - http://bmjopen.bmj.com/site/about/guidelines.xhtml

Ruxolitinib is an inhibitor of the JAK/STAT pathway, specifically inhibiting subtypes JAK1 and JAK2. It has a rapid mode of action with cytokine-induced STAT3 phosphorylation being inhibited withing 2 hours of administration, resulting in a reduction of tumour necrosis factor alpha (TNFα), IL6 and CRP. Several other trials studied the effect of ruxolitinib during the pandemic, with variable responses reported, some positive, but most were neutral.[14-16] There were many differences between studies including: types of patients recruited (i.e. differing severity of COVID-19), timing of the study in the pandemic (resulting in variability of the virus over time, changes in standard of care; and the vaccine role out in 2021), and different primary outcomes. As with MATIS, some of these trials were underpowered due to early termination. Furthermore, the appropriate dosing of ruxolitinib to treat or prevent hyperinflammation is yet to be determined. We chose a low to intermediate dose for MATIS, because we had concerns regarding immunosuppression during active phase of the infection. This dose appeared to be appropriate for this cohort of patients given the lower rate of progression to more severe disease and absence of deaths before 14 days in the ruxolitinib arm. Higher doses such as 25mg once a day, used in conditions such as graft versus host disease,[17] or longer duration of treatment (>14 days) may be more appropriate for patients with more severe COVID-19.

Given the CRP and IL6 drive in COVID-19, several clinical trials of other agents in this class (including baracitinib and tofacitinib) targeting patients at different disease stages were performed.[18 19] A meta-analysis of four randomised controlled trials of baracitinib in hospitalised patients with COVID-19, including 10,815 patients, showed a reduction in 28-day mortality (OR 0.69, 95% CI 0.5-0.94; p=0.04).[20] Results from two of the large studies included in the meta-analysis, RECOVERY and COV-

For peer review only - http://bmjopen.bmj.com/site/about/guidelines.xhtml

BARRIER,[21 22] led to the US Food and Drug Administration (FDA), allowing the emergency authorisation of baracitinib with remdesivir in patients with COVID-19 requiring supplemental oxygen, invasive mechanical ventilation, or extracorporeal membrane oxygenation (ECMO).[23]

The RECOVERY study also observed a 15% reduction mortality for tocilizumab (an IL6 inhibitor) compared to usual care.[24] Tociluzimab was added to standard of care during MATIS and was used in approximately one fifth of the patients in this trial.

Fostamatinib is a spleen tyrosine kinase (SYK) inhibitor, licensed for use in patients with immune thrombocytopenia. The SYK pathway appears to be critical in the pathogenesis of COVID-19 as well as other severe acute respiratory syndrome (SARS) infections, modulating cytokine secretion, and promoting the influx of immune cells such as macrophages and monocyte into the lung, contributing to both the hyperinflammatory state and long-term lung damage. As such, SYK inhibition appeared as a novel mechanism to treat COVID-19 pneumonia through the prevention of cytokine release, lung inflammation and platelet activation, all critical events in COVID-19 pathway. [25 26]

Despite a plausible biological mechanism of action, we found no evidence that fostamatinib prevented progression of COVID-19 to more severe disease in this cohort of patients. It is possible that our patient cohort were too mildly affected to have any benefit from fostamatinib. It is also possible we did not use the correct dose, or duration of fostamatinib. Another RCT comparing fostamatinib 100 mg twice daily + SOC to placebo +SOC included patients with more severe COVID-19 (over half of the patients required non-invasive ventilation or high-flow oxygen devices [56.7% fostamatinib and 51.7% placebo], or invasive mechanical ventilation and/or

For peer review only - http://bmjopen.bmj.com/site/about/guidelines.xhtml

ECMO [36.7% FOS and 41.1% placebo]).[27] In that study, the rates of SAEs by Day 29 (the primary end point) were not statistically different between the fostamatinib group and the placebo group (10.5% vs 22.0%, p-value=0.2), while secondary endpoints, which the study was not powered for, such as 28-day mortality, days on supplemental oxygen and ordinal scores of clinical status favoured fostamatinib. Both the inclusion criteria and the dose of fostamatinib were different to MATIS limiting their comparison, but potentially implying the timing of the use of fostamatinib in diseases such as COVID-19 might be critical.

Another double blind, randomized, placebo-controlled trial of fostamatinib in patients with COVID-19 on supplementary oxygen (the FOCUS trial) recruited 280 patients.[28] Although results are not yet published in full, the FOCUS trial met its primary endpoint with patients in the fostamatinib arm having a reduced number of days on supplemental oxygen compared to placebo (4.8 vs. 7.6 days, respectively, p=0.0136). Fostamatinib also showed a trend in reducing COVID-19 related mortality and morbidity. Unlike MATIS, this study also recruited patients with more severe COVID-19; all 3 participants with a baseline score of 6 on the 8-point COVID ordinal scale in the placebo group died by Day 30, and all 3 participants with this baseline score in the fostamatinib group survived, suggesting there may be particular benefit for patients with more severe COVID-19, although numbers in this cohort remain small.

The study designs were different for these trials, limiting comparisons, but overall, it is possible that the timing of the use of fostamatinib in diseases such as COVID-19 is critical, with greater benefit in patients with more severe disease, preventing the

For peer review only - http://bmjopen.bmj.com/site/about/guidelines.xhtml

progression of lung disease, rather than earlier in the infection, when anti-viral responses may be more important.

Strength and Limitations:

Patient recruitment:

Despite a strong study design, we were unable to recruit the numbers of participants needed to detect a treatment effect for ruxolitinib or fostamatinib due to reducing numbers of patients being admitted to hospital as the pandemic evolved. Recruitment to MATIS was also limited by competition from other large concurrent COVID trials. Another limitation is the representativeness of patients in the trial in terms of age of the patients recruited. Older patients, who had the highest mortality in COVID-19, although approached for MATIS, were less likely to consent to the trial. We do not know the reason for this, but possible causes include family members declining study; fear of the medical profession; fear of the virus; patients being too unwell to read the patient information sheet; flaws in recruitment strategies in a fast-moving time. On the other hand, the demographics of participants showed broad ethnic representation (approximately 40% of participants were white and 60% were non-white), reflecting the recruitment from inner city hospitals and was a strength of the study.

Evolving Standard of Care

At or prior to randomisation, 46.0% to 48.4% of patients in MATIS received anti-viral agents, 88.9% to 96.6% received immune-modulating agents, 58.6% to 74.6%

For peer review only - http://bmjopen.bmj.com/site/about/guidelines.xhtml

received antibiotics, 84.4% to 93.1% received anticoagulation treatment, and 1.6% to 10.3% received antiplatelet therapy. However, the standard of care for treating COVID-19 changed over time, reflecting the dynamic nature of the evolving pandemic. Initially treatments were focused on supportive care strategies. As standard of care evolved, antiviral medications, anti-inflammatory agents, and eventually vaccination campaigns were integrated into treatment protocols. In 2021, there was a successful role out of vaccines. The changes in recommended treatments over distinct phases of the pandemic created additional complexity to COVID-19 treatment clinical trials impacting on patient profile, recruitment numbers and the changing use of multiple immunomodulatory agents. Within MATIS, the use of IL6-inhibitors was introduced at Imperial College NHS Trust (the main study site) for patients who were developing hyper-inflammation. We note that fewer patients in the active treatment arms required treatment with an IL6 inhibitor post-randomisation.

Implications for practice and future research

One of the unique contributing factors to the high individual mortality, as well as the population level impact of COVID-19, was the triggering of a hyperinflammatory state causing a rapid escalation to ventilation in many patients. The excessive need for ventilation also impacted the rest of the population with hospitals running out of ventilators and intensive care staff.

In designing MATIS, we chose two different immune modulators as early interventions (within the first few days of admission), aiming to reduce inflammation, prevent the requirement for ventilation and improve the outcome for these patients, as well as reducing the impact of COVID-19 on UK hospitals.

For peer review only - http://bmjopen.bmj.com/site/about/guidelines.xhtml

To identify patients who we thought were at risk of hyperinflammation, and therefore more likely to benefit from immune modulation, we included patients with a raised CRP. However, this may not be specific enough. The RuxCoFlam trial which showed a significant effect of ruxolitinib selected patients based on a composite COVID-19 inflammation score (CIS) made up of multiple inflammatory markers.[29] This included inter alia CRP, ferritin, triglcycerides, fibrinogen, total white blood cell count, lymphocytes and D-dimer and activated partial thromboplastin time. For CRP to contribute to the CIS score, values were required to exceed 20 times the upper limit of the normal reference range.

Subsequent research, including evidence from our own studies, has shown the importance of host genetic factors in COVID-19 mortality demonstrating an exaggerated hyperinflammatory response to this novel virus.[30,31] Testing patients for these host factors, and measuring specific immune responses, may allow us to target patients more effectively for immune modulation, and clarify which modulators are most appropriate at an individual level. A biomarker study is now underway in samples stored prior to randomisation of MATIS to see if we can better fine-tune immune modulation therapy in virally induced hyperinflammation.

Other patient characteristics such as age, obesity, diabetes and cardiovascular risk factors are independently associated with the development of secondary infection thrombosis, requirement for ventilation and death.[32] Meta-analysis of JAK/STAT inhibition from all studies may help identify the cohorts of patients most likely to benefit from these treatments.

For peer review only

The timing and choice of immune-modulation in diseases such as COVID-19 could be critical for improving outcome, giving the immune system time to effectively eradicate the virus, but preventing the rapid escalation to hyperinflammation.

The results of these studies are likely to be important in guiding the management of other virally induced multisystemic disorders.

Conclusion

In conclusion, we did not observe a significant treatment effect for fostamatinib in comparison to standard of care in reducing the proportion of patients progressing from mild or moderate to severe COVID-19 pneumonia. Results for ruxolitinib were more favourable but could not be confirmed due to early termination of our trial. Other factors such as patient comorbidities, choice of dose and timing of treatment may also have contributed to these findings. Further research is needed to help us to further understand these results and further investigation of the role of JAK/STAT inhibition in hyperinflammatory conditions triggered by viruses is now warranted, as well as long term follow up of patients who received immune modulation.

For peer review only

CONFIDENTIAL PRE-PUBLICATION MANUSCRIPT

References

1. Huang C, Wang Y, Li X, et al. Clinical features of patients infected with 2019 novel coronavirus in Wuhan, China. Lancet 2020;395(10223):497-506. doi: 10.1016/S0140-6736(20)30183-5 [published Online First: 20200124]

2. Ruan Q, Yang K, Wang W, et al. Clinical predictors of mortality due to COVID-19 based on an analysis of data of 150 patients from Wuhan, China. Intensive Care Med 2020;46(5):846-48. doi: 10.1007/s00134-020-05991-x [published Online First: 20200303]

3. Siddiqi HK, Mehra MR. COVID-19 illness in native and immunosuppressed states: A clinical-therapeutic staging proposal. J Heart Lung Transplant 2020;39(5):405-07. doi: 10.1016/j.healun.2020.03.012 [published Online First: 20200320]

4. Spalton JC, Mori J, Pollitt AY, et al. The novel Syk inhibitor R406 reveals mechanistic differences in the initiation of GPVI and CLEC-2 signaling in platelets. J Thromb Haemost 2009;7(7):1192-9. doi: 10.1111/j.1538-7836.2009.03451.x [published Online First: 20090424]

5. Lhermusier T, van Rottem J, Garcia C, et al. The Syk-kinase inhibitor R406 impairs platelet activation and monocyte tissue factor expression triggered by

For peer review only - http://bmjopen.bmj.com/site/about/guidelines.xhtml

CONFIDENTIAL PRE-PUBLICATION MANUSCRIPT

heparin-PF4 complex directed antibodies. J Thromb Haemost 2011;9(10):2067-76. doi: 10.1111/j.1538-7836.2011.04470.x

6. Novartis. Jakavi 10mg tablets. 2022 [Available from: https://www.medicines.org.uk/emc/product/7786/smpc#gref.

7. Grifols UK Ltd. Tavlesse 100 mg film-coated tablets 2023 [Available from: https://www.medicines.org.uk/emc/product/11479/smpc#gref accessed 7th August 2024.

8. World Health Organisation. COVID-19 Therapeutic Trial Synopsis. Geneva Switzerland: World Health Organisation, 2020.

9. Vergis N, Phillips R, Cornelius V, et al. Multi-arm Trial of Inflammatory Signal Inhibitors (MATIS) for hospitalised patients with mild or moderate COVID-19 pneumonia: a structured summary of a study protocol for a randomised controlled trial. Trials 2021;22(1):270. doi: 10.1186/s13063-021-05190-z [published Online First: 20210412]

10. Schulz KF, Altman DG, Moher D, et al. CONSORT 2010 statement: updated guidelines for reporting parallel group randomised trials. BMJ 2010;340:c332. doi: 10.1136/bmj.c332 [published Online First: 20100323]

11. White IR, Royston P, Wood AM. Multiple imputation using chained equations: Issues and guidance for practice. Stat Med 2011;30(4):377-99. doi: 10.1002/sim.4067 [published Online First: 20101130]

12. Cook JA, MacLennan GS, Palmer T, et al. Instrumental variable methods for a binary outcome were used to informatively address noncompliance in a randomized trial in surgery. J Clin Epidemiol 2018;96:126-32. doi: 10.1016/j.jclinepi.2017.11.011 [published Online First: 20171120]

For peer review only - http://bmjopen.bmj.com/site/about/guidelines.xhtml

13. Rickham PP. Human Experimentation. Code of Ethics of the World Medical Association. Declaration of Helsinki. Br Med J 1964;2(5402):177. doi: 10.1136/bmj.2.5402.177

14. Cao Y, Wei J, Zou L, et al. Ruxolitinib in treatment of severe coronavirus disease 2019 (COVID-19): A multicenter, single-blind, randomized controlled trial. J Allergy Clin Immunol 2020;146(1):137-46 e3. doi: 10.1016/j.jaci.2020.05.019 [published Online First: 20200526]

15. Han MK, Antila M, Ficker JH, et al. Ruxolitinib in addition to standard of care for the treatment of patients admitted to hospital with COVID-19 (RUXCOVID): a randomised, double-blind, placebo-controlled, phase 3 trial. Lancet Rheumatol 2022;4(5):e351-e61. doi: 10.1016/S2665-9913(22)00044-3 [published Online First: 20220329]

16. Rein L, Calero K, Shah R, et al. Randomized Phase 3 Trial of Ruxolitinib for COVID-19-Associated Acute Respiratory Distress Syndrome. Crit Care Med 2022;50(12):1701-13. doi: 10.1097/CCM.0000000000005682 [published Online First: 20221013]

17. Broglie L, Pommert L, Rao S, et al. Ruxolitinib for treatment of refractory hemophagocytic lymphohistiocytosis. Blood Adv 2017;1(19):1533-36. doi: 10.1182/bloodadvances.2017007526 [published Online First: 20170817]

18. Guimaraes PO, Quirk D, Furtado RH, et al. Tofacitinib in Patients Hospitalized with Covid-19 Pneumonia. N Engl J Med 2021;385(5):406-15. doi: 10.1056/NEJMoa2101643 [published Online First: 20210616]

19. Kalil AC, Patterson TF, Mehta AK, et al. Baricitinib plus Remdesivir for Hospitalized Adults with Covid-19. N Engl J Med 2021;384(9):795-807. doi: 10.1056/NEJMoa2031994 [published Online First: 20201211]

For peer review only - http://bmjopen.bmj.com/site/about/guidelines.xhtml

20. Selvaraj V, Finn A, Lal A, et al. Baricitinib in hospitalised patients with COVID-19: A meta-analysis of randomised controlled trials. EClinicalMedicine 2022;49:101489. doi: 10.1016/j.eclinm.2022.101489 [published Online First: 20220603]

21. Recovery Collaborative Group. Baricitinib in patients admitted to hospital with COVID-19 (RECOVERY): a randomised, controlled, open-label, platform trial and updated meta-analysis. Lancet 2022;400(10349):359-68. doi: 10.1016/S0140-6736(22)01109-6

22. Marconi VC, Ramanan AV, de Bono S, et al. Efficacy and safety of baricitinib for the treatment of hospitalised adults with COVID-19 (COV-BARRIER): a randomised, double-blind, parallel-group, placebo-controlled phase 3 trial. Lancet Respir Med 2021;9(12):1407-18. doi: 10.1016/S2213-2600(21)00331-3 [published Online First: 20210901]

23. US Food and Drug Administration. Letter of authorization: EUA for baricitinib (Olumiant) for treatment of coronavirus disease 2019 (COVID-19). 2022. https://www.fda.gov/media/143822/download (accessed 23/12/2024).

24. Recovery Collaborative Group. Tocilizumab in patients admitted to hospital with COVID-19 (RECOVERY): a randomised, controlled, open-label, platform trial. Lancet 2021;397(10285):1637-45. doi: 10.1016/S0140-6736(21)00676-0

25. Nadeem A, Ahmad SF, Al-Harbi NO, et al. Inhibition of spleen tyrosine kinase signaling protects against acute lung injury through blockade of NADPH oxidase and IL-17A in neutrophils and gammadelta T cells respectively in mice. Int Immunopharmacol 2019;68:39-47. doi: 10.1016/j.intimp.2018.12.062 [published Online First: 20190102]

For peer review only - http://bmjopen.bmj.com/site/about/guidelines.xhtml

CONFIDENTIAL PRE-PUBLICATION MANUSCRIPT

26. Alimova M, Sidhom EH, Satyam A, et al. A High Content Screen for Mucin-1-Reducing Compounds Identifies Fostamatinib as a Candidate for Rapid Repurposing for Acute Lung Injury during the COVID-19 pandemic. bioRxiv 2020 doi: 10.1101/2020.06.30.180380 [published Online First: 20200630]

27. Strich JR, Tian X, Samour M, et al. Fostamatinib for the Treatment of Hospitalized Adults With Coronavirus Disease 2019: A Randomized Trial. Clin Infect Dis 2022;75(1):e491-e98. doi: 10.1093/cid/ciab732

28. Gotur DB, Malik A, Markovtsov V, et al. 88. Fostamatinib for the Treatment of Hospitalized Patients With COVID-19 Who Required Oxygen Supplementation: Results of a Phase 3 Trial. Open Forum Infectious Diseases 2023;10(Supplement_2) doi: 10.1093/ofid/ofad500.004

29. Hammersen J, Birndt S, Dohner K, et al. The JAK1/2 inhibitor ruxolitinib in patients with COVID-19 triggered hyperinflammation: the RuxCoFlam trial. Leukemia 2023;37(9):1879-86. doi: 10.1038/s41375-023-01979-w [published Online First: 20230728]

30. Velavan TP, Pallerla SR, Ruter J, et al. Host genetic factors determining COVID-19 susceptibility and severity. EBioMedicine 2021;72:103629. doi: 10.1016/j.ebiom.2021.103629 [published Online First: 20211013]

31. Malik A, Toth EN, Teng MS, et al. Distorted TCR repertoires define multisystem inflammatory syndrome in children. PLoS One 2022;17(10):e0274289. doi: 10.1371/journal.pone.0274289 [published Online First: 20221027]

32. Ruan Q, Yang K, Wang W, et al. Correction to: Clinical predictors of mortality due to COVID-19 based on an analysis of data of 150 patients from Wuhan, China. Intensive Care Med 2020;46(6):1294-97. doi: 10.1007/s00134-020-06028-z

For peer review only - http://bmjopen.bmj.com/site/about/guidelines.xhtml

CONFIDENTIAL PRE-PUBLICATION MANUSCRIPT

Acknowledgements

We would like to thank the patients (and their families) who took part in the trial, the clinical staff at participating hospitals and the research team at Imperial College Healthcare NHS Trust.

Data sharing

Anonymised data (excluding free text fields) can be made available upon reasonable request from the corresponding author (n.cooper@imperial.ac.uk) with appropriate human research ethics approvals and data transfer agreements in place.

For peer review only - http://bmjopen.bmj.com/site/about/guidelines.xhtml

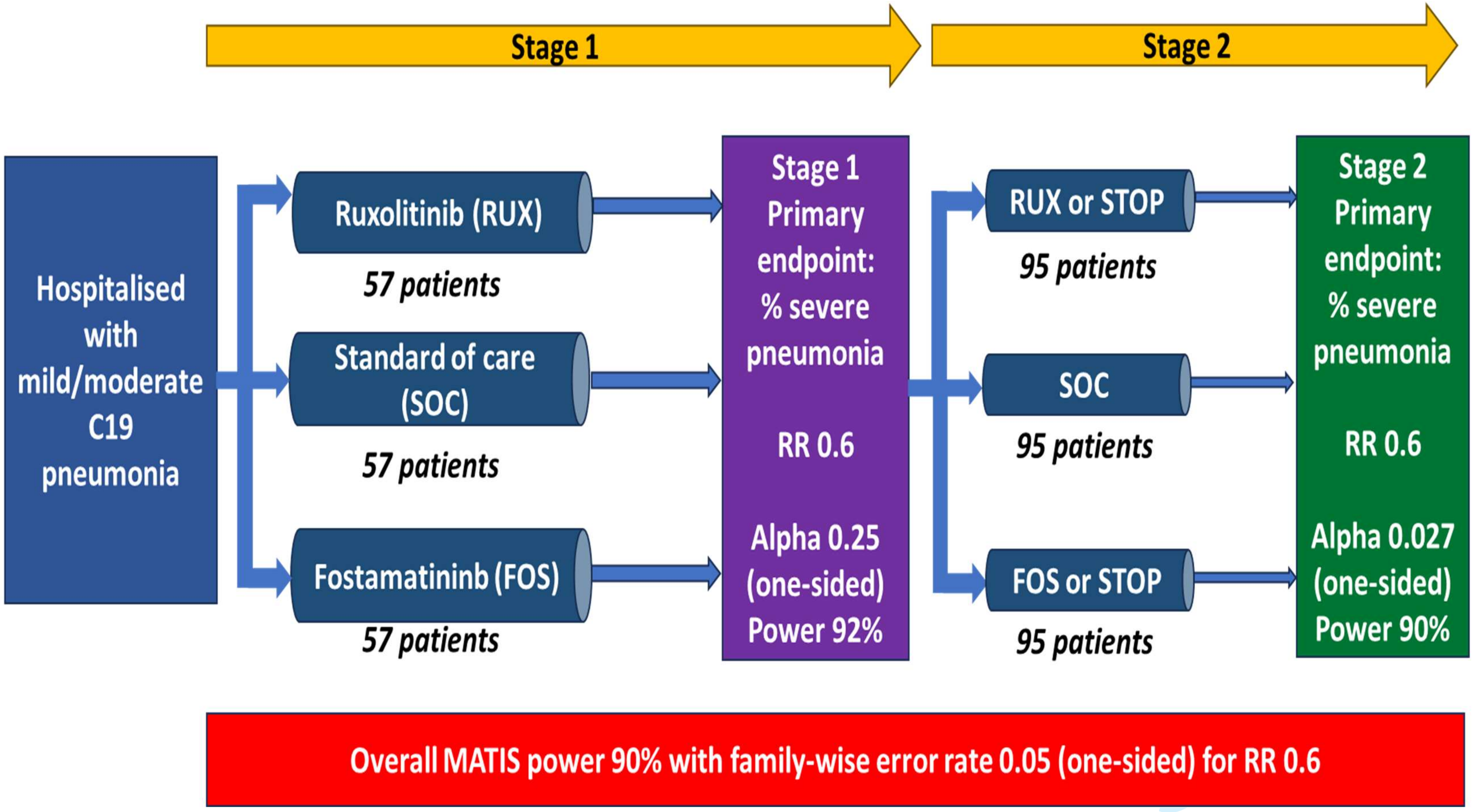

Figure 1: MATIS Trial Design

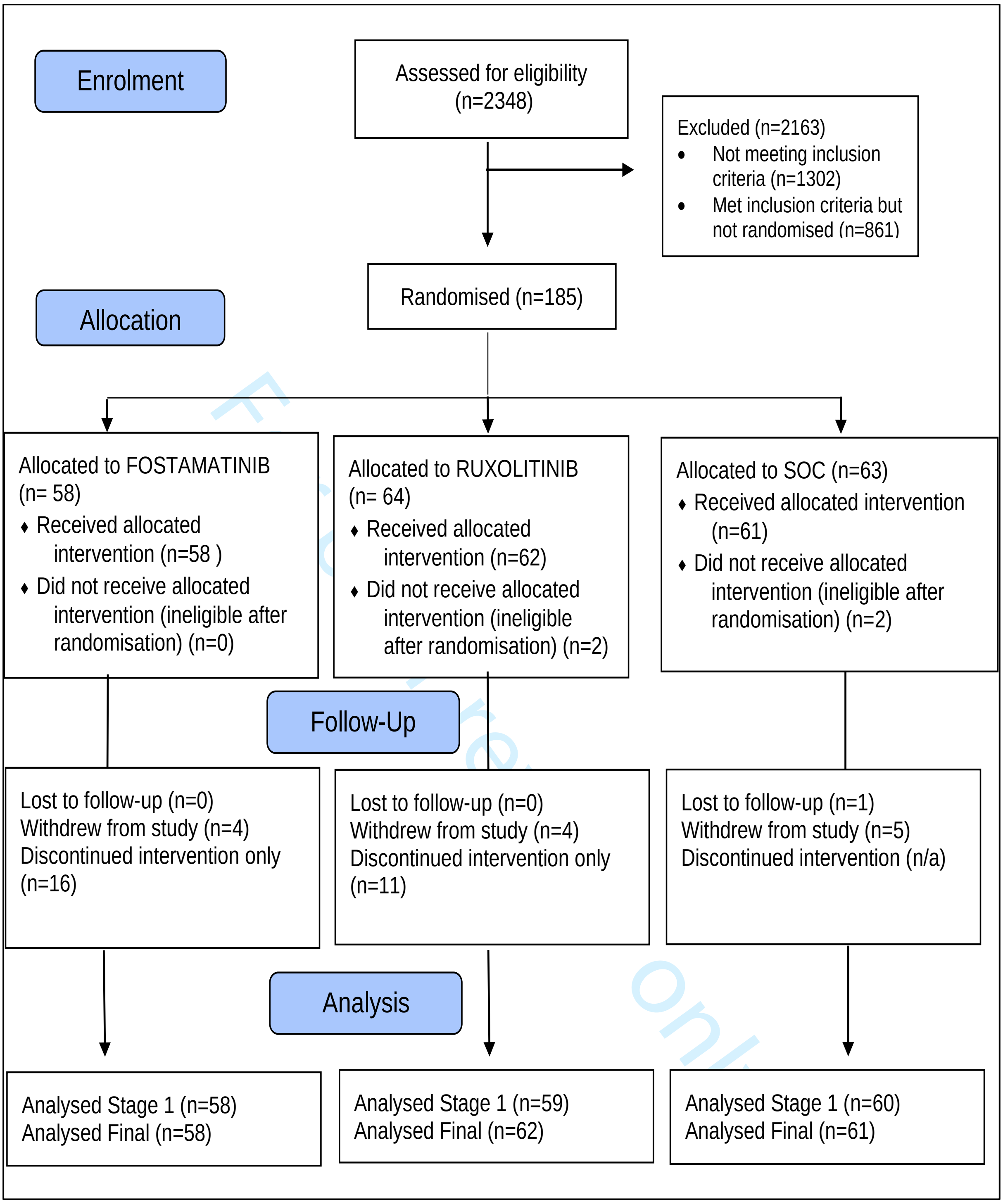

Figure 2: Participant flow

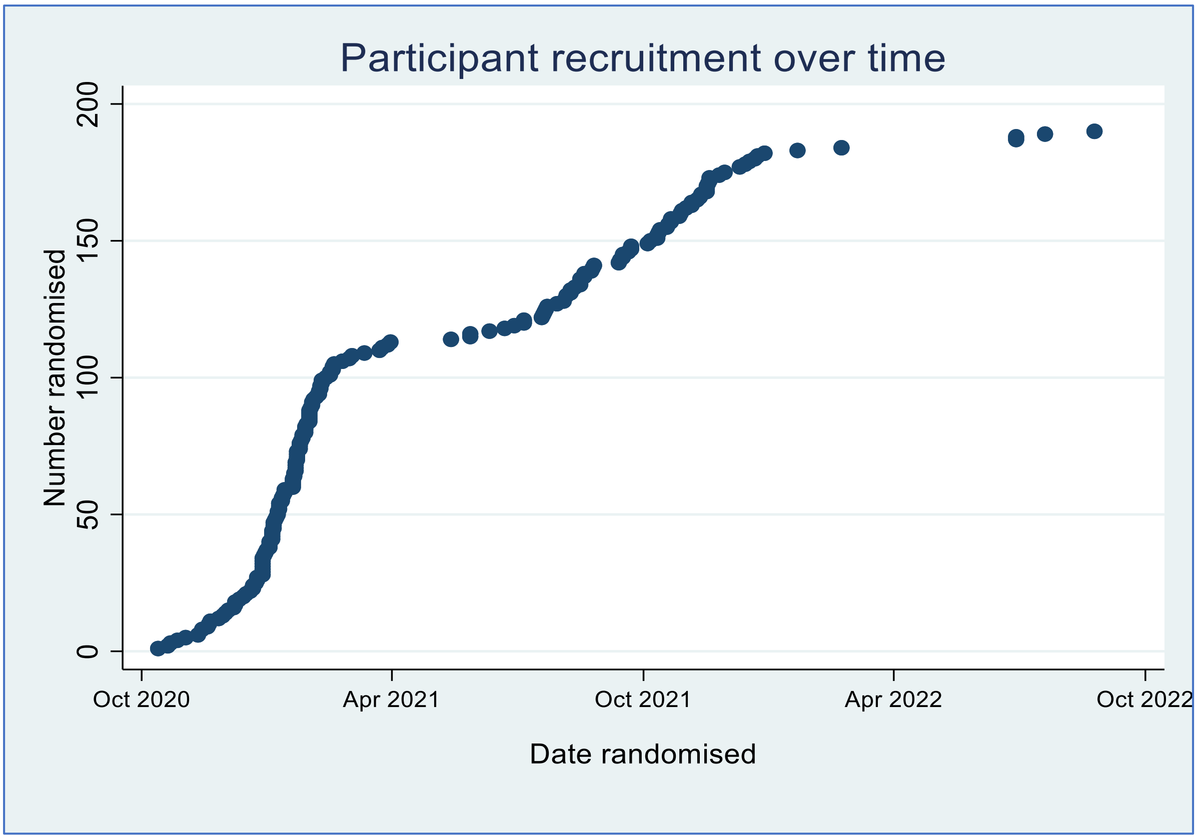

Figure 3: Recruitment over time

review only

BMJ Open

Multi-arm multi-stage randomised controlled trial of Inflammatory Signal Inhibitors (MATIS) for patients hospitalised with COVID-19 pneumonia during the UK pandemic.

Journal:	*BMJ Open*
Manuscript ID	bmjopen-2025-100583.R1
Article Type:	Original research
Date Submitted by the Author:	22-Oct-2025

Complete List of Authors:	Hazell, Lorna; Imperial College London, Imperial College Clinical Trials Unit<br>Pillay, Clio; Imperial College London, Department of Immunology and Inflammation; Imperial College Healthcare NHS Trust<br>Cornelius, Victoria; Imperial College London, Imperial College Clinical Trials Unit<br>Phillips, Rachel; Imperial College London, Imperial College Clinical Trials Unit<br>Charania, Asad; Imperial College London, Department of Immunology and Inflammation; Imperial College Healthcare NHS Trust<br>Wason, James; Newcastle University, Population Health Sciences Institute<br>Cherlin, Svetlana; Newcastle University, Population Health Sciences Institute<br>Savic, Sinisa; Leeds Teaching Hospitals NHS Trust, Immunology; University of Leeds,<br>Whittington, Ashley; Northwick Park Hospital, Infectious Diseases and Acute Medicine<br>Neelakantan, Pratap; Royal Berkshire NHS Foundation Trust, Haemato-Oncology<br>Collini, Paul; Sheffield Teaching Hospitals NHS Foundation Trust, Department of Infectious Diseases<br>Cook, Lucy; Imperial College London, Department of Immunology and Inflammation; Imperial College Healthcare NHS Trust<br>Willicome, Michelle; Imperial College London, Department of Immunology and Inflammation; Imperial College Healthcare NHS Trust<br>Milojkovic, Dragana; Imperial College London, Department of Immunology and Inflammation; Imperial College Healthcare NHS Trust<br>Kon, Onn Min; Imperial College Healthcare NHS Trust, Respiratory Medicine; Imperial College London, National Heart and Lung Institute<br>Youngstein, Taryn; Imperial College London, National Heart and Lung Institute; Imperial College Healthcare NHS Trust<br>Innes, Andrew; Imperial College London, Department of Immunology and Inflammation; Imperial College Healthcare NHS Trust<br>Thursz, Mark; Imperial College London, Department of Metabolism, Digestion and Reproduction; Imperial College Healthcare NHS Trust<br>Cooke, Graham; Imperial College London, Department of Infectious Diseases; Imperial College Healthcare NHS Trust<br>Vergis, Nikhil; Imperial College London, Department of Immunology and

	Inflammation; Imperial College Healthcare NHS Trust<br>Cooper, Nichola; Imperial College London, Department of Immunology and Inflammation; Imperial College Healthcare NHS Trust
**Primary Subject Heading**:	Immunology (including allergy)
Secondary Subject Heading:	Infectious diseases, Intensive care
Keywords:	Clinical Trial, COVID-19, Drug Therapy

SCHOLARONE™
Manuscripts

I, the Submitting Author has the right to grant and does grant on behalf of all authors of the Work (as defined in the below author licence), an exclusive licence and/or a non-exclusive licence for contributions from authors who are: i) UK Crown employees; ii) where BMJ has agreed a CC-BY licence shall apply, and/or iii) in accordance with the terms applicable for US Federal Government officers or employees acting as part of their official duties; on a worldwide, perpetual, irrevocable, royalty-free basis to BMJ Publishing Group Ltd ("BMJ") its licensees and where the relevant Journal is co-owned by BMJ to the co-owners of the Journal, to publish the Work in this journal and any other BMJ products and to exploit all rights, as set out in our licence.

The Submitting Author accepts and understands that any supply made under these terms is made by BMJ to the Submitting Author unless you are acting as an employee on behalf of your employer or a postgraduate student of an affiliated institution which is paying any applicable article publishing charge ("APC") for Open Access articles. Where the Submitting Author wishes to make the Work available on an Open Access basis (and intends to pay the relevant APC), the terms of reuse of such Open Access shall be governed by a Creative Commons licence – details of these licences and which Creative Commons licence will apply to this Work are set out in our licence referred to above.

Other than as permitted in any relevant BMJ Author's Self Archiving Policies, I confirm this Work has not been accepted for publication elsewhere, is not being considered for publication elsewhere and does not duplicate material already published. I confirm all authors consent to publication of this Work and authorise the granting of this licence.

For peer review only

CONFIDENTIAL PRE-PUBLICATION MANUSCRIPT

TITLE: Multi-arm multi-stage randomised controlled trial of Inflammatory Signal Inhibitors (MATIS) for patients hospitalised with COVID-19 pneumonia during the UK pandemic.

Authors:

Lorna Hazell, Trial Statistician (0000-0002-5962-0648) * [1]

Dr Clio Pillay, Trial Manager* [2,3]

Professor Victoria Cornelius, Professor in Medical Statistics and Trials Methodology (0000-0002-0080-1065) [1]

Dr Rachel Phillips, Associate Professor in Medical Statistics and Clinical Trials (0000-0002-3634-7845) [1]

Dr Asad Charania (0000-0001-8908-4535) [2,3]

Professor James M. S. Wason, Professor of Biostatistics (0000-0002-4691-126X) [4]

Dr Svetlana Cherlin, Senior Research Associate (0000-0001-9689-3699) [4]

Professor Sinisa Savic, Professor of Clinical Immunology (0000-0001-7910-0554) [5]

Dr Ashley Whittington, Consultant in Infectious Diseases and Acute Medicine [6]

Dr Pratap Neelakantan, Consultant Haematologist [7]

Dr Paul Collini, Senior Clinical Lecturer in Infectious Diseases (0000-0001-6696-6826) [8]

Dr Lucy Cook, Consultant Haematologist (0000-0002-8234-5090) [2,3]

Dr Michelle Willicome, Clinical Reader in Transplantation [2,3]

Dr Dragana Milojkovic, Consultant Haematologist [2,3]

Professor Onn Min Kon, Respiratory Consultant (0000-0003-2647-4688) [3,9]

Dr Taryn Youngstein, Consultant Rheumatologist (0000-0002-8297-556X) [3,9]

Dr Andrew J. Innes, Honorary Clinical Senior Lecturer (0000-0003-0918-8882) [2,3]

For peer review only - http://bmjopen.bmj.com/site/about/guidelines.xhtml

CONFIDENTIAL PRE-PUBLICATION MANUSCRIPT

Professor Mark Thurz, Director of Academic Health Science Centre (0000-0002-8218-192X) [3,10]

Professor Graham S. Cooke, Deputy Dean of the Faculty of Medicine (0000-0001-6475-5056) [3,11]

Dr Nikhil Vergis, Honorary Clinical Senior Lecturer (0000-0002-4157-6757) [$ 2,3]

Professor Nichola Cooper, Professor of Immune Haematology (0000-0002-9845-8292) [$ 2,3]

Corresponding author:

Nichola Cooper, n.cooper@imperial.ac.uk

[*] Both authors contributed equally to this work.

[$] Both authors contributed equally to this work.

For peer review only

CONFIDENTIAL PRE-PUBLICATION MANUSCRIPT

Author Affiliations

1. Imperial Clinical Trials Unit, Imperial College London, UK
2. Department of Immunology and Inflammation, Imperial College London, UK
3. Imperial College NHS Healthcare Trust, London, UK.
4. Population Health Sciences Institute, Newcastle University, Newcastle upon Tyne, UK
5. Clinical Immunology and Allergy, Leeds Teaching Hospitals NHS Trust, Leeds, UK.
6. Infectious Diseases and Acute Medicine, Northwick Park Hospital, London, UK
7. Department of Haematology, Royal Berkshire Hospital, Reading, UK
8. Department of Infectious Diseases, Sheffield Teaching Hospitals NHS Foundation Trust, Sheffield, UK
9. National Heart and Lung Institute, Imperial College London, UK
10. Department of Metabolism, Digestion and Reproduction, Imperial College London, UK
11. Department of Infectious Diseases, Imperial College London, UK

For peer review only

CONFIDENTIAL PRE-PUBLICATION MANUSCRIPT

Copyright/license for publication

The Corresponding Author has the right to grant on behalf of all authors and does grant on behalf of all authors, an exclusive licence (or non-exclusive for government employees) on a worldwide basis to the BMJ Publishing Group Ltd to permit this article (if accepted) to be published in BMJ editions and any other BMJPGL products and sublicences such use and exploit all subsidiary rights, as set out in our licence.

Competing interest statement

All authors have completed the Unified Competing Interest form (available on request from the corresponding author). LH, CP, RP, AW, PN, PC, LC, MT and NV declare no support from any organisation for the submitted work; no financial relationships with any organisations that might have an interest in the submitted work in the previous three years and no other relationships or activities that could appear to have influenced the submitted work. VC is a co-investigator leading statistical design, methods, and analysis for the MATIS trial, funded by Novartis and Rigel, is a committee member for the NIHR Academy Doctoral board, a DMEC member for the NIHR HTA-funded BAY Project. Behavioural Activation for Young people with depression in specialist child and adolescent mental health services and is chair of the DMC for NIHR HTA-funded RADAR: Research into Antipsychotic Discontinuation and Reductions. AC has been an employee of Roche since 2022. DM has received honorary payments from Novartis, Incyte and Ascentage Pharma, has participated on an advisory board for Ascentage Pharma and has received investigator-led institutional research funding from Incyte. MW has received honorary payments from AstraZeneca, has participated on an advisory board or data monitoring committee for

For peer review only - http://bmjopen.bmj.com/site/about/guidelines.xhtml

Hansa Pharmaceuticals, received equipment, materials, drugs, medical writing, gifts or other services from Moderna and has had a leadership or fiduciary role in other board, society, committee or advocacy group, paid or unpaid for Hansa Pharmaceuticals. OMK has participated on an advisory board or data monitoring committee for the NIHR DILI study and has had team members funded by grants from Cepheid. GC has participated in an end point review committee for Sanofi and is a board member for the MHRA. SS holds the Chair of British Society for Immunology-Clinical immunology professional network. JW and SC have had team members funded by grants from Novartis and Rigel. TY is recipient of a grant from UKRI MRC BHF Cardio-IMID Partnership, has received consulting fees for the Ad Board Abbvie 2024 and is Treasurer for the UK and Ireland Vasculitis and Rare Disease Group. AJI has received consulting fees, honoraria for speakers' bureaus, international travel support and/or personal fees from Novartis and Incyte. NC has received research grants from Novartis and Rigel for conducting MATIS, and honoraria for consultancy from Novartis, Sobi, Sanofi, Amgen, Argenyx.

Contributorship and guarantor information

Contributors: NC conceived the research idea and led the trial. NC attained funding for the trial. NC, CP, SS, AC, AW, PN, PC, LC, MW, DM, OMK, TY, AJI, MT, GC and NV were principal investigators or investigators and contributed to the inception, trial management, data collection and/or completion of the trial. RP, JW, VC and NC were responsible for the study design and statistical analysis plan. LH, SC and RP

For peer review only - http://bmjopen.bmj.com/site/about/guidelines.xhtml

CONFIDENTIAL PRE-PUBLICATION MANUSCRIPT

conducted the statistical analysis. LH wrote the first draft of the manuscript with input from RP, VC, CP and NC. All authors approved of the submitted version.

The corresponding author (NC) attests that all listed authors meet authorship criteria and that no others meeting the criteria have been omitted. NC, as guarantor, accepts full responsibility for the work and/or the conduct of the study, had access to the data, and controlled the decision to publish.

Transparency declaration

NC affirms that the manuscript is an honest, accurate, and transparent account of the study being reported; that no important aspects of the study have been omitted; and that any discrepancies from the study as originally planned (and registered) have been explained.

Role of the funding source

The MATIS trial was sponsored by Imperial College London and was jointly funded by NIHR Imperial Biomedical Research Centre, Novartis, the manufacturer of ruxolitinib (Jakavi®) and Rigel, the manufacturer of fostamatinib (Tavlesse®).

The sponsor and funders had no control over the study design; data collection, analysis, or interpretation of data; in the writing of the report; or in the decision to

For peer review only - http://bmjopen.bmj.com/site/about/guidelines.xhtml

submit the article for publication. All authors confirm independence from the funders, had full access to all of the data (including statistical reports and tables) and take responsibility for the integrity of the data and the accuracy of the data analysis.

Trial registration details

The MATIS trial was registered prospectively in ClinicalTrials.gov (NCT04581954 and EUDRA-CT (2020-001750-22) before enrolling the first participant.

Abstract

Objectives: To determine the safety and efficacy of ruxolitinib and fostamatinib compared to standard of care (SOC) in patients requiring hospital admission for the treatment of COVID-19 pneumonia.

Design: Adaptive multi-arm, multi-stage, randomised, open label trial (3-arm, 2-stage)

Setting: Five hospitals in England between October 2020 and September 2022.

Participants: Hospitalised patients (≥18 years) with COVID-19 pneumonia defined by a modified World Health Organisation (WHO) COVID-19 severity grade of 3 or 4.

For peer review only

Interventions: Participants were randomly assigned 1:1:1 to receive ruxolitinib (10mg bd for 7 days then 5mg bd for 7 days), fostamatinib (150mg bd for 7 days then 100mg bd for 7 days) or SOC.

Main outcome measures: Primary outcome was development of severe COVID-19 pneumonia (modified WHO severity grade ≥5) within 14 days of randomisation. Secondary outcomes included mortality, invasive and non-invasive ventilation, venous thromboembolism, duration of hospital stay, readmissions, inflammatory markers and serious adverse events (SAE).

Results: At Stage 1, 181 patients were randomised, with 4 assessed as ineligible post-randomisation. Fostamatinib was stopped early for futility with 16 participants (27.6%, N=58) developing severe COVID-19 pneumonia compared to 15 (25.0%, N=60) in the SOC arm (adjusted odds ratio (aOR) compared to SOC: 1.12; 95% confidence interval (CI): 0.49 to 2.58; p=0.608). Ruxolitinib progressed to Stage 2 but the trial was stopped early due to slow recruitment. At the final analysis, 10 participants (16.1%, N=62) developed severe COVID-19 pneumonia in the ruxolitinib arm compared to 15 (24.6%, N=61) in the SOC arm (aOR: 0.63; 95% CI: 0.25 to 1.57; p=0.161). Four (7.4%) participants in the fostamatinib arm, none in the ruxolitinib arm and three (5.5%) in the SOC arm died within 14 days of randomisation. Infections were the most frequently reported serious adverse event and were numerically higher in the fostamatinib (10, 17.2%) and ruxolitinib (10, 16.1%) arms compared to SOC (7, 11.5%). Two unexpected serious adverse reactions occurred in the ruxolitinib arm only.

For peer review only - http://bmjopen.bmj.com/site/about/guidelines.xhtml

Conclusions: We found no evidence that fostamatinib was superior to SOC for the treatment of COVID-19 pneumonia in patients requiring hospital admission. Progression to severe COVID-19 pneumonia was less frequent in the ruxolitinib arm, but due to early stopping, the trial was underpowered to confirm ruxolitinib's benefit in this population. Further study is needed.

Trial registration: NCT04581954; EUDRA-CT: 2020-001750-22

Strength and Limitations of the Study

- Participants in the MATIS trial were broadly representative of an urban population requiring hospital admission for the treatment of COVID-19 pneumonia in the UK.
- We used an efficient multi-arm, multi-stage trial design to focus research efforts on more promising treatments in the pandemic setting.
- We were able to determine that fostamatinib was unlikely to be efficacious in this patient population at an early stage of the trial.
- The trial was, however, subsequently stopped early due to slowing recruitment as the pandemic receded and mass vaccination was introduced.
- We were therefore unable to confirm the efficacy of ruxolitinib in this patient setting.

For peer review only - http://bmjopen.bmj.com/site/about/guidelines.xhtml

For peer review only

Introduction

Severe COVID-19 pneumonia is characterised by respiratory and multi-organ failure in the context of marked systemic inflammation and increased thrombotic risks. Elevations in circulating inflammatory molecules are associated with poor prognosis and in a subset of patients, drive acute lung injury which, in the early stages of the 2020 pandemic, resulted in high rates of catastrophic multi-organ failure and death.[1]

Therapeutic interventions targeting inflammatory signalling might reduce the severity of the inflammatory response phase and associated lung damage thereby averting respiratory failure and the need for mechanical ventilation. Data from China and Italy during the pandemic suggested that immediate resolution of symptoms could be achieved using anti-interleukin 6 (IL6) therapy and Janus kinase (JAK) inhibitors in patients with severe disease.[2 3] The spleen tyrosine kinase (SYK) pathway was also considered a potential target; it is activated by several receptors, including C-type lectin (CLEC) or C-type lectin-like receptors (CLR), and Fc receptors.[4] Inhibition of the SYK pathway has been shown to inhibit platelet aggregation in heparin-Induced thrombocytopenia models,[5] a disease with many similar features to COVID-19.

We hypothesised that there may be an early window of opportunity to treat the COVID-19 hyperinflammatory syndrome before acute lung injury leads to organ failure. We designed a randomised controlled, multi-arm multi-stage trial in patients hospitalised with COVID-19 pneumonia of grade 3 or 4 on the modified World Health Organisation (WHO) COVID-19 severity scale to evaluate the effect of early intervention with two inflammatory signal inhibitors which are already licensed for

For peer review only - http://bmjopen.bmj.com/site/about/guidelines.xhtml

use in other clinical indications: ruxolitinib (JAK inhibitor)[6] and fostamatinib (small molecule SYK inhibitor).[7]

Methods

Trial design and participants

The Multi-Arm Trial of Inflammatory Signal Inhibitors for COVID-19 (MATIS) was an open-label multi-arm, multi-stage randomised (1:1:1) controlled trial (RCT) of ruxolitinib (RUX) and fostamatinib (FOS) for the treatment of COVID-19 pneumonia compared to routine standard of care (SOC) in patients requiring hospital admission.

Inclusion criteria:

Eligible participants were hospitalised patients in the UK aged ≥18 years with clinically suspected or laboratory confirmed SARS-Cov2 infection, radiological change consistent with COVID-19 disease, a C-reactive protein (CRP) level ≥30mg/L at any time point and COVID-19 pneumonia defined by a severity grade 3 or 4 on the modified WHO severity scale (Table 1).[8] Non-English speakers were eligible to join the study; hospital translation services were provided where appropriate. Female participants of child-bearing potential were required to agree to abstain from sexual intercourse or use effective contraception for 42 days; after the last dose of study medication male participants were required to abstain from sperm donation for 42 days after the last dose of study medication.

Table 1. Modified WHO COVID-19 Severity Scale, from the WHO R&D Blueprint

Patient state	Descriptor	Grade
Uninfected	No clinical or virological evidence of infection	0

For peer review only - http://bmjopen.bmj.com/site/about/guidelines.xhtml

Ambulatory	No limitation of activities	1
	Limitation of activities	2
Hospitalised mild disease	Hospitalised, no oxygen therapy	3
	Oxygen by mask or nasal prongs	4
Hospitalised severe disease	SpO_2< 90% on FiO_2 ≥60% by face mask	5
	Non-invasive ventilation, CPAP or high-flow oxygen	6
	Intubation and mechanical ventilation	7
	Ventilation + additional organ support (vasopressors, RRT, ECMO)	8
Dead	Death	9

Published in the COVID Therapeutic Trial Synopsis, February 18th 2020[8]

CPAP: continuous positive airways pressure; SpO_2: peripheral arterial blood oxygen; FiO_2: fraction of inspired oxygen; RRT: renal replacement therapy; ECMO: extracorporeal membrane oxygenation

Exclusion criteria:

Patients with severity below Grade 3 on the modified WHO COVID-19 Ordinal Scale (i.e., non-hospitalised patients) were not eligible to participate in the MATIS trial. Patients were also ineligible if they had ≥Grade 5 severity pneumonia or had oxygen saturation levels < 90% on 60% inspired oxygen at baseline or if, in the opinion of the investigator, progression to death was inevitable within the next 24 hours, irrespective of the provision of therapy or if they required either invasive or non-invasive ventilation including continuous positive airway pressure (CPAP) or high flow nasal oxygen at any point after hospital admission and before baseline. Patients normally on non-invasive ventilation such as continuous positive airway pressure CPAP at home were eligible to take part. Further exclusion criteria were any medical condition/history or concomitant medication that would compromise participants' safety or put the participant at significant risk or compliance with study procedures or would compromise the scientific integrity of the study, any known severe allergic reactions to the investigational agents, use of drugs within the preceding 14 days

For peer review only - http://bmjopen.bmj.com/site/about/guidelines.xhtml

known to interact with any study treatment, Child Pugh B or C grade hepatic dysfunction, pregnancy and breast feeding.

The trial design (Figure 1) is further described in the trial protocol.[9] A planned formal interim analysis (Stage 1) was conducted on 10th March 2022 after 177 eligible participants had been randomised and completed 14 days of follow-up. A pre-specified threshold of one-sided p-value ≤0.25 was required to progress treatment with RUX and/or FOS to Stage 2.

Randomisation and blinding

Participants were randomly allocated to RUX, FOS or SOC using a central web-based randomisation service that used randomisation sequences with random block sizes stratified by age (<65 vs ≥65) and study site. Staff treating participants, participant-facing research staff and participants were unblind to study treatment. IDMC reports prior to the Stage 1 analysis were prepared by the trial statistician who was aware only of treatment groups labelled as Group A, B, and C. Stage 1 analysis was performed by an independent, unblind statistician at Newcastle University. The final analyses were undertaken by an unblind trial statistician at Imperial College Clinical Trials Unit. All analyses were undertaken after the Statistical Analysis Plan was signed off.

Interventions

The planned duration of treatment was 14 days. Participants in the RUX arm were prescribed 10mg twice daily for 7 days reducing to 5mg twice daily for a further 7 days. Participants in the FOS arm were prescribed 150mg twice daily for 7 days reducing to 100mg twice daily for a further 7 days. Treatment was given by mouth unless the participant was unable to take oral medicines, in which case treatments

For peer review only - http://bmjopen.bmj.com/site/about/guidelines.xhtml

were administered via a nasogastric tube. Study treatment was given in combination with SOC. Due to the evolving clinical landscape for the management of COVID-19, the SOC was not predefined and varied during the course of the trial. Co-enrolment to other trials, additional treatment and rescue therapy was permitted at the discretion of the treating clinicians.

Follow up

Participants were followed up at 1, 7, 14 and 28 days after randomisation. Those discharged from hospital before Day 14 completed trial medication at home and were followed up by telephone or using patient health records, where available, up to Day 28.

Outcomes

All comparisons were between RUX or FOS versus SOC. The primary outcome was defined as the proportion with severe COVID-19 pneumonia within 14 days of randomisation, where severe COVID-19 pneumonia was defined by a modified WHO COVID-19 Ordinal Grade ≥ 5 (on the 9-point scale, Table 1).[8] Secondary outcomes, examined at Day 14 and Day 28 included change in severity on the modified WHO COVID-19 Ordinal Scale (Grade 3 or 4 to 5,6,7,8,9), individual components of the primary outcome (mortality, non-invasive ventilation including continuous positive airway pressure (CPAP) and high flow nasal oxygen, invasive ventilation), organ support, venous thromboembolism, length of hospital stay, readmission within 28 days, inflammatory markers (CRP, lactose dehydrogenase (LDH), ferritin, D-dimer), serum creatinine and serious adverse events (SAEs). Non-serious adverse events were not collected.

For peer review only - http://bmjopen.bmj.com/site/about/guidelines.xhtml

For peer review only

Sample size

A sample size of 171 (57 per arm) participants at Stage 1 and an additional maximum of 285 (95 per arm) was selected to provide power of 90% (minimum marginal power) with a maximum 5% chance of an intervention arm being recommended when it provides no improvement over control (5% one-sided family-wise error rate), inflated by 5% for missing outcome data (full details are described in the trial protocol).[9] This sample size calculation assumed a 50% rate of severe pneumonia in the standard of care arm and a reduction of this risk in an experimental arm to 30% (relative risk, RR, 0.6).

Statistical analysis

Participant flow through the trial was described using a Consolidated Standards of Reporting Trials (CONSORT) flow chart.[10] A scatter plot was used to show the trajectory of participant recruitment over time. Simple descriptive statistics were used to summarise baseline participant characteristics, co-enrolment to other trials and SOC medications received at baseline and post-randomisation.

The modified intention-to-treat (ITT) population included all eligible randomised participants analysed in the treatment arm to which they were allocated to regardless of treatment subsequently received. Participants found to have been randomised in error were excluded from the modified ITT population. Participants were assumed to have received 14 days of treatment unless there was evidence for stopping earlier.

For peer review only - http://bmjopen.bmj.com/site/about/guidelines.xhtml

For the primary outcome, we used a generalized linear model with a binomial distribution and logit link function to compare the odds of developing severe COVID-19 pneumonia between RUX or FOS and SOC. At Stage 1, two separate models were used to compare the effects of FOS vs SOC and RUX vs SOC. These models were adjusted for using a propensity score derived from a logistic regression model that included baseline modified WHO COVID-19 severity grade (3 or 4), age (<65 or ≥65), receipt of steroids at baseline and receipt of interleukin-6 (IL6) inhibitors at baseline.

For the final analysis, we included all three trial arms in a single model. We planned to adjust our final analysis model for site, age category (<65 or ≥65), baseline modified WHO COVID-19 severity grade (3 or 4) and receipt of treatments assumed to be effective prior to randomisation based on current knowledge at that time of the MATIS trial. These effective treatments included dexamethasone, IL-6 inhibitors, selected antivirals and COVID vaccination. As the efficacy of remdesivir was unconfirmed at the time of the trial, Paxlovid® and/or molnupiravir were the only antivirals specified *a-priori*, however, no participants received these agents. As a large proportion of participants had received dexamethasone in each arm, prior steroid use was not included in our final analysis model to avoid instability due to zero cell counts in some strata. Similarly, as there were several sites with very few randomised participants, we excluded study site from the primary analysis model. Thus, our final model adjusted for prior use of IL-6 inhibitors, age category, modified WHO COVID-19 severity grade and receipt of COVID-19 vaccine. These were included as separate covariates in the final model. Multiple imputation was used to account for any missing data such that all eligible randomised participants were

For peer review only - http://bmjopen.bmj.com/site/about/guidelines.xhtml

included in both the Stage 1 and final analyses (See Supplementary Appendix 5).[11] Treatment effects were reported as odds ratios with corresponding 95% confidence intervals (CI) and one-sided p-values.

The original two-stage design specified that significance would be judged at the 0.027 level (i.e. a two-sided p-value <0.054 was to be used to indicate a significant treatment effect). However, as the planned sample size was not reached, the p-values presented should not be interpreted with reference to this threshold as the trial was significantly underpowered. Interpretation should be focused on the point estimate and 95% CI as a way to assess the evidence of a treatment effect and associated uncertainty, however, this interpretation should be regarded as hypothesis-generating only.

Analysis of secondary outcomes was performed for the final analysis only. Here, the primary analysis model was repeated to compare the odds of developing severe pneumonia by Day 28. This model was also run separately for each individual component of the primary outcome and the maximum COVID-19 severity grade reached by Day 14 and Day 28. In addition, COVID-19 severity grade was modelled as an ordinal variable using a mixed ordinal logistic regression model to compare the odds of progression to more severe disease at Day 14. Mixed linear regression models were used to compare changes in inflammatory markers and serum creatinine up to Day 28 and a Cox proportional hazards model was used to compare the rates of hospital discharge between trial arms. The frequency of venous thromboembolism, readmission within 28 days and SAEs were summarised

For peer review only - http://bmjopen.bmj.com/site/about/guidelines.xhtml

descriptively. Dot plots were used to visually display between group differences in SAE frequencies (as risk ratios) grouping events at system organ class level.

Prespecified sensitivity analyses were undertaken to examine alternative approaches to handling missing data firstly, assuming that all participants with missing outcome had poor outcome e.g., all missing assumed severity grade >=5, and secondly assuming that all participants with missing outcome had good outcome e.g., all missing assumed severity grade <5. A planned sensitivity analysis of the primary outcome adjusting for receipt of effective COVID-19 treatments through co-enrolment to the active arm of another randomised controlled was not performed since such treatments were not found to have been prescribed to trial participants as part of co-enrolment to another trial. Another sensitivity analysis to explore both the direct effect of treatment on the outcome and any indirect effect of receiving known effective treatments as rescue medications was not conducted as all of the participants who received these medications post-randomisation to MATIS received the medication on or after the index date for the primary outcome.

We performed two prespecified exploratory analyses to assess the impact of adherence to treatment on the primary outcome. Firstly, we conducted a naïve per-protocol analysis in participants who had a minimum of 90% of the planned treatment (at least 13 days of active treatment). In the SOC arm, patients were deemed to be 100% adherent to SOC by definition. Patients who died were considered adherent if they had not discontinued trial treatment at the time of death. Secondly, we calculated the treatment effect (as a risk difference) among compliers using a complier adjusted case (CACE) analysis with a two-stage residual inclusion estimator (2SRI) approach[12] and randomisation group as the instrumental variable.

For peer review only - http://bmjopen.bmj.com/site/about/guidelines.xhtml

CONFIDENTIAL PRE-PUBLICATION MANUSCRIPT

Estimates from the CACE analysis were generated from two separate models for each treatment comparison while the original ITT analysis was one model.

Additional exploratory analyses were performed to assess the impact of the time of enrolment to the trial. A categorical variable was created by splitting the recruitment period into quartiles such that participants were grouped into approximately four equal sized groups based on time of enrolment into the MATIS study. This variable was included as a covariate in the primary analysis model as a treatment group-by-time interaction term to assess the impact of time of recruitment on the treatment effect estimate. A crude odds ratio was calculated for each quartile. This analysis was performed using the unimputed dataset since the multiple imputation model could not produce the required estimates due to inconsistency in the omitted variables across the different imputed datasets. Subgroup analysis of the primary outcome by age, sex, BMI, cardiovascular disease, chronic lung disease, end-stage-renal failure, liver cirrhosis, diabetes, baseline CRP and D-dimer, immunocompromisation, smoking status and number of days since onset of COVID-19 at randomisation were also performed.

Stage 1 analysis was performed in R (https://www.R-project.org/). All other analysis was undertaken using Stata/IC version 16.1, (StataCorp, College Station, TX, USA). The Statistical Analysis Plan is provided in Supplementary Appendix 1.

Patient and Public Involvement

For peer review only - http://bmjopen.bmj.com/site/about/guidelines.xhtml

Information on MATIS trial design was included on clinicaltrial.gov. A twitter page was set up to explain the trial to a wider audience, and the protocol was published in Trials (2021).[9]

Ethics

The Surrey Research Ethics Committee (REC) and Health Regulator Authority (HRA) reviewed and granted approval for this trial (IRAS ID 282552). The study was conducted in accordance with the recommendations for physicians involved in research on human subjects adopted by the 18th World Medical Assembly, Helsinki 1964 and later revisions.[13] Informed consent was obtained for each participant either by the participant themselves or from a relative or an independent treating clinician acting as their legally designated personal representative in the event that the participant lacked capacity to provide consent.

For peer review only - http://bmjopen.bmj.com/site/about/guidelines.xhtml

Results

Participant flow and baseline characteristics

Between October 2020 and September 2022,185 patients from five UK hospitals in England were enrolled (Figure 2). The majority of participants were recruited from one London teaching hospital site (n=170) with a smaller proportion from other UK city hospitals (n=15). Recruitment followed the peaks and troughs of the COVID-19 pandemic waves observed in the UK during the trial period (Figure 3). This period predominantly covered the Alpha and Delta waves in the UK.[14] Although the Omicron variant was detected from around December 2021 only 12 participants were recruited to MATIS after that date.

During the 28-day follow-up, 13 participants withdrew consent and one was lost to follow-up. Four patients found to be ineligible post-randomisation were excluded from the analyses. A slightly higher proportion of participants completed follow up but discontinued study medication early in the FOS arm (13, 22.4%) than in the RUX arm (11, 17.2%). Thirty-eight (66.7%) participants in the FOS arm and 46 (71.9%) in the RUX arm were assumed to have completed the 14-day treatment course.

A higher proportion of participants in the RUX arm compared to the SOC arm were female (35.9% vs. 30.2%), were of white ethnicity (48.4% vs. 42.9%), had a modified WHO COVID-19 severity grade of 3 at baseline (21.9% vs. 14.3%) or had received a COVID-19 vaccination (25.0% vs. 17.5%) (Table 2). In contrast, these characteristics

For peer review only - http://bmjopen.bmj.com/site/about/guidelines.xhtml

were less prevalent in the FOS arm compared to SOC, with the exception of the baseline modified WHO severity grade of 3 (17.2% in FOS vs. 13.3% in SOC). The percentage of participants with a baseline modified WHO COVID-19 severity grade of 4 decreased over the trial recruitment period from 84.8% in the Alpha wave to 75% for those recruited in the Omicron wave, while the percentage of participants who had received the COVID-19 vaccination increased from 8% to 50% (Supplementary Table S1).

Table 2: Baseline characteristics by treatment arm

Patient characteristic, n (%) unless otherwise specified	Fostamatinib (N=58)	Ruxolitinib (N=64)	Standard of care (N=63)
Age in years			
Mean (SD)	58.9 (15.0)	60.5 (15.8)	59.1 (16.3)
Sex			
Male	43 (74.1)	41 (64.1)	44 (69.8)
Female	15 (25.9)	23 (35.9)	19 (30.2)
Ethnicity			
White	21 (36.2)	31 (48.4)	27 (42.9)
Mixed or multiple ethnic groups	1 (1.7)	0 (0.0)	1 (1.6)
Asian or Asian British	7 (12.1)	11 (17.2)	8 (12.7)
Black, Black British, Caribbean or African	5 (8.6)	7 (10.9)	7 (11.1)
Other ethnic group	24 (41.4)	15 (23.4)	20 (31.7)
BMI in kg/m2			
N (N missing)	55 (3)	57 (7)	60 (3)
Mean (SD)	30.1 (6.5)	30.6 (7.1)	29.9 (6.9)
Modified WHO COVID-19 severity			
Grade 3	10 (17.2)	14 (21.9)	9 (14.3)
Grade 4	48 (82.8)	50 (78.1)	54 (85.7)
Time from onset of symptoms			
N (N missing)	57 (1)	51 (13)	57 (6)
Mean (SD)	10 (4)	9 (4)	10 (4)
Chronic lung disease			
N (N missing)	58 (0)	62 (2)	63 (0)
Yes	7 (12.1)	12 (19.4)	11 (17.5)
Diabetes			
N (N missing)	58 (0)	62 (2)	63 (0)
Yes	15 (25.9)	19 (30.6)	20 (31.7)
Hypertension			
N (N missing)	58 (0)	62 (2)	63 (0)
Yes	25 (43.1)	28 (45.2)	28 (44.4)

For peer review only - http://bmjopen.bmj.com/site/about/guidelines.xhtml

Patient characteristic, n (%) unless otherwise specified	Fostamatinib (N=58)	Ruxolitinib (N=64)	Standard of care (N=63)
Ischaemic heart disease			
N (N missing)	58 (0)	62 (2)	63 (0)
Yes	11 (19.0)	10 (16.1)	10 (15.9)
Heart failure			
N (N missing)	58 (0)	62 (2)	63 (0)
Yes	2 (3.4)	3 (4.8)	2 (3.2)
Immunocompromised			
N (N missing)	58 (0)	62 (2)	63 (0)
Yes	1 (1.7)	5 (8.1)	2 (3.2)
End-stage renal failure			
N (N missing)	58 (0)	62 (2)	63 (0)
Yes	4 (6.9)	6 (9.7)	3 (4.8)
Liver cirrhosis			
N (N missing)	58 (0)	62 (2)	63 (0)
Yes	0 (0.0)	0 (0.0)	0 (0.0)
Current smoker			
N (N missing)	58 (0)	62 (2)	62 (1)
Yes	0 (0.0)	2 (3.2)	3 (4.8)
Prior Covid-19 Vaccination			
N (N missing)	58 (0)	64 (0)	63 (0)
Yes	8 (13.8)	16 (25.0)	11 (17.5)
Serum creatinine (μmol/L)			
N (N missing)	57 (1)	62 (2)	63 (0)
Mean (SD)	133 (203)	119 (145)	119 (172)
C- reactive protein (mg/L)			
N (N missing)	57 (1)	62 (2)	63 (0)
Mean (SD)	94.1 (61.5)	120.9 (78.4)	105.8 (69.5)
Lactate dehydrogenase (IU/L)			
N (N missing)	36 (22)	37 (27)	36 (27)
Mean (SD)	419 (208)	488 (309)	439.(136)
Ferritin (μg/L)			
N (N missing)	52 (6)	52 (12)	47 (16)
Mean (SD)	1471 (1459)	1671 (3069)	1254 (778)
D-dimer (ng/ml)			
N (N missing)	55 (3)	53 (11)	53 (10)
Mean (SD)	1601 (2749)	1732 (2614)	1341 (2781)

The majority of participants in all three trial arms were receiving anticoagulant therapy at baseline (Table 3), most were receiving an immuno-modulating agent, specifically dexamethasone (Supplementary Table S2). Just under half of participants had received an antiviral agent (Table 3), of whom 86 (98.9%) received remdesivir. These standard of care treatments were initiated a median of one day

For peer review only - http://bmjopen.bmj.com/site/about/guidelines.xhtml

before or on the same day as randomisation in all three trial arms and continued once randomised with similar durations in all three arms ranging from a median of 5 days for antivirals and 9 days for anticoagulants (Table 3). More participants in the SOC arm had received antibiotics at baseline (74.6%) compared to the FOS (58.6%) or RUX (64.1%) arms whereas fewer participants initiated antibiotics after randomisation in the SOC arm (22.2%) compared to the FOS (32.8%) and RUX (25.0%) arms (Supplementary Table S3). While fewer participants in the SOC (14.3%) arm had received an interleukin-6 (IL6) inhibitor at baseline compared to the FOS (19.0%) and RUX (20.3%) arms (Supplementary Table S2), these were initiated more frequently after randomisation in the SOC arm (14.3%) compared to the FOS (6.9%) and RUX (9.4%) arms (Supplementary Table S4).

Table 3: Standard of care treatments received at or prior to randomisation by treatment arm

Drug group	FOS (N=58)	RUX (N=64)	SOC (N=63)
Anti-viral agents			
Received ≥1 dose, n (%)	27 (46.6)	31 (48.4)	29 (46.0)
Median (IQR) time since initiation (days); n missing	0 (0, 1); 0	0 (0, 1); 0	0 (0, 1); 0
Median (IQR) duration (days); n missing	5 (4, 6); 0	5 (5, 6); 0	5 (4, 5); 0
Immuno-modulating agents			
Received ≥1 dose, n (%)	56 (96.6)	58 (90.6)	56 (88.9)
Median (IQR) time since initiation (days); n missing	1 (0, 1); 0	1 (0, 1); 0	1 (0, 1); 0
Median (IQR) duration (days); n missing	6 (4, 9); 0	8 (5, 11); 0	7 (4, 10); 1
Antibiotics			
Received ≥1 dose, n (%)	34 (58.6)	41 (64.1)	47 (74.6)
Median (IQR) time since initiation (days); n missing	1 (0, 2); 0	1 (0, 2); 0	1 (1, 2); 0
Median (IQR) duration (days); n missing	5 (2, 7); 0	4 (2, 7); 0	6 (2, 7); 0
Anticoagulation treatment			
Received ≥1 dose, n (%)	54 (93.1)	54 (84.4)	57 (90.5)
Median (IQR) time since initiation (days); n missing	1 (0, 1); 0	1 (0, 1); 0	1 (0, 1); 0
Median (IQR) duration (days); n missing	9 (5, 25); 1	9 (5, 13); 1	9 (6, 13); 0
Antiplatelet therapy			

For peer review only - http://bmjopen.bmj.com/site/about/guidelines.xhtml

Received ≥1 dose, n (%)	6 (10.3)	1 (1.6)	2 (3.2)
Median (IQR) time since initiation (days); n missing	1 (0, 1); 0	1 (1, 1); 0	2 (0, 2); 0
Median (IQR) duration (days); n missing	17 (3, 30); 4	Missing	1 (1, 1); 0
**Convalescent serum therapy**			
Received ≥1 dose, n (%)	0 (0.0)	1 (1.6)	1 (1.6)
Median (IQR) time since initiation (days); n missing	N/A	6 (6, 6); 0	0 (0, 0); 0
Median (IQR) duration (days); n missing	N/A	1 (1, 1); 0	2 (2, 2); 0
**Ronapreve®**			
Received ≥1 dose, n (%)	1 (1.7)	1 (1.6)	0 (0.0)
Median (IQR) time since initiation (days); n missing	0 (0, 0); 0	0 (0, 0); 0	N/A
Median (IQR) duration (days); n missing	1 (1, 1); 0	1 (1, 1); 0	N/A

N/A: not applicable

Prior to randomisation, similar proportions (approximately 6-9%) of participants had been co-enrolled on another RCT across the three trial arms. Co-enrolment after randomisation to MATIS was higher in the SOC arm (11.1%) and RUX arm (9.4%) than in the FOS arm (3.4%) (Supplementary Table S5).

Primary outcome

At Stage 1 (N=177), there was no evidence of a reduction in the odds of progression to severe COVID-19 pneumonia for FOS: adjusted odds ratio, compared to SOC: 1.12; 95% CI: 0.49 to 2.58; one-sided p value = 0.608 (Table 4). The Independent Data Monitoring Committee (IDMC) recommended stopping the FOS arm for futility at this stage on the basis of the one-sided p-value exceeding the prespecified threshold of 0.25[9]. Recruitment to the RUX (and SOC) arm was recommended to continue (p=0.152, Table 4), however, due to the slow recruitment because of the reduction in new COVID-19 cases, the trial was stopped early on 2nd September 2022 after only recruiting an additional four participants. Therefore, the final analysis presented in this manuscript includes all three trial arms as if they had been a conventional one-stage RCT comparing RUX and FOS with SOC.

For peer review only - http://bmjopen.bmj.com/site/about/guidelines.xhtml

Table 4: Primary Outcome Statistical Model Results

Grade >=5 by Day 14	N	n[a] (%)	Unadjusted		Adjusted[b]	
			Odds ratio (95% CI)	p-value[c]	Odds ratio (95% CI)	p-value[c]
Stage 1						
Fostamatinib	58	16 (27.6)	1.13 (0.50 to 2.58)	0.766	1.12 (0.49 to 2.58)	**0.608**
Ruxolitinib	59	10 (16.9)	0.62 (0.25 to 1.53)	0.300	0.60 (0.22 to 1.60)	**0.152**
SOC	60	15 (25.0)	1.0		1.0	
Final Analysis						
Fostamatinib	58	16 (27.6)	1.19 (0.52 to 2.71)	0.684	1.19 (0.51 to 2.76)	**0.659**
Ruxolitinib	62	10 (16.1)	0.59 (0.24 to 1.44)	0.243	0.63 (0.25 to 1.57)	**0.161**
SOC	61	15 (24.6)	1.0		1.0	

[a] multiple imputation used at Stage 1 and final analysis to impute primary outcome status for 3 patients in the FOS arm, 4 in the RUX arm and 4 in the SOC arm.

[b] Stage1: propensity score adjusted; Final analysis: adjusted for baseline modified WHO COVID severity, age category, use of IL6 inhibitor and prior COVID vaccination

[c] p-values are two-sided except for those in **bold** where one-sided p-values are presented; p-values for final analysis are nominal as planned sample size not reached

In the final analysis (N=181, Table 4), the numbers (%) of participants with severe COVID-19 pneumonia by Day 14 were 16 (27.6%, N=58) and 10 (16.1%, N=62) in the FOS and RUX arms respectively, and 15 (24.6%, N=61) in the SOC arm. There remained a numerically higher odds of reaching a modified WHO COVID-19 severity grade ≥5 in the FOS arm compared to SOC (OR: 1.19; 95% CI: 0.51 to 2.76; nominal one-sided p-value=0.659). In contrast, a numerically lower odds of severe

For peer review only - http://bmjopen.bmj.com/site/about/guidelines.xhtml

COVID-19 pneumonia was observed for RUX compared to SOC (OR: 0.63; 95% CI: 0.25 to 1.57; nominal p=0.161). In both comparisons, the 95% CI was and included 1. Study conclusions remained unchanged when applying alternative missing data assumptions (Supplementary Table S6).

Secondary outcomes

Results from the primary analysis model when extended to include data up to Day 28 (modified WHO COVID-19 severity grade≥ 5 by Day 28) were very similar to that for Day 14 (Supplementary Table S7). The primary outcome at Day 14 and Day 28 was most commonly triggered by participants experiencing oxygen saturation levels < 90% on receipt of ≥ 60% inspired oxygen or receipt of non-invasive ventilation (Supplementary Tables S8 to S10). Seven participants died (4 in the FOS arm and 3 in the SOC arm) on or before Day 14 and a further three participants died (2 in the RUX arm and one in the SOC arm) on or before Day 28. Individual models for each of the components of the primary outcome are presented, however, event counts are small and resulting model estimates are imprecise. Results from the mixed ordinal logistic regression model were broadly consistent with the primary analysis model (Supplementary Table S11).

Nine participants (4 in the FOS arm, 3 in the RUX arm and 2 in the SOC arm) received mechanical ventilation by Day 14, of whom 6 received organ support (inotropes) (Table Supplementary S8). Six participants (2 in the FOS arm, 3 in the RUX arm and one in the SOC arm) had a venous thromboembolism (VTE) event including one participant with a serious VTE in the SOC arm. Two participants (one in the RUX and one in the FOS arm) had the VTE event on the day of randomisation.

For peer review only - http://bmjopen.bmj.com/site/about/guidelines.xhtml

There was no evidence of a difference in the time to discharge from randomisation for either FOS or RUX compared to SOC (Supplementary Figure S1). Hazard ratios from the Cox model were 0.96 (95% CI 0.64 to 1.43) for FOS vs SOC and 1.05 (95% CI 0.71 to 1.55) for RUX vs SOC, after adjusting for age, site, prior COVID vaccination, receipt of steroids and receipt of IL6 inhibitors at baseline. Readmission by Day 28 was more frequent in the RUX (8 participants, 13.8%) and SOC (8, 14.6%) arms than in the FOS arm (3, 5.6%) (Supplementary Table S12). No difference in levels of inflammatory markers was observed between trial arms (Supplementary Table S13). Statistically significant differences in serum creatinine levels were noted with higher mean values in the FOS arm at Day 14 (adjusted mean difference vs. SOC: 34.3 μmol/L; 95% CI 6.8 to 61.8) and at Day 28 (43.6 μmol/L; 95% CI 15.7 to 71.5) and in the FOS arm at Day 28 (adjusted mean difference vs. SOC: 27.2 μmol/L; 95% CI 0.6 to 53.7) (Supplementary Table S13).

Safety

Safety was evaluated in all 181 eligible, randomised participants. Four patients were excluded from this analysis as they were randomised in error. A total of 48 SAEs were reported in 38 participants with a higher proportion experiencing at least one SAE in the RUX (14, 22.6% of participants) and FOS arms (13, 22.4%) compared to SOC (11, 17.5%) (Table 5).

Table 5: Summary of Serious Adverse Events type by treatment arm

	Fostamatinib (N=58)		Ruxolitinib (N=62)		Standard of care (N=61)	
Event type	Number of events	Number of participants with events (%)	Number of events	Number of participants with events (%)	Number of events	Number of participants with events (%)
SAE	14	13 (22.4)	18	14 (22.6)	16	11 (17.5)
SAR	1	1 (1.7)	2	2 (3.2)	0	0 (0.0)

For peer review only - http://bmjopen.bmj.com/site/about/guidelines.xhtml

For peer review only

USAR	0	0 (0.0)	2[a]	2 (3.2)	0	0 (0.0)

Acronyms: SAE – serious adverse event; SAR – serious adverse reaction; USAR – unexpected serious adverse reaction

[a] This included one patient with hypotension and one patient with lymphoedema

Three of the reported SAEs were considered to be possibly related to study treatment. These were classified as serious adverse reactions (SARs) and included one expected SAR in the FOS arm (lung infection) and two unexpected SARs in the RUX arm (one case each of hypotension and lymphoedema). The most frequently reported SAE in all three trial arms were infections with numerically higher frequencies in the FOS (10, 17.2%) and ruxolitinib (10, 16.1%) arms compared to SOC (7, 11.5%) (Supplementary Figures S2 and S3). These included 5 cases of lung infection in RUX arm (4 participants) and 3 cases in in the FOS arm (3 participants), both higher than the SOC arm (1 participant). One event of lung infection in the RUX arm resulted in a fatal outcome. None of the 10 fatal events recorded in the trial were considered to be related to study treatment.

Exploratory analyses

Results from the per-protocol analysis in participants who received ≥ 90% of prescribed dose were consistent with the primary analysis (Supplementary Table S14). The complier-adjusted risk difference (RD) estimates were slightly further away from the null than the corresponding ITT analyses but did not reach statistical significance (complier-adjusted vs ITT: FOS vs SOC: RD 0.05 vs 0.03; RUX vs SOC: RD -0.10 vs -0.06). Stratification of the analysis by grouping participants into approximately four equal sized groups based on time of randomisation resulted in somewhat imprecise estimates and were inconclusive (Supplementary Table S15).

For peer review only - http://bmjopen.bmj.com/site/about/guidelines.xhtml

There was no strong evidence for any subgroup effects (Supplementary Figures S4 and S5). These results should be interpreted with caution due to the small sample size within subgroups.

Discussion

Principal findings

MATIS was an open-label, multi-centre, multi-arm, and multi-stage randomised controlled trial of ruxolitinib and fostamatinib for COVID-19 pneumonia compared to routine standard of care. The study was designed between March and April 2020, with participants recruited largely from the second and third waves of the UK pandemic, with the majority of participants recruited from three hospitals in North-West London. Due to the success of the COVID-19 vaccine leading to a dramatic reduction in hospitalisation and mortality, the study was closed in September 2022, before reaching the power needed to conclude its end points.

Despite being underpowered to make a definitive conclusion, numerically lower odds (OR: 0.63) of progressing to severe COVID-19 pneumonia (modified WHO grade ≥5) were observed with ruxolitinib compared to SOC. However, the 95% CI is wide (0.25 to 1.57) and crosses 1 indicating uncertainty an requires cautious interpretation. In contrast, the odds of progression were numerically higher (OR 1.19) with fostamatinib compared to SOC. Although, again, results were imprecise with a wide 95% CI (0.51 to 2.76).

For peer review only - http://bmjopen.bmj.com/site/about/guidelines.xhtml

For peer review only

Although, the study was not powered to assess the secondary outcome of death, no deaths were recorded in the ruxolitinib arm in the first 14 days while 4 participants in the fostamatinib arm and 3 in the SOC arm died during this period. There was no difference in time to discharge between either active treatment and SOC, but there were fewer readmissions in the fostamatinib arm. In terms of safety, there were higher numbers of lung infections in the active treatment arms including one infection-related death in the ruxolitinib arm. However this event was not considered to be related to study treatment. We noted an increase in serum creatinine in both arms; however the clinical relevance of this finding is unclear.

Approximately one third of participants did not complete the 14-day treatment course for fostamatinib and ruxolitinib. The results of our complier-adjusted case analyses, which estimated the impact of adherence on the treatment effects, were consistent with our primary analysis. However, due to the pandemic setting, adherence measures were based on self-report for participants given take-home medication following hospital discharge rather than in-person pill counts. Our estimate of adherence and its impact on treatment effects may therefore be under-estimated.

Comparison with other studies

Ruxolitinib is an inhibitor of the JAK/STAT pathway (Janus kinases and Signal Transducer and Activator of Transcription proteins), specifically inhibiting subtypes JAK1 and JAK2. It has a rapid mode of action with cytokine-induced STAT3 phosphorylation being inhibited within 2 hours of administration, resulting in a reduction of tumour necrosis factor alpha (TNFα), IL6 and CRP. Several other trials

For peer review only - http://bmjopen.bmj.com/site/about/guidelines.xhtml

studied the effect of ruxolitinib during the pandemic, with variable responses reported, some positive, but most were neutral.[15-17] There were many differences between studies including: types of patients recruited (i.e. differing severity of COVID-19), timing of the study in the pandemic (resulting in variability of the virus over time, changes in standard of care; and the vaccine role out in 2021), and different primary outcomes. As with MATIS, some of these trials were underpowered due to early termination. Furthermore, the appropriate dosing of ruxolitinib to treat or prevent hyperinflammation is yet to be determined. We chose a low to intermediate dose for MATIS, because we had concerns regarding immunosuppression during active phase of the infection. This dose appeared to be appropriate for this cohort of patients given the lower rate of progression to more severe disease and absence of deaths before 14 days in the ruxolitinib arm. Higher doses such as 25mg once a day, used in conditions such as graft versus host disease,[18] or longer duration of treatment (>14 days) may be more appropriate for patients with more severe COVID-19.

Given the CRP and IL6 drive in COVID-19, several clinical trials of other agents in this class (including baracitinib and tofacitinib) targeting patients at different disease stages were performed.[19 20] A meta-analysis of four randomised controlled trials of baracitinib in hospitalised patients with COVID-19, including 10,815 patients, showed a reduction in 28-day mortality (OR 0.69, 95% CI 0.5-0.94; p=0.04).[21] Results from two of the large studies included in the meta-analysis, RECOVERY and COV-BARRIER,[22 23] led to the US Food and Drug Administration (FDA), allowing the emergency authorisation of baracitinib with remdesivir in patients with COVID-19 requiring supplemental oxygen, invasive mechanical ventilation, or extracorporeal membrane oxygenation (ECMO).[24]

For peer review only - http://bmjopen.bmj.com/site/about/guidelines.xhtml

CONFIDENTIAL PRE-PUBLICATION MANUSCRIPT

The RECOVERY study also observed a 15% reduction mortality for tocilizumab (an IL6 inhibitor) compared to usual care.[25] Tociluzimab was added to standard of care during MATIS and was used in approximately one fifth of the patients in this trial.

Fostamatinib is a SYK pathway inhibitor, licensed for use in patients with immune thrombocytopenia. The SYK pathway appears to be critical in the pathogenesis of COVID-19 as well as other severe acute respiratory syndrome (SARS) infections, modulating cytokine secretion, and promoting the influx of immune cells such as macrophages and monocyte into the lung, contributing to both the hyperinflammatory state and long-term lung damage. As such, SYK inhibition appeared as a novel mechanism to treat COVID-19 pneumonia through the prevention of cytokine release, lung inflammation and platelet activation, all critical events in COVID-19 pathway. [26 27]

Despite a plausible biological mechanism of action, we found no evidence that fostamatinib prevented progression of COVID-19 to more severe disease in this cohort of patients. It is possible that our patient cohort were too mildly affected to have any benefit from fostamatinib. It is also possible we did not use the correct dose, or duration of fostamatinib. Another RCT comparing fostamatinib 100 mg twice daily plus SOC to placebo plus SOC included patients with more severe COVID-19 (over half of the patients required non-invasive ventilation or high-flow oxygen devices [56.7% fostamatinib and 51.7% placebo], or invasive mechanical ventilation and/or ECMO [36.7% FOS and 41.1% placebo]).[28] In that study, the rates of SAEs by Day 29 (the primary end point) were not statistically different between the fostamatinib group and the placebo group (10.5% vs 22.0%, p=0.2), while secondary endpoints, which the study was not powered for, such as 28-day mortality, days on supplemental oxygen and ordinal scores of clinical status favoured fostamatinib. Both

For peer review only - http://bmjopen.bmj.com/site/about/guidelines.xhtml

the inclusion criteria and the dose of fostamatinib were different to MATIS limiting their comparison, but potentially implying the timing of the use of fostamatinib in diseases such as COVID-19 might be critical.

Another double blind, randomized, placebo-controlled trial of fostamatinib in patients with COVID-19 on supplementary oxygen (the FOCUS trial) recruited 280 patients.[29] Although results are not yet published in full, the FOCUS trial met its primary endpoint with patients in the fostamatinib arm having a reduced number of days on supplemental oxygen compared to placebo (4.8 vs. 7.6 days, respectively, p=0.0136). Fostamatinib also showed a trend in reducing COVID-19 related mortality and morbidity. Unlike MATIS, this study also recruited patients with more severe COVID-19; all 3 participants with a baseline score of 6 on the 8-point COVID-19 ordinal scale in the placebo group died by Day 30, and all 3 participants with this baseline score in the fostamatinib group survived, suggesting there may be particular benefit for patients with more severe COVID-19, although numbers in this cohort remain very small.

The study designs were different for these trials, limiting comparisons, but overall, it is possible that the timing of the use of fostamatinib in diseases such as COVID-19 is critical, with greater benefit in patients with more severe disease, preventing the progression of lung disease, rather than earlier in the infection, when anti-viral responses may be more important.

Strength and Limitations:

Patient population and recruitment:

CONFIDENTIAL PRE-PUBLICATION MANUSCRIPT

Despite a strong study design, we were unable to recruit the numbers of participants needed to detect a treatment effect for ruxolitinib or fostamatinib due to reducing numbers of patients being admitted to hospital as the pandemic evolved. Recruitment to MATIS was also limited by competition from other large concurrent COVID-19 trials. Another limitation is the representativeness of patients in the trial in terms of age of the patients recruited. Older patients, who had the highest mortality in COVID-19, although approached for MATIS, were less likely to consent to the trial. We do not know the reason for this, but possible causes include family members declining study; fear of the medical profession; fear of the virus; patients being too unwell to read the patient information sheet; flaws in recruitment strategies in a fast-moving time. Recruitment of participants was predominantly from one large teaching hospital in London with a small number recruited from hospitals in other UK cities. Patients were admitted to this hospital through general acute medicine pathways rather than through any tertiary referral mechanism. We therefore consider the study population to be broadly representative of the general urban UK population requiring hospital admission for treatment of COVID-19. The demographics of participants showed broad ethnic representation (approximately 40% of participants were white and 60% were non-white), reflecting the recruitment from inner city hospitals in the UK and was a strength of the study.

The target population in MATIS was restricted to hospitalised patients. This included patients admitted with escalating symptoms and/or with a higher risk of progression to more severe disease due to underlying comorbidities. The trial was not designed to explore whether these treatments may offer any benefits to the wider community

For peer review only - http://bmjopen.bmj.com/site/about/guidelines.xhtml

of patients with milder COVID-19 or to those that did not seek or receive hospital care.

Evolving Standard of Care

At or prior to randomisation, 46.0% to 48.4% of patients in MATIS received anti-viral agents, 88.9% to 96.6% received immune-modulating agents, 58.6% to 74.6% received antibiotics, 84.4% to 93.1% received anticoagulation treatment, and 1.6% to 10.3% received antiplatelet therapy. The frequency of use of these agents was similar between trial arms but we did not examine whether there were any differences in the numbers of agents or doses used. However, the standard of care for treating COVID-19 changed over time, reflecting the dynamic nature of the evolving pandemic. Initially treatments were focused on supportive care strategies. As standard of care evolved, antiviral medications, anti-inflammatory agents, and eventually vaccination campaigns were integrated into treatment protocols. In 2021, there was a successful role out of vaccines. The changes in recommended treatments over distinct phases of the pandemic created additional complexity to COVID-19 treatment clinical trials impacting on patient profile, recruitment numbers and the changing use of multiple immunomodulatory agents. Within MATIS, the use of IL6-inhibitors was introduced at Imperial College NHS Trust (the main study site) for patients who were developing hyper-inflammation. We note that fewer patients in the active treatment arms required treatment with an IL6 inhibitor post-randomisation.

Implications for practice and future research

One of the unique contributing factors to the high individual mortality, as well as the population level impact of COVID-19, was the triggering of a hyperinflammatory state causing a rapid escalation to ventilation in many patients. The excessive need for

For peer review only - http://bmjopen.bmj.com/site/about/guidelines.xhtml

ventilation also impacted the rest of the population with hospitals running out of ventilators and intensive care staff.

In designing MATIS, we chose two different immune modulators as early interventions (within the first few days of admission), aiming to reduce inflammation, prevent the requirement for ventilation and improve the outcome for these patients, as well as reducing the impact of COVID-19 on UK hospitals.

To identify patients who we thought were at risk of hyperinflammation, and therefore more likely to benefit from immune modulation, we included patients with a raised CRP. However, this may not be specific enough. The RuxCoFlam trial which showed a significant effect of ruxolitinib selected patients based on a composite COVID-19 inflammation score (CIS) made up of multiple inflammatory markers.[30] This included inter alia CRP, ferritin, triglcycerides, fibrinogen, total white blood cell count, lymphocytes and D-dimer and activated partial thromboplastin time. For CRP to contribute to the CIS score, values were required to exceed 20 times the upper limit of the normal reference range. Subsequent research, including evidence from our own studies, has shown the importance of host genetic factors in COVID-19 mortality demonstrating an exaggerated hyperinflammatory response to this novel virus.[31,32] Testing patients for these host factors, and measuring specific immune responses, may allow us to target patients more effectively for immune modulation, and clarify which modulators are most appropriate at an individual level. A biomarker study is now underway in samples stored prior to randomisation of MATIS to see if we can better fine-tune immune modulation therapy in virally induced hyperinflammation. Other patient characteristics such as age, obesity, diabetes and cardiovascular risk factors are independently associated with the development of secondary infection thrombosis, requirement for ventilation and death.[33] Meta-analysis of JAK/STAT

For peer review only - http://bmjopen.bmj.com/site/about/guidelines.xhtml

inhibition from all studies may help identify the cohorts of patients most likely to benefit from these treatments.

The timing and choice of immune-modulation in diseases such as COVID-19 could be critical for improving outcome, giving the immune system time to effectively eradicate the virus, but preventing the rapid escalation to hyperinflammation.

The results of these studies are likely to be important in guiding the management of other virally induced multisystemic disorders.

Conclusion

In conclusion, we did not observe a significant treatment effect for fostamatinib in comparison to standard of care in reducing the proportion of hospitalised patients progressing to severe COVID-19 pneumonia. Results for ruxolitinib could not be confirmed due to early termination of our trial. Other factors such as patient comorbidities, choice of dose and timing of treatment may also have contributed to these findings. Further research is needed to help us to further understand these results and further investigation of the role of JAK/STAT inhibition in hyperinflammatory conditions triggered by viruses is now warranted, as well as long term follow up of patients who received immune modulation.

For peer review only

CONFIDENTIAL PRE-PUBLICATION MANUSCRIPT

References

1. Huang C, Wang Y, Li X, et al. Clinical features of patients infected with 2019 novel coronavirus in Wuhan, China. Lancet 2020;395(10223):497-506. doi: 10.1016/S0140-6736(20)30183-5 [published Online First: 20200124]

2. Ruan Q, Yang K, Wang W, et al. Clinical predictors of mortality due to COVID-19 based on an analysis of data of 150 patients from Wuhan, China. Intensive Care Med 2020;46(5):846-48. doi: 10.1007/s00134-020-05991-x [published Online First: 20200303]

3. Siddiqi HK, Mehra MR. COVID-19 illness in native and immunosuppressed states: A clinical-therapeutic staging proposal. J Heart Lung Transplant 2020;39(5):405-07. doi: 10.1016/j.healun.2020.03.012 [published Online First: 20200320]

4. Spalton JC, Mori J, Pollitt AY, et al. The novel Syk inhibitor R406 reveals mechanistic differences in the initiation of GPVI and CLEC-2 signaling in platelets. J Thromb Haemost 2009;7(7):1192-9. doi: 10.1111/j.1538-7836.2009.03451.x [published Online First: 20090424]

5. Lhermusier T, van Rottem J, Garcia C, et al. The Syk-kinase inhibitor R406 impairs platelet activation and monocyte tissue factor expression triggered by

For peer review only - http://bmjopen.bmj.com/site/about/guidelines.xhtml

heparin-PF4 complex directed antibodies. J Thromb Haemost 2011;9(10):2067-76. doi: 10.1111/j.1538-7836.2011.04470.x

6. Novartis. Jakavi 10mg tablets. 2022 [Available from: https://www.medicines.org.uk/emc/product/7786/smpc#gref.

7. Grifols UK Ltd. Tavlesse 100 mg film-coated tablets 2023 [Available from: https://www.medicines.org.uk/emc/product/11479/smpc#gref accessed 7th August 2024.

8. World Health Organisation. COVID-19 Therapeutic Trial Synopsis. Geneva Switzerland: World Health Organisation, 2020.

9. Vergis N, Phillips R, Cornelius V, et al. Multi-arm Trial of Inflammatory Signal Inhibitors (MATIS) for hospitalised patients with mild or moderate COVID-19 pneumonia: a structured summary of a study protocol for a randomised controlled trial. Trials 2021;22(1):270. doi: 10.1186/s13063-021-05190-z [published Online First: 20210412]

10. Schulz KF, Altman DG, Moher D, et al. CONSORT 2010 statement: updated guidelines for reporting parallel group randomised trials. BMJ 2010;340:c332. doi: 10.1136/bmj.c332 [published Online First: 20100323]

11. White IR, Royston P, Wood AM. Multiple imputation using chained equations: Issues and guidance for practice. Stat Med 2011;30(4):377-99. doi: 10.1002/sim.4067 [published Online First: 20101130]

12. Cook JA, MacLennan GS, Palmer T, et al. Instrumental variable methods for a binary outcome were used to informatively address noncompliance in a randomized trial in surgery. J Clin Epidemiol 2018;96:126-32. doi: 10.1016/j.jclinepi.2017.11.011 [published Online First: 20171120]

For peer review only

For peer review only - http://bmjopen.bmj.com/site/about/guidelines.xhtml

13. Rickham PP. Human Experimentation. Code of Ethics of the World Medical Association. Declaration of Helsinki. Br Med J 1964;2(5402):177. doi: 10.1136/bmj.2.5402.177

14. Elliott P, Whitaker M, Tang D, et al. Design and Implementation of a National SARS-CoV-2 Monitoring Program in England: REACT-1 Study. Am J Public Health 2023;113(5):545-54. doi: 10.2105/AJPH.2023.307230 [published Online First: 20230309]

15. Cao Y, Wei J, Zou L, et al. Ruxolitinib in treatment of severe coronavirus disease 2019 (COVID-19): A multicenter, single-blind, randomized controlled trial. J Allergy Clin Immunol 2020;146(1):137-46 e3. doi: 10.1016/j.jaci.2020.05.019 [published Online First: 20200526]

16. Han MK, Antila M, Ficker JH, et al. Ruxolitinib in addition to standard of care for the treatment of patients admitted to hospital with COVID-19 (RUXCOVID): a randomised, double-blind, placebo-controlled, phase 3 trial. Lancet Rheumatol 2022;4(5):e351-e61. doi: 10.1016/S2665-9913(22)00044-3 [published Online First: 20220329]

17. Rein L, Calero K, Shah R, et al. Randomized Phase 3 Trial of Ruxolitinib for COVID-19-Associated Acute Respiratory Distress Syndrome. Crit Care Med 2022;50(12):1701-13. doi: 10.1097/CCM.0000000000005682 [published Online First: 20221013]

18. Broglie L, Pommert L, Rao S, et al. Ruxolitinib for treatment of refractory hemophagocytic lymphohistiocytosis. Blood Adv 2017;1(19):1533-36. doi: 10.1182/bloodadvances.2017007526 [published Online First: 20170817]

For peer review only

For peer review only - http://bmjopen.bmj.com/site/about/guidelines.xhtml

19. Guimaraes PO, Quirk D, Furtado RH, et al. Tofacitinib in Patients Hospitalized with Covid-19 Pneumonia. N Engl J Med 2021;385(5):406-15. doi: 10.1056/NEJMoa2101643 [published Online First: 20210616]

20. Kalil AC, Patterson TF, Mehta AK, et al. Baricitinib plus Remdesivir for Hospitalized Adults with Covid-19. N Engl J Med 2021;384(9):795-807. doi: 10.1056/NEJMoa2031994 [published Online First: 20201211]

21. Selvaraj V, Finn A, Lal A, et al. Baricitinib in hospitalised patients with COVID-19: A meta-analysis of randomised controlled trials. EClinicalMedicine 2022;49:101489. doi: 10.1016/j.eclinm.2022.101489 [published Online First: 20220603]

22. Recovery Collaborative Group. Baricitinib in patients admitted to hospital with COVID-19 (RECOVERY): a randomised, controlled, open-label, platform trial and updated meta-analysis. Lancet 2022;400(10349):359-68. doi: 10.1016/S0140-6736(22)01109-6

23. Marconi VC, Ramanan AV, de Bono S, et al. Efficacy and safety of baricitinib for the treatment of hospitalised adults with COVID-19 (COV-BARRIER): a randomised, double-blind, parallel-group, placebo-controlled phase 3 trial. Lancet Respir Med 2021;9(12):1407-18. doi: 10.1016/S2213-2600(21)00331-3 [published Online First: 20210901]

24. US Food and Drug Administration. Letter of authorization: EUA for baricitinib (Olumiant) for treatment of coronavirus disease 2019 (COVID-19). 2022. https://www.fda.gov/media/143822/download (accessed 23/12/2024).

25. Recovery Collaborative Group. Tocilizumab in patients admitted to hospital with COVID-19 (RECOVERY): a randomised, controlled, open-label, platform trial. Lancet 2021;397(10285):1637-45. doi: 10.1016/S0140-6736(21)00676-0

For peer review only

26. Nadeem A, Ahmad SF, Al-Harbi NO, et al. Inhibition of spleen tyrosine kinase signaling protects against acute lung injury through blockade of NADPH oxidase and IL-17A in neutrophils and gammadelta T cells respectively in mice. Int Immunopharmacol 2019;68:39-47. doi: 10.1016/j.intimp.2018.12.062 [published Online First: 20190102]

27. Alimova M, Sidhom EH, Satyam A, et al. A High Content Screen for Mucin-1-Reducing Compounds Identifies Fostamatinib as a Candidate for Rapid Repurposing for Acute Lung Injury during the COVID-19 pandemic. bioRxiv 2020 doi: 10.1101/2020.06.30.180380 [published Online First: 20200630]

28. Strich JR, Tian X, Samour M, et al. Fostamatinib for the Treatment of Hospitalized Adults With Coronavirus Disease 2019: A Randomized Trial. Clin Infect Dis 2022;75(1):e491-e98. doi: 10.1093/cid/ciab732

29. Gotur DB, Malik A, Markovtsov V, et al. 88. Fostamatinib for the Treatment of Hospitalized Patients With COVID-19 Who Required Oxygen Supplementation: Results of a Phase 3 Trial. Open Forum Infectious Diseases 2023;10(Supplement_2) doi: 10.1093/ofid/ofad500.004

30. Hammersen J, Birndt S, Dohner K, et al. The JAK1/2 inhibitor ruxolitinib in patients with COVID-19 triggered hyperinflammation: the RuxCoFlam trial. Leukemia 2023;37(9):1879-86. doi: 10.1038/s41375-023-01979-w [published Online First: 20230728]

31. Velavan TP, Pallerla SR, Ruter J, et al. Host genetic factors determining COVID-19 susceptibility and severity. EBioMedicine 2021;72:103629. doi: 10.1016/j.ebiom.2021.103629 [published Online First: 20211013]

For peer review only

32. Malik A, Toth EN, Teng MS, et al. Distorted TCR repertoires define multisystem inflammatory syndrome in children. PLoS One 2022;17(10):e0274289. doi: 10.1371/journal.pone.0274289 [published Online First: 20221027]

33. Ruan Q, Yang K, Wang W, et al. Correction to: Clinical predictors of mortality due to COVID-19 based on an analysis of data of 150 patients from Wuhan, China. Intensive Care Med 2020;46(6):1294-97. doi: 10.1007/s00134-020-06028-z

Acknowledgements

We would like to thank the patients (and their families) who took part in the trial, the clinical staff at participating hospitals and the research team at Imperial College Healthcare NHS Trust.

Data sharing

De-identified participant-level data (excluding free text fields) and supporting documentation (including the MATIS study protocol, Statistical Analysis Plan and data dictionary) can be made available upon reasonable request from the corresponding author (n.cooper@imperial.ac.uk) for the purposes of scientific research including secondary analysis of the data or for individual participant meta-analysis with appropriate human research ethics approvals and data transfer agreements in place.

For peer review only

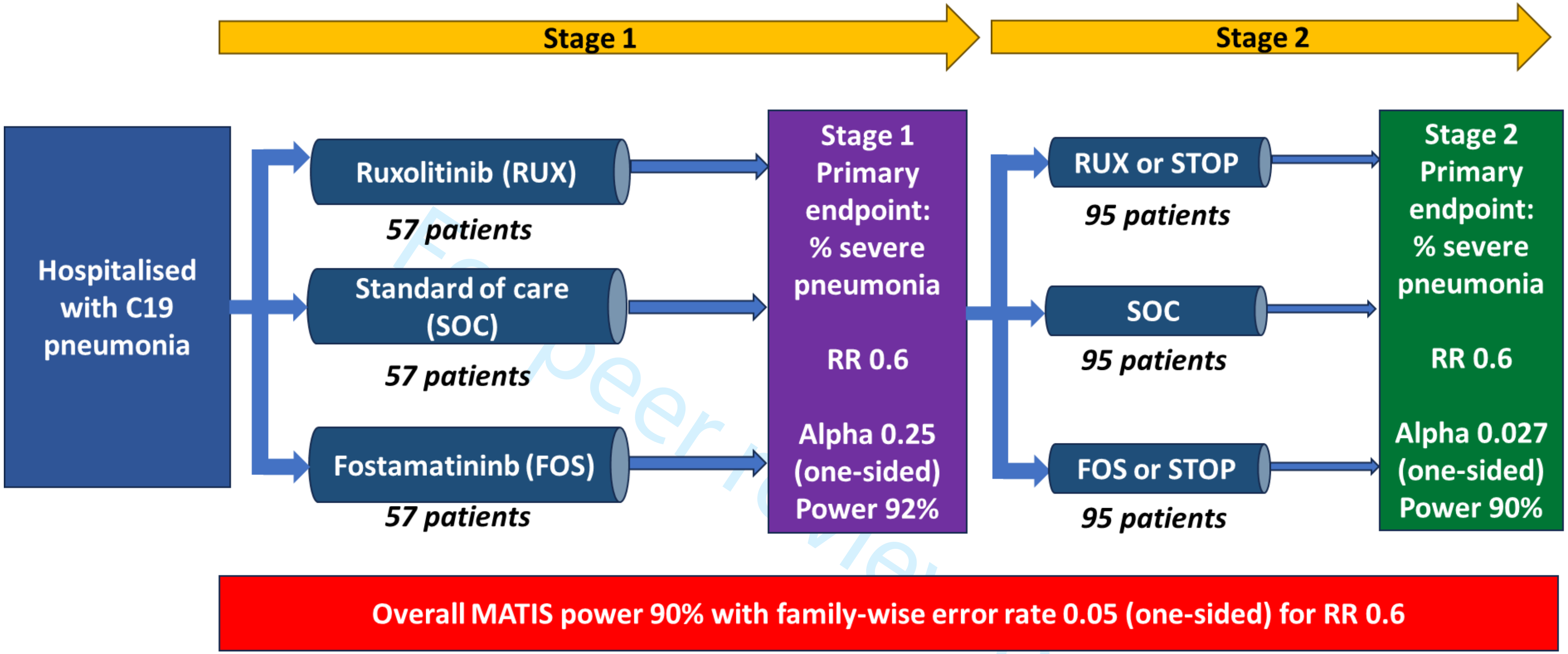

Figure 1: MATIS Trial Design

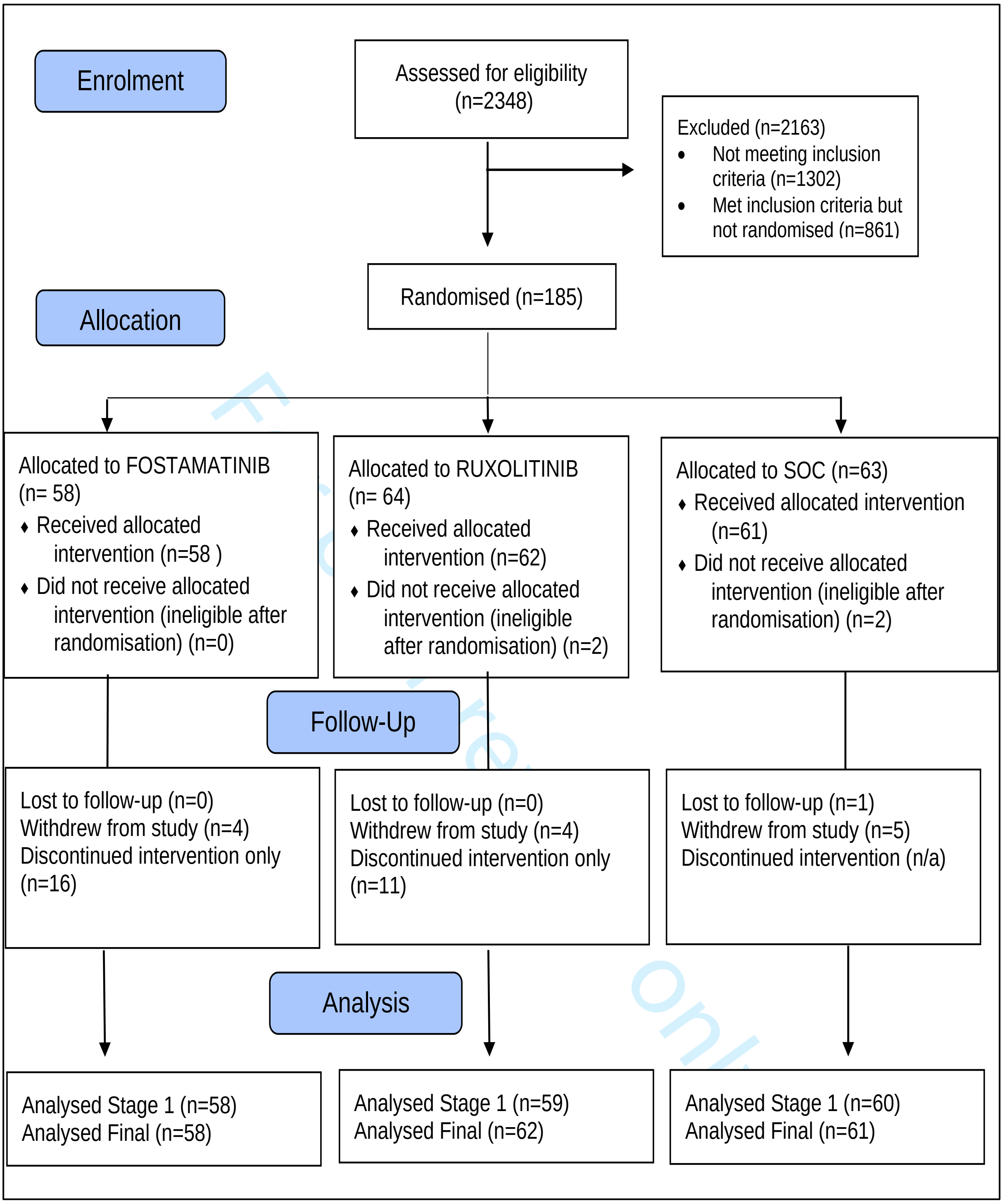

Figure 2: Participant flow

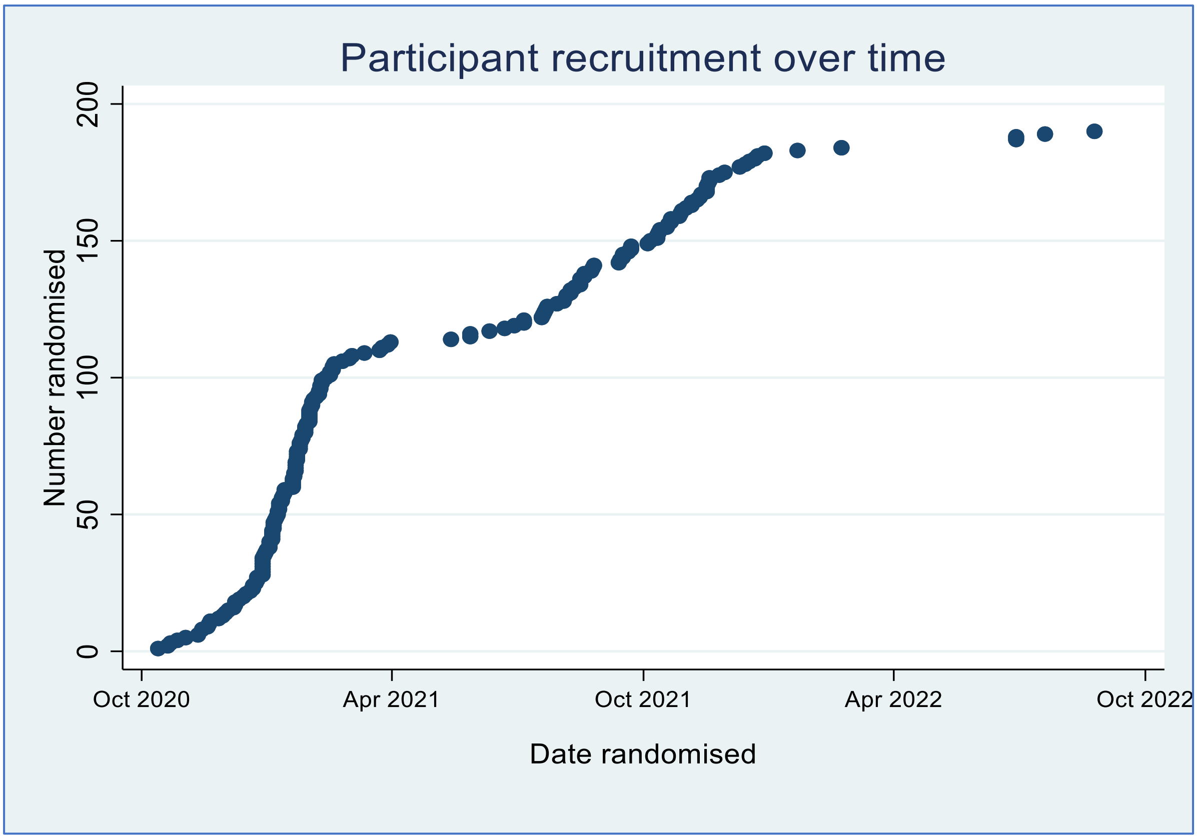

Figure 3: Recruitment over time

review only

Supplementary Appendix 2

Characteristics of participants in MATIS stratified by predominant COVID-19 variant at time of randomisation

For peer review only

Table S1: Baseline characteristics of all participants by predominant variant at time of randomisation

Patient characteristic, n(%) unless otherwise specified	Alpha (up to May 2021) (N=112)	Delta (May to Dec 2021) (N=61)	Omicron (post Dec 2021) (N=12)
Age in years			
Mean (SD)	60.8 (14.7)	55.9 (16.6)	65.8 (17.1)
Sex			
Male	79 (70.5)	43 (70.5)	6 (50.0)
Female	33 (29.5)	18 (29.5)	6 (50.0)
Ethnicity			
White	41 (36.6)	30 (49.2)	8 (66.7)
Mixed or multiple ethnic groups	1 (0.9)	1 (1.6)	0 (0.0)
Asian or Asian British	14 (12.5)	8 (13.1)	4 (33.3)
Black, Black British, Caribbean or African	12 (10.7)	7 (11.5)	0 (0.0)
Other ethnic group	44 (39.3)	15 (24.6)	0 (0.0)
BMI in kg/m2			
N (N missing)	106 (6)	55 (6)	11 (1)
Mean (SD)	30.2 (6.4)	31.0 (7.8)	26.5 (5.1)
Severity of Covid			
Grade 3	17 (15.2)	13 (21.3)	3 (25.0)
Grade 4	95 (84.8)	48 (78.7)	9 (75.0)
Chronic lung disease			
N (N missing)	111 (1)	60 (1)	12 (0)
Yes	15 (13.5)	13 (21.7)	2 (16.7)
Time from onset of symptoms			
N (N missing)	103 (9)	51 (10)	11 (1)
Mean (SD)	9 (4)	10 (4)	12 (5)
Diabetes			
N (N missing)	111 (1)	60 (1)	12 (0)
Yes	37 (33.3)	13 (21.7)	4 (33.3)
Hypertension			
N (N missing)	111 (1)	60 (1)	12 (0)
Yes	53 (47.7)	21 (35.0)	7 (58.3)
Ischaemic heart disease			
N (N missing)	111 (1)	60 (1)	12 (0)
Yes	17 (15.3)	9 (15.0)	5 (41.7)
Heart failure			
N (N missing)	111 (1)	60 (1)	12 (0)
Yes	3 (2.7)	2 (3.3)	2 (16.7)
Immunocompromised			
N (N missing)	111 (1)	60 (1)	12 (0)
Yes	4 (3.6)	2 (3.3)	2 (16.7)
End-stage renal failure			
N (N missing)	111 (1)	60 (1)	12 (0)
Yes	10 (9.0)	2 (3.3)	1 (8.3)
Liver cirrhosis			
N (N missing)	111 (1)	60 (1)	12 (0)
Yes	0 (0.0)	0 (0.0)	0 (0.0)
Current smoker			

For peer review only

N (N missing)	110 (2)	60 (1)	12 (0)
Yes	5 (4.5)	0 (0.0)	0 (0.0)
Prior Covid Vaccination			
N (N missing)	112 (0)	61 (0)	12 (0)
Yes	9 (8.0)	20 (32.8)	6 (50.0)
Serum creatinine (µmol/L)			
N (N missing)	111 (1)	59 (2)	12 (0)
Mean (SD)	139 (209)	95 (85)	115 (112)
C-reactive protein (mg/L)			
N (N missing)	111 (1)	59 (2)	12 (0)
Mean (SD)	115 (70)	100 (75)	76 (46)
Lactate dehydrogenase (IU/L)			
N (N missing)	73 (39)	28 (33)	8 (4)
Mean (SD)	478 (255)	388 (153)	390 (173)
Ferritin (µg/L)			
N (N missing)	98 (14)	43 (18)	10 (2)
Mean (SD)	1659 (2343)	1235 (1145)	472 (285)
D-dimer (ng/ml)			
N (N missing)	103 (9)	49 (12)	9 (3)
Mean (SD)	1851 (3302)	996 (764)	1391 (1082)

For peer review only

Supplementary Appendix 3

Standard of care treatments received pre- and post-randomisation

For peer review only

Table S2: Selected immunological therapies at or prior to randomisation by treatment arm

Drug	FOS (N=58)	RUX (N=64)	SOC (N=63)
Dexamethasone			
Received ≥1 dose, n (%)	56 (96.6)	57 (89.1)	55 (87.3)
Median (IQR) time since initiation (days); n missing	1 (0, 1); 0	1 (1, 2); 0	1 (0, 1); 0
Median (IQR) duration (days), n missing	7 (5, 9); 0	9 (5, 13); 0	8 (5, 10); 1
Prednisolone			
Received ≥1 dose, n (%)	0 (0.0)	1 (1.6)	1 (1.6)
Median (IQR) time since initiation (days); n missing	N/A	1 (1, 1); 0	1 (1, 1); 0
Median (IQR) duration (days), n missing	N/A	6 (6, 6); 0	1 (1, 1); 0
Methylprednisolone			
Received ≥1 dose, n (%)	0 (0.0)	0 (0.0)	0 (0.0)
Median (IQR) time since initiation (days); n missing	N/A	N/A	N/A
Median (IQR) duration (days), n missing	N/A	N/A	N/A
Hydrocortisone			
Received ≥1 dose, n (%)	0 (0.0)	1 (1.6)	0 (0.0)
Median (IQR) time since initiation (days); n missing	N/A	1 (1, 1); 0	N/A
Median (IQR) duration (days), n missing	N/A	1 (1, 1); 0	N/A
Tocilizumab			
Received ≥1 dose, n (%)	9 (15.5)	8 (12.5)	7 (11.1)
Median (IQR) time since initiation (days); n missing	0 (0, 1); 0	0 (0, 1); 0	0 (0, 1); 0
Median (IQR) duration (days), n missing	1 (1, 1); 0	1 (1, 1); 0	1 (1, 1); 0
Sarilumab			
Received ≥1 dose, n (%)	2 (3.4)	5 (7.8)	2 (3.2)
Median (IQR) time since initiation (days); n missing	1 (0, 1); 0	0 (0, 1); 0	1 (0, 1); 0
Median (IQR) duration (days), n missing	1 (1, 1); 0	1 (1, 1); 0	1 (1, 1); 0
Any IL6 therapy			
Received ≥1 dose, n (%)	11 (19.0)	13 (20.3)	9 (14.3)
Median (IQR) time since initiation (days); n missing	0 (0, 1); 0	0 (0, 1); 0	0 (0, 1); 0
Median (IQR) duration (days), n missing	1 (1, 1); 0	1 (1, 1); 0	1 (1, 1); 0
Baricitinib			
Received ≥1 dose, n (%)	0 (0.0)	0 (0.0)	0 (0.0)
Median (IQR) time since initiation (days); n missing	N/A	N/A	N/A
Median (IQR) duration (days), n missing	N/A	N/A	N/A

N/A: not applicable

For peer review only

Table S3 Standard of care treatments after randomisation by treatment arm

Drug group	FOS (N=58)	RUX (N=64)	SOC (N=63)
Anti-viral agents			
Received ≥1 dose, n (%)	3 (5.2)	3 (4.7)	3 (4.8)
Median (IQR) time since initiation (days); n missing	10 (6, 11); 0	1 (1, 3); 0	1 (1, 1); 0
Median (IQR) duration (days); n missing	1 (1, 4); 0	5 (5, 5); 0	5 (5, 6); 0
Immuno-modulating agents			
Received ≥1 dose, n (%)	14 (24.1)	13 (20.3)	16 (25.4)
Median (IQR) time since initiation (days); n missing	8 (5, 10); 0	9 (2, 14); 0	3 (1, 5); 0
Median (IQR) duration (days); n missing	18 (2, 22); 2	8 (1, 11); 0	3 (1, 24); 0
Antibiotics			
Received ≥1 dose, n (%)	19 (32.8)	16 (25.0)	14 (22.2)
Median (IQR) time since initiation (days); n missing	7 (4, 13); 0	8 (4, 14); 0	5 (1, 12); 0
Median (IQR) duration (days); n missing	6 (3, 8); 1	7 (3, 13); 0	6 (3, 8); 0
Anticoagulation treatment			
Received ≥1 dose, n (%)	6 (10.3)	7 (10.9)	10 (15.9)
Median (IQR) time since initiation (days); n missing	10 (2, 19); 0	5 (3, 8); 0	8 (3, 10); 0
Median (IQR) duration (days); n missing	20 (9, 31); 1	8 (2, 22); 0	18 (5, 28); 3
Antiplatelet therapy			
Received ≥1 dose, n (%)	3 (5.2)	2 (3.1)	2 (3.2)
Median (IQR) time since initiation (days); n missing	1 (1, 2); 0	6 (1, 11); 0	2 (2, 2); 0
Median (IQR) duration (days); n missing	4 (4, 27); 0	16 (16, 16); 1	29 (29, 29); 0
Convalescent serum therapy			
Received ≥1 dose, n (%)	0 (0.0)	2 (3.1)	1 (1.6)
Median (IQR) time since initiation (days); n missing	N/A	1 (1, 2); 0	1 (1, 1); 0
Median (IQR) duration (days); n missing	N/A	2 (1, 2); 0	2 (2, 2); 0
Ronapreve®			
Received ≥1 dose, n (%)	3 (5.2)	2 (3.1)	2 (3.2)
Median (IQR) time since initiation (days); n missing	2 (1, 5); 0	2 (1, 3); 0	2 (1, 2); 0
Median (IQR) duration (days); n missing	1 (1, 1); 0	1 (1, 1); 0	1 (1, 1); 0

NA: not applicable

For peer review only

Table S4 Selected immunological therapies after randomisation by treatment arm

Drug	FOS (N=58)	RUX (N=64)	SOC (N=63)
Dexamethasone			
Received ≥1 dose, n (%)	0 (0.0)	2 (3.1)	4 (6.3)
Median (IQR) time since initiation (days); n missing	N/A	1 (1, 1); 0	2 (1, 3); 0
Median (IQR) duration (days), n missing	N/A	5 (4, 5); 0	17 (4, 29); 0
Prednisolone			
Received ≥1 dose, n (%)	6 (10.3)	4 (6.3)	2 (3.2)
Median (IQR) time since initiation (days); n missing	8 (8, 10); 0	16 (14, 22); 0	13 (13, 14); 0
Median (IQR) duration (days), n missing	22 (18, 25); 2	12 (8, 15); 0	24 (20, 24); 0
Methylprednisolone			
Received ≥1 dose, n (%)	3 (5.2)	2 (3.1)	0 (0.0)
Median (IQR) time since initiation (days); n missing	3 (1, 4); 0	3 (3, 3); 0	N/A
Median (IQR) duration (days), n missing	3 (1, 4); 0	3 (3, 3); 0	N/A
Hydrocortisone			
Received ≥1 dose, n (%)	3 (5.2)	1 (1.6)	1 (1.6)
Median (IQR) time since initiation (days); n missing	7 (5, 16); 0	8 (8, 8); 0	5 (5, 5); 0
Median (IQR) duration (days), n missing	2 (1, 3); 0	1 (1, 1); 0	3 (3, 3); 0
Tocilizumab			
Received ≥1 dose, n (%)	4 (6.9)	6 (9.4)	9 (14.3)
Median (IQR) time since initiation (days); n missing	2 (1, 8); 0	2 (1, 2); 0	3 (1, 4); 0
Median (IQR) duration (days), n missing	1 (1, 1); 0	1 (1, 1); 0	1 (1, 2); 0
Sarilumab			
Received ≥1 dose, n (%)	0 (0.0)	0 (0.0)	0 (0.0)
Median (IQR) time since initiation (days); n missing	N/A	N/A	N/A
Median (IQR) duration (days), n missing	N/A	N/A	N/A
Any IL6 therapy			
Received ≥1 dose, n (%)	4 (6.9)	6 (9.4)	9 (14.3)
Median (IQR) time since initiation (days); n missing	2 (1, 8); 0	2 (1, 2); 0	3 (1, 4); 0
Median (IQR) duration (days), n missing	1 (1, 1); 0	1 (1, 1); 0	1 (1, 2); 0
Baricitinib			
Received ≥1 dose, n (%)	0 (0.0)	0 (0.0)	1 (1.6)
Median (IQR) time since initiation (days); n missing	N/A	N/A	11 (11, 11); 0
Median (IQR) duration (days), n missing	N/A	N/A	11 (11, 11); 0

N/A: not applicable

For peer review only

Supplementary Appendix 4

Co-enrolment to other randomised controlled trials

For peer review only

Table S5: Co-enrolment to other randomised clinical trials by treatment arm

Patient characteristic, n(%) unless otherwise specified	Fostamatinib (N=58)	Ruxolitinib (N=64)	Standard of care (N=63)
Co-enrolled in another study pre-randomisation n (%)			
Any randomised study	5 (8.6)	4 (6.3)	5 (7.9)
RECOVERY	3 (5.2)	3 (4.7)	2 (3.2)
REMAP-CAP	1 (1.7)	1 (1.6)	0 (0.0)
NOCoV2	0 (0.0)	0 (0.0)	1 (1.6)
C19-ACS	1 (1.7)	0 (0.0)	2 (3.2)
Co-enrolled in another study post-randomisation n (%)			
Any randomised study)	2 (3.4)	6 (9.4)	7 (11.1)
RECOVERY	1 (1.7)	1 (1.6)	3 (4.8)
REMAP-CAP	1 (1.7)	2 (3.1)	2 (3.2)
NOCoV2	0 (0.0)	2 (3.1)	2 (3.2)
C19-ACS	0 (0.0)	1 (1.6)	1 (1.6)

For peer review only

Supplementary Appendix 5

Missing data methods for the primary model and results using alternative assumptions

For peer review only

In the primary analysis model, we imputed missing WHO COVID-19 status using multiple imputation under the assumption of missing at random (MAR). That is the probability of being missing is not dependent on the values of unobserved data, conditional on the observed values of the variables included in the analysis model. We imputed *m* datasets using chained equations based on the percentage of participants with incomplete outcomes, where *m* was rounded to the nearest 10.[11] As outcome data were classified as missing in 6% of patients, an m=10 was used.

Variables in the imputation model included baseline WHO COVID-19 grade (3 or 4), age (<65 or ≥65), receipt of systemic corticosteroid and/or receipt of an IL6 inhibitor (at the time of or prior to randomisation into MATIS) and prior COVID vaccination. Auxiliary variables suspected to be associated with the outcome and to be predictors of missingness included receipt of any effective COVID-19 treatments (steroids or IL6 inhibitors) post-randomisation (>1 day post-randomisation) and WHO COVID-19 severity grades at day 1 and day 7.

Convergence issues were encountered when running the imputation model separately by treatment arm (the preferred approach).[11] Missing primary outcomes were therefore imputed using the full dataset with treatment arm included as an additional variable. The final analysis mode; was run omitting prior use of steroids as a covariate due to instability results from zero event counts within strata.

Alternative missing data assumptions were also considered: 1) all participants with missing outcome had poor outcome e.g. all missing >=5, and 2): that all participants with missing outcome had good outcome e.g. all missing <5 were also conducted (Table S5).

For peer review only

Table S6: Primary Outcome Statistical Model Results with Alternative Missingness Assumptions

Grade >=5 by Day 14	Adjusted odds ratio (95% CI; p-value[a])	
	Fostamatinib vs SOC	Ruxolitinib vs SOC
Sensitivity analyses		
Assume all missing have poor outcome	1.08 (0.49 to 2.40); 0.847	0.73 (0.32 to 1.67); 0.453
Assume all missing have good outcome	1.17 (0.51 to 2.69); 0.710	0.63 (0.26 to 1.57); 0.323

[a] p-values are two-sided

[b] adjusted for baseline COVID severity, age category, use of steroid (where model allows), use of IL6 inhibitor and prior COVID vaccination

For peer review only

Supplementary Appendix 6

Secondary Outcomes

For peer review only

Table S7: Secondary outcomes: Grade >=5 by Day 28

	N	n (%)	Adjusted odds ratio[a] (95% CI)
**Grade >=5 by Day 28[b]**			
Fostamatinib	58	16 (27.6)	1.17 (0.51 to 2.69)
Ruxolitinib	62	11 (17.7)	0.70 (0.29 to 1.72)
SOC	61	15 (24.6)	REF

[a] adjusted for baseline COVID severity, age category, use of IL6 inhibitor and prior COVID vaccination

[b] multiple imputation used to input missing outcome status for 4 patients in each arm

For peer review only

Table S8: Secondary outcomes up to Day 14

	N	n (%)	Adjusted odds ratio* (95% CI)
**Individual components of modified WHO scale**			
O2 saturation < 90% on ≥ 60% inspired oxygen			
Fostamatinib	55	16 (29.1)	1.84 (0.75 to 4.56)
Ruxolitinib	58	9 (15.5)	0.87 (0.32 to 2.37)
SOC	57	11 (19.3)	REF
Non-invasive ventilation			
Fostamatinib	55	13 (23.6)	0.95 (0.39 to 2.28)
Ruxolitinib	58	9 (15.5)	0.61 (0.24 to 1.56)
SOC	57	14 (24.6)	REF
Mechanical ventilation			
Fostamatinib	55	4 (7.3)	2.01 (0.35 to 11.66)
Ruxolitinib	58	3 (5.2)	1.58 (0.25 to 10.09)
SOC	57	2 (3.5)	REF
Mechanical ventilation with organ support			
Fostamatinib	54	3 (5.6)	3.02 (0.30 to 30.71)
Ruxolitinib	58	2 (3.4)	2.00 (0.17 to 23.44)
SOC	57	1 (1.8)	REF
Death			
Fostamatinib	55	4 (7.4)	1.60 (0.32 to 8.01)
Ruxolitinib	58	0 (0.0)	Not estimable
SOC	57	3 (5.5)	REF
**Maximum severity grade by Day 14**			
Grade 5			
Fostamatinib	55	1 (1.8)	Not estimable
Ruxolitinib	58	1 (1.7)	Not estimable
SOC	57	0 (0.0)	REF
Grade 6			
Fostamatinib	55	7 (12.7)	0.60 (0.21 to 1.69)
Ruxolitinib	58	6 (10.3)	0.52 (0.17 to 1.54)
SOC	57	11 (19.3)	REF
Grade 7			
Fostamatinib	55	1 (1.8)	0.95 (0.06 to 16.04)
Ruxolitinib	58	1 (1.7)	1.16 (0.07 to 19.73)
SOC	57	1 (1.8)	REF
Grade 8			
Fostamatinib	55	3 (5.5)	Not estimable
Ruxolitinib	58	2 (3.4)	Not estimable
SOC	57	0 (0.0)	REF
Grade 9			
Fostamatinib	55	4 (7.3)	1.60 (0.32 to 8.01)
Ruxolitinib	58	0 (0.0)	Not estimable
SOC	57	3 (5.3)	REF

*adjusted for baseline COVID severity, age category, use of IL6 inhibitor and prior COVID vaccination

For peer review only

Table S9: Individual components of modified WHO scale up to Day 28

Component of modified WHO scale	N	n (%)	Adjusted odds ratio[a] (95% CI)
O2 saturation < 90% on ≥ 60% inspired oxygen			
Fostamatinib	54	16 (29.6)	1.94 (0.78 to 4.82)
Ruxolitinib	58	10 (17.2)	0.97 (0.37 to 2.58)
SOC	57	11 (19.3)	REF
Non-invasive ventilation			
Fostamatinib	54	13 (24.1)	0.99 (0.41 to 2.37)
Ruxolitinib	58	10 (17.2)	0.68 (0.27 to 1.70)
SOC	57	14 (24.6)	REF
Mechanical ventilation			
Fostamatinib	54	4 (7.4)	1.95 (0.33 to 11.32)
Ruxolitinib	58	3 (5.2)	1.51 (0.24 to 9.71)
SOC	55	2 (3.6)	REF
Mechanical ventilation with organ support			
Fostamatinib	53	3 (5.7)	2.95 (0.29 to 30.05)
Ruxolitinib	57	3 (5.3)	3.14 (0.31 to 32.22)
SOC	55	1 (1.8)	REF
Death			
Fostamatinib	54	5 (9.3)	2.44 (0.51 to 11.71)
Ruxolitinib	58	2 (3.4)	0.67 (0.10 to 4.37);
SOC	55	3 (5.5)	REF

[a] adjusted for baseline COVID severity, age category, use of IL6 inhibitor and prior COVID vaccination

[b] multiple imputation used to input missing outcome status for 4 patients in each arm

For peer review only

Table S10: Maximum severity grade by Day 28

Maximum severity grade by Day 28	N	n (%)	Adjusted odds ratio[a] (95% CI)
Grade 5			
Fostamatinib	54	1 (1.9)	Not estimable
Ruxolitinib	58	1 (1.7)	Not estimable
SOC	55	0 (0.0)	REF
Grade 6			
Fostamatinib	54	7 (13.0)	0.76 (0.26 to 2.27)
Ruxolitinib	58	6 (10.3)	0.66 (0.21 to 2.04)
SOC	55	9 (16.4)	REF
Grade 7			
Fostamatinib	54	1 (1.9)	0.90 (0.05 to 15.48)
Ruxolitinib	58	0 (0.0)	Not estimable
SOC	55	1 (1.8)	REF
Grade 8			
Fostamatinib	54	2 (3.7)	Not estimable
Ruxolitinib	58	2 (3.4)	Not estimable
SOC	55	0 (0.0)	REF
Grade 9			
Fostamatinib	54	5 (9.3)	2.44 (0.51 to 11.71)
Ruxolitinib	58	2 (3.4)	0.67 (0.10 to 4.37)
SOC	55	3 (5.5)	REF

[a] adjusted for baseline COVID severity, age category, use of IL6 inhibitor and prior COVID vaccination

[b] multiple imputation used to input missing outcome status for 4 patients in each arm

Table S11: Change in odds of progressing to a worse severity between trial arms

Outcome definition	Adjusted[a] odds ratio (95% CI two-sided p-value)	
	Fostamatinib vs SOC	Ruxolitinib vs SOC
COVID severity at each time point (N=180[b])		
Day 1	1.00 (0.35 to 2.82); 0.994	0.68 (0.25 to 1.91); 0.469
Day 7	0.94 (0.31 to 2.89); 0.915	0.51 (0.16 to 1.57); 0.239
Day 14	2.37 (0.67 to 8.39); 0.182	0.77 (0.21 to 2.78); 0.689

[a] Adjusted for age category, baseline severity, use of steroid at baseline, use of IL6 inhibitor at baseline, received covid vaccine

[b] Includes N=180 ie all patients with at least one post-baseline outcome measurement

For peer review only

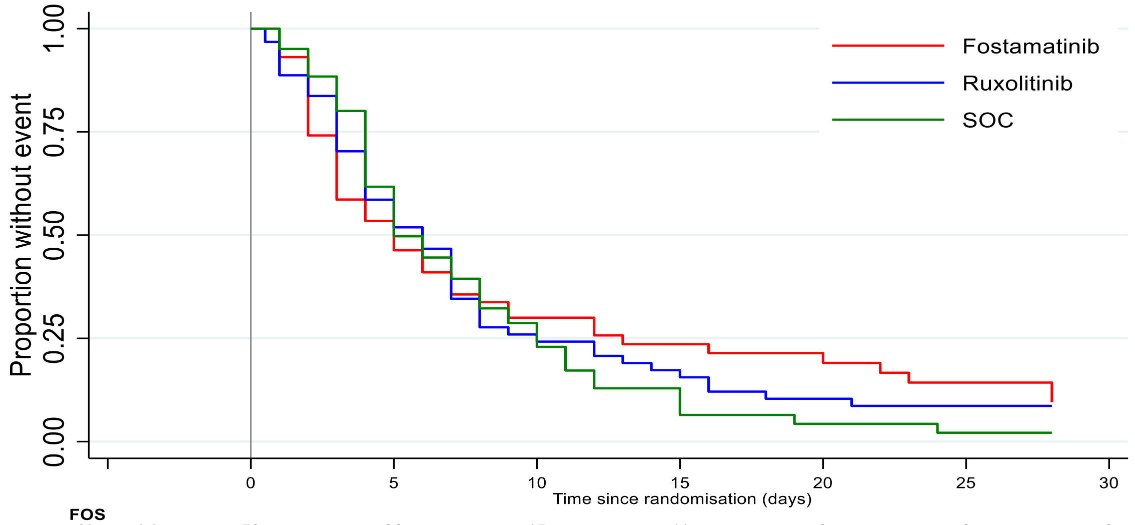

	0	5	10	15	20	25	30
**FOS**							
No. at-risk	58	30	15	11	9	6	0
Censored	0	1	3	4	5	5	9
Discharged	0	27	40	43	44	47	49
**RUX**							
No. at-risk	62	35	15	10	6	4	0
Censored	0	2	3	3	3	4	8
Discharged	0	25	44	49	53	54	54
**SOC**							
No. at-risk	61	36	15	6	2	1	0
Censored	0	2	4	5	5	5	6
Discharged	0	23	42	50	54	55	55

Figure S1: Kaplan Meier estimates of time to discharge (from randomisation) by treatment arm, censored at day 28[a]

[a] withdrawals and deaths are censored at the date of occurrence

Table S12: Readmissions by Day 28

Trial arm	N	n (%)	Adjusted odds ratio[a] (95% CI)
Fostamatinib	54	3 (5.6)	Not estimated
Ruxolitinib	58	8 (13.8)	Not estimated
SOC	55	8 (14.6)	Not estimated

Table S13: Results from mixed effects linear regression for inflammatory markers

Marker	Fostamatinib vs SOC		Ruxolitinib vs SOC	
	Adjusted[a] mean difference	95% CI two-sided p-value	Adjusted[a] mean difference	95% CI two-sided p-value
**CRP (mg/L)**				
Day 1	-6.2	-22.4 to 10.0; 0.452	-7.7	-23.8 to 8.4; 0.350
Day 7	9.4	-8.2 to 27.0; 0.294	-11.9	-29.1 to 5.3; 0.176
Day 14	18.2	-6.1 to 42.5; 0.141	-6.8	-30.0 to16.3; 0.562
Day 28	3.7	-19.7 to 27.2; 0.755	-18.3	-40.5 to 3.8; 0.105
**LDH (IU/L)**				
Day 1	-11.8	-81.2 to 57.6; 0.739	-33.0	-106.7 to 40.7; 0.380
Day 7	39.5	-31.9 to 110.8; 0.278	56.6	-17.9 to 131.2; 0.136
Day 14	9.6	-84.5 to 103.7; 0.841	-34.7	-137.3 to 67.8; 0.507
Day 28	97.3	3.7 to 190.8; 0.042	54.3	-42.8 to 151.4; 0.273
**Ferritin (ug/L)**				
Day 1	278	-447 to 1003; 0.452	-101	-826 to 624; 0.784
Day 7	227	-487 to 941; 0.533	35	-668 to 739; 0.921
Day 14	29	-701 to 759; 0.938	-403	-1121 to 316; 0.272
Day 28	-20	-782 to 742; 0.959	-357	-1103 to 388; 0.347
**D-dimer (ng/ml)**				
Day 1	-360	-1191 to 471; 0.396	-97	-964 to 770; 0.826
Day 7	-487	-1325 to 351; 0.255	22	-808 to 853; 0.958
Day 14	85	-891 to 1062; 0.864	80	-864 to 1023; 0.869
Day 28	615	-388 to 1619; 0.229	401	-568 to 1370; 0.417
**Creatinine (µmol/L)[b]**				
Day 1	-10.1	-29.8 to 9.7; 0.317	-10.1	-29.7 to 9.5; 0.312
Day 7	12.3	-8.9 to 33.5; 0.255	16.1	-4.9 to 37.2; 0.133
Day 14	34.3	6.8 to 61.8; 0.014	21.2	-5.4 to 47.9; 0.119
Day 28	43.6	15.7 to 71.5; 0.002	27.2	0.6 to 53.7; 0.045

[a] adjusted for baseline value, site, use of steroid at baseline, use of IL6 inhibitor at baseline, age category and receipt of COVID vaccine; [b] analysis excludes three patients in RUX arm receiving dialysis

For peer review only

Supplementary Appendix 7

Exploratory analyses

For peer review only

Table S14: Primary outcome model results in patients who received ≥ 90% of prescribed dose

Per-protocol	No. (%) of compliers	Adjusted[a] odds ratio (95% CI; p-value[b])	
		ITT	Per-protocol
Fostamatinib	40 (69.0)	1.19 (0.51 to 2.76); 0.681	1.19 (0.47 to 2.99); 0.716
Ruxolitinib	46 (74.2)	0.63 (0.25 to 1.57); 0.323	0.80 (0.31 to 2.06); 0.641
SOC	61 (100.0)	REF	REF
**CACE**	**No. (%) of compliers**	**Adjusted[a] risk difference (95% CI; p-value[b])**	
		**ITT**	**CACE**
Fostamatinib	40 (69.0)	0.03 (-0.12 to 0.17); 0.729	0.05 (-0.18 to 0.28); 0.642
Ruxolitinib	46 (74.2)	-0.06 (-0.17 to 0.06); 0.328	-0.10 (-0.29 to 0.10); 0.328
SOC	61 (100.0)	REF	REF

[a] adjusted for baseline COVID severity, age category, use of IL6 inhibitor, prior COVID vaccination; model run on imputed dataset

[b] p-values are two-sided

Table S15: Primary outcome model results stratified by time of randomisation

Grade >=5 by Day 14	N			Odds ratio (95% CI; p-value[a])	
	FOS	RUX	SOC	Fostamatinib vs SOC	Ruxolitinib vs SOC
Stratified analysis by time of enrolment					
Quartile 1 (Jan 2021)	16	15	15	0.45 (0.10 to 2.01; 0.299)	0.44 (0.09 to 2.15; 0.313)
Quartile 2 (Feb 2021)	15	16	14	4.71 (0.73 to 30.28; 0.102)	1.50 (0.21 to 10.81; 0.687)
Quartile 3 (Sep 2021)	15	15	15	Not estimable	0.92 (0.15 to 5.53; 0.924)
Quartile 4 (Aug 2022)	12	16	17	3.10 (0.56 to 16.96; 0.193)	Not estimable

a p-values are two-sided

Supplementary Appendix 8

Adverse Events by System Organ Class

For peer review only

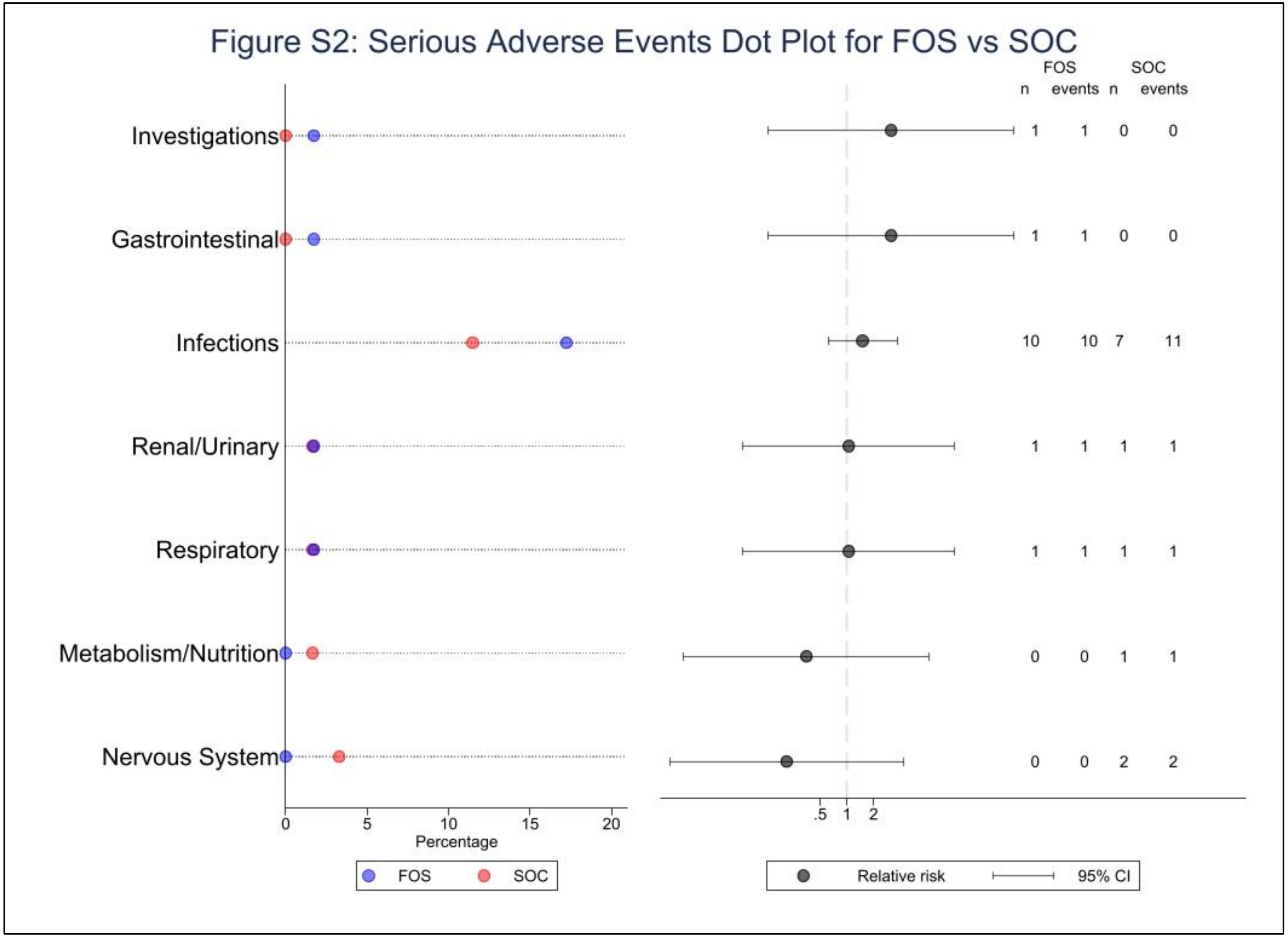
Figure S2: Serious Adverse Events Dot Plot for FOS vs SOC
FOS
SOC
n
events
n
events
Investigations
1 1 0 0
Gastrointestinal
1 1 0 0
Infections
10 10 7 11
Renal/Urinary
1 1 1 1
Respiratory
1 1 1 1
Metabolism/Nutrition
0 0 1 1
Nervous System
0 0 2 2
0 5 10 15 20
Percentage
.5 1 2
FOS
SOC
Relative risk
95% CI

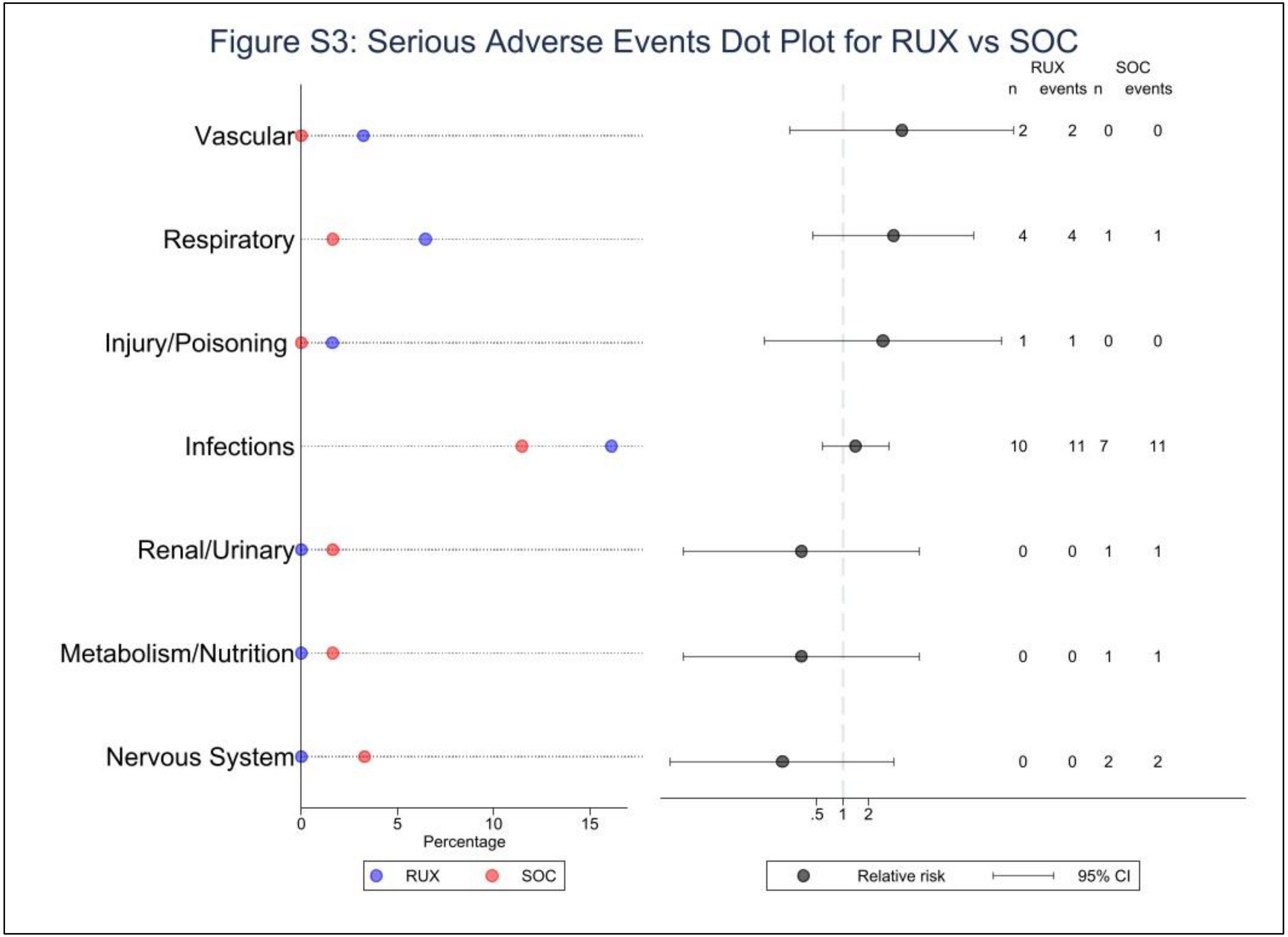
Figure S3: Serious Adverse Events Dot Plot for RUX vs SOC
RUX
SOC
n
events
n
events
Vascular
2 2 0 0
Respiratory
4 4 1 1
Injury/Poisoning
1 1 0 0
Infections
10 11 7 11
Renal/Urinary
0 0 1 1
Metabolism/Nutrition
0 0 1 1
Nervous System
0 0 2 2
0
5
10
15
Percentage
.5
1
2
RUX
SOC
Relative risk
95% CI

Supplementary Appendix 9

Subgroup Analysis

For peer review only

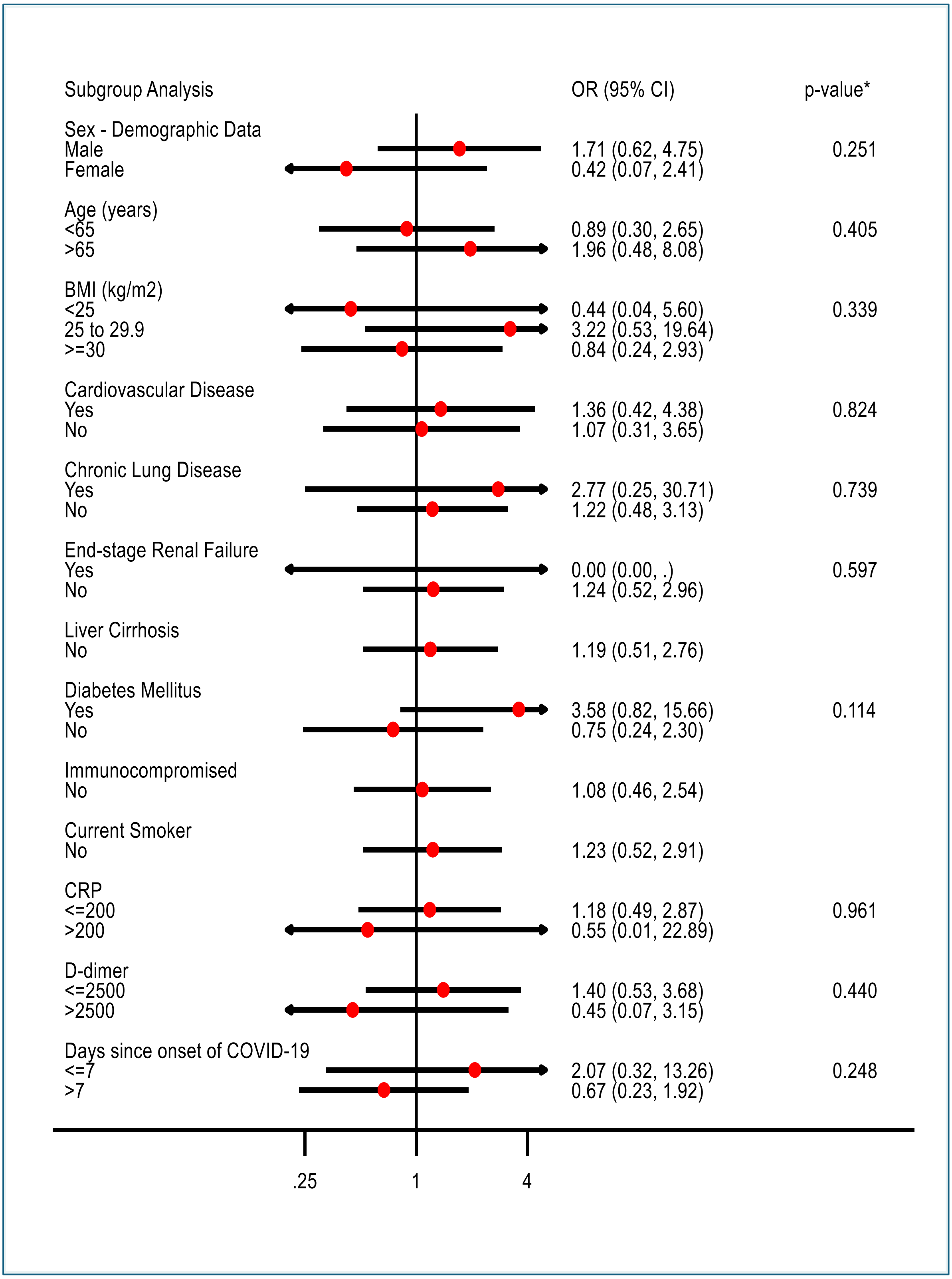

*p value for test of interaction

Figure S4: Subgroup analysis of primary outcome for fostamatinib versus SOC

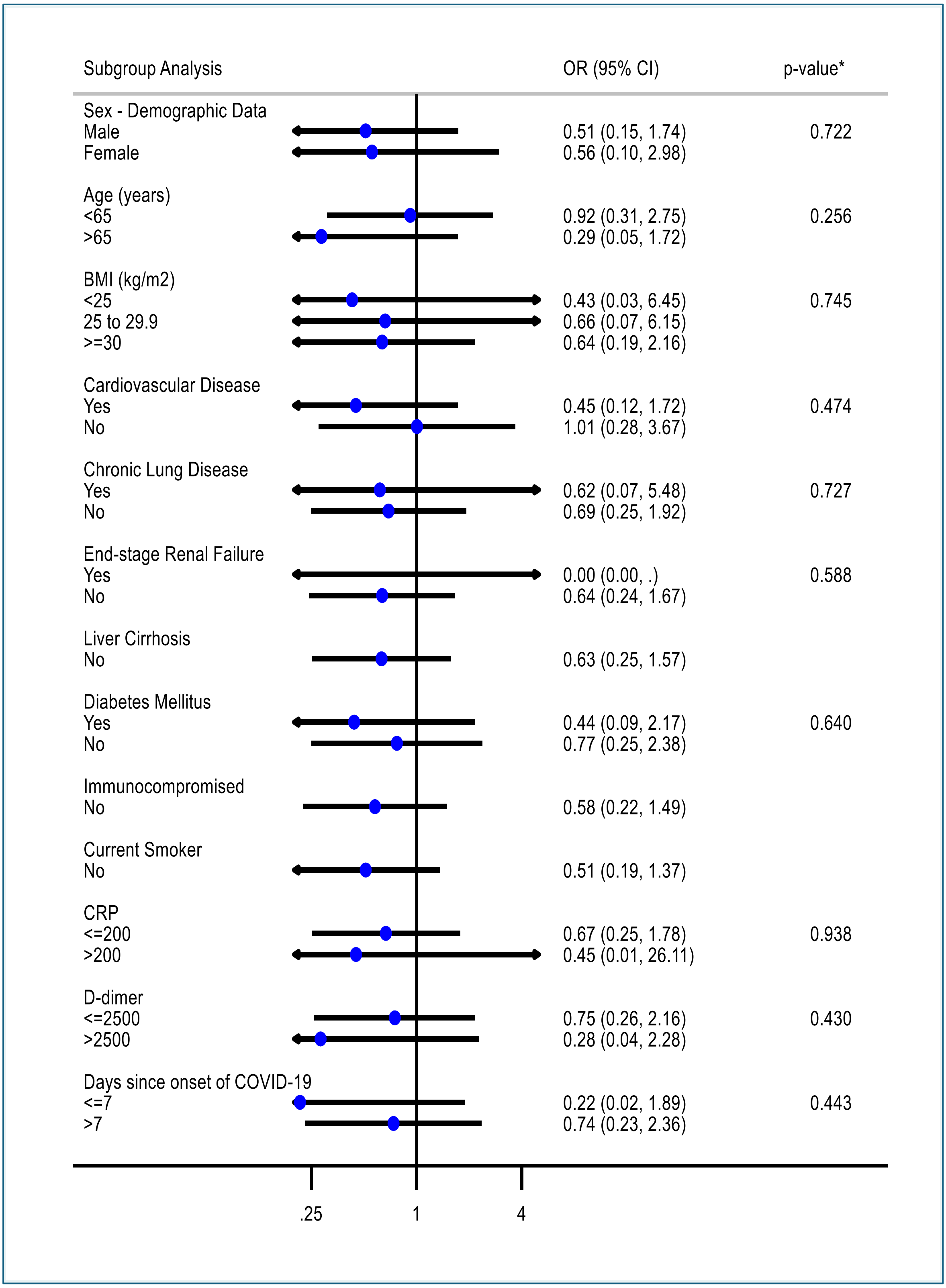

*p value for test of interaction

Figure S4: Subgroup analysis of primary outcome for ruxolitinib versus SOC

BMJ Open

Multi-arm multi-stage randomised controlled trial of Inflammatory Signal Inhibitors (MATIS) for patients hospitalised with COVID-19 pneumonia during the UK pandemic.

Journal:	*BMJ Open*
Manuscript ID	bmjopen-2025-100583.R2
Article Type:	Original research
Date Submitted by the Author:	12-Dec-2025
Complete List of Authors:	Hazell, Lorna; Imperial College London, Imperial College Clinical Trials Unit<br>Pillay, Clio; Imperial College London, Department of Immunology and Inflammation; Imperial College Healthcare NHS Trust<br>Cornelius, Victoria; Imperial College London, Imperial College Clinical Trials Unit<br>Phillips, Rachel; Imperial College London, Imperial College Clinical Trials Unit<br>Charania, Asad; Imperial College London, Department of Immunology and Inflammation; Imperial College Healthcare NHS Trust<br>Wason, James; Newcastle University, Population Health Sciences Institute<br>Cherlin, Svetlana; Newcastle University, Population Health Sciences Institute<br>Savic, Sinisa; Leeds Teaching Hospitals NHS Trust, Immunology; University of Leeds,<br>Whittington, Ashley; Northwick Park Hospital, Infectious Diseases and Acute Medicine<br>Neelakantan, Pratap; Royal Berkshire NHS Foundation Trust, Haemato-Oncology<br>Collini, Paul; Sheffield Teaching Hospitals NHS Foundation Trust, Department of Infectious Diseases<br>Cook, Lucy; Imperial College London, Department of Immunology and Inflammation; Imperial College Healthcare NHS Trust<br>Willicome, Michelle; Imperial College London, Department of Immunology and Inflammation; Imperial College Healthcare NHS Trust<br>Milojkovic, Dragana; Imperial College London, Department of Immunology and Inflammation; Imperial College Healthcare NHS Trust<br>Kon, Onn Min; Imperial College Healthcare NHS Trust, Respiratory Medicine; Imperial College London, National Heart and Lung Institute<br>Youngstein, Taryn; Imperial College London, National Heart and Lung Institute; Imperial College Healthcare NHS Trust<br>Innes, Andrew; Imperial College London, Department of Immunology and Inflammation; Imperial College Healthcare NHS Trust<br>Thursz, Mark; Imperial College London, Department of Metabolism, Digestion and Reproduction; Imperial College Healthcare NHS Trust<br>Cooke, Graham; Imperial College London, Department of Infectious Diseases; Imperial College Healthcare NHS Trust<br>Vergis, Nikhil; Imperial College London, Department of Immunology and

	Inflammation; Imperial College Healthcare NHS Trust<br>Cooper, Nichola; Imperial College London, Department of Immunology and Inflammation; Imperial College Healthcare NHS Trust

**Primary Subject Heading**:	Immunology (including allergy)
Secondary Subject Heading:	Infectious diseases, Intensive care
Keywords:	Clinical Trial, COVID-19, Drug Therapy

SCHOLARONE™
Manuscripts

I, the Submitting Author has the right to grant and does grant on behalf of all authors of the Work (as defined in the below author licence), an exclusive licence and/or a non-exclusive licence for contributions from authors who are: i) UK Crown employees; ii) where BMJ has agreed a CC-BY licence shall apply, and/or iii) in accordance with the terms applicable for US Federal Government officers or employees acting as part of their official duties; on a worldwide, perpetual, irrevocable, royalty-free basis to BMJ Publishing Group Ltd ("BMJ") its licensees and where the relevant Journal is co-owned by BMJ to the co-owners of the Journal, to publish the Work in this journal and any other BMJ products and to exploit all rights, as set out in our licence.

The Submitting Author accepts and understands that any supply made under these terms is made by BMJ to the Submitting Author unless you are acting as an employee on behalf of your employer or a postgraduate student of an affiliated institution which is paying any applicable article publishing charge ("APC") for Open Access articles. Where the Submitting Author wishes to make the Work available on an Open Access basis (and intends to pay the relevant APC), the terms of reuse of such Open Access shall be governed by a Creative Commons licence – details of these licences and which Creative Commons licence will apply to this Work are set out in our licence referred to above.

Other than as permitted in any relevant BMJ Author's Self Archiving Policies, I confirm this Work has not been accepted for publication elsewhere, is not being considered for publication elsewhere and does not duplicate material already published. I confirm all authors consent to publication of this Work and authorise the granting of this licence.

For peer review only

CONFIDENTIAL PRE-PUBLICATION MANUSCRIPT

TITLE: Multi-arm multi-stage randomised controlled trial of Inflammatory Signal Inhibitors (MATIS) for patients hospitalised with COVID-19 pneumonia during the UK pandemic.

Authors:

Lorna Hazell, Trial Statistician (0000-0002-5962-0648) * [1]

Dr Clio Pillay, Trial Manager* [2,3]

Professor Victoria Cornelius, Professor in Medical Statistics and Trials Methodology (0000-0002-0080-1065) [1]

Dr Rachel Phillips, Associate Professor in Medical Statistics and Clinical Trials (0000-0002-3634-7845) [1]

Dr Asad Charania (0000-0001-8908-4535) [2,3]

Professor James M. S. Wason, Professor of Biostatistics (0000-0002-4691-126X) [4]

Dr Svetlana Cherlin, Senior Research Associate (0000-0001-9689-3699) [4]

Professor Sinisa Savic, Professor of Clinical Immunology (0000-0001-7910-0554) [5]

Dr Ashley Whittington, Consultant in Infectious Diseases and Acute Medicine [6]

Dr Pratap Neelakantan, Consultant Haematologist [7]

Dr Paul Collini, Senior Clinical Lecturer in Infectious Diseases (0000-0001-6696-6826) [8]

Dr Lucy Cook, Consultant Haematologist (0000-0002-8234-5090) [2,3]

Dr Michelle Willicome, Clinical Reader in Transplantation [2,3]

Dr Dragana Milojkovic, Consultant Haematologist [2,3]

Professor Onn Min Kon, Respiratory Consultant (0000-0003-2647-4688) [3,9]

Dr Taryn Youngstein, Consultant Rheumatologist (0000-0002-8297-556X) [3,9]

Dr Andrew J. Innes, Honorary Clinical Senior Lecturer (0000-0003-0918-8882) [2,3]

For peer review only - http://bmjopen.bmj.com/site/about/guidelines.xhtml

CONFIDENTIAL PRE-PUBLICATION MANUSCRIPT

Professor Mark Thurz, Director of Academic Health Science Centre (0000-0002-8218-192X) [3,10]

Professor Graham S. Cooke, Deputy Dean of the Faculty of Medicine (0000-0001-6475-5056) [3,11]

Dr Nikhil Vergis, Honorary Clinical Senior Lecturer (0000-0002-4157-6757) [$ 2,3]

Professor Nichola Cooper, Professor of Immune Haematology (0000-0002-9845-8292) [$ 2,3]

Corresponding author:

Nichola Cooper, n.cooper@imperial.ac.uk

[*] Both authors contributed equally to this work.

[$] Both authors contributed equally to this work.

For peer review only

For peer review only - http://bmjopen.bmj.com/site/about/guidelines.xhtml

CONFIDENTIAL PRE-PUBLICATION MANUSCRIPT

Author Affiliations

1. Imperial Clinical Trials Unit, Imperial College London, UK
2. Department of Immunology and Inflammation, Imperial College London, UK
3. Imperial College NHS Healthcare Trust, London, UK.
4. Population Health Sciences Institute, Newcastle University, Newcastle upon Tyne, UK
5. Clinical Immunology and Allergy, Leeds Teaching Hospitals NHS Trust, Leeds, UK.
6. Infectious Diseases and Acute Medicine, Northwick Park Hospital, London, UK
7. Department of Haematology, Royal Berkshire Hospital, Reading, UK
8. Department of Infectious Diseases, Sheffield Teaching Hospitals NHS Foundation Trust, Sheffield, UK
9. National Heart and Lung Institute, Imperial College London, UK
10. Department of Metabolism, Digestion and Reproduction, Imperial College London, UK
11. Department of Infectious Diseases, Imperial College London, UK

For peer review only

Copyright/license for publication

The Corresponding Author has the right to grant on behalf of all authors and does grant on behalf of all authors, an exclusive licence (or non-exclusive for government employees) on a worldwide basis to the BMJ Publishing Group Ltd to permit this article (if accepted) to be published in BMJ editions and any other BMJPGL products and sublicences such use and exploit all subsidiary rights, as set out in our licence.

For peer review only

Competing interest statement

All authors have completed the Unified Competing Interest form (available on request from the corresponding author). LH, CP, RP, AW, PN, PC, LC, MT and NV declare no support from any organisation for the submitted work; no financial relationships with any organisations that might have an interest in the submitted work in the previous three years and no other relationships or activities that could appear to have influenced the submitted work. VC is a co-investigator leading statistical design, methods, and analysis for the MATIS trial, funded by Novartis and Rigel, is a committee member for the NIHR Academy Doctoral board, a DMEC member for the NIHR HTA-funded BAY Project. Behavioural Activation for Young people with depression in specialist child and adolescent mental health services and is chair of the DMC for NIHR HTA-funded RADAR: Research into Antipsychotic Discontinuation and Reductions. AC has been an employee of Roche since 2022. DM has received honorary payments from Novartis, Incyte and Ascentage Pharma, has participated on an advisory board for Ascentage Pharma and has received investigator-led institutional research funding from Incyte. MW has received honorary payments from AstraZeneca, has participated on an advisory board or data monitoring committee for

CONFIDENTIAL PRE-PUBLICATION MANUSCRIPT

Hansa Pharmaceuticals, received equipment, materials, drugs, medical writing, gifts or other services from Moderna and has had a leadership or fiduciary role in other board, society, committee or advocacy group, paid or unpaid for Hansa Pharmaceuticals. OMK has participated on an advisory board or data monitoring committee for the NIHR DILI study and has had team members funded by grants from Cepheid. GC has participated in an end point review committee for Sanofi and is a board member for the MHRA. SS holds the Chair of British Society for Immunology-Clinical immunology professional network. JW and SC have had team members funded by grants from Novartis and Rigel. TY is recipient of a grant from UKRI MRC BHF Cardio-IMID Partnership, has received consulting fees for the Ad Board Abbvie 2024 and is Treasurer for the UK and Ireland Vasculitis and Rare Disease Group. AJI has received consulting fees, honoraria for speakers' bureaus, international travel support and/or personal fees from Novartis and Incyte. NC has received research grants from Novartis and Rigel for conducting MATIS, honoraria for consultancy from Novartis, Sobi, Sanofi, Amgen, Argenyx, research support from Novartis and Argenx and has funding from the NIHR Imperial Biomedical Research Centre.

For peer review only

Contributorship and guarantor information

Contributors: NC conceived the research idea and led the trial. NC attained funding for the trial. NC, CP, SS, AC, AW, PN, PC, LC, MW, DM, OMK, TY, AJI, MT, GC and NV were principal investigators or investigators and contributed to the inception, trial

For peer review only - http://bmjopen.bmj.com/site/about/guidelines.xhtml

management, data collection and/or completion of the trial. RP, JW, VC and NC were responsible for the study design and statistical analysis plan. LH, SC and RP conducted the statistical analysis. LH wrote the first draft of the manuscript with input from RP, VC, CP and NC. All authors approved of the submitted version.

The corresponding author (NC) attests that all listed authors meet authorship criteria and that no others meeting the criteria have been omitted. NC, as guarantor, accepts full responsibility for the work and/or the conduct of the study, had access to the data, and controlled the decision to publish.

Transparency declaration

NC affirms that the manuscript is an honest, accurate, and transparent account of the study being reported; that no important aspects of the study have been omitted; and that any discrepancies from the study as originally planned (and registered) have been explained.

Role of the funding source

The MATIS trial was sponsored by Imperial College London and was jointly funded by NIHR Imperial Biomedical Research Centre, Novartis, the manufacturer of ruxolitinib (Jakavi®) and Rigel, the manufacturer of fostamatinib (Tavlesse®).

For peer review only - http://bmjopen.bmj.com/site/about/guidelines.xhtml

The sponsor and funders had no control over the study design; data collection, analysis, or interpretation of data; in the writing of the report; or in the decision to submit the article for publication. All authors confirm independence from the funders, had full access to all of the data (including statistical reports and tables) and take responsibility for the integrity of the data and the accuracy of the data analysis.

Trial registration details

The MATIS trial was registered prospectively in ClinicalTrials.gov (NCT04581954 and EUDRA-CT (2020-001750-22) before enrolling the first participant. The EUDRA-CT record was frozen on 1st January 2021 following the UK exit from the European Union and as such may not contain details of any trial protocol amendments or corrections after this date.

Abstract

Objectives: To determine the safety and efficacy of ruxolitinib and fostamatinib compared to standard of care (SOC) in patients requiring hospital admission for the treatment of COVID-19 pneumonia.

Design: Adaptive multi-arm, multi-stage, randomised, open label trial (3-arm, 2-stage)

Setting: Five hospitals in England between October 2020 and September 2022.

For peer review only - http://bmjopen.bmj.com/site/about/guidelines.xhtml

Participants: Hospitalised patients (≥18 years) with COVID-19 pneumonia defined by a modified World Health Organisation (WHO) COVID-19 severity grade of 3 or 4.

Interventions: Participants were randomly assigned 1:1:1 to receive ruxolitinib (10mg bd for 7 days then 5mg bd for 7 days), fostamatinib (150mg bd for 7 days then 100mg bd for 7 days) or SOC.

Main outcome measures: Primary outcome was development of severe COVID-19 pneumonia (modified WHO severity grade ≥5) within 14 days of randomisation. Secondary outcomes included mortality, invasive and non-invasive ventilation, venous thromboembolism, duration of hospital stay, readmissions, inflammatory markers and serious adverse events (SAE).

Results: At Stage 1, 181 patients were randomised, with 4 assessed as ineligible post-randomisation. Fostamatinib was stopped early for futility with 16 participants (27.6%, N=58) developing severe COVID-19 pneumonia compared to 15 (25.0%, N=60) in the SOC arm (adjusted odds ratio (aOR) compared to SOC: 1.12; 95% confidence interval (CI): 0.49 to 2.58; p=0.608). Ruxolitinib progressed to Stage 2 but the trial was stopped early due to slow recruitment. At the final analysis, 10 participants (16.1%, N=62) developed severe COVID-19 pneumonia in the ruxolitinib arm compared to 15 (24.6%, N=61) in the SOC arm (aOR: 0.63; 95% CI: 0.25 to 1.57; p=0.161). Four (7.4%) participants in the fostamatinib arm, none in the ruxolitinib arm and three (5.5%) in the SOC arm died within 14 days of randomisation. Infections were the most frequently reported serious adverse event and were numerically higher in the fostamatinib (10, 17.2%) and ruxolitinib (10,

For peer review only - http://bmjopen.bmj.com/site/about/guidelines.xhtml

CONFIDENTIAL PRE-PUBLICATION MANUSCRIPT

16.1%) arms compared to SOC (7, 11.5%). Two unexpected serious adverse reactions occurred in the ruxolitinib arm only.

Conclusions: We found no evidence that fostamatinib was superior to SOC for the treatment of COVID-19 pneumonia in patients requiring hospital admission. Due to early stopping, the trial was underpowered to establish ruxolitinib's effect in this population. Further study is needed.

Trial registration: NCT04581954; EUDRA-CT: 2020-001750-22

Strength and Limitations of the Study

- Participants in the MATIS trial were broadly representative of an urban population requiring hospital admission for the treatment of COVID-19 pneumonia in the UK.
- We used an efficient multi-arm, multi-stage trial design to focus research efforts on more promising treatments in the pandemic setting.
- We were able to determine that fostamatinib was unlikely to be efficacious in this patient population at an early stage of the trial.
- The trial was, however, subsequently stopped early due to slowing recruitment as the pandemic receded and mass vaccination was introduced.

For peer review only - http://bmjopen.bmj.com/site/about/guidelines.xhtml

For peer review only

- We were therefore unable to confirm the efficacy of ruxolitinib in this patient setting.

Introduction

Severe COVID-19 pneumonia is characterised by respiratory and multi-organ failure in the context of marked systemic inflammation and increased thrombotic risks. Elevations in circulating inflammatory molecules are associated with poor prognosis and in a subset of patients, drive acute lung injury which, in the early stages of the 2020 pandemic, resulted in high rates of catastrophic multi-organ failure and death.[1]

Therapeutic interventions targeting inflammatory signalling might reduce the severity of the inflammatory response phase and associated lung damage thereby averting respiratory failure and the need for mechanical ventilation. Data from China and Italy during the pandemic suggested that immediate resolution of symptoms could be achieved using anti-interleukin 6 (IL6) therapy and Janus kinase (JAK) inhibitors in patients with severe disease.[2 3] The spleen tyrosine kinase (SYK) pathway was also considered a potential target; it is activated by several receptors, including C-type lectin (CLEC) or C-type lectin-like receptors (CLR), and Fc receptors.[4] Inhibition of the SYK pathway has been shown to inhibit platelet aggregation in heparin-Induced thrombocytopenia models,[5] a disease with many similar features to COVID-19.

We hypothesised that there may be an early window of opportunity to treat the COVID-19 hyperinflammatory syndrome before acute lung injury leads to organ failure. We designed a randomised controlled, multi-arm multi-stage trial in patients hospitalised with COVID-19 pneumonia of grade 3 or 4 on the modified World Health

For peer review only - http://bmjopen.bmj.com/site/about/guidelines.xhtml

CONFIDENTIAL PRE-PUBLICATION MANUSCRIPT

Organisation (WHO) COVID-19 severity scale to evaluate the effect of early intervention with two inflammatory signal inhibitors which are already licensed for use in other clinical indications: ruxolitinib (JAK inhibitor)[6] and fostamatinib (small molecule SYK inhibitor).[7]

Methods

Trial design and participants

The Multi-Arm Trial of Inflammatory Signal Inhibitors for COVID-19 (MATIS) was an open-label multi-arm, multi-stage randomised (1:1:1) controlled trial (RCT) of ruxolitinib (RUX) and fostamatinib (FOS) for the treatment of COVID-19 pneumonia compared to routine standard of care (SOC) in patients requiring hospital admission.

Inclusion criteria:

Eligible participants were hospitalised patients in the UK aged ≥18 years with clinically suspected or laboratory confirmed SARS-Cov2 infection, radiological change consistent with COVID-19 disease, a C-reactive protein (CRP) level ≥30mg/L at any time point and COVID-19 pneumonia defined by a severity grade 3 or 4 on the modified WHO severity scale (Table 1).[8] The modified scale includes an additional grade to capture clinical deterioration in patients for whom escalation in organ support is not offered. Non-English speakers were eligible to join the study; hospital translation services were provided where appropriate. Female participants of child-bearing potential were required to agree to abstain from sexual intercourse or use effective contraception for 42 days; after the last dose of study medication male participants were required to abstain from sperm donation for 42 days after the last dose of study medication.

For peer review only - http://bmjopen.bmj.com/site/about/guidelines.xhtml

Table 1. Modified WHO COVID-19 Severity Scale, from the WHO R&D Blueprint

Patient state	Descriptor	Grade
Uninfected	No clinical or virological evidence of infection	0
Ambulatory	No limitation of activities	1
	Limitation of activities	2
Hospitalised mild disease	Hospitalised, no oxygen therapy	3
	Oxygen by mask or nasal prongs	4
Hospitalised severe disease	SpO_2< 90% on FiO_2 ≥60% by face mask	5
	Non-invasive ventilation, CPAP or high-flow oxygen	6
	Intubation and mechanical ventilation	7
	Ventilation + additional organ support (vasopressors, RRT, ECMO)	8
Dead	Death	9

Published in the COVID Therapeutic Trial Synopsis, February 18th 2020[8]

CPAP: continuous positive airways pressure; SpO_2: peripheral arterial blood oxygen; FiO_2: fraction of inspired oxygen; RRT: renal replacement therapy; ECMO: extracorporeal membrane oxygenation

Exclusion criteria:

Patients with severity below Grade 3 on the WHO COVID-19 Ordinal Scale (i.e., non-hospitalised patients) were not eligible to participate in the MATIS trial. Patients were also ineligible if they had ≥Grade 5 severity pneumonia or had oxygen saturation levels < 90% on 60% inspired oxygen at baseline or if, in the opinion of the investigator, progression to death was inevitable within the next 24 hours, irrespective of the provision of therapy or if they required either invasive or non-invasive ventilation (NIV) including continuous positive airway pressure (CPAP) or high flow nasal oxygen at any point after hospital admission and before baseline. Patients normally on non-invasive ventilation such as continuous positive airway

For peer review only - http://bmjopen.bmj.com/site/about/guidelines.xhtml

pressure CPAP at home were eligible to take part. Further exclusion criteria were any medical condition/history or concomitant medication that would compromise participants' safety or put the participant at significant risk or compliance with study procedures or would compromise the scientific integrity of the study, any known severe allergic reactions to the investigational agents, use of drugs within the preceding 14 days known to interact with any study treatment, Child Pugh B or C grade hepatic dysfunction, pregnancy and breast feeding.

The trial design (Figure 1) is further described in the trial protocol.[9] A planned formal interim analysis (Stage 1) was conducted on 10th March 2022 after 177 eligible participants had been randomised and completed 14 days of follow-up. A pre-specified threshold of one-sided p-value ≤0.25 was required to progress treatment with RUX and/or FOS to Stage 2.

Insert Figure 1 here

Figure 1: MATIS trial design

Randomisation and blinding

Participants were randomly allocated to RUX, FOS or SOC using a central web-based randomisation service that used randomisation sequences with random block sizes stratified by age (<65 vs ≥65) and study site. Staff treating participants, participant-facing research staff and participants were unblind to study treatment. IDMC reports prior to the Stage 1 analysis were prepared by the trial statistician who was aware only of treatment groups labelled as Group A, B, and C. Stage 1 analysis was performed by an independent, unblind statistician at Newcastle University. The final analyses were undertaken by an unblind trial statistician at Imperial College

For peer review only - http://bmjopen.bmj.com/site/about/guidelines.xhtml

CONFIDENTIAL PRE-PUBLICATION MANUSCRIPT

Clinical Trials Unit. All analyses were undertaken after the Statistical Analysis Plan was signed off.

Interventions

The planned duration of treatment was 14 days. Participants in the RUX arm were prescribed 10mg twice daily for 7 days reducing to 5mg twice daily for a further 7 days. Participants in the FOS arm were prescribed 150mg twice daily for 7 days reducing to 100mg twice daily for a further 7 days. Treatment was given by mouth unless the participant was unable to take oral medicines, in which case treatments were administered via a nasogastric tube. Study treatment was given in combination with SOC. Due to the evolving clinical landscape for the management of COVID-19, the SOC was not predefined and varied during the course of the trial. Co-enrolment to other trials, additional treatment and rescue therapy was permitted at the discretion of the treating clinicians.

Follow up

Participants were followed up at 1, 7, 14 and 28 days after randomisation. Those discharged from hospital before Day 14 completed trial medication at home and were followed up by telephone or using patient health records, where available, up to Day 28.

Outcomes

All comparisons were between RUX or FOS versus SOC. The primary outcome was defined as the proportion of participants with severe COVID-19 pneumonia within 14 days of randomisation, where severe COVID-19 pneumonia was defined by a

For peer review only - http://bmjopen.bmj.com/site/about/guidelines.xhtml

modified WHO COVID-19 Ordinal Grade ≥ 5 (on the 9-point scale, Table 1).[8] This included participants who died (from any cause), required invasive ventilation or NIV (CPAP or high flow oxygen) or had O_2 saturation <90% on ≥60% inspired oxygen within 14 days of randomisation.

Secondary outcomes, examined at Day 14 and Day 28 included all-cause mortality; the proportions of participants requiring non-invasive ventilation (NIV) (including CPAP or high flow nasal oxygen), invasive ventilation and renal replacement therapy (RRT); incidence of venous thromboembolism (VTE); length of hospital stay; change in absolute severity on the modified WHO COVID-19 Ordinal Scale (Grade 3 or 4 to 5,6,7,8,9); inflammatory markers (CRP, lactose dehydrogenase (LDH), ferritin) D-dimer); and serum creatinine levels. Safety outcomes included serious adverse events (SAEs), reactions (SARs) and treatment discontinuations. Non-serious adverse events were not collected. Additional outcomes including readmissions to hospital within 28 days, the proportion of participants with severe pneumonia at Day 28 and separate components of the primary outcome were not specified as such in the trial protocol but were included in the Statistical Analysis Plan (SAP).

A protocol amendment on 2nd November 2022 included the addition of a sub-study to collect further data to evaluate longer-term outcomes. The results from this sub-study will be published separately from the current manuscript.

Sample size

A sample size of 171 (57 per arm) participants at Stage 1 and an additional maximum of 285 (95 per arm) was selected to provide power of 90% (minimum marginal power) with a maximum 5% chance of an intervention arm being recommended when it provides no improvement over control (5% one-sided family-

For peer review only - http://bmjopen.bmj.com/site/about/guidelines.xhtml

wise error rate), inflated by 5% for missing outcome data (full details are described in the trial protocol).[9] This sample size calculation assumed a 50% rate of severe pneumonia in the standard of care arm and a reduction of this risk in an experimental arm to 30% (relative risk, RR, 0.6).

Statistical analysis

Participant flow through the trial was described using a Consolidated Standards of Reporting Trials (CONSORT) flow chart.[10] A scatter plot was used to show the trajectory of participant recruitment over time. Simple descriptive statistics were used to summarise baseline participant characteristics, co-enrolment to other trials and SOC medications received at baseline and post-randomisation.

The modified intention-to-treat (ITT) population included all eligible randomised participants analysed in the treatment arm to which they were allocated to regardless of treatment subsequently received. Participants found to have been randomised in error were excluded from the modified ITT population. Participants were assumed to have received 14 days of treatment unless there was evidence for stopping earlier.

For the primary outcome, we used a generalized linear model with a binomial distribution and logit link function to compare the odds of developing severe COVID-19 pneumonia between RUX or FOS and SOC. At Stage 1, two separate models were used to compare the effects of FOS vs SOC and RUX vs SOC. These models were adjusted for using a propensity score derived from a logistic regression model that included baseline modified WHO COVID-19 severity grade (3 or 4), age (<65 or ≥65), receipt of steroids at baseline and receipt of interleukin-6 (IL6) inhibitors at baseline.

For peer review only - http://bmjopen.bmj.com/site/about/guidelines.xhtml

For the final analysis, we included all three trial arms in a single model. We planned to adjust our final analysis model for site, age category (<65 or ≥65), baseline modified WHO COVID-19 severity grade (3 or 4) and receipt of treatments assumed to be effective prior to randomisation based on current knowledge at that time of the MATIS trial. These effective treatments included dexamethasone, IL-6 inhibitors, selected antivirals and COVID vaccination. As the efficacy of remdesivir was unconfirmed at the time of the trial, Paxlovid® and/or molnupiravir were the only antivirals specified *a-priori*, however, no participants received these agents. As a large proportion of participants had received dexamethasone in each arm, prior steroid use was not included in our final analysis model to avoid instability due to zero cell counts in some strata. Similarly, as there were several sites with very few randomised participants, we excluded study site from the primary analysis model. Thus, our final model adjusted for prior use of IL-6 inhibitors, age category, modified WHO COVID-19 severity grade and receipt of COVID-19 vaccine. These were included as separate covariates in the final model. Multiple imputation was used to account for any missing data such that all eligible randomised participants were included in both the Stage 1 and final analyses (See Supplementary Appendix 5).[11] Treatment effects were reported as odds ratios with corresponding 95% confidence intervals (CI) and one-sided p-values.

The original two-stage design specified that significance would be judged at the 0.027 level (i.e. a two-sided p-value < 0.054 was to be used to indicate a significant treatment effect). However, as the planned sample size was not reached, the p-values presented should not be interpreted with reference to this threshold as the

For peer review only - http://bmjopen.bmj.com/site/about/guidelines.xhtml

trial was significantly underpowered. Interpretation should be focused on the point estimate and 95% CI as a way to assess the evidence of a treatment effect and associated uncertainty, however, this interpretation should be regarded as hypothesis-generating only.

Analysis of secondary outcomes was performed for the final analysis only. Here, the primary analysis model was repeated to compare the odds of developing severe pneumonia by Day 28. This model was also run separately for each individual component of the primary outcome (death, NIV, invasive ventilation with and without organ support and O_2 <90% on ≥60% inspired oxygen) and for the maximum COVID-19 severity grade reached by Day 14 and Day 28. The trial protocol included a secondary objective to compare the proportions of participants requiring invasive ventilation and/or ECMO, however, this was not assessed as no participants received ECMO in MATIS. COVID-19 severity grade was further modelled as an ordinal variable using a mixed ordinal logistic regression model to compare the odds of progression to more severe disease at Day 14. Mixed linear regression models were used to compare changes in inflammatory markers and serum creatinine up to Day 28 and a Cox proportional hazards model was used to compare the rates of hospital discharge between trial arms. The frequencies of renal replacement therapy, venous thromboembolism, readmission within 28 days, SAEs, SARs and treatment discontinuations were summarised descriptively. Dot plots were used to visually display between group differences in SAE frequencies (as risk ratios) grouping events at system organ class level.

For peer review only - http://bmjopen.bmj.com/site/about/guidelines.xhtml

Prespecified sensitivity analyses were undertaken to examine alternative approaches to handling missing data firstly, assuming that all participants with missing outcome had poor outcome e.g., all missing assumed severity grade >=5, and secondly assuming that all participants with missing outcome had good outcome e.g., all missing assumed severity grade <5. A planned sensitivity analysis of the primary outcome adjusting for receipt of effective COVID-19 treatments through co-enrolment to the active arm of another randomised controlled was not performed since such treatments were not found to have been prescribed to trial participants as part of co-enrolment to another trial. Another sensitivity analysis to explore both the direct effect of treatment on the outcome and any indirect effect of receiving known effective treatments as rescue medications was not conducted as all of the participants who received these medications post-randomisation to MATIS received the medication on or after the index date for the primary outcome.

We performed two prespecified exploratory analyses to assess the impact of adherence to treatment on the primary outcome. Firstly, we conducted a naïve per-protocol analysis in participants who had a minimum of 90% of the planned treatment (at least 13 days of active treatment). In the SOC arm, patients were deemed to be 100% adherent to SOC by definition. Patients who died were considered adherent if they had not discontinued trial treatment at the time of death. Secondly, we calculated the treatment effect (as a risk difference) among compliers using a complier adjusted case (CACE) analysis with a two-stage residual inclusion estimator (2SRI) approach[12] and randomisation group as the instrumental variable. Estimates from the CACE analysis were generated from two separate models for each treatment comparison while the original ITT analysis was one model.

For peer review only - http://bmjopen.bmj.com/site/about/guidelines.xhtml

CONFIDENTIAL PRE-PUBLICATION MANUSCRIPT

Additional exploratory analyses were performed to assess the impact of the time of enrolment to the trial. A categorical variable was created by splitting the recruitment period into quartiles such that participants were grouped into approximately four equal sized groups based on time of enrolment into the MATIS study. This variable was included as a covariate in the primary analysis model as a treatment group-by-time interaction term to assess the impact of time of recruitment on the treatment effect estimate. A crude odds ratio was calculated for each quartile. This analysis was performed using the unimputed dataset since the multiple imputation model could not produce the required estimates due to inconsistency in the omitted variables across the different imputed datasets. Subgroup analysis of the primary outcome by age, sex, BMI, cardiovascular disease, chronic lung disease, end-stage-renal failure, liver cirrhosis, diabetes, baseline CRP and D-dimer, immunocompromisation, smoking status and number of days since onset of COVID-19 at randomisation were also performed.

Stage 1 analysis was performed in R (https://www.R-project.org/). All other analysis was undertaken using Stata/IC version 16.1, (StataCorp, College Station, TX, USA). The Statistical Analysis Plan is provided in Supplementary Appendix 1.

Patient and Public Involvement

Information on MATIS trial design was included on clinicaltrial.gov. A twitter page was set up to explain the trial to a wider audience, and the protocol was published in Trials (2021).[9]

Ethics

For peer review only - http://bmjopen.bmj.com/site/about/guidelines.xhtml

The Surrey Research Ethics Committee (REC) and Health Regulator Authority (HRA) reviewed and granted approval for this trial (IRAS ID 282552). The study was conducted in accordance with the recommendations for physicians involved in research on human subjects adopted by the 18th World Medical Assembly, Helsinki 1964 and later revisions.[13] Informed consent was obtained for each participant either by the participant themselves or from a relative or an independent treating clinician acting as their legally designated personal representative in the event that the participant lacked capacity to provide consent.

For peer review only

Results

Participant flow and baseline characteristics

Between October 2020 and September 2022,185 patients from five UK hospitals in England were enrolled (Figure 2).

Insert Figure 2 here

Figure 2: Participant Flow

The majority of participants were recruited from one London teaching hospital site (n=170) with a smaller proportion from other UK city hospitals (n=15). Recruitment followed the peaks and troughs of the COVID-19 pandemic waves observed in the UK during the trial period (Figure 3). This period predominantly covered the Alpha and Delta waves in the UK.[14] Although the Omicron variant was detected from around December 2021 only 12 participants were recruited to MATIS after that date.

Insert Figure 3 here

Figure 3: Recruitment over time

During the 28-day follow-up, 13 participants withdrew consent and one was lost to follow-up. Four patients found to be ineligible post-randomisation were excluded from the analyses. A slightly higher proportion of participants completed follow up but discontinued study medication early in the FOS arm (13, 22.4%) than in the RUX

For peer review only - http://bmjopen.bmj.com/site/about/guidelines.xhtml

arm (11, 17.2%). Thirty-eight (66.7%) participants in the FOS arm and 46 (71.9%) in the RUX arm were assumed to have completed the 14-day treatment course.

A higher proportion of participants in the RUX arm compared to the SOC arm were female (35.9% vs. 30.2%), were of white ethnicity (48.4% vs. 42.9%), had a modified WHO COVID-19 severity grade of 3 at baseline (21.9% vs. 14.3%) or had received a COVID-19 vaccination (25.0% vs. 17.5%) (Table 2). In contrast, these characteristics were less prevalent in the FOS arm compared to SOC, with the exception of the baseline severity grade of 3 (17.2% in FOS vs. 13.3% in SOC). The percentage of participants with a baseline modified WHO COVID-19 severity grade of 4 decreased over the trial recruitment period from 84.8% in the Alpha wave to 75% for those recruited in the Omicron wave, while the percentage of participants who had received the COVID-19 vaccination increased from 8% to 50% (Supplementary Table S1).

Table 2: Baseline characteristics by treatment arm

Patient characteristic, n (%) unless otherwise specified	Fostamatinib (N=58)	Ruxolitinib (N=64)	Standard of care (N=63)
Age in years			
Mean (SD)	58.9 (15.0)	60.5 (15.8)	59.1 (16.3)
Sex			
Male	43 (74.1)	41 (64.1)	44 (69.8)
Female	15 (25.9)	23 (35.9)	19 (30.2)
Ethnicity			
White	21 (36.2)	31 (48.4)	27 (42.9)
Mixed or multiple ethnic groups	1 (1.7)	0 (0.0)	1 (1.6)
Asian or Asian British	7 (12.1)	11 (17.2)	8 (12.7)
Black, Black British, Caribbean or African	5 (8.6)	7 (10.9)	7 (11.1)
Other ethnic group	24 (41.4)	15 (23.4)	20 (31.7)
BMI in kg/m2			
N (N missing)	55 (3)	57 (7)	60 (3)
Mean (SD)	30.1 (6.5)	30.6 (7.1)	29.9 (6.9)
Modified WHO COVID-19 severity			
Grade 3	10 (17.2)	14 (21.9)	9 (14.3)

For peer review only - http://bmjopen.bmj.com/site/about/guidelines.xhtml

CONFIDENTIAL PRE-PUBLICATION MANUSCRIPT

Patient characteristic, n (%) unless otherwise specified	Fostamatinib (N=58)	Ruxolitinib (N=64)	Standard of care (N=63)
Grade 4	48 (82.8)	50 (78.1)	54 (85.7)
Time from onset of symptoms			
N (N missing)	57 (1)	51 (13)	57 (6)
Mean (SD)	10 (4)	9 (4)	10 (4)
Chronic lung disease			
N (N missing)	58 (0)	62 (2)	63 (0)
Yes	7 (12.1)	12 (19.4)	11 (17.5)
Diabetes			
N (N missing)	58 (0)	62 (2)	63 (0)
Yes	15 (25.9)	19 (30.6)	20 (31.7)
Hypertension			
N (N missing)	58 (0)	62 (2)	63 (0)
Yes	25 (43.1)	28 (45.2)	28 (44.4)
Ischaemic heart disease			
N (N missing)	58 (0)	62 (2)	63 (0)
Yes	11 (19.0)	10 (16.1)	10 (15.9)
Heart failure			
N (N missing)	58 (0)	62 (2)	63 (0)
Yes	2 (3.4)	3 (4.8)	2 (3.2)
Immunocompromised			
N (N missing)	58 (0)	62 (2)	63 (0)
Yes	1 (1.7)	5 (8.1)	2 (3.2)
End-stage renal failure			
N (N missing)	58 (0)	62 (2)	63 (0)
Yes	4 (6.9)	6 (9.7)	3 (4.8)
Liver cirrhosis			
N (N missing)	58 (0)	62 (2)	63 (0)
Yes	0 (0.0)	0 (0.0)	0 (0.0)
Current smoker			
N (N missing)	58 (0)	62 (2)	62 (1)
Yes	0 (0.0)	2 (3.2)	3 (4.8)
Prior Covid-19 Vaccination			
N (N missing)	58 (0)	64 (0)	63 (0)
Yes	8 (13.8)	16 (25.0)	11 (17.5)
Serum creatinine (µmol/L)			
N (N missing)	57 (1)	62 (2)	63 (0)
Mean (SD)	133 (203)	119 (145)	119 (172)
C- reactive protein (mg/L)			
N (N missing)	57 (1)	62 (2)	63 (0)
Mean (SD)	94.1 (61.5)	120.9 (78.4)	105.8 (69.5)
Lactate dehydrogenase (IU/L)			
N (N missing)	36 (22)	37 (27)	36 (27)
Mean (SD)	419 (208)	488 (309)	439.(136)
Ferritin (µg/L)			
N (N missing)	52 (6)	52 (12)	47 (16)
Mean (SD)	1471 (1459)	1671 (3069)	1254 (778)
D-dimer (ng/ml)			

For peer review only - http://bmjopen.bmj.com/site/about/guidelines.xhtml

Patient characteristic, n (%) unless otherwise specified	Fostamatinib (N=58)	Ruxolitinib (N=64)	Standard of care (N=63)
N (N missing)	55 (3)	53 (11)	53 (10)
Mean (SD)	1601 (2749)	1732 (2614)	1341 (2781)

The majority of participants in all three trial arms were receiving anticoagulant therapy at baseline (Table 3), most were receiving an immuno-modulating agent, specifically dexamethasone (Supplementary Table S2). Just under half of participants had received an antiviral agent (Table 3), of whom 86 (98.9%) received remdesivir. These standard of care treatments were initiated a median of one day before or on the same day as randomisation in all three trial arms and continued once randomised with similar durations in all three arms ranging from a median of 5 days for antivirals and 9 days for anticoagulants (Table 3). More participants in the SOC arm had received antibiotics at baseline (74.6%) compared to the FOS (58.6%) or RUX (64.1%) arms whereas fewer participants initiated antibiotics after randomisation in the SOC arm (22.2%) compared to the FOS (32.8%) and RUX (25.0%) arms (Supplementary Table S3). While fewer participants in the SOC (14.3%) arm had received an interleukin-6 (IL6) inhibitor at baseline compared to the FOS (19.0%) and RUX (20.3%) arms (Supplementary Table S2), these were initiated more frequently after randomisation in the SOC arm (14.3%) compared to the FOS (6.9%) and RUX (9.4%) arms (Supplementary Table S4).

Table 3: Standard of care treatments received at or prior to randomisation by treatment arm

Drug group	FOS (N=58)	RUX (N=64)	SOC (N=63)
Anti-viral agents			
Received ≥1 dose, n (%)	27 (46.6)	31 (48.4)	29 (46.0)
Median (IQR) time since initiation (days); n missing	0 (0, 1); 0	0 (0, 1); 0	0 (0, 1); 0
Median (IQR) duration (days); n missing	5 (4, 6); 0	5 (5, 6); 0	5 (4, 5); 0

For peer review only - http://bmjopen.bmj.com/site/about/guidelines.xhtml

Immuno-modulating agents			
Received ≥1 dose, n (%)	56 (96.6)	58 (90.6)	56 (88.9)
Median (IQR) time since initiation (days); n missing	1 (0, 1); 0	1 (0, 1); 0	1 (0, 1); 0
Median (IQR) duration (days); n missing	6 (4, 9); 0	8 (5, 11); 0	7 (4, 10); 1
Antibiotics			
Received ≥1 dose, n (%)	34 (58.6)	41 (64.1)	47 (74.6)
Median (IQR) time since initiation (days); n missing	1 (0, 2); 0	1 (0, 2); 0	1 (1, 2); 0
Median (IQR) duration (days); n missing	5 (2, 7); 0	4 (2, 7); 0	6 (2, 7); 0
Anticoagulation treatment			
Received ≥1 dose, n (%)	54 (93.1)	54 (84.4)	57 (90.5)
Median (IQR) time since initiation (days); n missing	1 (0, 1); 0	1 (0, 1); 0	1 (0, 1); 0
Median (IQR) duration (days); n missing	9 (5, 25); 1	9 (5, 13); 1	9 (6, 13); 0
Antiplatelet therapy			
Received ≥1 dose, n (%)	6 (10.3)	1 (1.6)	2 (3.2)
Median (IQR) time since initiation (days); n missing	1 (0, 1); 0	1 (1, 1); 0	2 (0, 2); 0
Median (IQR) duration (days); n missing	17 (3, 30); 4	Missing	1 (1, 1); 0
Convalescent serum therapy			
Received ≥1 dose, n (%)	0 (0.0)	1 (1.6)	1 (1.6)
Median (IQR) time since initiation (days); n missing	N/A	6 (6, 6); 0	0 (0, 0); 0
Median (IQR) duration (days); n missing	N/A	1 (1, 1); 0	2 (2, 2); 0
Ronapreve®			
Received ≥1 dose, n (%)	1 (1.7)	1 (1.6)	0 (0.0)
Median (IQR) time since initiation (days); n missing	0 (0, 0); 0	0 (0, 0); 0	N/A
Median (IQR) duration (days); n missing	1 (1, 1); 0	1 (1, 1); 0	N/A

N/A: not applicable

Prior to randomisation, similar proportions (approximately 6-9%) of participants had been co-enrolled on another RCT across the three trial arms. Co-enrolment after randomisation to MATIS was higher in the SOC arm (11.1%) and RUX arm (9.4%) than in the FOS arm (3.4%) (Supplementary Table S5).

Primary outcome

At Stage 1 (N=177), there was no evidence of a reduction in the odds of progression to severe COVID-19 pneumonia for FOS: adjusted odds ratio, compared to SOC:

For peer review only - http://bmjopen.bmj.com/site/about/guidelines.xhtml

1.12; 95% CI 0.49 to 2.58; one-sided p value = 0.608 (Table 4). The Independent Data Monitoring Committee (IDMC) recommended stopping the FOS arm for futility at this stage on the basis of the one-sided p-value exceeding the prespecified threshold of 0.25[9]. Recruitment to the RUX (and SOC) arm was recommended to continue (p=0.152, Table 4), however, due to the slow recruitment because of the reduction in new COVID-19 cases, the trial was stopped early on 2nd September 2022 after only recruiting an additional four participants. Therefore, the final analysis presented in this manuscript includes all three trial arms as if they had been a conventional one-stage RCT comparing RUX and FOS with SOC.

Table 4: Primary Outcome Statistical Model Results

Grade >=5 by Day 14	N	n[a] (%)	Unadjusted		Adjusted[b]	
			Odds ratio (95% CI)	p-value[c]	Odds ratio (95% CI)	p-value[c]
Stage 1						
Fostamatinib	58	16 (27.6)	1.13 (0.50 to 2.58)	0.766	1.12 (0.49 to 2.58)	**0.608**
Ruxolitinib	59	10 (16.9)	0.62 (0.25 to 1.53)	0.300	0.60 (0.22 to 1.60)	**0.152**
SOC	60	15 (25.0)	1.0		1.0	
Final Analysis						
Fostamatinib	58	16 (27.6)	1.19 (0.52 to 2.71)	0.684	1.19 (0.51 to 2.76)	**0.659**
Ruxolitinib	62	10 (16.1)	0.59 (0.24 to 1.44)	0.243	0.63 (0.25 to 1.57)	**0.161**
SOC	61	15 (24.6)	1.0		1.0	

[a] multiple imputation used at Stage 1 and final analysis to impute primary outcome status for 3 patients in the FOS arm, 4 in the RUX arm and 4 in the SOC arm.

For peer review only - http://bmjopen.bmj.com/site/about/guidelines.xhtml

[b] Stage1: propensity score adjusted; Final analysis: adjusted for baseline modified WHO COVID severity, age category, use of IL6 inhibitor and prior COVID vaccination

[c] p-values are two-sided except for those in **bold** where one-sided p-values are presented; p-values for final analysis are nominal as planned sample size not reached

In the final analysis (N=181, Table 4), the numbers (%) of participants with severe COVID-19 pneumonia by Day 14 were 16 (27.6%, N=58) and 10 (16.1%, N=62) in the FOS and RUX arms respectively, and 15 (24.6%, N=61) in the SOC arm. There remained a numerically higher odds of reaching a modified WHO COVID-19 severity grade ≥5 in the FOS arm compared to SOC (OR: 1.19; 95% CI: 0.51 to 2.76; nominal one-sided p-value=0.659). In contrast, a numerically lower odds of severe COVID-19 pneumonia was observed for RUX compared to SOC (OR: 0.63; 95% CI: 0.25 to 1.57; nominal p=0.161). In both comparisons, the 95% CI was wide and included 1. Study conclusions remained unchanged when applying alternative missing data assumptions (Supplementary Table S6).

Secondary outcomes

Results from the primary analysis model when extended to include data up to Day 28 (modified WHO COVID-19 severity grade≥ 5 by Day 28) were very similar to that for Day 14 (Supplementary Table S7). The primary outcome at Day 14 and Day 28 was most commonly triggered by participants experiencing oxygen saturation levels <90% on receipt of ≥60% inspired oxygen or receipt of non-invasive ventilation (Supplementary Tables S8 to S10). Seven participants died (4 in the FOS arm and 3 in the SOC arm) on or before Day 14 and a further three died (2 in the RUX arm and one in the SOC arm) on or before Day 28. Individual models for each of the components of the primary outcome are presented, however, event counts are small and resulting model estimates are imprecise. Results from the mixed ordinal logistic

For peer review only - http://bmjopen.bmj.com/site/about/guidelines.xhtml

regression model were broadly consistent with the primary analysis model (Supplementary Table S11).

Nine participants (4 in the FOS arm, 3 in the RUX arm and 2 in the SOC arm) received mechanical ventilation by Day 14, of whom 6 received organ support (inotropes only; no participants received ECMO) (Table Supplementary S8). Separately, one participant with pre-existing ESRF received RRT. Six participants (2 in the FOS arm, 3 in the RUX arm and one in the SOC arm) had a venous thromboembolism (VTE) event including one participant with a serious VTE in the SOC arm. Two participants (one in the RUX and one in the FOS arm) had the VTE event on the day of randomisation.

There was no evidence of a difference in the time to discharge from randomisation for either FOS or RUX compared to SOC (Supplementary Figure S1). Hazard ratios from the Cox model were 0.96 (95% CI 0.64 to 1.43) for FOS vs SOC and 1.05 (95% CI 0.71 to 1.55) for RUX vs SOC, after adjusting for age, site, prior COVID vaccination, receipt of steroids and receipt of IL6 inhibitors at baseline. Readmission by Day 28 was more frequent in the RUX (8 participants, 13.8%) and SOC (8, 14.6%) arms than in the FOS arm (3, 5.6%) (Supplementary Table S12). No difference in levels of inflammatory markers was observed between trial arms (Supplementary Table S13). Statistically significant differences in serum creatinine levels were noted with higher mean values in the FOS arm at Day 14 (adjusted mean difference vs. SOC: 34.3 μmol/L; 95% CI 6.8 to 61.8) and at Day 28 (43.6 μmol/L; 95% CI 15.7 to 71.5) and in the FOS arm at Day 28 (adjusted mean difference vs. SOC: 27.2 μmol/L; 95% CI 0.6 to 53.7) (Supplementary Table S13).

Safety

For peer review only - http://bmjopen.bmj.com/site/about/guidelines.xhtml

Safety was evaluated in all 181 eligible, randomised participants. Four patients were excluded from this analysis as they were randomised in error. A total of 48 SAEs were reported in 38 participants with a higher proportion experiencing at least one SAE in the RUX (14, 22.6% of participants) and FOS arms (13, 22.4%) compared to SOC (11, 17.5%) (Table 5).

Table 5: Summary of Serious Adverse Events type by treatment arm

	Fostamatinib (N=58)		Ruxolitinib (N=62)		Standard of care (N=61)	
Event type	Number of events	Number of participants with events (%)	Number of events	Number of participants with events (%)	Number of events	Number of participants with events (%)
SAE	14	13 (22.4)	18	14 (22.6)	16	11 (17.5)
SAR	1	1 (1.7)	2	2 (3.2)	0	0 (0.0)
USAR	0	0 (0.0)	2[a]	2 (3.2)	0	0 (0.0)

Acronyms: SAE – serious adverse event; SAR – serious adverse reaction; USAR – unexpected serious adverse reaction

[a] This included one patient with hypotension and one patient with lymphoedema

Three of the reported SAEs were considered to be possibly related to study treatment. These were classified as serious adverse reactions (SARs) and included one expected SAR in the FOS arm (lung infection) and two unexpected SARs in the RUX arm (one case each of hypotension and lymphoedema). The most frequently reported SAE in all three trial arms were infections with numerically higher frequencies in the FOS (10, 17.2%) and ruxolitinib (10, 16.1%) arms compared to SOC (7, 11.5%) (Supplementary Figures S2 and S3). These included 5 cases of lung infection in RUX arm (4 participants) and 3 cases in in the FOS arm (3 participants), both higher than the SOC arm (1 participant). One event of lung

For peer review only - http://bmjopen.bmj.com/site/about/guidelines.xhtml

infection in the RUX arm resulted in a fatal outcome. None of the 10 fatal events recorded in the trial were considered to be related to study treatment.

Exploratory analyses

Results from the per-protocol analysis in participants who received ≥ 90% of prescribed dose were consistent with the primary analysis (Supplementary Table S14). The complier-adjusted risk difference (RD) estimates were slightly further away from the null than the corresponding ITT analyses but did not reach statistical significance (complier-adjusted vs ITT: FOS vs SOC: RD 0.05 vs 0.03; RUX vs SOC: RD -0.10 vs -0.06). Stratification of the analysis by grouping participants into approximately four equal sized groups based on time of randomisation resulted in somewhat imprecise estimates and were inconclusive (Supplementary Table S15). There was no strong evidence for any subgroup effects (Supplementary Figures S4 and S5). These results should be interpreted with caution due to the small sample size within subgroups.

Discussion

Principal findings

MATIS was an open-label, multi-centre, multi-arm, and multi-stage randomised controlled trial of ruxolitinib and fostamatinib for COVID-19 pneumonia compared to routine standard of care. The study was designed between March and April 2020, with participants recruited largely from the second and third waves of the UK pandemic, with the majority of participants recruited from three hospitals in North-

For peer review only - http://bmjopen.bmj.com/site/about/guidelines.xhtml

West London. Our efficient, multi-stage, multi-arm design allowed early stopping of the fostamatinib arm for futility. This design allows recruitment efforts to focus on more promising treatments, a particularly important feature in the pandemic setting. However, due to the success of the COVID-19 vaccine leading to a dramatic reduction in hospitalisation and mortality, the study was closed in September 2022, before reaching the power needed to determine the effect of ruxolitinib in the treatment of COVID-19 pneumonia in patients requiring hospital admission.

Although, the study was not powered to assess the secondary outcome of death, no deaths were recorded in the ruxolitinib arm in the first 14 days while 4 participants in the fostamatinib arm and 3 in the SOC arm died during this period. There was no difference in time to discharge between either active treatment and SOC, but there were fewer readmissions in the fostamatinib arm. In terms of safety, there were higher numbers of lung infections in the active treatment arms including one infection-related death in the ruxolitinib arm. However this event was not considered to be related to study treatment. We noted an increase in serum creatinine in both arms; however the clinical relevance of this finding is unclear.

Approximately one third of participants did not complete the 14-day treatment course for fostamatinib and ruxolitinib. The results of our complier-adjusted case analyses, which estimated the impact of adherence on the treatment effects, were consistent with our primary analysis. However, due to the pandemic setting, adherence measures were based on self-report for participants given take-home medication following hospital discharge rather than in-person pill counts. Our estimate of adherence and its impact on treatment effects may therefore be under-estimated.

For peer review only - http://bmjopen.bmj.com/site/about/guidelines.xhtml

Comparison with other studies

Ruxolitinib is an inhibitor of the JAK/STAT pathway (Janus kinases and Signal Transducer and Activator of Transcription proteins), specifically inhibiting subtypes JAK1 and JAK2. It has a rapid mode of action with cytokine-induced STAT3 phosphorylation being inhibited within 2 hours of administration, resulting in a reduction of tumour necrosis factor alpha (TNFα), IL6 and CRP. Several other trials studied the effect of ruxolitinib during the pandemic, with variable responses reported, some positive, but most were neutral.[15-17] There were many differences between studies including: types of patients recruited (i.e. differing severity of COVID-19), timing of the study in the pandemic (resulting in variability of the virus over time, changes in standard of care; and the vaccine role out in 2021), and different primary outcomes. As with MATIS, some of these trials were underpowered due to early termination. Furthermore, the appropriate dosing of ruxolitinib to treat or prevent hyperinflammation is yet to be determined. We chose a low to intermediate dose for MATIS, because we had concerns regarding immunosuppression during active phase of the infection. This dose appeared to be appropriate for this cohort of patients given the lower rate of progression to more severe disease and absence of deaths before 14 days in the ruxolitinib arm. Higher doses such as 25mg once a day, used in conditions such as graft versus host disease,[18] or longer duration of treatment (>14 days) may be more appropriate for patients with more severe COVID-19.

Given the CRP and IL6 drive in COVID-19, several clinical trials of other agents in this class (including baracitinib and tofacitinib) targeting patients at different disease stages were performed.[19 20] A meta-analysis of four randomised controlled trials of

For peer review only - http://bmjopen.bmj.com/site/about/guidelines.xhtml

baracitinib in hospitalised patients with COVID-19, including 10,815 patients, showed a reduction in 28-day mortality (OR 0.69, 95% CI 0.5-0.94; p=0.04).[21] Results from two of the large studies included in the meta-analysis, RECOVERY and COV-BARRIER,[22 23] led to the US Food and Drug Administration (FDA), allowing the emergency authorisation of baracitinib with remdesivir in patients with COVID-19 requiring supplemental oxygen, invasive mechanical ventilation, or extracorporeal membrane oxygenation (ECMO).[24]

The RECOVERY study also observed a 15% reduction mortality for tocilizumab (an IL6 inhibitor) compared to usual care.[25] Tociluzimab was added to standard of care during MATIS and was used in approximately one fifth of the patients in this trial.

Fostamatinib is a SYK pathway inhibitor, licensed for use in patients with immune thrombocytopenia. The SYK pathway appears to be critical in the pathogenesis of COVID-19 as well as other severe acute respiratory syndrome (SARS) infections, modulating cytokine secretion, and promoting the influx of immune cells such as macrophages and monocyte into the lung, contributing to both the hyperinflammatory state and long-term lung damage. As such, SYK inhibition appeared as a novel mechanism to treat COVID-19 pneumonia through the prevention of cytokine release, lung inflammation and platelet activation, all critical events in COVID-19 pathway. [26 27]

Despite a plausible biological mechanism of action, we found no evidence that fostamatinib prevented progression of COVID-19 to more severe disease in this cohort of patients. It is possible that our patient cohort were too mildly affected to have any benefit from fostamatinib. It is also possible we did not use the correct dose, or duration of fostamatinib. Another RCT comparing fostamatinib 100 mg twice

For peer review only - http://bmjopen.bmj.com/site/about/guidelines.xhtml

CONFIDENTIAL PRE-PUBLICATION MANUSCRIPT

daily plus SOC to placebo plus SOC included patients with more severe COVID-19 (over half of the patients required non-invasive ventilation or high-flow oxygen devices [56.7% fostamatinib and 51.7% placebo], or invasive mechanical ventilation and/or ECMO [36.7% FOS and 41.1% placebo]).[28] In that study, the rates of SAEs by Day 29 (the primary end point) were not statistically different between the fostamatinib group and the placebo group (10.5% vs 22.0%, p=0.2), while secondary endpoints, which the study was not powered for, such as 28-day mortality, days on supplemental oxygen and ordinal scores of clinical status favoured fostamatinib. Both the inclusion criteria and the dose of fostamatinib were different to MATIS limiting their comparison, but potentially implying the timing of the use of fostamatinib in diseases such as COVID-19 might be critical.

Another double blind, randomized, placebo-controlled trial of fostamatinib in patients with COVID-19 on supplementary oxygen (the FOCUS trial) recruited 280 patients.[29] Although results are not yet published in full, the FOCUS trial met its primary endpoint with patients in the fostamatinib arm having a reduced number of days on supplemental oxygen compared to placebo (4.8 vs. 7.6 days, respectively, p=0.0136). Fostamatinib also showed a trend in reducing COVID-19 related mortality and morbidity. Unlike MATIS, this study also recruited patients with more severe COVID-19; all 3 participants with a baseline score of 6 on the 8-point COVID-19 ordinal scale in the placebo group died by Day 30, and all 3 participants with this baseline score in the fostamatinib group survived, suggesting there may be particular benefit for patients with more severe COVID-19, although numbers in this cohort remain very small.

For peer review only - http://bmjopen.bmj.com/site/about/guidelines.xhtml

The study designs were different for these trials, limiting comparisons, but overall, it is possible that the timing of the use of fostamatinib in diseases such as COVID-19 is critical, with greater benefit in patients with more severe disease, preventing the progression of lung disease, rather than earlier in the infection, when anti-viral responses may be more important.

Strength and Limitations:

Patient population and recruitment:

Despite a strong study design, we were unable to recruit the numbers of participants needed to detect a treatment effect for ruxolitinib or fostamatinib due to reducing numbers of patients being admitted to hospital as the pandemic evolved. Recruitment to MATIS was also limited by competition from other large concurrent COVID-19 trials. Another limitation is the representativeness of patients in the trial in terms of age of the patients recruited. Older patients, who had the highest mortality in COVID-19, although approached for MATIS, were less likely to consent to the trial. We do not know the reason for this, but possible causes include family members declining study; fear of the medical profession; fear of the virus; patients being too unwell to read the patient information sheet; flaws in recruitment strategies in a fast-moving time. Recruitment of participants was predominantly from one large teaching hospital in London with a small number recruited from hospitals in other UK cities. Patients were admitted to this hospital through general acute medicine pathways rather than through any tertiary referral mechanism. We therefore consider the study population to be broadly representative of the general urban UK population requiring hospital admission for treatment of COVID-19. The demographics of participants showed broad ethnic representation (approximately 40% of participants were white

For peer review only - http://bmjopen.bmj.com/site/about/guidelines.xhtml

and 60% were non-white), reflecting the recruitment from inner city hospitals in the UK and was a strength of the study.

The target population in MATIS was restricted to hospitalised patients. This included patients admitted with escalating symptoms and/or with a higher risk of progression to more severe disease due to underlying comorbidities. The trial was not designed to explore whether these treatments may offer any benefits to the wider community of patients with milder COVID-19 or to those that did not seek or receive hospital care.

Evolving Standard of Care

At or prior to randomisation, 46.0% to 48.4% of patients in MATIS received anti-viral agents, 88.9% to 96.6% received immune-modulating agents, 58.6% to 74.6% received antibiotics, 84.4% to 93.1% received anticoagulation treatment, and 1.6% to 10.3% received antiplatelet therapy. The frequency of use of these agents was similar between trial arms but we did not examine whether there were any differences in the numbers of agents or doses used. However, the standard of care for treating COVID-19 changed over time, reflecting the dynamic nature of the evolving pandemic. Initially treatments were focused on supportive care strategies. As standard of care evolved, antiviral medications, anti-inflammatory agents, and eventually vaccination campaigns were integrated into treatment protocols. In 2021, there was a successful role out of vaccines. The changes in recommended treatments over distinct phases of the pandemic created additional complexity to COVID-19 treatment clinical trials impacting on patient profile, recruitment numbers and the changing use of multiple immunomodulatory agents. Within MATIS, the use

For peer review only - http://bmjopen.bmj.com/site/about/guidelines.xhtml

of IL6-inhibitors was introduced at Imperial College NHS Trust (the main study site) for patients who were developing hyper-inflammation. We note that fewer patients in the active treatment arms required treatment with an IL6 inhibitor post-randomisation.

Implications for practice and future research

One of the unique contributing factors to the high individual mortality, as well as the population level impact of COVID-19, was the triggering of a hyperinflammatory state causing a rapid escalation to ventilation in many patients. The excessive need for ventilation also impacted the rest of the population with hospitals running out of ventilators and intensive care staff.

In designing MATIS, we chose two different immune modulators as early interventions (within the first few days of admission), aiming to reduce inflammation, prevent the requirement for ventilation and improve the outcome for these patients, as well as reducing the impact of COVID-19 on UK hospitals.

To identify patients who we thought were at risk of hyperinflammation, and therefore more likely to benefit from immune modulation, we included patients with a raised CRP. However, this may not be specific enough. The RuxCoFlam trial which showed a significant effect of ruxolitinib selected patients based on a composite COVID-19 inflammation score (CIS) made up of multiple inflammatory markers.[30] This included inter alia CRP, ferritin, triglcycerides, fibrinogen, total white blood cell count, lymphocytes and D-dimer and activated partial thromboplastin time. For CRP to contribute to the CIS score, values were required to exceed 20 times the upper limit of the normal reference range. Subsequent research, including evidence from our own studies, has shown the importance of host genetic factors in COVID-19 mortality demonstrating an exaggerated hyperinflammatory response to this novel virus.[31,32]

For peer review only - http://bmjopen.bmj.com/site/about/guidelines.xhtml

Testing patients for these host factors, and measuring specific immune responses, may allow us to target patients more effectively for immune modulation, and clarify which modulators are most appropriate at an individual level. A biomarker study is now underway in samples stored prior to randomisation of MATIS to see if we can better fine-tune immune modulation therapy in virally induced hyperinflammation. Other patient characteristics such as age, obesity, diabetes and cardiovascular risk factors are independently associated with the development of secondary infection thrombosis, requirement for ventilation and death.[33] Meta-analysis of JAK/STAT inhibition from all studies may help identify the cohorts of patients most likely to benefit from these treatments.

The timing and choice of immune-modulation in diseases such as COVID-19 could be critical for improving outcome, giving the immune system time to effectively eradicate the virus, but preventing the rapid escalation to hyperinflammation.

The results of these studies are likely to be important in guiding the management of other virally induced multisystemic disorders.

For peer review only

Conclusion

In conclusion, we did not observe a significant treatment effect for fostamatinib in comparison to standard of care in reducing the proportion of hospitalised patients progressing to severe COVID-19 pneumonia. Results for ruxolitinib could not be confirmed due to early termination of our trial. Other factors such as patient comorbidities, choice of dose and timing of treatment may also have contributed to

For peer review only - http://bmjopen.bmj.com/site/about/guidelines.xhtml

these findings. Further research is needed to help us to further understand these results and further investigation of the role of JAK/STAT inhibition in hyperinflammatory conditions triggered by viruses is now warranted, as well as long term follow up of patients who received immune modulation.

For peer review only

References

1. Huang C, Wang Y, Li X, et al. Clinical features of patients infected with 2019 novel coronavirus in Wuhan, China. *Lancet* 2020;395(10223):497-506. doi: 10.1016/S0140-6736(20)30183-5 [published Online First: 20200124]

2. Ruan Q, Yang K, Wang W, et al. Clinical predictors of mortality due to COVID-19 based on an analysis of data of 150 patients from Wuhan, China. *Intensive Care*

For peer review only - http://bmjopen.bmj.com/site/about/guidelines.xhtml

CONFIDENTIAL PRE-PUBLICATION MANUSCRIPT

Med 2020;46(5):846-48. doi: 10.1007/s00134-020-05991-x [published Online First: 20200303]

3. Siddiqi HK, Mehra MR. COVID-19 illness in native and immunosuppressed states: A clinical-therapeutic staging proposal. *J Heart Lung Transplant* 2020;39(5):405-07. doi: 10.1016/j.healun.2020.03.012 [published Online First: 20200320]

4. Spalton JC, Mori J, Pollitt AY, et al. The novel Syk inhibitor R406 reveals mechanistic differences in the initiation of GPVI and CLEC-2 signaling in platelets. *J Thromb Haemost* 2009;7(7):1192-9. doi: 10.1111/j.1538-7836.2009.03451.x [published Online First: 20090424]

5. Lhermusier T, van Rottem J, Garcia C, et al. The Syk-kinase inhibitor R406 impairs platelet activation and monocyte tissue factor expression triggered by heparin-PF4 complex directed antibodies. *J Thromb Haemost* 2011;9(10):2067-76. doi: 10.1111/j.1538-7836.2011.04470.x

6. Novartis. Jakavi 10mg tablets. 2022 [Available from: https://www.medicines.org.uk/emc/product/7786/smpc#gref.

7. Grifols UK Ltd. Tavlesse 100 mg film-coated tablets 2023 [Available from: https://www.medicines.org.uk/emc/product/11479/smpc#gref accessed 7th August 2024.

8. World Health Organisation. COVID-19 Therapeutic Trial Synopsis. Geneva Switzerland: World Health Organisation, 2020.

9. Vergis N, Phillips R, Cornelius V, et al. Multi-arm Trial of Inflammatory Signal Inhibitors (MATIS) for hospitalised patients with mild or moderate COVID-19 pneumonia: a structured summary of a study protocol for a randomised controlled trial. *Trials* 2021;22(1):270. doi: 10.1186/s13063-021-05190-z [published Online First: 20210412]

For peer review only

For peer review only - http://bmjopen.bmj.com/site/about/guidelines.xhtml

10. Schulz KF, Altman DG, Moher D, et al. CONSORT 2010 statement: updated guidelines for reporting parallel group randomised trials. *BMJ* 2010;340:c332. doi: 10.1136/bmj.c332 [published Online First: 20100323]

11. White IR, Royston P, Wood AM. Multiple imputation using chained equations: Issues and guidance for practice. *Stat Med* 2011;30(4):377-99. doi: 10.1002/sim.4067 [published Online First: 20101130]

12. Cook JA, MacLennan GS, Palmer T, et al. Instrumental variable methods for a binary outcome were used to informatively address noncompliance in a randomized trial in surgery. *J Clin Epidemiol* 2018;96:126-32. doi: 10.1016/j.jclinepi.2017.11.011 [published Online First: 20171120]

13. Rickham PP. Human Experimentation. Code of Ethics of the World Medical Association. Declaration of Helsinki. *Br Med J* 1964;2(5402):177. doi: 10.1136/bmj.2.5402.177

14. Elliott P, Whitaker M, Tang D, et al. Design and Implementation of a National SARS-CoV-2 Monitoring Program in England: REACT-1 Study. *Am J Public Health* 2023;113(5):545-54. doi: 10.2105/AJPH.2023.307230 [published Online First: 20230309]

15. Cao Y, Wei J, Zou L, et al. Ruxolitinib in treatment of severe coronavirus disease 2019 (COVID-19): A multicenter, single-blind, randomized controlled trial. *J Allergy Clin Immunol* 2020;146(1):137-46 e3. doi: 10.1016/j.jaci.2020.05.019 [published Online First: 20200526]

16. Han MK, Antila M, Ficker JH, et al. Ruxolitinib in addition to standard of care for the treatment of patients admitted to hospital with COVID-19 (RUXCOVID): a randomised, double-blind, placebo-controlled, phase 3 trial. *Lancet Rheumatol*

For peer review only

CONFIDENTIAL PRE-PUBLICATION MANUSCRIPT

2022;4(5):e351-e61. doi: 10.1016/S2665-9913(22)00044-3 [published Online First: 20220329]

17. Rein L, Calero K, Shah R, et al. Randomized Phase 3 Trial of Ruxolitinib for COVID-19-Associated Acute Respiratory Distress Syndrome. *Crit Care Med* 2022;50(12):1701-13. doi: 10.1097/CCM.0000000000005682 [published Online First: 20221013]

18. Broglie L, Pommert L, Rao S, et al. Ruxolitinib for treatment of refractory hemophagocytic lymphohistiocytosis. *Blood Adv* 2017;1(19):1533-36. doi: 10.1182/bloodadvances.2017007526 [published Online First: 20170817]

19. Guimaraes PO, Quirk D, Furtado RH, et al. Tofacitinib in Patients Hospitalized with Covid-19 Pneumonia. *N Engl J Med* 2021;385(5):406-15. doi: 10.1056/NEJMoa2101643 [published Online First: 20210616]

20. Kalil AC, Patterson TF, Mehta AK, et al. Baricitinib plus Remdesivir for Hospitalized Adults with Covid-19. *N Engl J Med* 2021;384(9):795-807. doi: 10.1056/NEJMoa2031994 [published Online First: 20201211]

21. Selvaraj V, Finn A, Lal A, et al. Baricitinib in hospitalised patients with COVID-19: A meta-analysis of randomised controlled trials. *EClinicalMedicine* 2022;49:101489. doi: 10.1016/j.eclinm.2022.101489 [published Online First: 20220603]

22. Recovery Collaborative Group. Baricitinib in patients admitted to hospital with COVID-19 (RECOVERY): a randomised, controlled, open-label, platform trial and updated meta-analysis. *Lancet* 2022;400(10349):359-68. doi: 10.1016/S0140-6736(22)01109-6

23. Marconi VC, Ramanan AV, de Bono S, et al. Efficacy and safety of baricitinib for the treatment of hospitalised adults with COVID-19 (COV-BARRIER): a randomised, double-blind, parallel-group, placebo-controlled phase 3 trial. *Lancet Respir Med*

For peer review only - http://bmjopen.bmj.com/site/about/guidelines.xhtml

2021;9(12):1407-18. doi: 10.1016/S2213-2600(21)00331-3 [published Online First: 20210901]

24. US Food and Drug Administration. Letter of authorization: EUA for baricitinib (Olumiant) for treatment of coronavirus disease 2019 (COVID-19). 2022. https://www.fda.gov/media/143822/download (accessed 23/12/2024).

25. Recovery Collaborative Group. Tocilizumab in patients admitted to hospital with COVID-19 (RECOVERY): a randomised, controlled, open-label, platform trial. *Lancet* 2021;397(10285):1637-45. doi: 10.1016/S0140-6736(21)00676-0

26. Nadeem A, Ahmad SF, Al-Harbi NO, et al. Inhibition of spleen tyrosine kinase signaling protects against acute lung injury through blockade of NADPH oxidase and IL-17A in neutrophils and gammadelta T cells respectively in mice. *Int Immunopharmacol* 2019;68:39-47. doi: 10.1016/j.intimp.2018.12.062 [published Online First: 20190102]

27. Alimova M, Sidhom EH, Satyam A, et al. A High Content Screen for Mucin-1-Reducing Compounds Identifies Fostamatinib as a Candidate for Rapid Repurposing for Acute Lung Injury during the COVID-19 pandemic. *bioRxiv* 2020 doi: 10.1101/2020.06.30.180380 [published Online First: 20200630]

28. Strich JR, Tian X, Samour M, et al. Fostamatinib for the Treatment of Hospitalized Adults With Coronavirus Disease 2019: A Randomized Trial. *Clin Infect Dis* 2022;75(1):e491-e98. doi: 10.1093/cid/ciab732

29. Gotur DB, Malik A, Markovtsov V, et al. 88. Fostamatinib for the Treatment of Hospitalized Patients With COVID-19 Who Required Oxygen Supplementation: Results of a Phase 3 Trial. *Open Forum Infectious Diseases* 2023;10(Supplement_2) doi: 10.1093/ofid/ofad500.004

For peer review only - http://bmjopen.bmj.com/site/about/guidelines.xhtml

30. Hammersen J, Birndt S, Dohner K, et al. The JAK1/2 inhibitor ruxolitinib in patients with COVID-19 triggered hyperinflammation: the RuxCoFlam trial. *Leukemia* 2023;37(9):1879-86. doi: 10.1038/s41375-023-01979-w [published Online First: 20230728]

31. Velavan TP, Pallerla SR, Ruter J, et al. Host genetic factors determining COVID-19 susceptibility and severity. *EBioMedicine* 2021;72:103629. doi: 10.1016/j.ebiom.2021.103629 [published Online First: 20211013]

32. Malik A, Toth EN, Teng MS, et al. Distorted TCR repertoires define multisystem inflammatory syndrome in children. *PLoS One* 2022;17(10):e0274289. doi: 10.1371/journal.pone.0274289 [published Online First: 20221027]

33. Ruan Q, Yang K, Wang W, et al. Correction to: Clinical predictors of mortality due to COVID-19 based on an analysis of data of 150 patients from Wuhan, China. *Intensive Care Med* 2020;46(6):1294-97. doi: 10.1007/s00134-020-06028-z

For peer review only

Acknowledgements

The authors would like to thank the patients (and their families) who generously gave their time to take part in this study, the nurses and clinicians who assisted with patient recruitment, and the pharmacy and laboratory teams at participating hospitals. We also acknowledge the invaluable contributions of the research staff and coordinators at all participating sites and Sapna Dave for statistical support in preparing the study results. We would like to thank the members of the trial steering

For peer review only - http://bmjopen.bmj.com/site/about/guidelines.xhtml

committee, the data management committee and the trial management group for their oversight and guidance throughout the trial.

Data sharing

De-identified participant-level data (excluding free text fields) and supporting documentation (including the MATIS study protocol, Statistical Analysis Plan and data dictionary) can be made available upon reasonable request from the corresponding author (n.cooper@imperial.ac.uk) for the purposes of scientific research including secondary analysis of the data or for individual participant meta-analysis with appropriate human research ethics approvals and data transfer agreements in place.

For peer review only

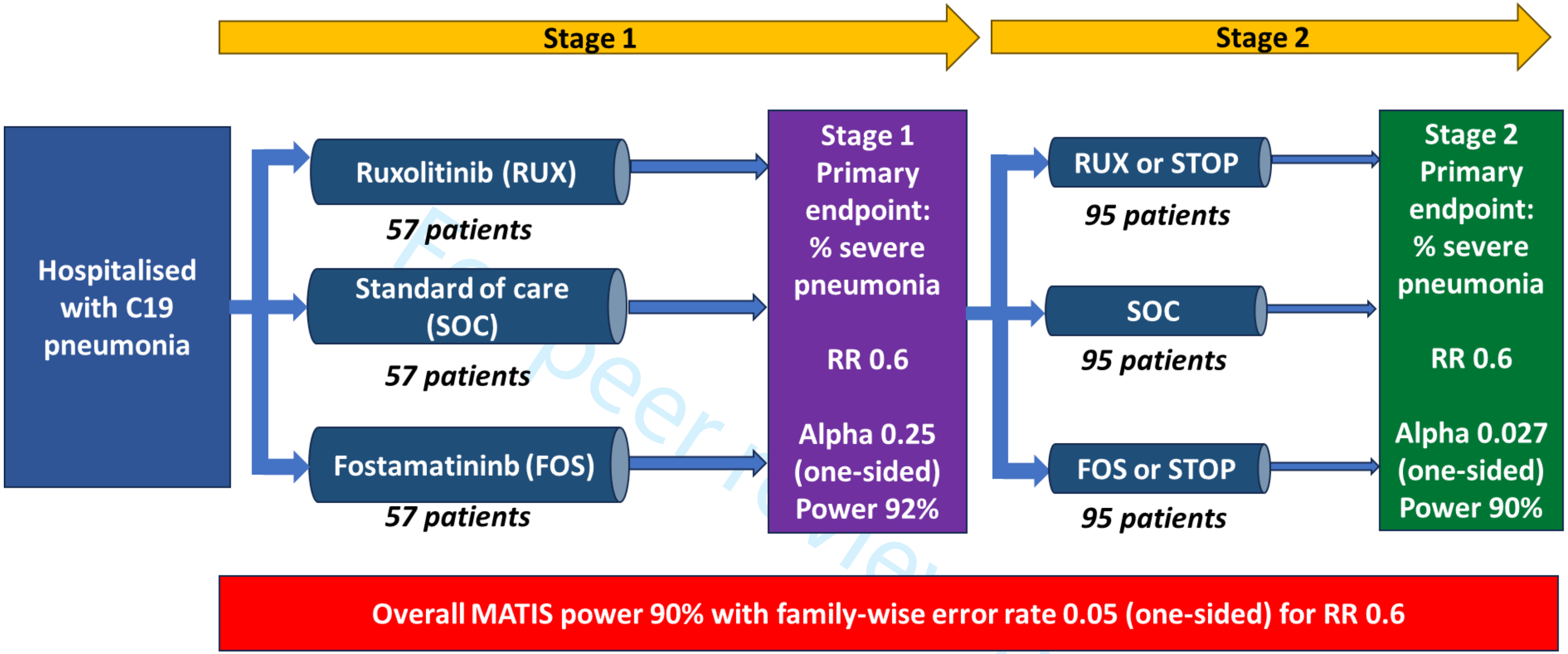

Figure 1: MATIS Trial Design

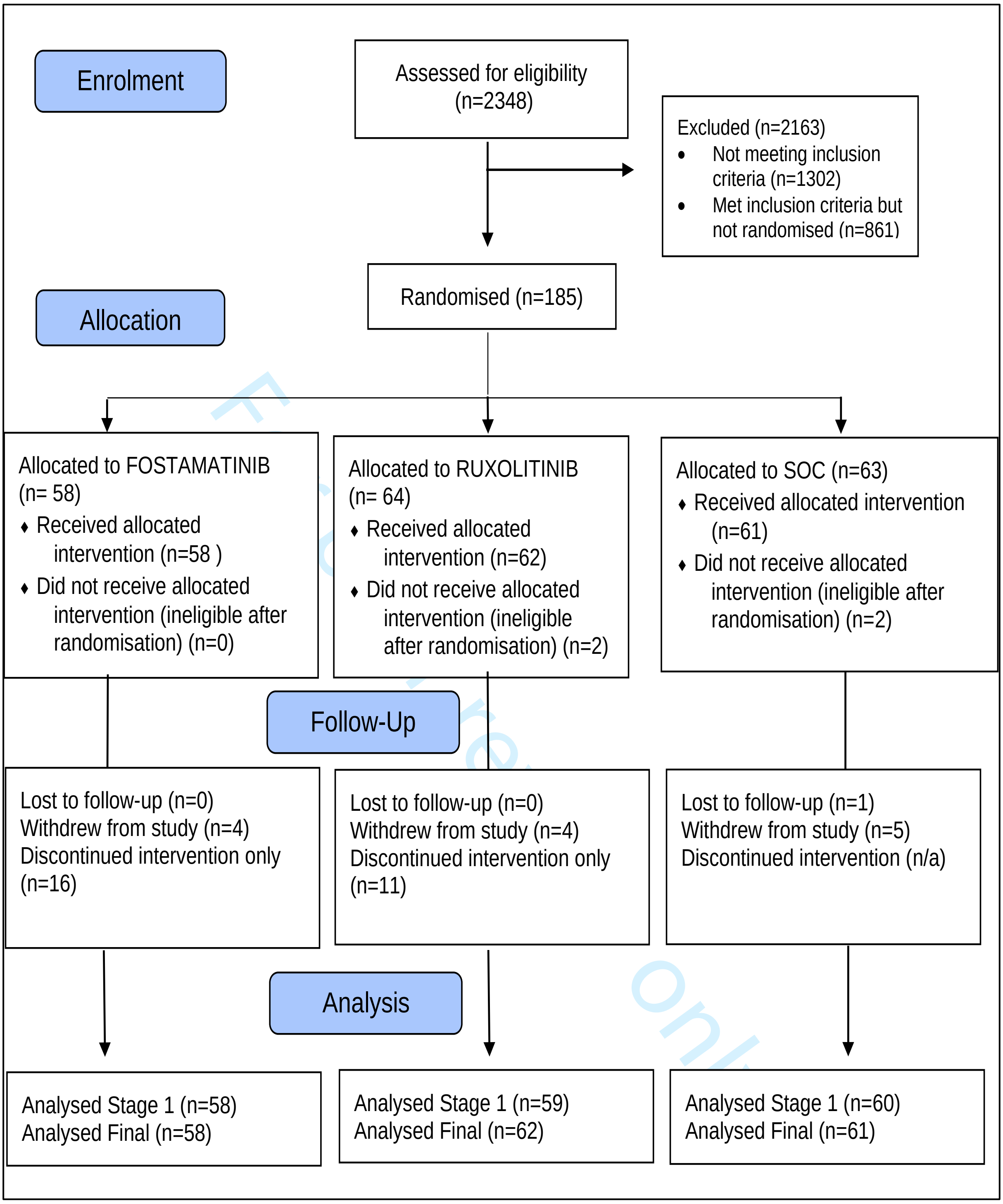

Figure 2: Participant flow

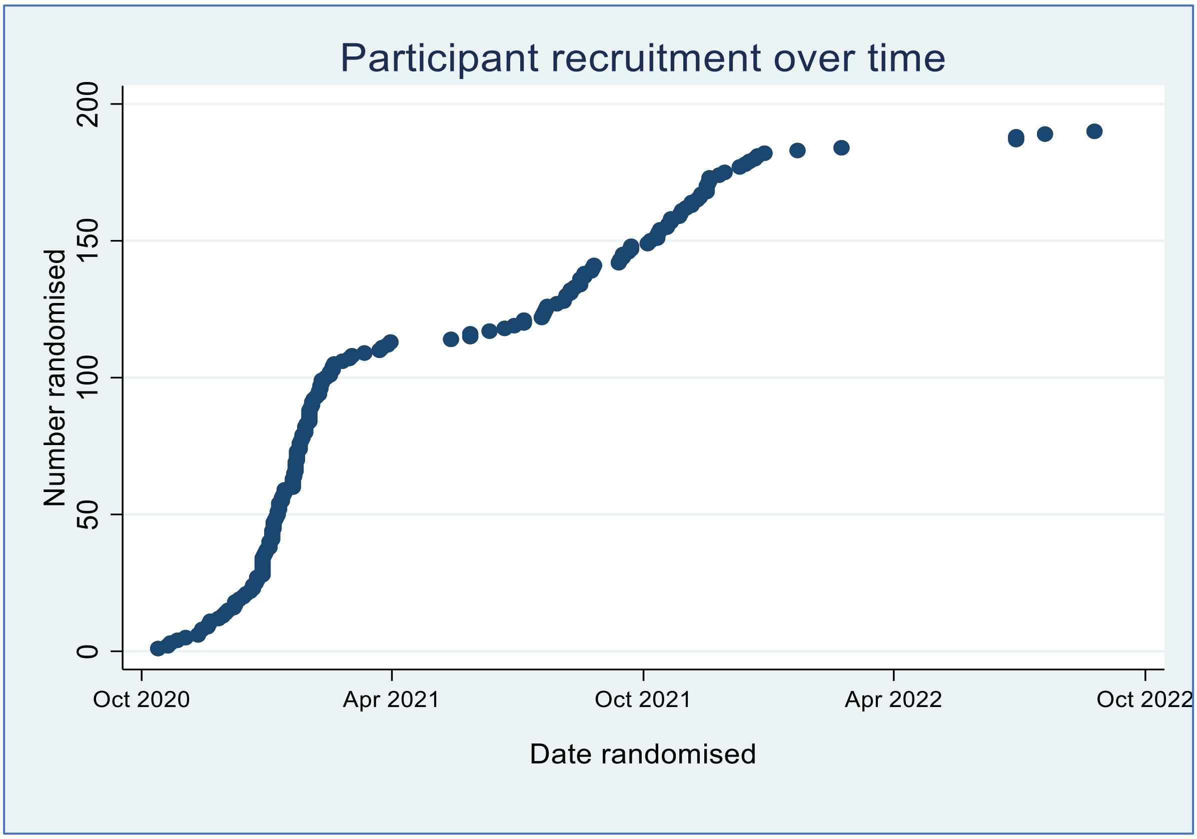

Figure 3: Recruitment over time

review only

MATIS TRIAL

Multi-arm trial of Inflammatory Signal Inhibitors for COVID-19

Statistical Analysis Plan II – Formal Interim Analysis and Final Analysis

Version 1.0

Date: 29/11/2021

EudraCT Number: 2020-001750-22

IRAS ID: 282552

[redacted]

[redacted]

[redacted]

[redacted]

[redacted]

[redacted]

[redacted]

For peer review only - http://bmjopen.bmj.com/site/about/guidelines.xhtml

[redacted]

For peer review only

For peer review only - http://bmjopen.bmj.com/site/about/guidelines.xhtml

Document version history log

Date	Version	Changes made
29/11/2021	1.0	

For peer review only

For peer review only - http://bmjopen.bmj.com/site/about/guidelines.xhtml

Table of contents

1. Study summary ... 7
1.1 Principal research objectives ... 7
1.1.1 Primary objective ... 7
1.1.2 Primary outcome of the main study ... 7
1.1.3 Secondary outcomes of the main study ... 7
1.2 Trial design ... 8
1.3 Study population ... 10
1.3.1 Eligibility criteria ... 10
1.4 Treatment groups ... 11
1.4.1 Standard of care ... 11
1.4.2 Future changes to Standard of Care ... 11
1.4.3 Ruxolitinib ... 12
1.4.4 Fostamatinib ... 12
1.4.5 Treatment duration ... 12
1.5 Sample size estimation ... 12
1.5.1 Sample size for formal interim analysis (Stage 1) ... 13
1.5.2 Sample size for full trial (Stage 2) ... 13
1.5.3 Informal interim analysis ... 13
1.6 Randomisation ... 14
1.7 Blinding ... 14
1.8 Brief description of proposed analysis ... 14
1.9 Informal interim analysis outcomes - Completed 29th Jan 2021 ... 15
2. Data analysis plan – data description ... 17
2.1 Recruitment and participant flow ... 17
2.2 Baseline characteristics ... 17
2.3 Co-enrolment ... 17
2.4 Standard of Care ... 17
2.5 Withdrawals, loss to follow-up and other missing data ... 17
2.6 Adherence to allocated treatment and dose summaries ... 17
2.7 Descriptive statistics for outcome measures ... 17
2.8 Serious adverse events ... 18
3. Data analysis plan - Inferential analysis ... 20
3.1 Primary outcome of the main study - Modified WHO grading ... 20
3.1.1 Definition of the primary outcome ... 20
3.1.2 Statistical model at stage 1 ... 20
3.1.3 Statistical model at stage 2 ... 23

For peer review only - http://bmjopen.bmj.com/site/about/guidelines.xhtml

3.1.4 Model assessment of stage 2 analysis 25
3.1.5 Stage 2 exploratory analysis for primary outcome 26
3.1.6 Stage 2 subgroup analysis for primary outcome 28
3.2 Secondary outcomes of the main study 28
3.2.1 Severe COVID-19 within 28 days 29
3.2.2 Modified WHO COVID-19 score 29
3.2.3 Components of the modified WHO COVID-19 score 30
3.2.4 Length of stay 31
3.2.5 Continuous laboratory values 31
References 34

For peer review only

For peer review only - http://bmjopen.bmj.com/site/about/guidelines.xhtml

STUDY MANAGEMENT

**CHIEF INVESTIGATOR**	Dr Nichola Cooper
	Imperial College Healthcare NHS Trust, Hammersmith Hospital, Du Cane Road, W12 0HS, London
	[redacted]
**SPONSOR**	Imperial College London
	JRCO, Imperial College London and Imperial College Healthcare NHS Trust; Room 215, Level 2, Medical School Building, St Marys Campus
	[redacted]
	[redacted]
**FUNDER**	Imperial Biomedical Research Centre
**CO-INVESTIGATORS**	[redacted]
	[redacted]
	[redacted]
	[redacted]
	[redacted]
	[redacted]
	[redacted]
	[redacted]
	[redacted]
	[redacted]
**STATISTICIANS**	[redacted]
	[redacted]
	[redacted]
	[redacted]

For peer review only - http://bmjopen.bmj.com/site/about/guidelines.xhtml

1. Study summary

The MATIS trial is a multi-arm, multi-stage randomised controlled trial (RCT) of ruxolitinib (RUX) and fostamatinib (FOS) for COVID-19 pneumonia. The statistical analysis plan (SAP) is based on protocol version 2.2 which can be seen for full design details.

1.1 Principal research objectives

1.1.1 Primary objective

To evaluate the efficacy of ruxolitinib (RUX) and fostamatinib (FOS) compared to standard of care (SOC) in the treatment of COVID-19 pneumonia in participants with mild disease.

1.1.2 Primary outcome of the main study

The proportion with severe COVID-19 pneumonia within 14 days of randomization. Where severe COVID-19 pneumonia is defined by a modified WHO COVID-19 Ordinal Score ≥ 5 (on the 9 point scale), comprising the following indicators of disease severity:

- Death *OR*
- Requirement for invasive ventilation plus organ support OR
- Requirement for invasive ventilation *OR*
- Requirement for non-invasive ventilation including CPAP or high flow oxygen *OR*
- O_2 saturation < 90% on ≥60% inspired oxygen

1.1.3 Secondary outcomes of the main study

Each of the outcomes below at 14 and 28 days between RUX or FOS versus SOC:

- Change in severity on the modified WHO COVID-19 Ordinal Scale (3 or 4 to 5,6,7,8,9)
- Mortality (Y/N)
- Invasive ventilation (Y/N)
- ECMO (Y/N)
- Non-invasive ventilation including CPAP and high flow nasal oxygen (Y/N)
- Renal replacement therapy (Y/N)
- Venous thromboembolism (Y/N)
- Inflammatory markers CRP, LDH, ferritin, D-dimer (units)
- Length of stay (days)
- Serum creatinine (units).

For peer review only - http://bmjopen.bmj.com/site/about/guidelines.xhtml

- Re-admission data – were they readmitted within 28 days? (from free text in SAEs)

1.2 Trial design

A two stage, open label, randomized (1:1:1) controlled trial with formal interim analysis after 171 participants (57 per arm) which also includes an informal interim analysis after 30 participants (10 per arm) complete follow-up. Due to the three different stages in this trial, the MATIS SAP is separated into two sections. The aim of this statistical analysis plan (SAP) is to describe in detail the analysis that will be undertaken for the formal interim analysis and final analysis. The informal interim analysis was to provide an early opportunity to check the dosing regimen, see MATIS SAP I – Dose Verification for a detailed description of the statistical analysis conducted at this stage.

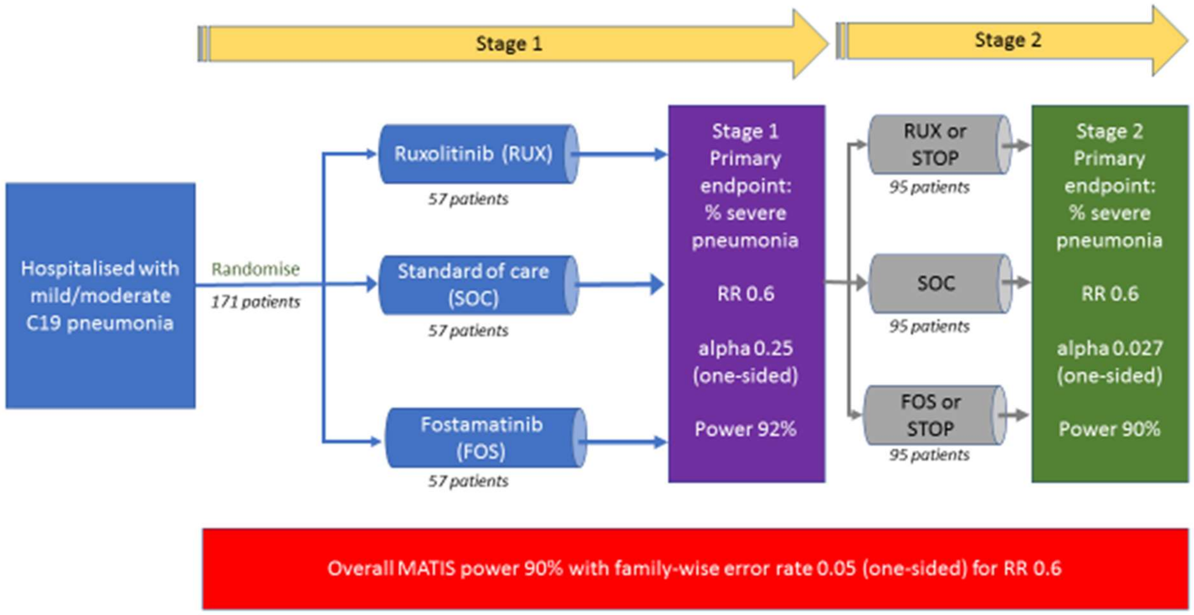

Figure 1: Schematic of study Design

Treatment is for 14 days from baseline. Patients will receive follow-up assessments at 1, 7, 14 and 28 days after the first dose. The time allowed for screening assessments allows time for eligibility assessments to be included.

- Screening Period: up to 7 days to the day of randomisation
- Treatment Period: 14 days
- Follow-up period: Day 14 to 28

Patients who have recovered and are fit for discharge from hospital during the treatment period, will be discharged home with trial medication to complete a fixed 14 day course. Such patients will be followed up weekly with telephone monitoring until day 28 and additional blood test monitoring where practically possible.

For peer review only - http://bmjopen.bmj.com/site/about/guidelines.xhtml

Table 1. Study visits

	Screening	Baseline				Last visit
Study Day	***-6 to 0***	***0***	***1[+]***	***7[#]***	***14[#]***	***28[#]***
Time window		-2 days	*+1 day*	*± 3 day*	*± 3 day*	*± 3 day*
Informed consent	x					
Inclusion/Exclusion criteria	x	x				
Demographic data	x					
Medical history including major comorbidities	x					
Physical exam	x					
COVID-19 diagnosis, duration and severity	x					
Pregnancy test	x					
Vital signs	x	x	x	x	x	x
NEWS score	x	x	x	x	x	x
Inflammatory markers (CRP, D-dimer, LDH, ferritin, full blood count)		x	x	x	x	x
PK levels*					x	x
Full blood count[¶]		*Daily while on CYP3A4 inhibitor*				
FiO_2	x	x	x	x	x	x
SpO_2 and/or PaO_2	x	x	x	x	x	x
Randomisation		x				
Weight and Estimated height		x				
Safety monitoring of liver and renal function	x	x	x	x	x	x
Length of stay						
Serious Adverse Events	x	x	x	x	x	x
Concomitant medications	x	x	x	x	x	x
Serum		x		x		x
Plasma		x		x		x
RNA in PAX gene tube		x		x		x
PBMC		x		x		x
Nasosorption biomarkers		x		x		x

[+] data that is routinely collected for clinical purposes will be recorded and a visit is not required
[#] in the event that the patient recovers from COVID19 and is discharged, these visits will not be mandated. Instead a visit should occur on the date of discharge. Where possible, patients will be contacted by telephone at day 7, 14 and 28 and invited to attend for blood tests to monitor resolution of illness.
*Ruxolitinib dose for these patients will be assessed after 10 dialysis patients complete Day 14 through pharmacokinetics (PK) levels which will be assessed.
[¶]For patients randomized to ruxolitinib and prescribed a strong CYP3A4 inhibitor or dual CYP3A4/CYP2C9 inhibitor. Please refer to Section 7.8 of study protocol v1.9 for details.

For peer review only

For peer review only - http://bmjopen.bmj.com/site/about/guidelines.xhtml

1.3 Study population

1.3.1 Eligibility criteria

Inclusion criteria

- Patients age ≥ 18 years at screening.
- Patients meeting criteria of:
  - Hospitalization *AND*
  - SARS-CoV2 infection (clinically suspected[1] or laboratory confirmed) *AND*
  - Radiological change consistent with COVID-19 disease
- Mild or moderate (Grade 3 or 4 severity by modified WHO COVID-19 Ordinal Scale) C19 pneumonia.
- CRP ≥ 30mg/L at any time point.
- Informed consent from patient or personal or professional representative.
- No medical history that might, in the opinion of the responsible clinician, put the patient at significant risk if he/she were to participate in the trial.
- Agreement to abstain from sexual intercourse or use contraception that is >99% effective for all participants of childbearing potential for 42 days.[2]
- For male participants, agreement to abstain from sperm donation for 42 days after the last dose of study drug.[3]
- Non-English speakers will be able to join the study. If patients are unable to understand verbal or written information in English - hospital translation services will be requested at the participating site for the participant where possible.
- Patients normally on non-invasive ventilation such as continuous positive airway pressure (CPAP) at home are eligible.

[1] Clinical suspicion of COVID-19 pneumonia will be confirmed by two independent clinicians.
[2] Females must be either post-menopausal for at least 1 year or surgically sterile; or if female of child-bearing potential, must not be pregnant or lactating and must agree to use an acceptable method of birth control throughout the duration of the trial and for 30 days following the last dose. Acceptable methods of birth control are defined as: hormonal contraception (pill, injection or implant) used consistently for at least 30 days prior to enrollment, intrauterine device (IUD), double-barrier (ie, condom and spermicide, or condom and diaphragm), or true abstinence (when this is in line with the preferred and usual lifestyle of the subject).
[3] Male subject with a partner of childbearing potential must agree to use an appropriate double-barrier method of contraception during the time interval between administration of the first dose of study drug and 30 days following the last dose, or must agree to true abstinence (when this is in line with the preferred and usual lifestyle of the subject).

For peer review only - http://bmjopen.bmj.com/site/about/guidelines.xhtml

Exclusion criteria

- Requiring either invasive or non-invasive ventilation including CPAP or high flow nasal oxygen at any point after hospital admission and before baseline not related to a pre-existing condition (e.g. obstructive sleep apnoea)
- Grade ≥ 5 severity on the WHO COVID-19 Ordinal Scale.
- O_2 saturation < 90% on ≥ 60% inspired oxygen at baseline.
- In the opinion of the investigator, progression to death is inevitable within the next 24 hours, irrespective of the provision of therapy.
- Any medical condition or concomitant medication that in the opinion of the investigator would compromise subjects' safety or compliance with study procedures.
- Any medical condition which in the opinion of the principal investigator would compromise the scientific integrity of the study.
- Known severe allergic reactions to the investigational agents.
- Use of drugs within the preceding 14 days that are known to interact with any study treatment (FOS or RUX), as listed in the Summary of Product Characteristics.
- Child Pugh B or C grade hepatic dysfunction.
- Pregnant or breast feeding.

1.4 Treatment groups

1.4.1 Standard of care

It is accepted that SOC may change during a rapidly evolving pandemic. Co-enrolment to other trials and rescue therapy, either pre- or post-randomisation, is permitted.

1.4.2 Future changes to Standard of Care

The study team will remain cognisant of the prevailing global literature during the current pandemic on effective therapies for COVID-19 pneumonia. If a treatment emerges with efficacy and safety profile that is superior to SOC then options for adoption of the new therapy as SOC will be discussed with the DMC. Specifically, the impact that the new SOC might have on original trial statistical parameters and outcomes measures will be considered noting that SOC is received by participants in all three arms.

For peer review only - http://bmjopen.bmj.com/site/about/guidelines.xhtml

1.4.3 Ruxolitinib

Table 2. Ruxolitinib (RUX)

Period	***Dose***	***Frequency***
Day 1-7	10mg	Twice daily
Day 8-14	5mg	Twice daily

1.4.4 Fostamatinib

Table 3. Fostamatinib (FOS)

Period	***Dose***	***Frequency***
Day 1-7	150mg	Twice daily
Day 8-14	100mg	Twice daily

1.4.5 Treatment duration

The planned duration of treatment is 14 days. Participants may be discontinued from treatment earlier due to unacceptable toxicity or disease progression, which will be recorded as serious adverse events (SAEs). If a participant is discharged prior to day 14 they will go home and continue with the treatment plan.

1.5 Sample size estimation

In the following Stage 1 refers to the formal interim analysis after 171 participants have been recruited and completed 14 days of follow-up. Stage 2 refers to the final analysis when recruitment and follow-up are complete, and it includes all the participants.

171 (57 per arm) patients with COVID-19 pneumonia will be recruited to Stage 1, if the trial progresses to Stage 2 an additional maximum of 285 (95 per arm) will be recruited, resulting in a potential sample size of 456 if three trial arms continue for the whole trial (152 per arm). These numbers have been chosen to provide power of 90% (minimum marginal power) with a maximum 5% chance of an intervention arm being recommended when it provides no improvement over control (5% one-sided family-wise error rate) and has been inflated by 5% for missing outcome data. Patients will be enrolled into RUX, FOS or SOC groups, as shown in figure 1.

The trial is designed to compare two experimental treatment arms to a shared control arm, with equal treatment allocation over two unequally spaced stages. Stage one will be undertaken after 37.5% of maximum sample size have been recruited and completed follow-up. A separate stopping rule will be

For peer review only - http://bmjopen.bmj.com/site/about/guidelines.xhtml

used which means the trial will continue until a decision is made for all experimental treatment arms. The sample size in each stage is allowed to vary according to the number of treatments present. The sample size calculation is based on the calculation in the R Shiny app for multi-stage group-sequential multi-arm clinical trials for a Bernoulli distributed primary outcome found here https://mjgrayling.shinyapps.io/multiarm/ but with an inflation to allow for unequal allocation across stages (0.375 of maximum possible sample in stage 1 and 0.625 of sample in stage 2).

1.5.1 Sample size for formal interim analysis (Stage 1)

Current rate of severe pneumonia in the standard of care is 50%. In the case that an experimental arm reduces this to 30% (relative risk, RR, 0.6), then the design provides a 92% chance (one-sided alpha of 0.25, equivalent to stopping for futility if the one-sided p-value is >0.25) of it being recommended to continue to stage 2.

The design recommends stopping an intervention arm at stage 1 if the one-sided p-value is >0.25 but this is a non-binding rule (i.e. the overall power is calculated assuming this rule is always followed and the family-wise error rate (FWER) is calculated assuming it is not followed). The actual FWER will be lower if the rule is always followed).

Recruitment to the trial will continue during this interim analysis stage.

1.5.2 Sample size for full trial (Stage 2)

If the trial is recommended to continue at Stage 2 based on DMC interim review, the full trial will need to recruit an additional 95 for each arm that continues (152 per arm in total; 456 to the complete trial). This will provide 90% power to detect a change of RR of 0.6 (50% to 30%), with one-sided alpha of 0.027 (chosen to give a 5% FWER) and has been inflated by 5% for missing outcome data. Here the power is the chance of an effective treatment passing stage 1 and being recommended after stage 2.

1.5.3 Informal interim analysis

An informal analysis will take place after 10 participants have been recruited to each arm and followed up for 14 days. No hypothesis testing will be performed, the analysis has been set out in "MATIS SAP I Dose Verification Informal Analysis ver1.0". The DMC will review the data with guidance contained in the SAP I.

For peer review only - http://bmjopen.bmj.com/site/about/guidelines.xhtml

1.6 Randomisation

Patients will be randomized to one of three actively recruiting trial arms, RUX, FOS or SOC. Eligible patients will be allocated using a central web-based randomisation service that uses randomisation sequences with random block sizes that are stratified by age (<65 vs ≥65) and site. If stage 2 occurs with one intervention arm stopped then the randomisation ratio will be 1:1.

1.7 Blinding

The analysis described in this SAP will be undertaken primarily by the trial statistician who will be blind to treatment assignment. The trial statistician will only see sub-group bind data throughout the trial, which means treatment assignment will be coded as group A versus group B versus group C, without knowing which treatments A, B and C refer to. Any analysis of primary efficacy outcomes at the interim analysis stage or data with the potential to unblind the trial statistician (e.g. adherence to allocated intervention) at the interim analysis will be undertaken by an independent statistician from Newcastle University. All final analysis will be undertaken by the blinded statistician who will perform the blinded analysis prior to analysis of any data with the potential to unblind, for example analysis of the adherence data. The senior statistician based in Imperial Clinical Trials Unit will hold the information to unblind the data for closed DMC discussions.

1.8 Brief description of proposed analysis

Efficacy analysis population: We will use the intention-to-treat (ITT) population which includes all randomised participants analysed in the treatment arm to which they were allocated to regardless of treatment subsequently received, multiple imputation will be used to account for any missing data such that all randomised participants are included in the final analysis. At the formal interim analysis multiple imputation will be undertaken if greater than 5% of outcome data are missing, if ≤5% missing a complete case analysis will be undertaken.

Safety analysis population: We will include participants who received at least one dose of study medication and analysis will be according to treatment received, regardless of randomised treatment assignment.

The formal interim analysis will be conducted after 171 participants have been recruited and have completed 14 days of follow-up after randomisation. If an experimental treatment is recommended to continue an additional 95 participants will be recruited for each arm that continues and final analysis will be completed after all participants have completed 28 days of follow-up after randomisation.

For peer review only - http://bmjopen.bmj.com/site/about/guidelines.xhtml

Formal interim and final analyses will be carried out sub-group blind and follow the ITT principle. That is, all randomised patients will be analysed in the treatment arm to which they were allocated to regardless of treatment subsequently received, multiple imputation will be used to include all randomise participants for the final analysis, at the formal interim analysis multiple imputation will be undertaken if greater than 5% of outcome data are missing. The analysis of the serious adverse events will be undertaken on the safety population.

All regression analysis will include adjustment for age group (<65 years or ≥65 years) and site where appropriate, since these were stratification factors in the randomisation.

All confidence intervals will be presented at the 95% level.

For the formal interim analysis (stage 1) one-sided p-values ≤ 0.25 will indicate that an experimental treatment should be recommended to continue to stage 2 and for the final (stage 2) analysis one-sided p-value ≤ 0.027 will be interpreted as statistically significant for the primary outcome.

1.9 Informal interim analysis outcomes - Completed 29th Jan 2021

At the end of the initial stage, the mean CRP values over days 1, 7, 14 and 28 (adjusted for baseline) will be calculated by treatment arm. The mean CRP will also be calculated by treatment arm and visit (adjusted for baseline). These results will form part of the evidence to verify the dose and duration of treatment for the next stage. The pre-specified criteria guiding this inference are listed in full below.

- Dose continues unchanged: For each treatment arm (RUX or FOX) the dose and duration will remain unchanged if there is an absolute mean CRP decrease of approximately 25% or more compared to SOC by day 14 and judged to be maintained by day 28.

- Dose escalation or duration increase: For each treatment arm (RUX or FOX) an adjustment to the dose and duration will be discussed if the mean CRP decrease is less than approximately 25% compared to SOC by day 14.

The results were taken into consideration along with the number of SAEs and SARs and consistency of treatment effect across subgroups including renal failure, use of immunomodulators and age group. Platelet counts and neutrophil counts were also examined over time to monitor the treatment effect in renal failure patients with a dose reduction.

For peer review only - http://bmjopen.bmj.com/site/about/guidelines.xhtml

The Data Monitoring Committee and chief investigator, with contribution from the unblinded senior statistician and independent statistician reviewed the unblinded efficacy data and made a recommendation on the 29th January 2021 to the Trial Steering Committee to continue to the next stage maintaining existing dosing schedule.

For peer review only

For peer review only - http://bmjopen.bmj.com/site/about/guidelines.xhtml

2. Data analysis plan – data description

2.1 Recruitment and participant flow

Please see section 2.1 in SAP I. This will be undertaken for stage I and stage II

2.2 Baseline characteristics

Please see section 2.2 in SAP I. For ease of presentation median and IQRs will be removed from this analysis. This will be undertaken for stage I and stage II

2.3 Co-enrolment

Please see section 2.3 in SAP I. This will be undertaken for stage I and stage II

2.4 Standard of Care

Please see section 2.4 in SAP I. This will be undertaken for stage I and stage II

2.5 Withdrawals, loss to follow-up and other missing data

Please see section 2.5 in SAP I. This will all be undertaken for stage II, only withdrawal summaries will be prepared for Stage I.

2.6 Adherence to allocated treatment and dose summaries

Please see section 2.6 in SAP I. This will be undertaken for stage I and stage II

All analysis of adherence data at the formal interim analysis will be completed by the independent statistician in Newcastle University to ensure the trial statistician remains blind and only DMC members will see these results.

2.7 Descriptive statistics for outcome measures

The proportion of participants with the primary outcome of WHO COVID-19 $\geq$ 5 by day 14 will be summarised by treatment arm and repeated for day 28. This will be undertaken for stage I and stage II.

In addition, the proportion of participants experiencing each of the components of the WHO COVID-19 severity scale score (mortality, invasive ventilation plus organ support (renal replacement therapy, ECMO or inotropic support), invasive ventilation, non-invasive ventilation including CPAP and high

For peer review only - http://bmjopen.bmj.com/site/about/guidelines.xhtml

flow nasal oxygen) at any point up to day 14 (repeated up to day 28) will be summarised by treatment arm. This will be undertaken for stage I and stage II.

Trajectories of mean (SD) grades over time by treatment arm will also be plotted. This will be undertaken for stage II only.

Time spent at each of the components will be summarised as medians and IQRs by treatment arm. This will be undertaken for stage II only.

All stage 1 analysis of the WHO COVID-19 grades will be completed by the independent statistician in Newcastle University to ensure the trial statistician remains blind and only DMC members will see these results.

Inflammatory markers CRP, LDH, ferritin, D-dimer, and serum creatinine values will be summarised by treatment arm over time and presented as means (SD) and median (IQR). This will be undertaken for stage II

Summaries (means (SD) and median (IQR)) of length of stay will be tabulated amongst those that do not withdraw or die. This will be undertaken for stage II

2.8 Serious adverse events

Incidence of venous thromboembolism and serious adverse events will be collected in this study to assess safety.

The proportion of participants experiencing a venous thromboembolism will be summarised by treatment arm and summaries of the time since randomisation will be presented. The number of readmissions will also be extracted from free text in SAE reports and will be summarised by treatment arm.

Serious adverse events coded as per the CTCAE will be summarised at the preferred term level and body system class. Events will be tabulated by treatment arm including information on the number with at least one event and the number of events to account for recurrent events.

Raw data for each SAE will be listed with information on event type, timing duration and outcome.

All above analysis will be undertaken for stage I and stage II

For stage II analysis we will also undertake the following analysis:

For peer review only - http://bmjopen.bmj.com/site/about/guidelines.xhtml

In addition, incident rate ratios for each event between treatment arms (RUX vs SOC or FOS vs SOC) with corresponding 95% CI will be calculated to account for differential follow-up and recurrent events with 95% CIs using a suitable model e.g. a negative binomial model or ZIP as appropriate. These results will be presented in both a table and graphically using a dot plot.

For events where time-to-event analysis is deemed appropriate we will present Kaplan-Meier plots with extended at risk tables for events that occur once within participants and mean cumulative function plots where events can recur within participants.[1, 2]

Blood measurements will be analysed as continuous outcomes calculating a mean difference and 95% CIs over the study follow-up period using a linear mixed effect model and the between arm effect will be plotted over time. Participants will be included as a random intercept with fixed effects for treatment arm, time and baseline outcome values. Stratification variables age and site, plus receipt of known effective treatments dexamethasone and/or tociluzimab, prior vaccination against COVID-19 and receipt of paxlovid and/or molnupiravir at the time of or prior to randomisation into MATIS will also be included as fixed effects. These outcomes will also be categorised as abnormal/normal and analysed using the distributional approach to retain power at day 14 and 28.[3]

For peer review only

For peer review only - http://bmjopen.bmj.com/site/about/guidelines.xhtml

3. Data analysis plan - Inferential analysis

3.1 Primary outcome of the main study - Modified WHO grading

3.1.1 Definition of the primary outcome

The proportion of participants progressing from mild to severe COVID-19 pneumonia within 14 days. Where severe COVID-19 pneumonia is defined by a modified WHO COVID-19 Ordinal Score ≥ 5 (on the 9 point scale), comprising the following indicators of disease severity: Death (9) *OR* Requirement for invasive ventilation plus organ support (8) Requirement for invasive ventilation (7) *OR* Requirement for non-invasive ventilation including CPAP or high flow oxygen (6) *OR* O_2 saturation < 90% on ≥60% inspired oxygen (5). These are captured in the database as follows:

Modified WHO COVID-19 Ordinal Score	Database
(9) Death	
(8) Requirement for invasive ventilation plus organ support	Has the patient required renal replacement therapy? OR Has the patient required ECMO? OR Has the patient required inotropic support? AND Has the patient required intubation and mechanical ventilation?
(7) Requirement for invasive ventilation	Has the patient required intubation and mechanical ventilation?
(6) Requirement for non-invasive ventilation including CPAP or high flow oxygen	Has the patient required NIV, CPAP or high flow nasal oxygen?
(5) O_2 saturation < 90% on ≥60% inspired oxygen	Has the patient's oxygen saturation been < 90% on 60% inspired oxygen?

3.1.2 Statistical model at stage 1

The stage one primary analysis will consist of fitting two separate binomial regression models for the RUX vs SOC and FOS vs SOC comparisons in order that we can undertake an adjusted analysis using a propensity score approach. Specifically a generalized linear model with WHO COVID-19 status (<5 or ≥5) as the outcome and treatment group as a covariate. We will adjust the treatment effect for the

For peer review only - http://bmjopen.bmj.com/site/about/guidelines.xhtml

following prespecified covariates using a propensity score approach. The propensity score model will include:

- baseline WHO COVID-19 score (3 or 4)
- age (<65 or ≥65)
- receipt of dexamethasone (prior randomisation or up to 1 day post randomisation)
- receipt of tociluzimab (prior randomisation or up to 1 day post randomisation)

In the event that all participants are on one of the effective treatments that covariate will be removed from the propensity score. We will adjust using the propensity score from a logistic regression model including the above covariates. We will also fit an unadjusted generalized linear model with WHO COVID-19 status (<5 or ≥5) as the outcome and treatment group as a covariate.

The models will be fitted with a binomial distribution and logit link function and the treatment effect will be reported as an odds ratio with corresponding 95% confidence interval and p-values. P-values will be one-sided and judged at the 0.25 level. Given that the number of sites is likely to be small we will not include site as a covariate in this stage 1 analysis.

Every effort will be made to obtain follow-up data for all participants including those that stop treatment. The number and proportion of participants missing WHO COVID-19 status by treatment group and visit will be tabulated (see section 2.5 above). For participants who are discharged early the assumption will be made that they did not progress to grade 5 WHO scale. If there is a >5% rate of missing outcomes after this, we will impute missing WHO COVID-19 data in the primary analysis model using multiple imputation under the assumption of missing at random (MAR). That is the probability of being missing is not dependent on the values of unobserved data, conditional on the observed values of the variables included in the analysis model. Imputation under MAR using chained equations following the guidance of White et al. will be undertaken.[4] Variables in the imputation model will include the variables in the primary analysis model described above plus baseline WHO COVID-19 score (3 or 4), age (<65 or ≥65), receipt of dexamethasone and/or tociluzimab at the time of or prior to randomisation into MATIS. Additional auxiliary variables that are suspected to be associated with the outcome and are predictors of missingness will also be included. These are receipt of any effective treatments post randomisation as a result of co-enrolment when occurring post-randomisation, and receipt of known effective treatments dexamethasone and tociluzimab post-randomisation (>1 day post randomisation) and WHO COVID-19 scores at day 1 and day 7. Using White et al.'s rule of thumb we will impute m datasets based on the percentage of participants with incomplete outcomes, where m will be rounded to the nearest 10, for example if 8% of participants have incomplete primary outcome then m=10 will be used.

For peer review only - http://bmjopen.bmj.com/site/about/guidelines.xhtml

Before undertaking the multiple imputation as described above we will impute any missing baseline data deterministically only using other baseline data, this includes the baseline WHO COVID-19 status, age, receipt of dexamethasone and/or tociluzimab at the time of or prior to randomisation into MATIS. To do this we will impute missing values with the mean values of observed data. In line with White et al., outcomes will be imputed separately for each treatment group.

Imputed datasets will then be analysed using the primary analysis model described above.

A sensitivity analysis on the primary analysis will be undertaken if greater than 5% of outcome data are missing to examine the missing at random assumption. We will examine extreme examples assuming 1) that all participants with missing outcome had poor outcome e.g. all missing >=5, and 2) that all participants with missing outcome had good outcome e.g. all missing <5.

For peer review only

i. Compliance

Adherence to assigned treatment (RUX and FOS) will be calculated for each participant as the proportion of dose received per day of the prescribed dose per day and presented as the percentage received. This will be summarised by treatment group as described in section 2.6 above.

To explore the impact of compliance with the planned treatment protocol, we will undertake a per-protocol analysis to examine the treatment effect in those that take most of the treatment. We will define 'per-protocol' population as individuals who receives ≥ 90% of prescribed dose and refit the primary analysis model on this population.

ii. Time of enrolment

An exploratory analysis of the primary outcome will be undertaken in order to assess the impact of time of enrolment into the MATIS study. A categorical variable will be created by splitting the recruitment period into quartiles such that participants are grouped into approximately four equal sized groups based on time of enrolment into the MATIS study. A crude odds ratio will be calculated for each quartile.

All stage 1 analysis of the WHO COVID-19 grades will be completed by the independent statistician in Newcastle University to ensure the trial statistician remains blind and only DMC members will see these results.

For peer review only - http://bmjopen.bmj.com/site/about/guidelines.xhtml

3.1.3 Statistical model at stage 2

The stage two primary analysis will consist of fitting one binomial regression model regardless of whether one or two experimental treatments progress from stage one. Specifically a generalized linear model with WHO COVID-19 status (<5 or ≥5) as the outcome and treatment group, baseline WHO COVID-19 score (3 or 4), age (<65 or ≥65), receipt of known effective treatments dexamethasone and/or tociluzimab, prior vaccination against COVID-19 (y/n) and receipt of paxlovid and/or molnupiravir at the time of or prior to randomisation into MATIS as covariates will be fitted. Effective treatments will be included as separate covariates in the primary analysis model. Given the anticipated small number of sites in this study, site will also be included in the model as a fixed effect.[5] In the event that all participants are on one of the effective treatments that covariate will be removed from the model.

If including site as a fixed effect results in unstable model estimates e.g. if there are a number of sites with very few randomisations we will exclude site from the model and we will only adjust for baseline WHO COVID score, age group, receipt of known effective treatments dexamethasone and/or tociluzimab, prior vaccination against COVID-19, and receipt of paxlovid and/or molnupiravir.[6] We will also consider not adjusting for paxlovid and/or molnupiravir if the number of participants receiving these are low. If after removing sites and treatments the model is not stable due to event rate:covariate ratio we will adjust for all covariates using a propensity score approach.

The model will be fitted with a binomial distribution and logit link function and the treatment effect will be reported as an odds ratio with corresponding 95% confidence interval and p-values. P-values will be one-sided and judged at the 0.027 level. Therefore, a two-sided p-value < 0.054 will be used to indicate a significant treatment effect.

The stage two model to be fitted to estimate the treatment effects will be:

For peer review only - http://bmjopen.bmj.com/site/about/guidelines.xhtml

$$logit(\pi_{ij}) = \beta_0 + \beta_1 RUX_{ij} + \beta_2 FOS_{ij} + \beta_3 WHO^0_{ij} + \beta_4 AGE^0_{ij} + \beta_5 DEX^0_{ij} + \beta_6 TOC^0_{ij} + \beta_7 VAC^0_{ij} + \beta_8 PAX^0_{ij} + \beta_9 MOL^0_{ij} + \beta_{10}\, SITE_1 + \beta_{11}\, SITE_2 + \cdots + \beta_{12}\, SITE_{j-1}$$

RUX_{ij}: dummy variable for treatment ($RUX_{ij} = 0\ or\ 1$) for participant i in j[th] site

FOS_{ij}: dummy variable for treatment ($FOS_{ij} = 0\ or\ 1$) for participant i in j[th] site

WHO^0_{ij}: dummy variable for baseline WHO COVID-19 value for participant i in j[th] site ($WHO_{ij} = 0\ or\ 1\ where\ 0\ indicates\ score\ of\ 3\ and\ 1\ indicates\ score\ of\ 4$)

AGE^0_{ij}: dummy variable for age-group ($AGE_{ij} = 0\ or\ 1\ where\ 0\ indicates < 65\ years\ and\ 1\ indicates\ \geq 65$) for participant i

DEX^0_{ij}: dummy variable for receipt of dexamethasone at the time of or prior to randomisation (includes day of randomisation or one day post randomisation) ($DEX_{ij} = 0\ or\ 1$) for participant i

TOC^0_{ij}: dummy variable for receipt of tociluzimab at the time of or prior to randomisation (includes day of randomisation or one day post randomisation) ($TOC_{ij} = 0\ or\ 1$) for participant i

VAC^0_{ij}: dummy variable for prior vaccination against COVID-19 at the time of or prior to randomisation (includes day of randomisation or one day post randomisation) ($VAC_{ij} = 0\ or\ 1$) for participant i

PAX^0_{ij}: dummy variable for receipt of paxlovid at the time of or prior to randomisation (includes day of randomisation or one day post randomisation) ($PAX_{ij} = 0\ or\ 1$) for participant i

MOL^0_{ij}: dummy variable for receipt of molnupiravir at the time of or prior to randomisation (includes day of randomisation or one day post randomisation) ($MOL_{ij} = 0\ or\ 1$) for participant i$Site_j$: dummy variable for each of the jth sites

π_{ij}: probability of WHO COVID-19 ≥5 for the ith participant in j[th] site

To account for missing outcomes in the primary analysis model we will impute missing WHO COVID-19 status using multiple imputation under the assumption of missing at random (MAR) as described in the primary statistical model at stage 1 section 3.1.2 plus the imputation model will contain the following additional variables: prior vaccination against COVID-19, and receipt of paxlovid and/or molnupiravir at the time of or prior to randomisation into MATIS, plus post-randomisation receipt of paxlovid and/or molnupiravir. Multiple imputation will be used for the final analysis regardless of levels of missingness at this stage.

For peer review only - http://bmjopen.bmj.com/site/about/guidelines.xhtml

As well as reporting odds ratios and 95% CIs, the model will be used to estimate the difference in proportion of participants with modified WHO COVID-19 Ordinal Score ≥ 5.

3.1.4 Model assessment of stage 2 analysis

The primary analysis model will be assessed for model fit and outlying or influential observations as per methods described below.

To determine the fit of the model by using summary measures of goodness of fit and by assessing the estimation ability of the model:

i. Perform the goodness-of-fit test using the Hosmer-Lemeshow test to look at the discrepancy between the observed and estimated frequency of outcomes (Stata command *estat gof*).
ii. Examination of the ROC curve (Stata command *lroc*) to assess the correct identification of outcome based on varying probability thresholds.
iii. Plot of the Pearson's standardised residuals (*predict.., rstand*) against predicted values (*predict..*). Plots showing trends suggest poor model fit or misspecification of model.

If model assumptions are deemed to be invalid or the model appears to be miss-specified, we will undertake a sensitivity analysis with an alternative model.

To determine if there are any observations that do not fit the model or have a large influence on the model:

i. Check for outliers/predicted values that have poor fit by plotting deviance residuals (*predict .., deviance*) against predicted values (*predict ..*); standardised Pearson's residuals (*predict…, rstand*) against predicted; and leverage (predict .., hat) against predicted.
ii. Investigate whether the extreme outlying observations have been recorded correctly and if so, compare model coefficient estimates with and without the identified influential observations
iii. Use Cook's distance (stata command *predict.., cook*) to identify influential observations

For peer review only - http://bmjopen.bmj.com/site/about/guidelines.xhtml

3.1.5 Stage 2 exploratory analysis for primary outcome

i. Co-enrolment at time of randomisation

An exploratory analysis of the primary outcome will be undertaken in order to assess the impact of co-enrolment to an 'active'* arm of another study at the time of or prior to randomisation into MATIS (includes day of randomisation or one day post randomisation). Individual treatments will be included as separate covariates in the primary analysis model described above. In the event of low event rates the sensitivity analysis will be repeated removing one covariate at a time.

*We will only consider those co-enrolled to an 'active' arm of another study. Co-enrolment to the standard of care arm in other studies will not be considered an active treatment.

ii. Co-enrolment post randomisation and receipt of rescue medication

We will explore what the direct effect of the treatment is on the outcome, and whether there is an indirect effect of receipt of active treatment as a result of co-enrolment when occurring post-randomisation, and receipt of known effective treatments dexamethasone, tociluzimab, paxlovid and molnupiravir post-randomisation (>1 day post randomisation). Where post-randomisation co-enrolment or receipt of known effective treatments must occur prior to the occurrence of the outcome. We will do this via a mediation analysis using a structural equation modelling approach using the Stata *paramed* command.[7, 8]

The analysis will allow us to decompose the total observed treatment effect into mediated (indirect, described by the natural indirect effect in the Stata output) and non-mediated (direct, described by the natural direct (and controlled direct effect) effect estimate in Stata output) components. Each post-randomisation active intervention and known effective treatments will be treated as an individual mediator, thus we will estimate the mediated effect separately for each. Analysis models will adjust for baseline WHO COVID-19 score (3 or 4), age (<65 or ≥65), receipt of known effective treatments dexamethasone and/or tociluzimab, prior vaccination against COVID-19, and receipt of paxlovid and/or molnupiravir at the time of or prior to randomisation into MATIS and site as fixed as per the primary analysis model described in section 3.1.3. The model will be modified in a similar manner as the primary outcome modelling in the event of low event rates.

For peer review only - http://bmjopen.bmj.com/site/about/guidelines.xhtml

iii. Compliance

We will undertake an exploratory analysis to examine the treatment effect (reported as a risk ratio (RR)) adjusted for compliance. This will be achieved through a complier adjusted case (CACE) analysis using a two-stage residual inclusion estimator (2SRI) approach with randomisation group as the instrumental variable. The primary analysis model described in 3.1.3 will be refitted with the log link function (instead of logit function) to give the treatment effect as a RR with corresponding 95% confidence interval and p-values. The CACE will then be estimated for this model and we will compare the CACE estimates of the RR to the RR obtained from the refitted primary analysis model. Randomisation meets the criteria for an adequate instrument variable since (i) randomisation predicts the treatment receipt, (ii) randomisation is unconfounded with the outcome and we assume (iii) no direct effect of randomisation on the outcome (other than via treatment receipt) – *the exclusion restriction.* After the second stage of estimation, the standard errors of the estimates will be corrected using the method of Terza.[9, 10]

This approach can only include adherence information in the intervention groups as the equivalent usage in the control participants is not recorded (as the control intervention is SOC and can differ between participants and is likely to evolve with time). SOC will be assumed to be 100% compliant with prescribed treatment.

We will define a complier as an individual who receives ≥ 90% of prescribed dose. In addition to the analysis using a 90% threshold to define compliance we will undertake alternative analysis defining a ‘complier’ as an individual who receives ≥ 60%, ≥ 70% and ≥ 80% of prescribed dose to explore the impact of alternative definitions of compliance.

Analysis will adjust for baseline WHO COVID-19 score (3 or 4), age (<65 or ≥65)), receipt of known effective treatments dexamethasone and/or tociluzimab, prior vaccination against COVID-19, and receipt of paxlovid and/or molnupiravir at the time of or prior to randomisation into MATIS and site as fixed as per the primary analysis model described in section 3.1.3. The model will be modified in a similar manner as the primary outcome modelling in the event of low event rates.

iv. Time of enrolment

An exploratory analysis of the primary outcome will be undertaken in order to assess the impact of time of enrolment. A categorical variable will be created by splitting the recruitment period into quartiles such that participants are grouped into approximately four equal sized groups based on time of enrolment into the MATIS study. This variable will then be included as a covariate in the primary analysis model described above as a treatment group-by-time interaction term to assess the impact of time of recruitment on the treatment effect estimate.

For peer review only - http://bmjopen.bmj.com/site/about/guidelines.xhtml

3.1.6 Stage 2 subgroup analysis for primary outcome

Subgroup analysis will be performed for the primary outcome for the final analysis only to explore the uniformity of the treatment effect. The following subgroups will be examined by adding a treatment-by-subgroup interaction term to the same analysis model as for the primary outcome:

- Sex: male vs female;
- Age: <65 years vs ≥65 years
- Obesity: <25, 25-29.9, ≥30
- Chronic heart disease: yes vs no;
- Lung disease: yes vs no;
- Kidney disease: yes vs no;
- Liver disease: yes vs no;
- Diabetes: yes vs no;
- Immunocompromised: yes vs no;
- Smoking status: current vs non-smoker;
- CRP > 200mg/L: yes vs no and/or D-dimer >2500mg/mL: yes vs no;
- Time since onset of symptoms:0-5, 6-10, >10 days prior to randomisation

P-values for each interaction term will be presented. No adjustment for multiple tests will be made and the results will be viewed as hypothesis generating only. The consistency of estimates will be depicted visually by means of a forest plot.

3.2 Secondary outcomes of the main study

Analysis of the secondary outcomes listed below will be undertaken for the final analysis only. Each of the outcomes will be examined at 14 and 28 days and comparisons will be between RUX or FOS versus SOC:

- Change in severity on the modified WHO COVID-19 Ordinal Scale (3 or 4 to 5,6,7,8,9)
- Death (yes/no)
- Invasive ventilation (yes/no)
- ECMO (yes/no)
- Non-invasive ventilation including CPAP and high flow nasal oxygen (yes/no)
- Renal replacement therapy (yes/no)
- Venous thromboembolism (yes/no)
- Inflammatory markers CRP, LDH, ferritin, D-dimer (units)

For peer review only - http://bmjopen.bmj.com/site/about/guidelines.xhtml

- Length of stay (days)
- Serum creatinine

3.2.1 Severe COVID-19 within 28 days

The proportion of participants progressing from mild to severe COVID-19 pneumonia within 28 days will be analysed using the main analysis model as described in section 3.1.3, replacing the 14 day outcome with the 28 day outcome.

3.2.2 Modified WHO COVID-19 score

A mixed effects ordered logistic regression will be used to estimate the odds ratio of disease severity as measured by the modified WHO COVID-19 (ordinal scale) over time by treatment arm. Specifically a generalized linear model with modified WHO COVID-19 status (scores 3/4 through to 9) as the outcome (ordered categorical variable) with S ordinal categories s (s=3/4, 5, 6, 7, 8, 9) and treatment group, time, time-by-treatment arm interaction, baseline WHO COVID-19 score (3 or 4), age (<65 or ≥65), receipt of known effective treatments dexamethasone and/or tociluzimab, prior vaccination against COVID-19, and receipt of paxlovid and/or molnupiravir at the time of or prior to randomisation into MATIS as covariates will be fitted. Participants will be included as a random intercept (ζ_{ij}). Given the anticipated small number of sites in this study, site will be included in the model as a fixed effect.[5] The model will be fitted using the *ologit* link function (or the *meologit* command) in Stata and the treatment effect will be reported as an odds ratio with corresponding 95% confidence intervals.

The model to be fitted will be:

$$logit\big(pr(\pi_{ij} > s)\big) = \beta_1 RUX_{ij} + \beta_2 FOS_{ij} + \beta_3 WHO^0_{ij} + \beta_4 AGE^0_{ij} + \beta_5 DEX^0_{ij} + \beta_6 TOC^0_{ij} + \beta_7 VAC^0_{ij} + \beta_8 PAX^0_{ij} + \beta_9 MOL^0_{ij} + \beta_{10}\, SITE_1 + \beta_{11}\, SITE_2 + \cdots + \beta_{12}\, SITE_{j-1} + \beta_{13} time_7 + \beta_{14} time_{14} + \beta_{15} RUX_{ij} * time_7 + \beta_{16} RUX_{ij} * time_{14} + \beta_{17} FOS_{ij} * time_7 + \beta_{18} FOS_{ij} * time_{14} + \zeta_{ij} - \kappa_s$$

RUX_{ij}: dummy variable for treatment ($RUX_{ij} = 0\ or\ 1$) for participant i in j[th] site

FOS_{ij}: dummy variable for treatment ($FOS_{ij} = 0\ or\ 1$) for participant i in j[th] site

WHO^0_{ij}: dummy variable for baseline WHO COVID-19 value for participant i in j[th] site ($WHO_{ij} = 0\ or\ 1\ where\ 0\ indicates\ score\ of\ 3\ and\ 1\ indicates\ score\ of\ 4$)

For peer review only - http://bmjopen.bmj.com/site/about/guidelines.xhtml

AGE_{ij}^0: dummy variable for age-group ($AGE_{ij} = 0\ or\ 1\ where\ 0\ indicates <$ $65\ years\ and\ 1\ indicates\ \geq 65$) for participant i

DEX_{ij}^0: dummy variable for receipt of dexamethasone at the time of or prior to randomisation (includes day of randomisation or one day post randomisation) ($DEX_{ij} = 0\ or\ 1$) for participant i

TOC_{ij}^0: dummy variable for receipt of toczulimab at the time of or prior to randomisation (includes day of randomisation or one day post randomisation) ($TOC_{ij} = 0\ or\ 1$) for participant i

VAC_{ij}^0: dummy variable for prior vaccination against COVID-19 at the time of or prior to randomisation (includes day of randomisation or one day post randomisation) ($VAC_{ij} = 0\ or\ 1$) for participant i

PAX_{ij}^0: dummy variable for receipt of paxlovid at the time of or prior to randomisation (includes day of randomisation or one day post randomisation) ($PAX_{ij} = 0\ or\ 1$) for participant i

MOL_{ij}^0: dummy variable for receipt of molnupiravir at the time of or prior to randomisation (includes day of randomisation or one day post randomisation) ($MOL_{ij} = 0\ or\ 1$) for participant i

$Site_j$: dummy variable for each of the jth sites

$time_x$: dummy variable for time (= 0 or 1) at time point x days. Day 1 is represented by $time_7 = 0$ and $time_{14} = 0$

$\zeta_{ij} \sim N(0, \sigma^2)$: participant specific random intercept

κ_s: indicates the $\kappa_1, \kappa_{2,....}\kappa_{S-1}$ cut-points for each of the possible S outcomes of the WHO COVID-19 ordinal scale

$\left(pr(\pi_{ij} > s)\right)$: cumulative probability of the response being in a category higher than s

π_{ij}: is the odds ratio of more severe versus less severe illness (regardless of where the ordinal scale is cut)

The model will be modified in a similar manner as the primary outcome modelling in the event of low event rates.

This analysis will be repeated on the original WHO COVID-19 (ordinal scale).

3.2.3 Components of the modified WHO COVID-19 score

The following outcomes will be examined at day 14 and day 28 by replacing the primary outcome in the main analysis model as described in section 3.1.3:

- Death (yes/no)
- Requirement for invasive ventilation plus organ support (yes/no)
- Invasive ventilation (yes/no)

For peer review only - http://bmjopen.bmj.com/site/about/guidelines.xhtml

- Non-invasive ventilation including CPAP and high flow nasal oxygen (yes/no)
- Renal replacement therapy (yes/no)
- Venous thromboembolism (yes/no)

Each model will be fitted on complete cases and no imputation of missing values or sensitivity analysis will be undertaken.

3.2.4 Length of stay

The time to discharge will be summarised by treatment arm using a Kaplan-Meier plot with extended at risk tables, detailing the numbers at risk, censored and discharged at each discrete time point, censoring at the day 28 visit. Withdrawals and deaths will be censored at the date of occurrence. In addition, summaries (means (SD) and median (IQR)) of length of stay will be tabulated amongst those that do not withdraw or die.

In addition, a Cox proportional hazards model will be used to estimate the hazard ratio of time to discharge between intervention arms with age (<65 or ≥65), site and receipt of known effective treatments dexamethasone and/or tociluzimab and vaccination status at the time of or prior to randomisation into MATIS included as covariates.

3.2.5 Continuous laboratory values

For inflammatory markers of CRP, LDH, ferritin, D-dimer and serum creatinine levels a linear mixed effect model will be used to estimate the mean difference in values between intervention arms (RUX and FOS) and SOC at each follow-up visit (day 1, day 7 and day 14).[11] Participants will be included as a random intercept with fixed effects for time, time-by-treatment arm interaction and baseline values. Stratification variables age and site, plus receipt of known effective treatments dexamethasone and/or tociluzimab, prior vaccination against COVID-19, and receipt of paxlovid and/or molnupiravir at the time of or prior to randomisation into MATIS will also be included as fixed effects. Baseline adjusted treatment group differences with 95% confidence intervals will be presented for each time-point.

For example for CRP the model to be fitted to estimate the treatment effect at each visit up to day 14 will be:

For peer review only - http://bmjopen.bmj.com/site/about/guidelines.xhtml

$$
\begin{aligned}
Y_{ijt} = \beta_0 + \beta_1 RUX_{ij} + \beta_2 FOS_{ij} + \beta_3 CRP_{ij}^0 + \beta_4 AGE_{ij}^0 + \beta_5 DEX_{ij}^0 \\
+ \beta_6 TOC_{ij}^0 + \beta_7 VAC_{ij}^0 + \beta_8 PAX_{ij}^0 + \beta_9 MOL_{ij}^0\ \beta_{10} time_7 \\
+ \beta_{11} time_{14} + \beta_{12} RUX_{ij} * time_7 + \beta_{13} RUX_{ij} * time_{14} \\
+ \beta_{14} FOS_{ij} * time_7 + \beta_{15} FOS_{ij} * time_{14} + \beta_{16}\ SITE_1 \\
+ \beta_{17}\ SITE_2 + \cdots + \beta_{18}\ SITE_{j-1} + b_{1,i} + e_{it}
\end{aligned}
$$

for

t = 1 to 3 time points (day 1, 7 and 14), i = 1 to 30 participants

RUX_{ij}: dummy variable for treatment ($RUX_{ij} = 0\ or\ 1$) for participant i

FOX_{ij}: dummy variable for treatment ($FOS_{ij} = 0\ or\ 1$) for participant i

CRP_{ij}^0: baseline CRP value for participant i

DEX_{ij}^0: dummy variable for receipt of dexamethasone at the time of or prior to randomisation (includes day of randomisation or one day post randomisation) ($DEX_{ij} = 0\ or\ 1$) for participant i

TOC_{ij}^0: dummy variable for receipt of tociluzimab at the time of or prior to randomisation (includes day of randomisation or one day post randomisation) ($TOC_{ij} = 0\ or\ 1$) for participant i

VAC_{ij}^0: dummy variable for prior vaccination against COVID-19 at the time of or prior to randomisation (includes day of randomisation or one day post randomisation) ($VAC_{ij} = 0\ or\ 1$) for participant i

PAX_{ij}^0: dummy variable for receipt of paxlovid at the time of or prior to randomisation (includes day of randomisation or one day post randomisation) ($PAX_{ij} = 0\ or\ 1$) for participant i

MOL_{ij}^0: dummy variable for receipt of molnupiravir at the time of or prior to randomisation (includes day of randomisation or one day post randomisation) ($MOL_{ij} = 0\ or\ 1$) for participant i

$time_x$: dummy variable for time (= 0 or 1) at time point x days. Day 1 is represented by $time_7 = 0$ and $time_{14} = 0$

$Site_j$: dummy variable for each of the jth sites

$b_{1,i} \sim N(0, \sigma_{b1}^2)$, $e_{it} \sim N(0, \sigma_e^2)$

Where $b_{1,i}$ is the random intercept at the participant level. Each of $b_{1,i}$ and e_{it} are assumed to follow normal distributions. An unstructured covariance matrix will be used and the model will be fitted using restricted maximum likelihood (REML). This model makes assumptions about random effects distributions, correlation structures and residuals, which will all be investigated. If the model fails to converge model estimates will not be presented.

The above will be repeated for each inflammatory marker up to day 14 and additionally up to day 28.

For peer review only - http://bmjopen.bmj.com/site/about/guidelines.xhtml

For peer review only

For peer review only - http://bmjopen.bmj.com/site/about/guidelines.xhtml

References

1. Morris TP, Jarvis CI, Cragg W, et al. Proposals on Kaplan–Meier plots in medical research and a survey of stakeholder views: KMunicate. *BMJ Open* 2019; 9: e030215. DOI: 10.1136/bmjopen-2019-030215.
2. Siddiqui O. Statistical methods to analyze adverse events data of randomized clinical trials. *Journal of Biopharmaceutical Statistics* 2009; 19: 889-899. DOI: http://dx.doi.org/10.1080/10543400903105463.
3. Chis Ster A, Phillips R, Sauzet O, et al. Improving analysis practice of continuous adverse event outcomes in randomised controlled trials - a distributional approach. *Trials* 2021; 22: 419. DOI: 10.1186/s13063-021-05343-0.
4. White IR, Royston P and Wood AM. Multiple imputation using chained equations: Issues and guidance for practice. *Statistics in Medicine* 2011; 30: 377-399. DOI: https://doi.org/10.1002/sim.4067.
5. Kahan BC. Accounting for centre-effects in multicentre trials with a binary outcome – when, why, and how? *BMC Medical Research Methodology* 2014; 14: 20. DOI: 10.1186/1471-2288-14-20.
6. Kahan BC and Morris TP. Improper analysis of trials randomised using stratified blocks or minimisation. *Stat Med* 2012; 31: 328-340. 2011/12/06. DOI: 10.1002/sim.4431.
7. Valeri L and Vanderweele TJ. Mediation analysis allowing for exposure-mediator interactions and causal interpretation: theoretical assumptions and implementation with SAS and SPSS macros. *Psychological methods* 2013; 18: 137-150. 2013/02/04. DOI: 10.1037/a0031034.
8. Liu H, Emsley R, Dunn G, et al. paramed: A command to perform causal mediation analysis using parametric models. 2014.
9. Cook JA, MacLennan GS, Palmer T, et al. Instrumental variable methods for a binary outcome were used to informatively address noncompliance in a randomized trial in surgery. *J Clin Epidemiol* 2018; 96: 126-132. 2017/11/22. DOI: 10.1016/j.jclinepi.2017.11.011.
10. Terza JV. Simpler Standard Errors for Two-stage Optimization Estimators. *The Stata Journal* 2016; 16: 368-385. DOI: 10.1177/1536867x1601600206.
11. Twisk J, Bosman L, Hoekstra T, et al. Different ways to estimate treatment effects in randomised controlled trials. *Contemporary Clinical Trials Communications* 2018; 10: 80-85. DOI: https://doi.org/10.1016/j.conctc.2018.03.008.

For peer review only

For peer review only - http://bmjopen.bmj.com/site/about/guidelines.xhtml

Supplementary Appendix 2

Characteristics of participants in MATIS stratified by predominant COVID-19 variant at time of randomisation

For peer review only

Table S1: Baseline characteristics of all participants by predominant variant at time of randomisation

Patient characteristic, n(%) unless otherwise specified	Alpha (up to May 2021) (N=112)	Delta (May to Dec 2021) (N=61)	Omicron (post Dec 2021) (N=12)
Age in years			
Mean (SD)	60.8 (14.7)	55.9 (16.6)	65.8 (17.1)
Sex			
Male	79 (70.5)	43 (70.5)	6 (50.0)
Female	33 (29.5)	18 (29.5)	6 (50.0)
Ethnicity			
White	41 (36.6)	30 (49.2)	8 (66.7)
Mixed or multiple ethnic groups	1 (0.9)	1 (1.6)	0 (0.0)
Asian or Asian British	14 (12.5)	8 (13.1)	4 (33.3)
Black, Black British, Caribbean or African	12 (10.7)	7 (11.5)	0 (0.0)
Other ethnic group	44 (39.3)	15 (24.6)	0 (0.0)
BMI in kg/m2			
N (N missing)	106 (6)	55 (6)	11 (1)
Mean (SD)	30.2 (6.4)	31.0 (7.8)	26.5 (5.1)
Severity of Covid			
Grade 3	17 (15.2)	13 (21.3)	3 (25.0)
Grade 4	95 (84.8)	48 (78.7)	9 (75.0)
Chronic lung disease			
N (N missing)	111 (1)	60 (1)	12 (0)
Yes	15 (13.5)	13 (21.7)	2 (16.7)
Time from onset of symptoms			
N (N missing)	103 (9)	51 (10)	11 (1)
Mean (SD)	9 (4)	10 (4)	12 (5)
Diabetes			
N (N missing)	111 (1)	60 (1)	12 (0)
Yes	37 (33.3)	13 (21.7)	4 (33.3)
Hypertension			
N (N missing)	111 (1)	60 (1)	12 (0)
Yes	53 (47.7)	21 (35.0)	7 (58.3)
Ischaemic heart disease			
N (N missing)	111 (1)	60 (1)	12 (0)
Yes	17 (15.3)	9 (15.0)	5 (41.7)
Heart failure			
N (N missing)	111 (1)	60 (1)	12 (0)
Yes	3 (2.7)	2 (3.3)	2 (16.7)
Immunocompromised			
N (N missing)	111 (1)	60 (1)	12 (0)
Yes	4 (3.6)	2 (3.3)	2 (16.7)
End-stage renal failure			
N (N missing)	111 (1)	60 (1)	12 (0)
Yes	10 (9.0)	2 (3.3)	1 (8.3)
Liver cirrhosis			
N (N missing)	111 (1)	60 (1)	12 (0)
Yes	0 (0.0)	0 (0.0)	0 (0.0)
Current smoker			

For peer review only

N (N missing)	110 (2)	60 (1)	12 (0)
Yes	5 (4.5)	0 (0.0)	0 (0.0)
Prior Covid Vaccination			
N (N missing)	112 (0)	61 (0)	12 (0)
Yes	9 (8.0)	20 (32.8)	6 (50.0)
Serum creatinine (µmol/L)			
N (N missing)	111 (1)	59 (2)	12 (0)
Mean (SD)	139 (209)	95 (85)	115 (112)
C-reactive protein (mg/L)			
N (N missing)	111 (1)	59 (2)	12 (0)
Mean (SD)	115 (70)	100 (75)	76 (46)
Lactate dehydrogenase (IU/L)			
N (N missing)	73 (39)	28 (33)	8 (4)
Mean (SD)	478 (255)	388 (153)	390 (173)
Ferritin (µg/L)			
N (N missing)	98 (14)	43 (18)	10 (2)
Mean (SD)	1659 (2343)	1235 (1145)	472 (285)
D-dimer (ng/ml)			
N (N missing)	103 (9)	49 (12)	9 (3)
Mean (SD)	1851 (3302)	996 (764)	1391 (1082)

For peer review only

For peer review only

Supplementary Appendix 3

Standard of care treatments received pre- and post-randomisation

Table S2: Selected immunological therapies at or prior to randomisation by treatment arm

Drug	FOS (N=58)	RUX (N=64)	SOC (N=63)
Dexamethasone			
Received ≥1 dose, n (%)	56 (96.6)	57 (89.1)	55 (87.3)
Median (IQR) time since initiation (days); n missing	1 (0, 1); 0	1 (1, 2); 0	1 (0, 1); 0
Median (IQR) duration (days), n missing	7 (5, 9); 0	9 (5, 13); 0	8 (5, 10); 1
Prednisolone			
Received ≥1 dose, n (%)	0 (0.0)	1 (1.6)	1 (1.6)
Median (IQR) time since initiation (days); n missing	N/A	1 (1, 1); 0	1 (1, 1); 0
Median (IQR) duration (days), n missing	N/A	6 (6, 6); 0	1 (1, 1); 0
Methylprednisolone			
Received ≥1 dose, n (%)	0 (0.0)	0 (0.0)	0 (0.0)
Median (IQR) time since initiation (days); n missing	N/A	N/A	N/A
Median (IQR) duration (days), n missing	N/A	N/A	N/A
Hydrocortisone			
Received ≥1 dose, n (%)	0 (0.0)	1 (1.6)	0 (0.0)
Median (IQR) time since initiation (days); n missing	N/A	1 (1, 1); 0	N/A
Median (IQR) duration (days), n missing	N/A	1 (1, 1); 0	N/A
Tocilizumab			
Received ≥1 dose, n (%)	9 (15.5)	8 (12.5)	7 (11.1)
Median (IQR) time since initiation (days); n missing	0 (0, 1); 0	0 (0, 1); 0	0 (0, 1); 0
Median (IQR) duration (days), n missing	1 (1, 1); 0	1 (1, 1); 0	1 (1, 1); 0
Sarilumab			
Received ≥1 dose, n (%)	2 (3.4)	5 (7.8)	2 (3.2)
Median (IQR) time since initiation (days); n missing	1 (0, 1); 0	0 (0, 1); 0	1 (0, 1); 0
Median (IQR) duration (days), n missing	1 (1, 1); 0	1 (1, 1); 0	1 (1, 1); 0
Any IL6 therapy			
Received ≥1 dose, n (%)	11 (19.0)	13 (20.3)	9 (14.3)
Median (IQR) time since initiation (days); n missing	0 (0, 1); 0	0 (0, 1); 0	0 (0, 1); 0
Median (IQR) duration (days), n missing	1 (1, 1); 0	1 (1, 1); 0	1 (1, 1); 0
Baricitinib			
Received ≥1 dose, n (%)	0 (0.0)	0 (0.0)	0 (0.0)
Median (IQR) time since initiation (days); n missing	N/A	N/A	N/A
Median (IQR) duration (days), n missing	N/A	N/A	N/A

N/A: not applicable

For peer review only

Table S3 Standard of care treatments after randomisation by treatment arm

Drug group	FOS (N=58)	RUX (N=64)	SOC (N=63)
Anti-viral agents			
Received ≥1 dose, n (%)	3 (5.2)	3 (4.7)	3 (4.8)
Median (IQR) time since initiation (days); n missing	10 (6, 11); 0	1 (1, 3); 0	1 (1, 1); 0
Median (IQR) duration (days); n missing	1 (1, 4); 0	5 (5, 5); 0	5 (5, 6); 0
Immuno-modulating agents			
Received ≥1 dose, n (%)	14 (24.1)	13 (20.3)	16 (25.4)
Median (IQR) time since initiation (days); n missing	8 (5, 10); 0	9 (2, 14); 0	3 (1, 5); 0
Median (IQR) duration (days); n missing	18 (2, 22); 2	8 (1, 11); 0	3 (1, 24); 0
Antibiotics			
Received ≥1 dose, n (%)	19 (32.8)	16 (25.0)	14 (22.2)
Median (IQR) time since initiation (days); n missing	7 (4, 13); 0	8 (4, 14); 0	5 (1, 12); 0
Median (IQR) duration (days); n missing	6 (3, 8); 1	7 (3, 13); 0	6 (3, 8); 0
Anticoagulation treatment			
Received ≥1 dose, n (%)	6 (10.3)	7 (10.9)	10 (15.9)
Median (IQR) time since initiation (days); n missing	10 (2, 19); 0	5 (3, 8); 0	8 (3, 10); 0
Median (IQR) duration (days); n missing	20 (9, 31); 1	8 (2, 22); 0	18 (5, 28); 3
Antiplatelet therapy			
Received ≥1 dose, n (%)	3 (5.2)	2 (3.1)	2 (3.2)
Median (IQR) time since initiation (days); n missing	1 (1, 2); 0	6 (1, 11); 0	2 (2, 2); 0
Median (IQR) duration (days); n missing	4 (4, 27); 0	16 (16, 16); 1	29 (29, 29); 0
Convalescent serum therapy			
Received ≥1 dose, n (%)	0 (0.0)	2 (3.1)	1 (1.6)
Median (IQR) time since initiation (days); n missing	N/A	1 (1, 2); 0	1 (1, 1); 0
Median (IQR) duration (days); n missing	N/A	2 (1, 2); 0	2 (2, 2); 0
Ronapreve®			
Received ≥1 dose, n (%)	3 (5.2)	2 (3.1)	2 (3.2)
Median (IQR) time since initiation (days); n missing	2 (1, 5); 0	2 (1, 3); 0	2 (1, 2); 0
Median (IQR) duration (days); n missing	1 (1, 1); 0	1 (1, 1); 0	1 (1, 1); 0

NA: not applicable

For peer review only

Table S4 Selected immunological therapies after randomisation by treatment arm

Drug	FOS (N=58)	RUX (N=64)	SOC (N=63)
Dexamethasone			
Received ≥1 dose, n (%)	0 (0.0)	2 (3.1)	4 (6.3)
Median (IQR) time since initiation (days); n missing	N/A	1 (1, 1); 0	2 (1, 3); 0
Median (IQR) duration (days), n missing	N/A	5 (4, 5); 0	17 (4, 29); 0
Prednisolone			
Received ≥1 dose, n (%)	6 (10.3)	4 (6.3)	2 (3.2)
Median (IQR) time since initiation (days); n missing	8 (8, 10); 0	16 (14, 22); 0	13 (13, 14); 0
Median (IQR) duration (days), n missing	22 (18, 25); 2	12 (8, 15); 0	24 (20, 24); 0
Methylprednisolone			
Received ≥1 dose, n (%)	3 (5.2)	2 (3.1)	0 (0.0)
Median (IQR) time since initiation (days); n missing	3 (1, 4); 0	3 (3, 3); 0	N/A
Median (IQR) duration (days), n missing	3 (1, 4); 0	3 (3, 3); 0	N/A
Hydrocortisone			
Received ≥1 dose, n (%)	3 (5.2)	1 (1.6)	1 (1.6)
Median (IQR) time since initiation (days); n missing	7 (5, 16); 0	8 (8, 8); 0	5 (5, 5); 0
Median (IQR) duration (days), n missing	2 (1, 3); 0	1 (1, 1); 0	3 (3, 3); 0
Tocilizumab			
Received ≥1 dose, n (%)	4 (6.9)	6 (9.4)	9 (14.3)
Median (IQR) time since initiation (days); n missing	2 (1, 8); 0	2 (1, 2); 0	3 (1, 4); 0
Median (IQR) duration (days), n missing	1 (1, 1); 0	1 (1, 1); 0	1 (1, 2); 0
Sarilumab			
Received ≥1 dose, n (%)	0 (0.0)	0 (0.0)	0 (0.0)
Median (IQR) time since initiation (days); n missing	N/A	N/A	N/A
Median (IQR) duration (days), n missing	N/A	N/A	N/A
Any IL6 therapy			
Received ≥1 dose, n (%)	4 (6.9)	6 (9.4)	9 (14.3)
Median (IQR) time since initiation (days); n missing	2 (1, 8); 0	2 (1, 2); 0	3 (1, 4); 0
Median (IQR) duration (days), n missing	1 (1, 1); 0	1 (1, 1); 0	1 (1, 2); 0
Baricitinib			
Received ≥1 dose, n (%)	0 (0.0)	0 (0.0)	1 (1.6)
Median (IQR) time since initiation (days); n missing	N/A	N/A	11 (11, 11); 0
Median (IQR) duration (days), n missing	N/A	N/A	11 (11, 11); 0

N/A: not applicable

For peer review only

Supplementary Appendix 4

Co-enrolment to other randomised controlled trials

For peer review only

Table S5: Co-enrolment to other randomised clinical trials by treatment arm

Patient characteristic, n(%) unless otherwise specified	Fostamatinib (N=58)	Ruxolitinib (N=64)	Standard of care (N=63)
Co-enrolled in another study pre-randomisation n (%)			
Any randomised study	5 (8.6)	4 (6.3)	5 (7.9)
RECOVERY	3 (5.2)	3 (4.7)	2 (3.2)
REMAP-CAP	1 (1.7)	1 (1.6)	0 (0.0)
NOCoV2	0 (0.0)	0 (0.0)	1 (1.6)
C19-ACS	1 (1.7)	0 (0.0)	2 (3.2)
Co-enrolled in another study post-randomisation n (%)			
Any randomised study)	2 (3.4)	6 (9.4)	7 (11.1)
RECOVERY	1 (1.7)	1 (1.6)	3 (4.8)
REMAP-CAP	1 (1.7)	2 (3.1)	2 (3.2)
NOCoV2	0 (0.0)	2 (3.1)	2 (3.2)
C19-ACS	0 (0.0)	1 (1.6)	1 (1.6)

For peer review only

Supplementary Appendix 5

Missing data methods for the primary model and results using alternative assumptions

For peer review only

In the primary analysis model, we imputed missing WHO COVID-19 status using multiple imputation under the assumption of missing at random (MAR). That is the probability of being missing is not dependent on the values of unobserved data, conditional on the observed values of the variables included in the analysis model. We imputed *m* datasets using chained equations based on the percentage of participants with incomplete outcomes, where *m* was rounded to the nearest 10.[11] As outcome data were classified as missing in 6% of patients, an m=10 was used.

Variables in the imputation model included baseline WHO COVID-19 grade (3 or 4), age (<65 or ≥65), receipt of systemic corticosteroid and/or receipt of an IL6 inhibitor (at the time of or prior to randomisation into MATIS) and prior COVID vaccination. Auxiliary variables suspected to be associated with the outcome and to be predictors of missingness included receipt of any effective COVID-19 treatments (steroids or IL6 inhibitors) post-randomisation (>1 day post-randomisation) and WHO COVID-19 severity grades at day 1 and day 7.

Convergence issues were encountered when running the imputation model separately by treatment arm (the preferred approach).[11] Missing primary outcomes were therefore imputed using the full dataset with treatment arm included as an additional variable. The final analysis mode; was run omitting prior use of steroids as a covariate due to instability results from zero event counts within strata.

Alternative missing data assumptions were also considered: 1) all participants with missing outcome had poor outcome e.g. all missing >=5, and 2): that all participants with missing outcome had good outcome e.g. all missing <5 were also conducted (Table S5).

For peer review only

Table S6: Primary Outcome Statistical Model Results with Alternative Missingness Assumptions

Grade >=5 by Day 14	Adjusted odds ratio (95% CI; p-value[a])	
	Fostamatinib vs SOC	Ruxolitinib vs SOC
Sensitivity analyses		
Assume all missing have poor outcome	1.08 (0.49 to 2.40); 0.847	0.73 (0.32 to 1.67); 0.453
Assume all missing have good outcome	1.17 (0.51 to 2.69); 0.710	0.63 (0.26 to 1.57); 0.323

[a] p-values are two-sided

[b] adjusted for baseline COVID severity, age category, use of steroid (where model allows), use of IL6 inhibitor and prior COVID vaccination

For peer review only

Supplementary Appendix 6

Secondary Outcomes

For peer review only

Table S7: Secondary outcomes: Grade >=5 by Day 28

	N	n (%)	Adjusted odds ratio[a] (95% CI)
**Grade >=5 by Day 28[b]**			
Fostamatinib	58	16 (27.6)	1.17 (0.51 to 2.69)
Ruxolitinib	62	11 (17.7)	0.70 (0.29 to 1.72)
SOC	61	15 (24.6)	REF

[a] adjusted for baseline COVID severity, age category, use of IL6 inhibitor and prior COVID vaccination

[b] multiple imputation used to input missing outcome status for 4 patients in each arm

For peer review only

Table S8: Secondary outcomes up to Day 14

	N	n (%)	Adjusted odds ratio* (95% CI)
**Individual components of modified WHO scale**			
O2 saturation < 90% on ≥ 60% inspired oxygen			
Fostamatinib	55	16 (29.1)	1.84 (0.75 to 4.56)
Ruxolitinib	58	9 (15.5)	0.87 (0.32 to 2.37)
SOC	57	11 (19.3)	REF
Non-invasive ventilation			
Fostamatinib	55	13 (23.6)	0.95 (0.39 to 2.28)
Ruxolitinib	58	9 (15.5)	0.61 (0.24 to 1.56)
SOC	57	14 (24.6)	REF
Mechanical ventilation			
Fostamatinib	55	4 (7.3)	2.01 (0.35 to 11.66)
Ruxolitinib	58	3 (5.2)	1.58 (0.25 to 10.09)
SOC	57	2 (3.5)	REF
Mechanical ventilation with organ support			
Fostamatinib	54	3 (5.6)	3.02 (0.30 to 30.71)
Ruxolitinib	58	2 (3.4)	2.00 (0.17 to 23.44)
SOC	57	1 (1.8)	REF
Death			
Fostamatinib	55	4 (7.4)	1.60 (0.32 to 8.01)
Ruxolitinib	58	0 (0.0)	Not estimable
SOC	57	3 (5.5)	REF
**Maximum severity grade by Day 14**			
Grade 5			
Fostamatinib	55	1 (1.8)	Not estimable
Ruxolitinib	58	1 (1.7)	Not estimable
SOC	57	0 (0.0)	REF
Grade 6			
Fostamatinib	55	7 (12.7)	0.60 (0.21 to 1.69)
Ruxolitinib	58	6 (10.3)	0.52 (0.17 to 1.54)
SOC	57	11 (19.3)	REF
Grade 7			
Fostamatinib	55	1 (1.8)	0.95 (0.06 to 16.04)
Ruxolitinib	58	1 (1.7)	1.16 (0.07 to 19.73)
SOC	57	1 (1.8)	REF
Grade 8			
Fostamatinib	55	3 (5.5)	Not estimable
Ruxolitinib	58	2 (3.4)	Not estimable
SOC	57	0 (0.0)	REF
Grade 9			
Fostamatinib	55	4 (7.3)	1.60 (0.32 to 8.01)
Ruxolitinib	58	0 (0.0)	Not estimable
SOC	57	3 (5.3)	REF

*adjusted for baseline COVID severity, age category, use of IL6 inhibitor and prior COVID vaccination

For peer review only

Table S9: Individual components of modified WHO scale up to Day 28

Component of modified WHO scale	N	n (%)	Adjusted odds ratio[a] (95% CI)
O2 saturation < 90% on ≥ 60% inspired oxygen			
Fostamatinib	54	16 (29.6)	1.94 (0.78 to 4.82)
Ruxolitinib	58	10 (17.2)	0.97 (0.37 to 2.58)
SOC	57	11 (19.3)	REF
Non-invasive ventilation			
Fostamatinib	54	13 (24.1)	0.99 (0.41 to 2.37)
Ruxolitinib	58	10 (17.2)	0.68 (0.27 to 1.70)
SOC	57	14 (24.6)	REF
Mechanical ventilation			
Fostamatinib	54	4 (7.4)	1.95 (0.33 to 11.32)
Ruxolitinib	58	3 (5.2)	1.51 (0.24 to 9.71)
SOC	55	2 (3.6)	REF
Mechanical ventilation with organ support			
Fostamatinib	53	3 (5.7)	2.95 (0.29 to 30.05)
Ruxolitinib	57	3 (5.3)	3.14 (0.31 to 32.22)
SOC	55	1 (1.8)	REF
Death			
Fostamatinib	54	5 (9.3)	2.44 (0.51 to 11.71)
Ruxolitinib	58	2 (3.4)	0.67 (0.10 to 4.37);
SOC	55	3 (5.5)	REF

[a] adjusted for baseline COVID severity, age category, use of IL6 inhibitor and prior COVID vaccination

[b] multiple imputation used to input missing outcome status for 4 patients in each arm

For peer review only

Table S10: Maximum severity grade by Day 28

Maximum severity grade by Day 28	N	n (%)	Adjusted odds ratio[a] (95% CI)
Grade 5			
Fostamatinib	54	1 (1.9)	Not estimable
Ruxolitinib	58	1 (1.7)	Not estimable
SOC	55	0 (0.0)	REF
Grade 6			
Fostamatinib	54	7 (13.0)	0.76 (0.26 to 2.27)
Ruxolitinib	58	6 (10.3)	0.66 (0.21 to 2.04)
SOC	55	9 (16.4)	REF
Grade 7			
Fostamatinib	54	1 (1.9)	0.90 (0.05 to 15.48)
Ruxolitinib	58	0 (0.0)	Not estimable
SOC	55	1 (1.8)	REF
Grade 8			
Fostamatinib	54	2 (3.7)	Not estimable
Ruxolitinib	58	2 (3.4)	Not estimable
SOC	55	0 (0.0)	REF
Grade 9			
Fostamatinib	54	5 (9.3)	2.44 (0.51 to 11.71)
Ruxolitinib	58	2 (3.4)	0.67 (0.10 to 4.37)
SOC	55	3 (5.5)	REF

[a] adjusted for baseline COVID severity, age category, use of IL6 inhibitor and prior COVID vaccination

[b] multiple imputation used to input missing outcome status for 4 patients in each arm

Table S11: Change in odds of progressing to a worse severity between trial arms

Outcome definition	Adjusted[a] odds ratio (95% CI two-sided p-value)	
	Fostamatinib vs SOC	Ruxolitinib vs SOC
COVID severity at each time point (N=180[b])		
Day 1	1.00 (0.35 to 2.82); 0.994	0.68 (0.25 to 1.91); 0.469
Day 7	0.94 (0.31 to 2.89); 0.915	0.51 (0.16 to 1.57); 0.239
Day 14	2.37 (0.67 to 8.39); 0.182	0.77 (0.21 to 2.78); 0.689

[a] Adjusted for age category, baseline severity, use of steroid at baseline, use of IL6 inhibitor at baseline, received covid vaccine

[b] Includes N=180 ie all patients with at least one post-baseline outcome measurement

For peer review only

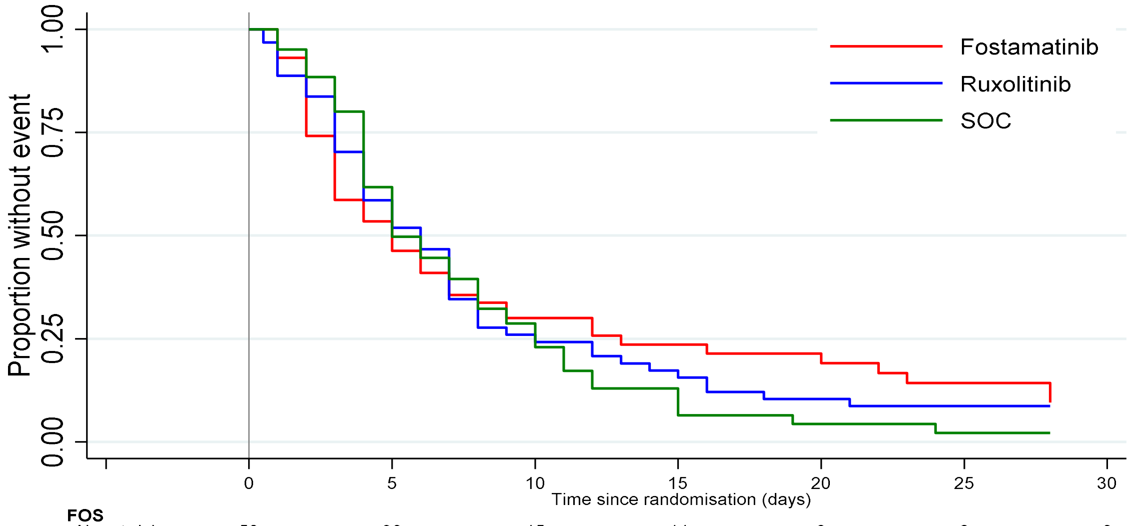

	0	5	10	15	20	25	30
**FOS**							
No. at-risk	58	30	15	11	9	6	0
Censored	0	1	3	4	5	5	9
Discharged	0	27	40	43	44	47	49
**RUX**							
No. at-risk	62	35	15	10	6	4	0
Censored	0	2	3	3	3	4	8
Discharged	0	25	44	49	53	54	54
**SOC**							
No. at-risk	61	36	15	6	2	1	0
Censored	0	2	4	5	5	5	6
Discharged	0	23	42	50	54	55	55

Figure S1: Kaplan Meier estimates of time to discharge (from randomisation) by treatment arm, censored at day 28[a]

[a] withdrawals and deaths are censored at the date of occurrence

Table S12: Readmissions by Day 28

Trial arm	N	n (%)	Adjusted odds ratio[a] (95% CI)
Fostamatinib	54	3 (5.6)	Not estimated
Ruxolitinib	58	8 (13.8)	Not estimated
SOC	55	8 (14.6)	Not estimated

For peer review only - http://bmjopen.bmj.com/site/about/guidelines.xhtml

Table S13: Results from mixed effects linear regression for inflammatory markers and serum creatinine

Marker	Fostamatinib vs SOC		Ruxolitinib vs SOC	
	Adjusted[a] mean difference	95% CI two-sided p-value	Adjusted[a] mean difference	95% CI two-sided p-value
**CRP (mg/L)**				
Day 1	-6.2	-22.4 to 10.0; 0.452	-7.7	-23.8 to 8.4; 0.350
Day 7	9.4	-8.2 to 27.0; 0.294	-11.9	-29.1 to 5.3; 0.176
Day 14	18.2	-6.1 to 42.5; 0.141	-6.8	-30.0 to16.3; 0.562
Day 28	3.7	-19.7 to 27.2; 0.755	-18.3	-40.5 to 3.8; 0.105
**LDH (IU/L)**				
Day 1	-11.8	-81.2 to 57.6; 0.739	-33.0	-106.7 to 40.7; 0.380
Day 7	39.5	-31.9 to 110.8; 0.278	56.6	-17.9 to 131.2; 0.136
Day 14	9.6	-84.5 to 103.7; 0.841	-34.7	-137.3 to 67.8; 0.507
Day 28	97.3	3.7 to 190.8; 0.042	54.3	-42.8 to 151.4; 0.273
**Ferritin (ug/L)**				
Day 1	278	-447 to 1003; 0.452	-101	-826 to 624; 0.784
Day 7	227	-487 to 941; 0.533	35	-668 to 739; 0.921
Day 14	29	-701 to 759; 0.938	-403	-1121 to 316; 0.272
Day 28	-20	-782 to 742; 0.959	-357	-1103 to 388; 0.347
**D-dimer (ng/ml)**				
Day 1	-360	-1191 to 471; 0.396	-97	-964 to 770; 0.826
Day 7	-487	-1325 to 351; 0.255	22	-808 to 853; 0.958
Day 14	85	-891 to 1062; 0.864	80	-864 to 1023; 0.869
Day 28	615	-388 to 1619; 0.229	401	-568 to 1370; 0.417
**Creatinine (µmol/L)[b]**				
Day 1	-10.1	-29.8 to 9.7; 0.317	-10.1	-29.7 to 9.5; 0.312
Day 7	12.3	-8.9 to 33.5; 0.255	16.1	-4.9 to 37.2; 0.133
Day 14	34.3	6.8 to 61.8; 0.014	21.2	-5.4 to 47.9; 0.119
Day 28	43.6	15.7 to 71.5; 0.002	27.2	0.6 to 53.7; 0.045

[a] adjusted for baseline value, site, use of steroid at baseline, use of IL6 inhibitor at baseline, age category and receipt of COVID vaccine; [b] analysis excludes three patients in RUX arm receiving dialysis

For peer review only

Supplementary Appendix 7

Exploratory analyses

For peer review only

Table S14: Primary outcome model results in patients who received ≥ 90% of prescribed dose

Per-protocol	No. (%) of compliers	Adjusted[a] odds ratio (95% CI; p-value[b])	
		ITT	Per-protocol
Fostamatinib	40 (69.0)	1.19 (0.51 to 2.76); 0.681	1.19 (0.47 to 2.99); 0.716
Ruxolitinib	46 (74.2)	0.63 (0.25 to 1.57); 0.323	0.80 (0.31 to 2.06); 0.641
SOC	61 (100.0)	REF	REF
**CACE**	**No. (%) of compliers**	**Adjusted[a] risk difference (95% CI; p-value[b])**	
		**ITT**	**CACE**
Fostamatinib	40 (69.0)	0.03 (-0.12 to 0.17); 0.729	0.05 (-0.18 to 0.28); 0.642
Ruxolitinib	46 (74.2)	-0.06 (-0.17 to 0.06); 0.328	-0.10 (-0.29 to 0.10); 0.328
SOC	61 (100.0)	REF	REF

[a] adjusted for baseline COVID severity, age category, use of IL6 inhibitor, prior COVID vaccination; model run on imputed dataset

[b] p-values are two-sided

Table S15: Primary outcome model results stratified by time of randomisation

Grade >=5 by Day 14	N			Odds ratio (95% CI; p-value[a])	
	FOS	RUX	SOC	Fostamatinib vs SOC	Ruxolitinib vs SOC
Stratified analysis by time of enrolment					
Quartile 1 (Jan 2021)	16	15	15	0.45 (0.10 to 2.01; 0.299)	0.44 (0.09 to 2.15; 0.313)
Quartile 2 (Feb 2021)	15	16	14	4.71 (0.73 to 30.28; 0.102)	1.50 (0.21 to 10.81; 0.687)
Quartile 3 (Sep 2021)	15	15	15	Not estimable	0.92 (0.15 to 5.53; 0.924)
Quartile 4 (Aug 2022)	12	16	17	3.10 (0.56 to 16.96; 0.193)	Not estimable

a p-values are two-sided

For peer review only

Supplementary Appendix 8

Adverse Events by System Organ Class

For peer review only

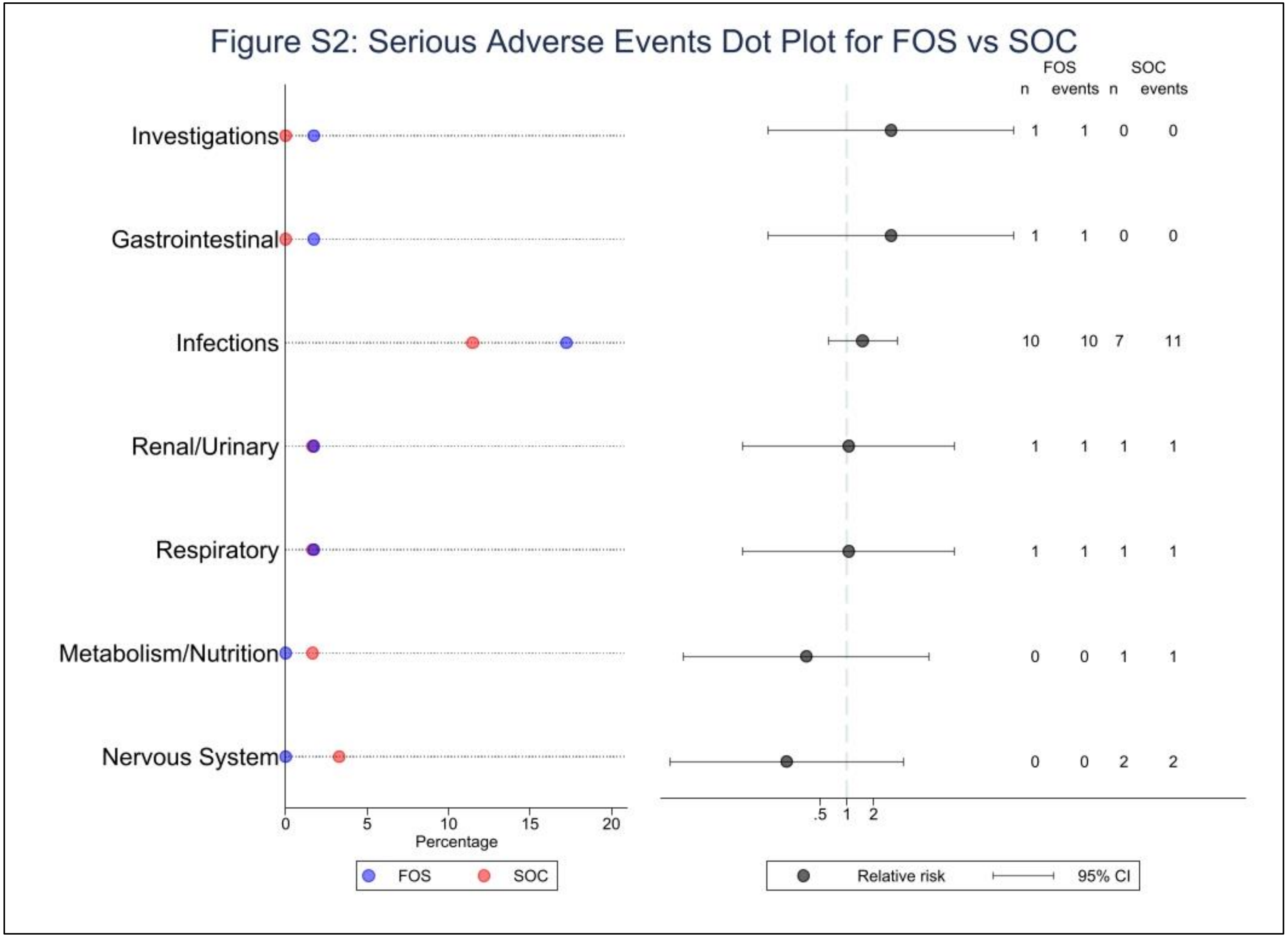
Figure S2: Serious Adverse Events Dot Plot for FOS vs SOC
FOS
SOC
n
events
n
events
Investigations
Gastrointestinal
Infections
Renal/Urinary
Respiratory
Metabolism/Nutrition
Nervous System
1 1 0 0
1 1 0 0
10 10 7 11
1 1 1 1
1 1 1 1
0 0 1 1
0 0 2 2
0
5
10
15
20
Percentage
.5
1
2
FOS
SOC
Relative risk
95% CI

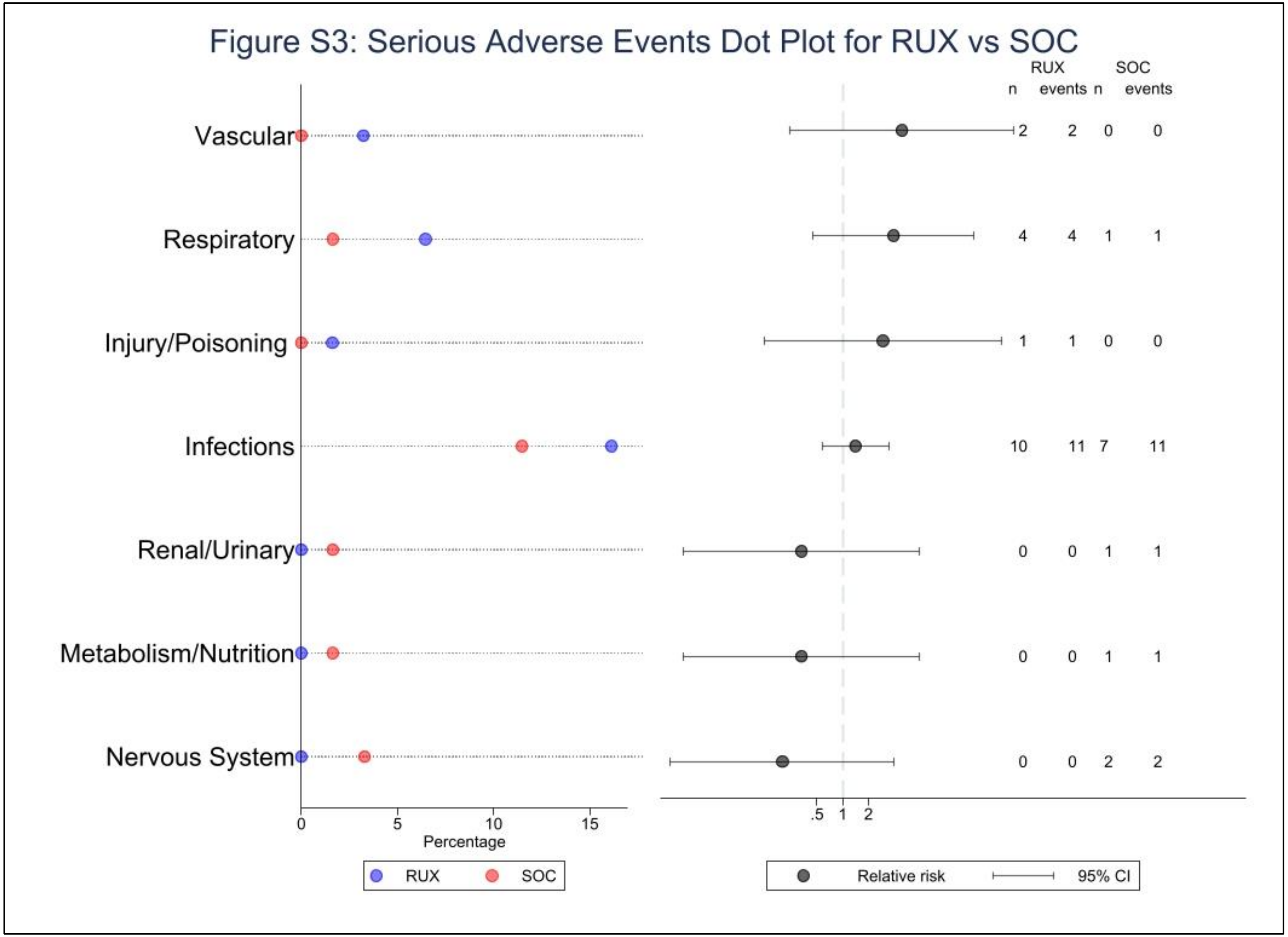
Figure S3: Serious Adverse Events Dot Plot for RUX vs SOC
RUX
SOC
n
events
n
events
Vascular
2 2 0 0
Respiratory
4 4 1 1
Injury/Poisoning
1 1 0 0
Infections
10 11 7 11
Renal/Urinary
0 0 1 1
Metabolism/Nutrition
0 0 1 1
Nervous System
0 0 2 2
0
5
10
15
Percentage
.5
1
2
RUX
SOC
Relative risk
95% CI

Supplementary Appendix 9

Subgroup Analysis

For peer review only

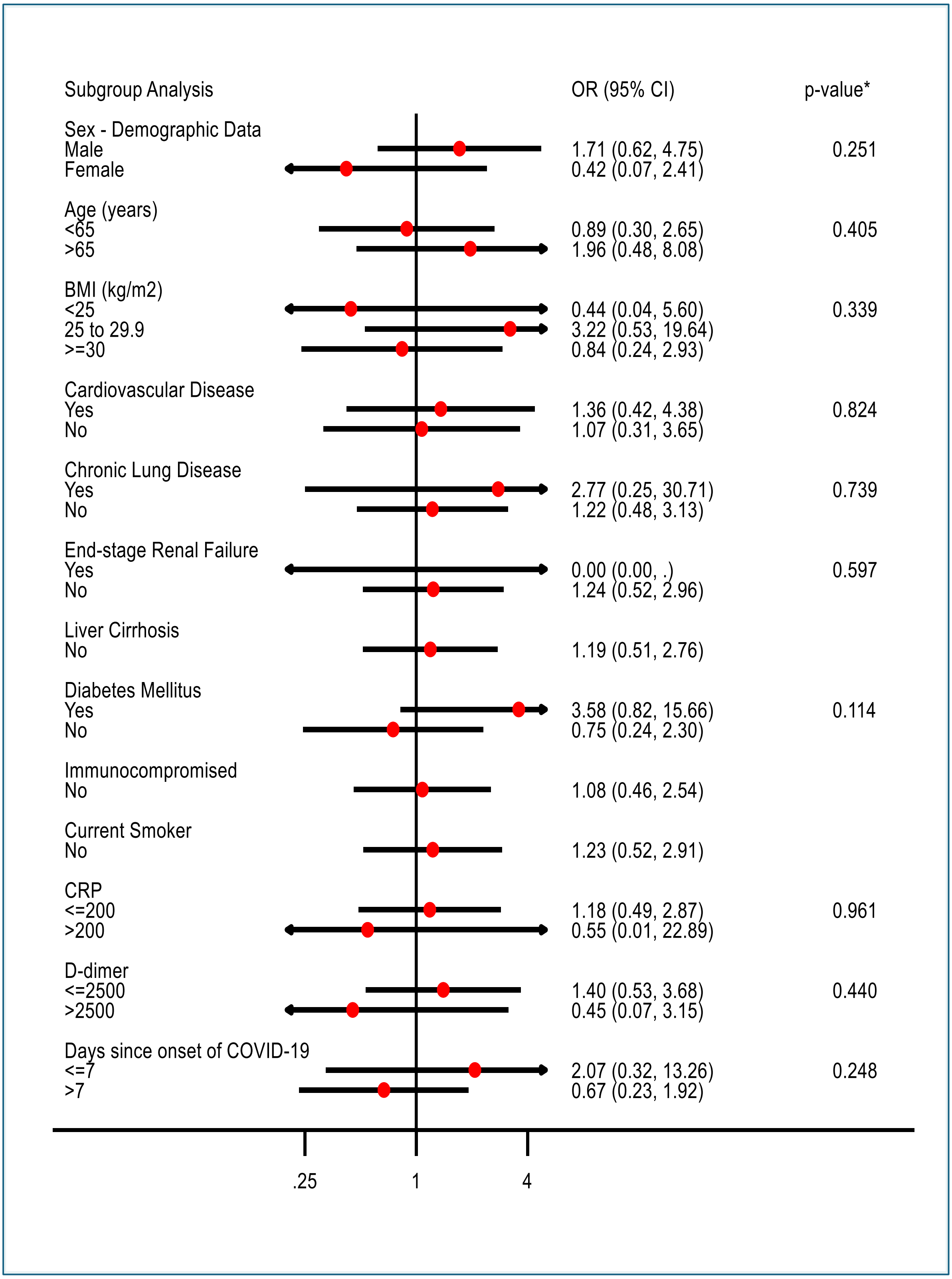

*p value for test of interaction

Figure S4: Subgroup analysis of primary outcome for fostamatinib versus SOC

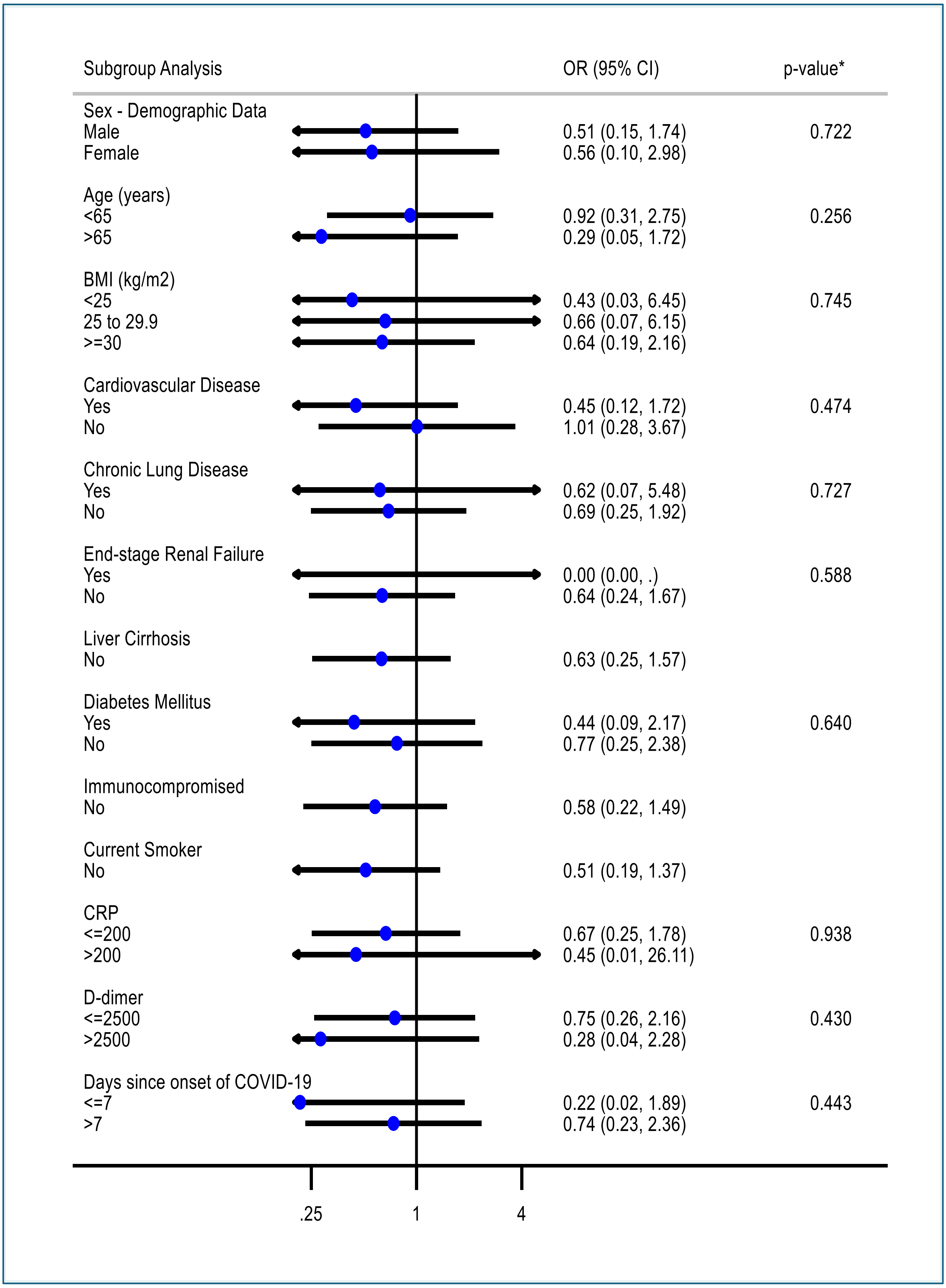

*p value for test of interaction

Figure S4: Subgroup analysis of primary outcome for ruxolitinib versus SOC
